# Reactogenicity and immunogenicity after a late second dose or a third dose of ChAdOx1 nCoV-19 in the UK: a substudy of two randomised controlled trials (COV001 and COV002)

**DOI:** 10.1016/S0140-6736(21)01699-8

**Published:** 2021-09-11

**Authors:** Amy Flaxman, Natalie G Marchevsky, Daniel Jenkin, Jeremy Aboagye, Parvinder K Aley, Brian Angus, Sandra Belij-Rammerstorfer, Sagida Bibi, Mustapha Bittaye, Federica Cappuccini, Paola Cicconi, Elizabeth A Clutterbuck, Sophie Davies, Wanwisa Dejnirattisai, Christina Dold, Katie J Ewer, Pedro M Folegatti, Jamie Fowler, Adrian V S Hill, Simon Kerridge, Angela M Minassian, Juthathip Mongkolsapaya, Yama F Mujadidi, Emma Plested, Maheshi N Ramasamy, Hannah Robinson, Helen Sanders, Emma Sheehan, Holly Smith, Matthew D Snape, Rinn Song, Danielle Woods, Gavin Screaton, Sarah C Gilbert, Merryn Voysey, Andrew J Pollard, Teresa Lambe, Oxford COVID Vaccine Trial group, Syed Adlou, Robert Aley, Aabidah Ali, Rachel Anslow, Megan Baker, Phillip Baker, Jordan R. Barrett, Louise Bates, Kirsten Beadon, Rebecca Beckley, Jonathan Bell, Duncan Bellamy, Amy Beveridge, Cameron Bissett, Luke Blackwell, Heather Bletchly, Amy Boyd, Alice Bridges-Webb, Charlie Brown, Nicholas Byard, Susana Camara, Liliana Cifuentes Gutierrez, Andrea M. Collins, Rachel Cooper, Wendy E.M. Crocker, Thomas C. Darton, Hannah Davies, Judith Davies, Tesfaye Demissie, Claudio Di Maso, Tanya Dinesh, Francesca R. Donnellan, Alexander D. Douglas, Rachael Drake-Brockman, Christopher J.A. Duncan, Sean C. Elias, Katherine R.W. Emary, Mutjaba Ghulam Farooq, Saul N. Faust, Sally Felle, Daniela Ferreira, Carla Ferreira Da Silva, Adam Finn, Karen J. Ford, Emma Francis, Julie Furze, Michelle Fuskova, Eva Galiza, Ana Gibertoni Cruz, Leila Godfrey, Anna L. Goodman, Catherine Green, Christopher A. Green, Nicola Greenwood, Daisy Harrison, Thomas C. Hart, Sophia Hawkins, Paul T. Heath, Helen Hill, Kushalinii Hillson, Bryn Horsington, Mimi M. Hou, Elizabeth Howe, Nicola Howell, Carina Joe, Elizabeth Jones, Mwila Kasanyinga, Jade Keen, Sarah Kelly, David Kerr, Liaquat Khan, Baktash Khozoee, Jasmin Kinch, Patrick Kinch, Stanislava Koleva, Jonathan Kwok, Colin W. Larkworthy, Alison M. Lawrie, Rajeka Lazarus, Emily A. Lees, Grace Li, Vincenzo Libri, Patrick J. Lillie, Aline Linder, Fei Long, Raquel Lopez Ramon, Reece Mabbett, Rebecca Makinson, Spyridoula Marinou, Emma Marlow, Julia L. Marshall, Olga Mazur, Joanne McEwan, Alastair C. McGregor, Jolynne Mokaya, Ella Morey, Gertraud Morshead, Richard Morter, Jilly Muller, Philomena Mweu, Rabiullah Noristani, Nelly Owino, Marco Polo Peralta Alvarez, Abigail Platt, Katrina M. Pollock, Ian Poulton, Samuel Provstgaard-Morys, David Pulido-Gomez, Matthew Rajan, Fernando Ramos Lopez, Adam Ritchie, Hannah Roberts, Christine Rollier, Indra Rudiansyah, Katherine Sanders, Jack E. Saunders, Samiullah Seddiqi, Hannah R. Sharpe, Robert Shaw, Laura Silva-Reyes, Nisha Singh, David J. Smith, Catherine C. Smith, Andrew Smith, Alexandra J. Spencer, Arabella S.V. Stuart, Rebecca Sutherland, Anna Szigeti, Karly Tang, Merin Thomas, Tonia M. Thomas, Amber Thompson, Emma C. Thomson, Estée M. Török, Mark Toshner, Nguyen Tran, Rose Trivett, Iain Turnbull, Cheryl Turner, David P.J. Turner, Marta Ulaszewska, Iason Vichos, Laura Walker, Marion E. Watson, Conor Whelan, Rachel White, Sarah J. Williams, Christopher J.A. Williams, Daniel Wright, Andy Yao

**Affiliations:** a Jenner Institute, Nuffield Department of Medicine, University of Oxford, Oxford, UK; b Oxford Vaccine Group, Department of Paediatrics, University of Oxford, Oxford, UK; c Chinese Academy of Medical Science (CAMS) Oxford Institute, University of Oxford, Oxford, UK; d NIHR Oxford Biomedical Research Centre, Oxford, UK; e Wellcome Centre for Human Genetics, University of Oxford, Oxford, UK

## Abstract

**Background:**

COVID-19 vaccine supply shortages are causing concerns about compromised immunity in some countries as the interval between the first and second dose becomes longer. Conversely, countries with no supply constraints are considering administering a third dose. We assessed the persistence of immunogenicity after a single dose of ChAdOx1 nCoV-19 (AZD1222), immunity after an extended interval (44–45 weeks) between the first and second dose, and response to a third dose as a booster given 28–38 weeks after the second dose.

**Methods:**

In this substudy, volunteers aged 18–55 years who were enrolled in the phase 1/2 (COV001) controlled trial in the UK and had received either a single dose or two doses of 5 × 10^10^ viral particles were invited back for vaccination. Here we report the reactogenicity and immunogenicity of a delayed second dose (44–45 weeks after first dose) or a third dose of the vaccine (28–38 weeks after second dose). Data from volunteers aged 18–55 years who were enrolled in either the phase 1/2 (COV001) or phase 2/3 (COV002), single-blinded, randomised controlled trials of ChAdOx1 nCoV-19 and who had previously received a single dose or two doses of 5 × 10^10^ viral particles are used for comparison purposes. COV001 is registered with ClinicalTrials.gov, NCT04324606, and ISRCTN, 15281137, and COV002 is registered with ClinicalTrials.gov, NCT04400838, and ISRCTN, 15281137, and both are continuing but not recruiting.

**Findings:**

Between March 11 and 21, 2021, 90 participants were enrolled in the third-dose boost substudy, of whom 80 (89%) were assessable for reactogenicity, 75 (83%) were assessable for evaluation of antibodies, and 15 (17%) were assessable for T-cells responses. The two-dose cohort comprised 321 participants who had reactogenicity data (with prime-boost interval of 8–12 weeks: 267 [83%] of 321; 15–25 weeks: 24 [7%]; or 44–45 weeks: 30 [9%]) and 261 who had immunogenicity data (interval of 8–12 weeks: 115 [44%] of 261; 15–25 weeks: 116 [44%]; and 44–45 weeks: 30 [11%]). 480 participants from the single-dose cohort were assessable for immunogenicity up to 44–45 weeks after vaccination. Antibody titres after a single dose measured approximately 320 days after vaccination remained higher than the titres measured at baseline (geometric mean titre of 66·00 ELISA units [EUs; 95% CI 47·83–91·08] *vs* 1·75 EUs [1·60–1·93]). 32 participants received a late second dose of vaccine 44–45 weeks after the first dose, of whom 30 were included in immunogenicity and reactogenicity analyses. Antibody titres were higher 28 days after vaccination in those with a longer interval between first and second dose than for those with a short interval (median total IgG titre: 923 EUs [IQR 525–1764] with an 8–12 week interval; 1860 EUs [917–4934] with a 15–25 week interval; and 3738 EUs [1824–6625] with a 44–45 week interval). Among participants who received a third dose of vaccine, antibody titres (measured in 73 [81%] participants for whom samples were available) were significantly higher 28 days after a third dose (median total IgG titre: 3746 EUs [IQR 2047–6420]) than 28 days after a second dose (median 1792 EUs [IQR 899–4634]; Wilcoxon signed rank test p=0·0043). T-cell responses were also boosted after a third dose (median response increased from 200 spot forming units [SFUs] per million peripheral blood mononuclear cells [PBMCs; IQR 127–389] immediately before the third dose to 399 SFUs per milion PBMCs [314–662] by day 28 after the third dose; Wilcoxon signed rank test p=0·012). Reactogenicity after a late second dose or a third dose was lower than reactogenicity after a first dose.

**Interpretation:**

An extended interval before the second dose of ChAdOx1 nCoV-19 leads to increased antibody titres. A third dose of ChAdOx1 nCoV-19 induces antibodies to a level that correlates with high efficacy after second dose and boosts T-cell responses.

**Funding:**

UK Research and Innovation, Engineering and Physical Sciences Research Council, National Institute for Health Research, Coalition for Epidemic Preparedness Innovations, National Institute for Health Research Oxford Biomedical Research Centre, Chinese Academy of Medical Sciences Innovation Fund for Medical Science, Thames Valley and South Midlands NIHR Clinical Research Network, AstraZeneca, and Wellcome.

## Introduction

The COVID-19 pandemic continues to put a substantial burden on health-care systems and a massive global effort is underway to protect populations through vaccination. COVID-19 vaccine supply shortages in many countries are causing concern about compromised immunity as the interval between the first and second dose extends beyond 12 weeks.^1^ WHO recommends that the second dose of the ChAdOx1 nCoV-19 vaccine is given 8–12 weeks after the first dose because the clinical trial data provide support for good levels of protection with this interval;^2^, ^3^ however, many countries cannot obtain sufficient supplies to allow second doses to be administered by 12 weeks. These supply shortages are leading to longer intervals and uncertainty among policy makers about whether protection against COVID-19^2^, ^3^ will be maintained because no data exist on the efficacy of the immunisation schedules with intervals between the first and second dose that extend beyond this limit.

Conversely, some high-income countries with highly vaccinated populations are considering administration of a third dose of a COVID-19 vaccine because of uncertainty about duration of immunity after the first two doses and the possible risk of breakthrough infection as new variants emerge.

ChAdOx1 nCoV-19 (AZD1222), a replication deficient adenoviral vectored vaccine that encodes the SARS-CoV-2 spike protein, is one of the most widely used vaccines globally. More than half a billion doses have been distributed to more than 168 countries across six continents, including provision through the CoVax facility. Here, we describe tolerability and immune response to a late second dose (44–45 weeks after the first dose) of ChAdOx1 nCoV-19, and after a third dose (28–38 weeks after the second dose). We also report the persistence of antibody and cellular responses at 182 days and for antibodies up to 320 days after first dose of ChAdOx1 nCoV-19.

## Methods

### Study design and participants

In this substudy, we extended the data already collected as part of the UK COV001 and COV002 trials. In these trials, participants were randomly assigned to receive ChAdOx1 nCoV-19 or a meningococcal conjugate vaccine (MenACWY) as a control. Procedures, safety, immune responses, and efficacy before late vaccination and after second dose have been previously published.^2^, ^4^, ^5^ The trials were originally planned as single-dose vaccine studies, but the strong neutralising titres seen in COV001 induced by a second dose of vaccine^4^ prompted a protocol amendment to allow the addition of booster doses to most study participants across both trials. Most participants in both COV001 and COV002 were invited to receive a second dose from July, 2020, onwards. The timing of the second dose varied and allows for comparisons of immunogenicity between the recommended vaccination schedule in the UK of 8–12 weeks and a longer interval of 15–25 weeks.^3^ The initial phase 1 immunogenicity group in COV001 was retained as a single-dose cohort to observe the persistence of immune responses after a single dose.

**Research in context**

**Evidence before this study**

Multiple vaccines against SARS-CoV-2 have now been authorised for use in various countries. Most vaccines are given in a two-dose primary schedule, and further doses might be required to maintain protective immunity or control emerging variants. We searched PubMed for research articles published between database inception and June 23, 2021, using the search terms “SARS-CoV-2”, “vaccine”, “clinical trial”, AND (“third dose” OR “late boost”) with no language restrictions. We identified animal studies using combinations of three-dose vaccine delivery in prime-boost schedules. Additionally, we identified three clinical trials of three-dose delivery, including two in solid organ transplant recipients. In the first study in transplant recipients, antibody titres increased after the third dose of either BNT162b2 (Pfizer–BioNTech) or mRNA-1273 (Moderna) vaccines in a third of patients who had negative antibody titres and in all patients who had low-positive antibody titres. In the second study in transplant recipients, prevalence of antibody titres increased from 44% after a second dose to 68% after a third dose. In a phase 1 and 2 trial of a protein subunit vaccine ZF2001, the safety and immunogenicity data support the use of a 25 μg dose in a three-dose schedule. A number of clinical studies are measuring the effect of a third dose of vaccine, including a phase 1 study of 144 participants who received a homologous third dose of BNT162b2, 6 or 12 months after the second dose.

**Added value of this study**

We report immune responses to ChAdOx1 nCoV-19 following a second dose after an extended interval between the first and second dose, and after a third dose with an extended interval between the second and third dose. The extended interval between the first two doses (44–45 weeks) resulted in higher antibody titres after the second dose than with a shortened interval. A third dose given 28–38 weeks after the primary series increased the antibody titres to above those after a second dose with a shortened interval. Reactogenicity was lower after the second or third dose than after the first dose.

**Implications of all the available evidence**

Vaccine shortages have resulted in some people receiving a first dose of ChAdOx1 nCoV-19 without receiving the second dose within the recommended 4–12 week period. We report that increasing the interval up to 45 weeks results in increased antibody titres after the second dose, offering increased flexibility in vaccination schedules. A third dose at an extended interval after the second dose resulted in a further increase in antibody titres, mitigating concerns that antibodies raised against the ChAdOx1 vector would limit repeated use of the vaccine.

For analysis of immunogenicity after a single dose of ChAdOx1 nCoV-19, we included all participants in COV001 and COV002 who had yet to receive a second dose of vaccine and for whom immunogenicity data were available.

A substudy was added to the COV001 trial as a protocol amendment on March 1, 2021, to investigate the immunogenicity and tolerability of a third dose of the vaccine. Participants who had previously received two doses of ChAdOx1 nCoV-19 were recruited for this substudy, along with control participants, who had received two doses of MenACWY previously, to maintain blinding of reactogenicity data. All recruits received ChAdOx1 nCoV-19; for the control participants this was their first dose. For participants from this three-dose cohort to be eligible for inclusion in these analyses, they had to have an 8–16 week interval between first and second doses.

Some participants from the COV001 single-dose cohort were also offered a second dose at this time. The single dose cohort originally comprised a 1:1 ratio of ChAdOx1 nCoV-19 recipients to MenACWY controls. These participants were invited back in a 2:1 ratio, so participants who had previously received a single dose of ChAdOx1 nCoV-19 received their second dose, with an interval of 44–45 weeks, and those who had been controls received their first dose of ChAdOx1 nCoV-19. Participants were targeted for inclusion in the substudy if they had not previously been unmasked to treatment allocation or offered a vaccine as part of the UK Government COVID-19 vaccine programme. A subset of two-dose recipients (for whom reactogenicity or immunogenicity data, or both, were available) were selected for inclusion in analyses for comparison with those who received two doses 44–45 weeks apart. Participants who had a positive PCR test for SARS-CoV-2 were removed from the analysis if the infection occurred before the blood draw. Participants for this substudy were only enrolled at the Oxford site.

In the UK, the COV001 and COV002 studies were approved by the South Central Berkshire Research Ethics Committee (COV001 reference 20/SC/0145, on March 23, 2020; COV002 reference 20/SC/0179; conditional approval on April 8, 2020, and full approval on April 19, 2020). The protocol for COV001 is provided in appendix 1 and the protocol for COV002 is provided in appendix 2.

### Procedures

Participants who were included as part of this substudy were vaccinated with a standard dose of ChAdOx1 nCoV-19 (5 × 10^10^ viral particles). For control participants who had previously received either one or two doses of MenACWY, this vaccination was their first dose of ChAdOx1 nCoV-19. For participants who had previously received ChAdOx1 nCoV-19, this vaccination was either their second dose (44–45 weeks after the first) or their third dose. These late vaccinations occurred 10 months (plus or minus 56 days) from enrolment. 7 days after vaccination participants were unmasked to treatment allocation, so that those who had received only one dose of ChAdOx1 nCoV-19 could subsequently receive a second dose, in line with national vaccination roll-out in the UK.

Participants enrolled in the substudy had blood samples taken on the day of vaccination, and then at 14 days and 28 days after vaccination to allow immunogenicity assessments to be made.

Binding antibody titres were measured using standardised single dilution total IgG ELISAs as previously described.^5^ This assay was used to measure antibody responses before and after vaccination to Victoria/01/2020 SARS-CoV-2 spike protein and adapted to measure responses to beta (B.1.351) SARS-CoV-2 protein. ELISA assays to Victoria/01/2020 were performed on samples from single-dose recipients up to 1 year after vaccination, in two-dose recipients up to 6 months after the second vaccination, and in three-dose recipients up to 28 days after the third vaccination. ELISA assays on the beta SARS-CoV-2 variant were only done on samples from participants recruited to the substudy who received either a late second vaccination or a third vaccination, up to 28 days after the late vaccination. Meso Scale Discovery multiplex immunoassay was used to assess antibody titres against spike proteins from different variants (Victoria/01/2020, D614G, alpha [B.1.17], beta, and gamma [P.1]). V-PLEX SARS-CoV-2 Panel 6 (IgG) kits were used following manufacturer's instructions (Meso Scale Discovery, K15433U; full details are in appendix 3 [p 1]). Meso Scale Discovery assays were done on samples from participants recruited to the substudy who received a late second dose of vaccine (44–45 weeks after the first dose. Ex-vivo IFN-γ ELISpot assays were done as previously described^5^ to assess T-cell responses to Victoria/01/2020 SARS-CoV-2 spike overlapping peptide pools before and after vaccination. Isolated peripheral blood mononuclear cells (PBMCs) were stimulated overnight with peptides spanning the SARS-CoV-2 spike insert. ELISpot assays were done on samples from participants in the single dose cohort up to 182 days after vaccination. ELISpot assays were also done in some participants (due to laboratory capacity) recruited to the substudy who received a third dose, up to 28 days after third dose. Focus reduction neutralisation assays were done as described previously^6^ to measure neutralising antibody titres against alpha, beta, and delta (B.1.617.2) SARS-CoV-2 viral variants. Neutralisation assays were done in a randomly selected subset of participants (due to laboratory capacity) who received a third dose of vaccine. Timepoints assessed were 28 days after second vaccination and 28 days after third vaccination.

For all immunogenicity assessments, data were excluded upon earliest occurrence of a positive PCR test result or external COVID-19 vaccination. For single dose immunogenicity assessments, data were excluded from after receipt of second dose. For the three-dose cohort, data were included only for those who had an interval of 8–16 weeks between first and second doses.

Participants were asked to complete a diary card for 7 days after each vaccination to record solicited local (induration, itch, pain, redness, swelling, tenderness, and warmth at the injection site) and systemic (chills, fatigue, fever of ≥38°C, feverish [self-reported feeling of feverishness, whereas fever is an objective fever measurement], headache, joint pain, malaise, muscle ache, and nausea) adverse reactions. Participants reported the severity of their adverse reactions as mild, moderate, severe, or life threatening as per definitions provided (appendix 1 pp 92–94).

### Statistical analysis

We present summary statistics for individuals vaccinated with one, two, or three doses of ChAdOx1 nCoV-19 as median (IQR) or geometric mean titre (GMT) with 95% CIs. We do not include data from control participants (who had previously received one or two doses of MenACWY); they received ChAdOx1 nCoV-19 on recruitment to the substudy. For the purposes of ensuring trial personnel were masked to treatment assignment, data for both vaccinnees and controls were collected. Unmasking information was only available to those performing the final data analyses. Upon unmasking of participants and study personnel, control participants were excluded.

We used the Wilcoxon rank sum and Kruskal-Wallis tests for comparisons between independent groups and we used the Wilcoxon sign rank test to compare paired data. Geometric mean ratios (GMRs) with 95% CIs were produced when comparing groups. When appropriate, adjusted GMTs and GMRs were also presented to adjust for the effect of age. We did not do a sample size calculation for the immunogenicity subgroups in this analysis because of logistical considerations, including laboratory capacity. Sample size calculations for COV001 and COV002 were based on the primary efficacy outcome, which have been previously reported.

The reactogenicity cohorts included masked participants who received at least two standard doses of ChAdOx1 nCoV-19 in the two-dose cohort or three standard doses of ChAdOx1 nCoV-19 in the three-dose cohort, and had completed at least one entry in their adverse event diary after each dose. For consistency between cohorts, participants were excluded from the two-dose reactogenicity cohort if the interval between first and second dose was outside of the intervals defined in the two-dose immunogenicity cohort. All analyses of COV002 participants were restricted to those aged 18–55 years, to align with the inclusion criteria of the COV001 study, which only enrolled participants in this age range.

Our analysis of the decay of antibodies and T-cell responses over time after a single dose included all available data from timepoints up until the booster dose was administered (ie, day 28 and the day of the second dose, which varied across all participants). We modelled data using an unadjusted restricted-maximum likelihood-based mixed-effects regression approach (SAS proc mixed) with participant-level random intercepts fitted to log-transformed antibody values. We used the variance components covariance structure. We estimated GMRs and GMTs from the linear combination of model parameters. We chose the linear models after comparison with quadratic models and generalised additive (smoothed) models (GAM). The quadratic term was non-significant in the linear models and the GAM results were similar and did not substantially improve the model fits (compared using Akaike information criterion [known as AIC] statistics) from the linear models; therefore, the linear models were retained.

We did all statistical analyses using R (version 4.0.2 or later) and SAS (version 9.2). p values of less than 0·05 were considered to be significant and we made no adjustments for multiple comparisons. COV001 is registered with ClinicalTrials.gov, NCT04324606, and ISRCTN, 15281137, and COV002 is registered with ClinicalTrials.gov, NCT04400838, and ISRCTN, 15281137, and both are no longer recruiting.

### Role of the funding source

AstraZeneca reviewed the manuscript before submission, but the academic authors retained editorial control. All other funders of the study had no role in the study design, data collection, data analysis, data interpretation, or writing of the report.

## Results

**Figure 1:**
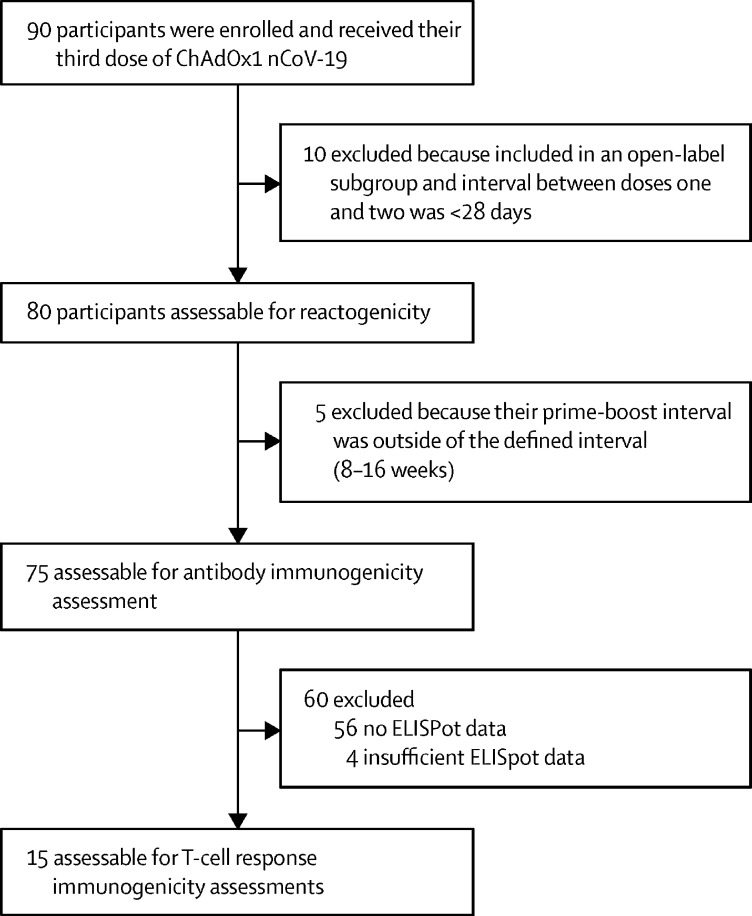
Trial profile for three-dose cohort

Between March 11 and 21, 2021, 90 participants were enrolled into the COV001 third-dose boost substudy and vaccinated with ChAdOx1 nCoV-19. Ten participants from this substudy were excluded from analyses because they were enrolled from an open-label subgroup and the interval between their first and second doses was shorter than 28 days. The remaining 80 participants who were assessable for reactogenicity received their third dose of vaccine 20–38 weeks after their second dose (median 30 weeks [IQR 30–30]). A further five participants were excluded from all immunogenicity assessments because their prime-boost interval was outside the defined range of 8–16 weeks. For immunogenicity analysis of T-cell responses, another 60 participants were excluded because they did not have or had insufficient ELISpot data, leaving an analysable cohort of 15 participants (figure 1). To maintain blinding at the time of vaccination, 40 control participants (who had previously received two doses of MenACWY) were also recruited and vaccinated with ChAdOx1 nCoV-19. Data for these participants have not been included in these analyses.

Of 1110 participants from the COV001 and COV002 studies who had received a single dose of vaccine, 480 were included in the single-dose immunogenicity assessment of antibodies and 44 were included in the immunogenicity assessment of T-cell responses (appendix 3 p 2). 66 participants from the single-dose cohort of COV001 were also offered a second dose, of whom 44 were ChAdOx1 nCoV-19 recipients and 22 were control group participants who had received MenACWY. 32 (73%) of 44 participants who had previously received a single dose of ChAdOx1 nCoV-19 received their second dose, with an interval of 44–45 weeks. Two of these participants were subsequently excluded from reactogenicity and immunogenicity analyses because they had positive PCR tests for SARS-CoV-2 infection within the follow-up period, leaving 30 participants for inclusion in the two-dose cohort analyses.

The two-dose cohort comprised 321 participants from COV001 and COV002 with prime-boost intervals of 8–12 weeks (267 [83%] of 321), 15–25 weeks (24 [7%]), or 44–45 weeks (30 [9%]) who had reactogenicity data available and were included in our analyses, and 261 who had immunogenicity data available (115 [44%] of 261 had an 8–12 week interval, 116 [44%] had a 15–25 week interval, and 30 [11%] had a 44–45 week interval; appendix 3 p 3).

Baseline characteristics for the one-dose, two-dose, and three-dose cohorts are shown in appendix 3 (pp 4–5). More than 90% of participants were White. There were small differences in the median age of reactogenicity cohorts and immunogenicity cohorts. The median age of participants in the two-dose cohort antibody immunogenicity cohort with an 8–12 week interval between the first and second dose was 39 years (IQR 30–49), in the 15–25 weeks interval group was 36 years (30–43), and in the 44–45 weeks interval group was 32 years (25–44). In the three-dose cohort, the median age of participants in the reactogenicity cohort was 37 years (IQR 31–42), in the immunogenicity antibody cohort was 37 years (31–42), and in the immunogenicity T-cell response cohort 40 years (32–44).

**Figure 2:**
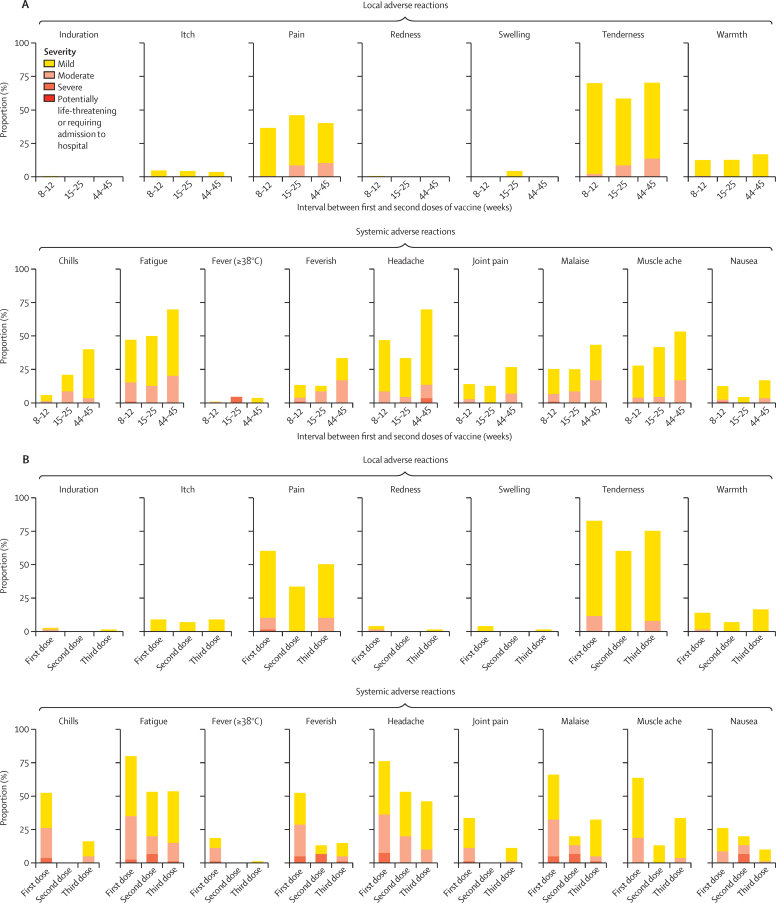
Solicited adverse reactions up to 7 days after ChAdOx1 nCoV-19 vaccination by interval between first and second doses (A) and after the first, second, and third dose for participants who received a third dose of vaccine (B) Figure shows maximum severity of respective solicited adverse event recorded for each participant during days 0–7 after vaccination. In panel A, reactogenicity data after the second dose are shown for 263 participants for fever (≥38°C) and 267 participants for all other symptoms for the 8–12 week interval, for 23 participants for fever (≥38°C) and 24 participants for all other symptoms for the 15–25 week interval, and 28 participants for fever (≥38°C) and 30 participants for all other symptoms for the 44–45 week interval. In panel B, reactogenicity data are from after each dose recorded by participants who received a third dose of vaccine, with data available for 80 participants for all symptoms after dose 1; 15 participants for all symptoms after dose 2; and 77 participants for fever and 80 participants for all symptoms after dose 3. Participants included in panel B received their third dose 20–38 weeks after the second dose (median of 30 weeks [IQR 30–30]).

The severity of local and systemic solicited adverse reactions 7 days after a second dose were mostly mild to moderate irrespective of the interval between doses. Local symptoms occurred after a second dose in 201 (75%) of 267 participants in the 8–12 week interval group, 15 (63%) of 24 participants in the 15–25 week interval group, and 23 (77%) of 30 participants in the 44–45 wee interval group (figure 2A; appendix 3 pp 6–9). Systemic reactions occurred in 190 (71%) of 267 participants in the 8–12 week interval group, 18 (75%) of 24 participants in the 15–25 week interval group, and 26 (87%) of 30 participants in the 44–45 week interval group (figure 2A; appendix 3 pp 6–9). 65 (81%) of 80 participants in the three-dose group reported at least one local symptom after a third dose (figure 2B; appendix 3 pp 10–14).

Second dose vaccinations in the two-dose cohort were less reactogenic than first dose vaccinations; with 72 (22%) of 321 participants reporting more than two moderate-to-severe systemic symptoms after first vaccination compared with 21 (7%) of 321 participants after second vaccinations (appendix 3 pp 6–9). Third dose vaccinations were also less reactogenic than first doses, with four (5%) of 80 participants in the three-dose cohort reporting more than two moderate-to-severe systemic symptoms after a third dose compared with 27 (34%) of 80 participants after the first dose (appendix 3 pp 10–14).

**Figure 3:**
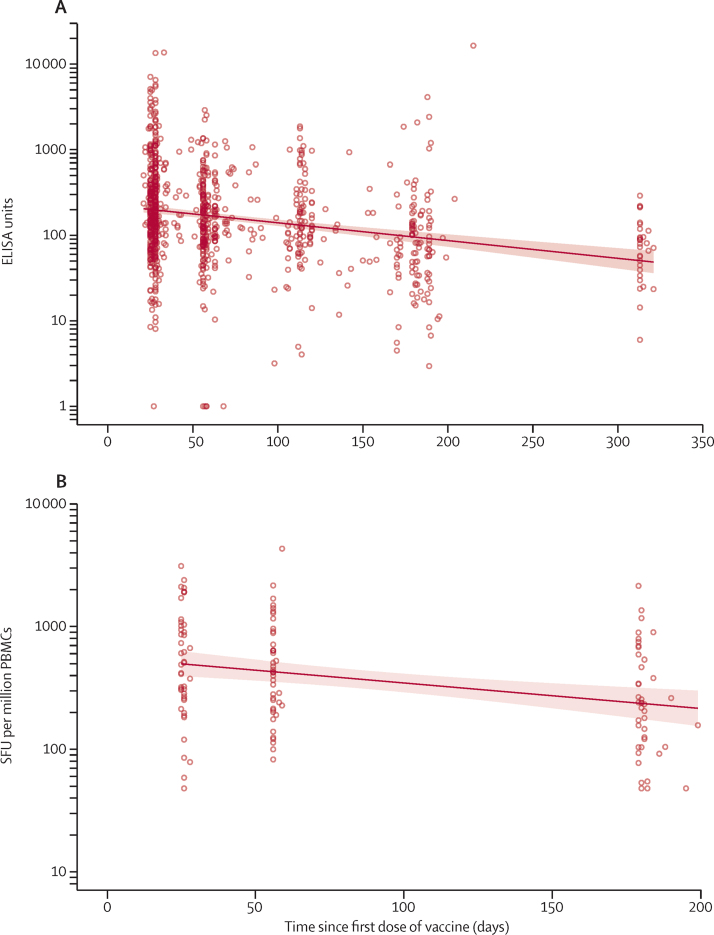
Antibody (A) and T-cell (B) persistence after one dose of ChAdOx1 nCoV-19 vaccine Datapoints represent individual participants and the solid line represents estimates from a linear regression model, with shaded areas showing the 95% CI. Antibody levels to SARS-CoV-2 Victoria/01/2020 spike measured by total IgG ELISA over 1 year after a single dose. Data are from 480 participants across COV001 and COV002 who received a standard dose of ChAdOx1 nCoV-19. Vaccine-induced T-cell responses against the SARS-CoV-2 spike insert were monitored up to day 182 in a cohort of 44 participants who received a single dose of ChAdOx1 nCoV-19. For participants who were excluded from these analyses due to positive PCR test result, second dose on trial, or external COVID-19 vaccination, no ELISA results or ELISpot results beyond the date of censoring were used. PBMCs=peripheral blood mononuclear cells. SFUs=spot-forming units

Antibody responses after a single dose of vaccine and measured approximately 320 days after vaccination remained higher than responses measured at baseline (GMTs of 66·00 ELISA units [EUs; 95% CI 47·83–91·08 *vs* 1·75 EUs [1·60–1·93]). At day 180, geometric mean antibody levels were half the levels observed at the day 28 peak (GMR 0·51 [95% CI 0·45–0·57]), and by day 320 were less than a third of the levels at the peak (GMR 0·27 [0·22–0·34; figure 3A; appendix 3 p 15).

Vaccine-induced cellular immune responses after a single dose of ChAdOx1 nCoV-19 followed a similar pattern of decay as antibody responses. T-cell responses decreased over the course of 6 months but were maintained above baseline levels, and at day 180 geometric mean T-cell levels were half the levels observed at the day 28 peak (GMR 0·50 [95% CI 0·41–0·60]; figure 3B; appendix 3 p 15).

**Figure 4:**
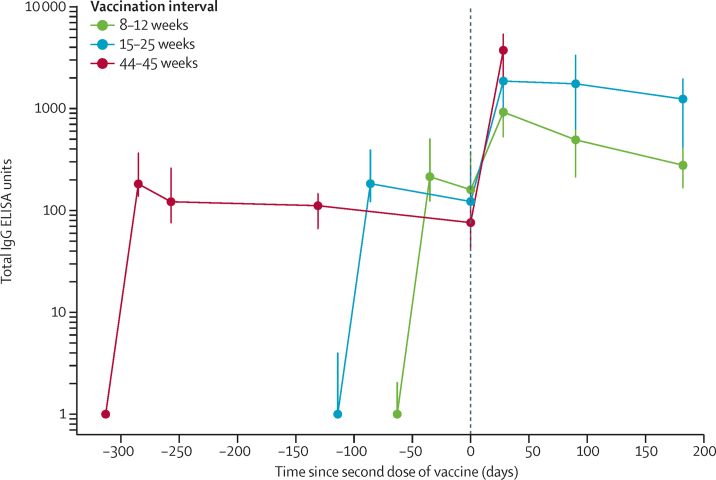
Antibody response by interval between first and second vaccination Datapoints are medians for each group, with error bars showing IQRs. Antibody levels to SARS-CoV-2 Victoria/01/2020 spike measured by total IgG ELISA. Data are shown for 115 participants for the 8–12 week interval; 116 participants for the 15–25 week interval, and 30 participants for the 44–45 week interval. Unadjusted and age-adjusted geometric mean ratios are shown in appendix 3 (p 16).

Antibody levels 28 days after a second dose of vaccine were higher among those with longer intervals between doses than among those with shorter intervals between doses (median total IgG titre of 923 EUs [IQR 525–1764] for 8–12 week interval; 1860 EUs [917–4934] with 15–25 week interval; and 3738 EUs [1824–6625] with 44–45 week interval; Kruskal-Wallis test p<0·0001; figure 4; appendix 3 p 16). Age was not statistically significant in adjusted models (appendix 3 p 16).

6 months after the second dose of vaccine, antibody levels remained significantly higher in the group with a 15–25 week interval between doses compared with an 8–12 week interval (median 1240 EUs [IQR 432–2002] *vs* 278 EUs [166–499]; Wilcoxon rank sum test with continuity correction p<0·0001; figure 4; appendix 3 p 16).

IgG binding titres to all four variants tested (D614G, alpha, beta, and gamma) were significantly greater after second dose than after the first dose (p<0·0001 for all comparisons [pairwise comparisons using Wilcoxon sign rank test]; appendix 3 pp 17, 19).

**Figure 5:**
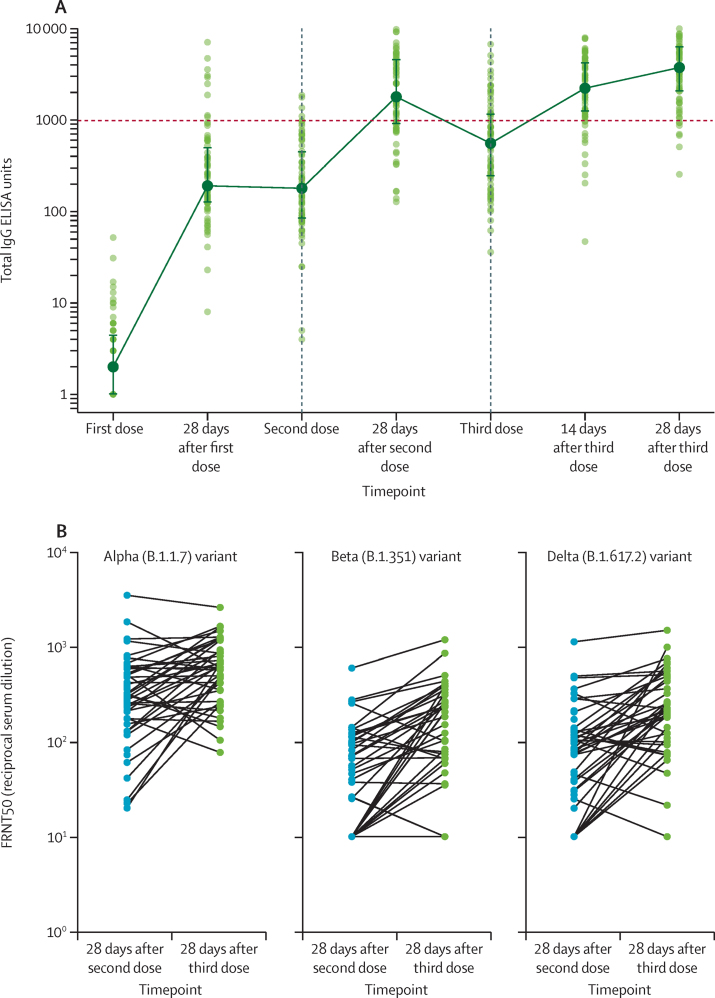
Antibody responses in participants who received a third dose of ChAdOx1 nCoV-19 (A) Antibody levels to SARS-CoV-2 Victoria/01/2020 spike protein measured by total IgG ELISA (n=75). Datapoints in lighter colours represent individual participants and darker datapoints show median values with error bars showing the IQRs and with solid lines connecting these median values. (B) Neutralisation titres from a randomly selected subset of participants (45 of 75 participants who received a third dose of vaccine and who had an interval of 8–16 weeks between their first and second dose). Datapoints represent individual participants for the three variants of concern investigated. FRNT50=focus reduction neutralisation titres with 50% neutralisation cutoff.

Antibody responses after a third dose of vaccine were assessed in 75 participants who had received their first two doses with an interval of 8–16 weeks, and who subsequently received their third dose 28–38 weeks after the second (median 30 weeks [IQR 30–30]). Administering a third dose of vaccine boosted antibody response to Victoria/01/2020 SARS-CoV-2 spike protein (figure 5A; appendix 3 p 18). Antibody levels after the third dose were significantly higher than after the second dose (median total IgG titre was 1792 EUs [IQR 899–4634] at 28 days after the second dose *vs* 3746 EUs [2047–6420] 28 days after the third dose; pairwise comparison in 73 participants due to two samples not being available at these timepoints using Wilcoxon signed rank test p=0·0043). Binding antibody titres to the beta variant increased after a third dose (appendix 3 p 19). Neutralising antibody titres after a third dose were higher than those after the second dose against alpha (p=0·0023), beta (p<0·0001), and delta (p<0·0001) variants (Wilcoxon signed rank test; figure 5B; appendix 3 p 20).

**Figure 6:**
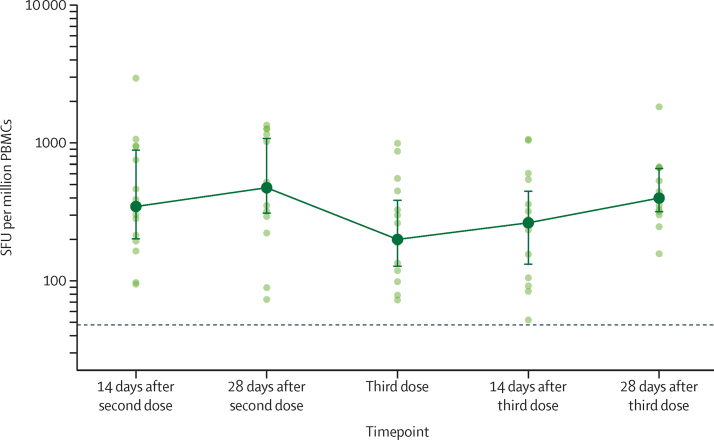
IFN-γ ELISpot responses in participants who received a third dose of ChAdOx1 nCoV-19 15 participants with an interval of 8 weeks between their first and second doses were assessed for ELISpot responses. These participants received their third dose 37–38 weeks after the second dose (median 38 weeks [IQR 38–38]). Datapoints in lighter colours represent individual participants and darker datapoints show median values with error bars showing the IQRs and with solid lines connecting these median values. The dotted horizontal line represents the lower limit of detection of the assay (48 SFU per million PBMCs). SFU=spot-forming unit. PBMC=peripheral blood mononuclear cells.

Spike-specific cellular immune responses were measured after a third dose of ChAdOx1 nCoV-19 in 15 individuals. These individuals had received their first two doses with an interval of 8 weeks, and subsequently received their third dose 37–38 weeks after the second (median 38 weeks [IQR 38–38]). Median response increased from 200 spot-forming units (SFUs) per million PBMCs (IQR 127–389) immediately before the third dose to 264 SFUs per million PBMCs (131–452) 14 days after the third dose (p=0·57), and to 399 SFUs per million PBMCs (314–662) by 28 days after the third dose (p=0·012; figure 6; appendix 3 p 20). Peak responses at day 28 after the third dose were not significantly different to the responses after the second dose (p=0·060; appendix 3 p 20).

## Discussion

Antibody levels induced by a single dose of ChAdOx1 nCoV-19 decreased gradually but remained above baseline levels after 1 year. We have previously shown that administration of a second dose of vaccine induces higher antibody responses by 1 month after the second dose than before the second dose, with higher responses with a dose interval up to 3 months between the first two doses.^3^ Here, we found that a long extension of the dose interval (up to 45 weeks) between the first and second dose further enhances the immune response to the second dose when compared with shorter dose intervals. Furthermore, for the first time, we showed that a third dose of ChAdOx1 nCoV-19 can induce a strong boost to immune responses to the transgene product, SARS-CoV-2 spike protein, and that these responses result in increased neutralising antibody titres and enhanced antibody activity against variants.

The devastating impact of COVID-19 is most apparent in countries with low vaccine coverage and little health-care infrastructure, including low-income and middle-income countries. Global vaccine shortages and policy decisions implemented at national levels have curtailed vaccine supplies for some countries where substantial numbers of individuals have already received one dose of vaccine. We have previously shown that protection against symptomatic COVID-19 is maintained after a single dose of ChAdOx1 nCoV-19 for at least 3 months, despite some waning of antibody levels^3^ and we now report that the antibody levels remain above baseline for at least 1 year after single dose immunisation. These data are important for those countries where administration of a second dose is delayed because of a shortage of supply. We also showed here that an extended interval between the first and second dose of ChAdOx1 nCoV-19 results in a significantly higher antibody response 28 days after the second dose than with shorter intervals. This finding is consistent with previous data showing a longer interval between first and second dose of ChAdOx1 nCoV-19 resulted in an increase in antibody titres,^3^ thus providing further reassurance that a delay in administration of the second dose will not compromise the level of protection attained. Similar findings have been reported with other vaccines;^1^, ^7^, ^8^ a delayed two-dose regimen against HPV, given at least 6 months apart, results in as good or better antibody response than does three doses. A second dose of ChAdOx1 nCoV-19 is well tolerated in a delayed two-dose schedule and a third dose is also well tolerated. Reports have emerged of thrombosis and thrombocytopenia after the first dose of ChAdOx1 nCoV-19,^9^ and information from Public Health England indicates that this very rare event might not occur after a second dose.^10^

If booster vaccinations against SARS-CoV-2 will be required, perhaps to counter waning immunity or to augment protection against emerging variants, is not yet known. Here, we show that a third dose of ChAdOx1 nCoV-19 is well tolerated and significantly boosts antibody titres above those measured after the second dose to the level associated with 80% vaccine efficacy, or higher, after two vaccine doses (unpublished; preprint data available^11^). Higher titre neutralising antibodies against alpha, beta, and delta variants of SARS-CoV-2 were induced 28 days after a third dose vaccination than after the second dose. Spike-specific T-cell responses were boosted after a third dose of ChAdOx1 nCoV-19 and were similar in magnitude to the responses measured after two doses. Although pre-existing immunity to human adenoviral vectors has been shown to dampen vaccine-induced immune responses,^12^, ^13^ here we found no evidence that repeated use of a replication-deficient simian adenoviral vector induces antivector immunity at a level sufficient to impair responses to further vaccination. The third dose was well tolerated by participants with lower reactogenicity than after the first dose.

Our study has several limitations, including a paucity of T-cell data after a late second dose, a paucity of tolerability data after the second dose for those who were recruited to receive a third dose, and the small number of participants who were available at 1 year after single dose who still had only received one dose (mainly due to being offered a second dose after unblinding, as per protocol). Thus far, data are only available 28 days after the third dose; however, follow-up at 6 months is planned. Participants were aged 18–55 years and caution should be taken when extrapolating our findings to beyond this age range. The sample size in this study is not sufficiently large to assess rare vaccine side-effects, but there were no tolerability concerns reported in those receiving a late second dose or a third dose booster. These results are from a mainly White population and cannot necessarily be generalised to other populations.

Here, we found that immunity induced by the viral vectored vaccine ChAdOx1 nCoV-19 is maintained for long periods after a first dose, with greater boosting of effects after the second dose after a longer interval between doses than shorter intervals. Therefore, a single dose of ChAdOx1 nCoV-19 with a second dose given after an extended period might be an effective strategy^3^, ^14^ in settings where vaccine supplies are scarce in the short term. A third dose resulted in a further increase in immune responses, including increased neutralisation of variant SARS-CoV-2 viruses, and could be used to increase vaccine efficacy against variants in susceptible populations.

## Data sharing

The current study protocol for COV001 is provided in appendix 1 and for COV002 in appendix 2. Anonymised participant data will be made available when the trial is complete, upon requests directed to the corresponding author. Proposals will be reviewed and approved by the sponsor, investigator, and collaborators on the basis of scientific merit. After approval of a proposal, data can be shared through a secure online platform after signing a data access agreement. All data will be made available for a minimum of 5 years from the end of the trial.

## Declaration of interests

SCG and AVSH are cofounders of and shareholders in Vaccitech (collaborators in the early development of ChAdOx1 nCoV-19) and named as inventors on a patent covering use of ChAdOx1-vectored vaccines (PCT/GB2012/000467) and a patent application covering this SARS-CoV-2 vaccine (SCG only). TL is named as an inventor on a patent covering use of ChAdOx1-vectored vaccines (PCT/GB2012/000467) and was a consultant to Vaccitech. PMF is a consultant to Vaccitech. AJP is chair of the UK Department of Health and Social Care's Joint Committee on Vaccination and Immunisation, but does not participate in policy advice on coronavirus vaccines, and is a member of the WHO Strategic Advisory Group of Experts (SAGE). AJP is an NIHR Senior Investigator. All other authors declare no competing interests.
