# Supplementary material for: Reactogenicity and immunogenicity after a late second dose or a third dose of ChAdOx1 nCoV-19 in the UK: a substudy of two randomised controlled trials (COV001 and COV002)
Source: Lancet. 2021 Sep 11;398(10304):981–90. doi: 10.1016/S0140-6736(21)01699-8 (PMC8409975; doi:10.1016/S0140-6736(21)01699-8)
Supplement: Supplementary appendix 1 [file mmc1.pdf]

THE LANCET

Supplementary appendix

This appendix formed part of the original submission and has been peer reviewed. We post it as supplied by the authors.

Supplement to: Flaxman A, Marchevsky NG, Jenkin D, et al. Reactogenicity and immunogenicity after a late second dose or a third dose of ChAdOx1 nCoV-19 in the UK: a substudy of two randomised controlled trials (COV001 and COV002). *Lancet* 2021; published online Sept 1. http://dx.doi.org/10.1016/S0140-6736(21)01699-8.

Trial Title: A phase I/II study to determine efficacy, safety and immunogenicity of the candidate Coronavirus Disease (COVID-19) vaccine ChAdOx1 nCoV-19 in UK healthy adult volunteers

Short title A phase I/II study of a candidate COVID-19 vaccine (COV001)

Study Reference: COV001

Protocol Version: 18.0

Date: 12 April 2021

EudraCT number: 2020-001072-15

REC Reference: 20/SC/0145

IRAS Reference: 281259

Chief Investigator: Prof Andrew Pollard

Sponsor: University of Oxford

Funder: UK Research and Innovation

Key Trial Contacts

**Chief Investigator**	Prof Andrew Pollard
**Address**	Centre for Clinical Vaccinology and Tropical Medicine<br>University of Oxford,<br>Churchill Hospital, Old Road, Headington<br>Oxford, OX3 7LE<br>Email: andrew.pollard@paediatrics.ox.ac.uk
**Clinical Trials Unit Investigators**	Primary Care and Vaccines Collaborative CTU<br>Prof Brian Angus<br>Centre for Clinical Vaccinology and Tropical Medicine<br>Churchill Hospital, Old Road, Headington<br>Oxford, OX3 7LE<br>Email: brian.angus@ndm.ox.ac.uk<br><br>Prof Adrian Hill<br>The Jenner Institute, University of Oxford<br>Old Road Campus Research Building (ORCRB)<br>Roosevelt Drive Oxford OX3 7DQ<br>Email: adrian.hill@ndm.ox.ac.uk<br><br>Prof Sarah Gilbert<br>The Jenner Institute, University of Oxford<br>Old Road Campus Research Building (ORCRB)<br>Roosevelt Drive Oxford OX3 7DQ<br>Email: sarah.gilbert@ndm.ox.ac.uk<br><br>Dr Pedro Folegatti<br>Centre for Clinical Vaccinology and Tropical Medicine<br>Churchill Hospital, Old Road, Headington<br>Oxford, OX3 7LE<br>Email: pedro.folegatti@ndm.ox.ac.uk<br><br>Dr Alexander Douglas<br>The Jenner Institute<br>Wellcome Centre for Human Genetics<br>Roosevelt Drive<br>Oxford, OX3 7BN<br>Email: sandy.douglas@ndm.ox.ac.uk<br><br>Dr Maheshi Ramasamy<br>Oxford Vaccine Group<br>Centre for Clinical Vaccinology and Tropical Medicine<br>University of Oxford,<br>Churchill Hospital, Old Road, Headington<br>Oxford, OX3 7LE<br>Email : maheshi.ramasamy@paediatrics.ox.ac.uk

Prof Matthew Snape
Oxford Vaccine Group
Centre for Clinical Vaccinology and Tropical Medicine
University of Oxford,
Churchill Hospital, Old Road, Headington
Oxford, OX3 7LE
Email : matthew.snape@paediatrics.ox.ac.uk

Prof Teresa Lambe
The Jenner Institute, University of Oxford
Old Road Campus Research Building (ORCRB)
Roosevelt Drive Oxford OX3 7DQ
Email: teresa.lambe@ndm.ox.ac.uk

Prof Katie Ewer
The Jenner Institute, University of Oxford
Old Road Campus Research Building (ORCRB)
Roosevelt Drive Oxford OX3 7DQ
Email: katie.ewer@ndm.ox.ac.uk

Dr Angela Minassian
Centre for Clinical Vaccinology and Tropical Medicine
University of Oxford,
Churchill Hospital, Old Road, Headington
Oxford, OX3 7LE
Email : angela.minassian@ndm.ox.ac.uk

Trial Sites

Centre for Clinical Vaccinology & Tropical Medicine
University of Oxford
Churchill Hospital, Old Road, Headington, Oxford, OX3 7LE
PI at Site: Professor Adrian Hill
Email: adrian.hill@ndm.ox.ac.uk

Oxford University Hospital Foundation Trust
Prof Brian Angus
John Radcliffe Hospital
Headley Way, Headington
Oxford
OX3 9DU
Email: brian.angus@ndm.ox.ac.uk

NIHR WTCRF
University Hospital Southampton NHS Foundation Trust Southampton, SO16 6YD
PI at Site: Professor Saul Faust
Email: s.faust@soton.ac.uk

IHR Imperial CRF
NIHR Imperial Clinical Research Facility Imperial College
Hammersmith Hospital, Imperial College NHS Trust

	150 Du Cane Road, London, W12 0HS<br>PI at Site: Dr Katrina M. Pollock<br>Email: k.pollock@imperial.ac.uk
	**St Georges University Hospital NHS Foundation Trust**<br>Blackshaw Road, Tooting, London<br>SW17 0QT<br>PI at site: Professor Paul Heath<br>Email: pheath@sgul.ac.uk
	**St Georges University of London**<br>Paediatric Infectious Diseases Research Group<br>Jenner Wing, Level 2, Room 2.216F, Mail Point J2C<br>London<br>SW17 0RE<br>Email: pheath@sgul.ac.uk
	**University Hospitals Bristol and Weston NHS Foundation Trust**<br>Marlborough Street, Bristol, Avon<br>BS1 3NU<br>PI at site: Dr Rajeka Lazarus<br>Co Investigator: Professor Adam Finn<br>PI Email: rajeka.lazarus@uhbw.nhs.uk<br>Co- investigator email: Adam.Finn@bristol.ac.uk
**Sponsoring Institution**	**University of Oxford**<br>Clinical Trials and Research Governance<br>Joint Research Office,<br>Boundary Brook House<br>Churchill Drive, Headington<br>Oxford OX3 7GB<br>Email: ctrg@admin.ox.ac.uk
**Funder**	**UK Government**
**Monitor**	**Clinical Trials & Research Governance (Oxford Sites)**<br>Joint Research Office,<br>Boundary Brook House<br>Churchill Drive, Headington<br>Oxford OX3 7GB
	**Oxford Vaccine Centre (other sites)**<br>CCVTM<br>Churchill Hospital<br>Headington<br>OXON<br>OX3 7LE<br>Email: covid19@ndm.ox.ac.uk
**DSMB Chair**	Professor Christopher Chiu<br>Imperial College London

Professor Robert Heyderman (Co-chair)
University College London

Statistician

Merryn Voysey
Oxford Vaccine Centre
CCVTM, Churchill Hospital
Headington, OXON
OX3 7LE
Merryn.voysey@paediatrics.ox.ac.uk

Signature:

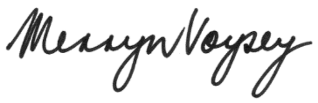

Confidentiality Statement

This document contains confidential information that must not be disclosed to anyone other than the Sponsor, the Investigator Team, HRA, host organisation, and members of the Research Ethics Committee and other regulatory bodies. This information cannot be used for any purpose other than the evaluation or conduct of the clinical investigation without the prior written consent of Prof Andrew Pollard.

Statement of Compliance
The trial will be conducted in compliance with the protocol, the principles of Good Clinical Practice, Medicines for Human Use (Clinical Trial) Regulations 2004 (as amended) and all other applicable regulatory requirements.

Investigator Agreement and Notification of Conflict of Interest I approve this protocol for use in the above named clinical trial and agree to abide by all provisions set forth therein.

According to the Declaration of Helsinki, 2008, I have read this protocol, and declare no/~~the following (delete as appropriate)~~ conflict of interest

Chief Investigator	Signature	Date: 13/04/2021
Prof Andrew Pollard		

Site: **Centre for Clinical Vaccinology and Tropical Medicine, University of Oxford**

I have read this protocol and agree to abide by all provisions set forth therein.

According to the Declaration of Helsinki, 2008, I have read this protocol, and declare the following conflict of interest. AH is a cofounder of and minor shareholder in an Oxford University spin-off company, Vaccitech Ltd, that has some non-exclusive rights to the vector, ChAdOx1, used in the vaccine to be tested, that may be of commercial value"

Principal Investigator	Signature	Date: 13/04/2021
Adrian Hill		

Site: **NIHR WTCRF**

I have read this protocol and agree to abide by all provisions set forth therein.

According to the Declaration of Helsinki, 2008, I have read this protocol, and declare no/~~the following (delete as appropriate)~~ conflict of interest

Principal Investigator Signature Date: 13/04/2021

Saul Faust

Site: **NIHR Imperial CRF**

I have read this protocol and agree to abide by all provisions set forth therein.

According to the Declaration of Helsinki, 2008, I have read this protocol, and declare no/~~the following (delete as appropriate)~~ conflict of interest

Principal Investigator Signature Date: 15/04/2021

Katrina M. Pollock

Site: **Oxford University Hospital Foundation Trust**

I have read this protocol and agree to abide by all provisions set forth therein.

According to the Declaration of Helsinki, 2008, I have read this protocol, and declare no/~~the following (delete as appropriate)~~ conflict of interest

Principal Investigator Signature Date: 13/04/2021

Brian Angus

Site: **St Georges University Hospital NHS Foundation Trust**

I have read this protocol and agree to abide by all provisions set forth therein.

According to the Declaration of Helsinki, 2008, I have read this protocol, and declare no/~~the following (delete as appropriate)~~ conflict of interest

Principal Investigator Signature Date: 14/04/2021

Paul Heath

Site: **St Georges University of London**

I have read this protocol and agree to abide by all provisions set forth therein.

According to the Declaration of Helsinki, 2008, I have read this protocol, and declare no/~~the following (delete as appropriate)~~ conflict of interest

Principal Investigator Signature Date: 14/04/2021

Paul Heath

Site: **University Hospitals Bristol and Weston NHS Foundation Trust**

I have read this protocol and agree to abide by all provisions set forth therein.

According to the Declaration of Helsinki, 2008, I have read this protocol, and declare no/~~the following (delete as appropriate)~~ conflict of interest

Principal Investigator Signature Date: 14/04/2021

Rajeka Lazarus

Table of Contents

1 SYNOPSIS 14
2 ABBREVIATIONS 21
3 BACKGROUND AND RATIONALE 23
3.1 Background 23
3.2 Pre-Clinical Studies 24
3.2.1 Immunogenicity (Jenner Institute, unpublished) 24
3.2.2 Efficacy 25
3.3 Disease Enhancement 25
3.4 Previous clinical experience 26
3.5 Rationale 31
4 OBJECTIVES AND ENDPOINTS 34
5 TRIAL DESIGN 37
5.1 Study groups 38
5.2 Trial volunteers 40
5.3 Definition of End of Trial 40
5.4 Duration of study 40
5.5 Potential Risks for volunteers 40
5.6 Known Potential Benefits 42
6 RECRUITMENT AND WITHDRAWAL OF TRIAL VOLUNTEERS 43
6.1 Identification of Trial Volunteers 43
6.2 Informed consent 44
6.3 Inclusion and exclusion criteria 45
6.3.1 Inclusion Criteria 45
6.3.2 Exclusion Criteria 45
6.3.3 Re-vaccination exclusion criteria 47
6.3.4 Effective contraception for female volunteers 48

6.3.4.1 Contraception for female volunteers following unblinding and provision of ChAdOx1 nCoV-19 vaccines (Protocol V17.0) 48
6.3.5 Prevention of 'Over Volunteering' 49
6.3.6 Withdrawal of Volunteers 49
6.3.6.1 Discontinuation from Study Procedures 50
6.4 Pregnancy 50
7 TRIAL PROCEDURES 51
7.1 Schedule of Attendance 51
7.2 Observations 51
7.3 Blood tests, Nose/Throat Swab and urinalysis 51
7.4 Study visits 53
7.4.1 Screening visit 53
7.4.2 Day 0: Enrolment and vaccination visit 54
7.4.2.1 Vaccination 54
7.4.2.2 Sequence of Enrolment and Vaccination of Volunteers 55
7.4.3 Subsequent visits: 55
7.4.4 Symptomatic volunteers 75
7.4.4.1 Diagnostic SARS-CoV-2 NAAT testing outside of the trial 75
7.4.4.2 COVID-19 testing visit 75
7.4.4.3 COVID-19 Testing plus 7 day phone call 76
7.4.4.4 Positive SARS-CoV-2 NAAT test and vaccination 76
7.4.5 Household Weekly Questionnaire (optional) 76
7.4.6 Stool samples (optional) 76
7.4.7 Medical notes review 77
7.4.8 Randomisation, blinding and code-breaking 77
7.4.8.1 Unblinding of all trial participants (protocol V17.0) 78
7.4.9 Unblinded participants who are eligible to receive an approved or licensed SARS-CoV-2 vaccine 78
7.4.10 Late Vaccination sub-groups 79
8 INVESTIGATIONAL PRODUCT AND TRIAL INTERVENTIONS 81
8.1 Manufacturing and presentation 81

8.1.1 Description of ChAdOx1 nCoV-19 81
8.2 Supply 81
8.3 Storage 81
8.4 Administration 81
8.5 Rationale for selected dose 82
8.6 Minimising environmental contamination with genetically modified organisms (GMO) 85
8.7 Control Vaccine 85
8.8 Compliance with Trial Treatment 86
8.9 Accountability of the Trial Treatment 86
8.10 Paracetamol (non-IMP) 86
8.11 Concomitant Medication 86
8.12 Provision of Treatment for Controls 86
9 ASSESSMENT OF SAFETY 88
9.1 Definitions 88
9.1.1 Adverse Event (AE) 88
9.1.2 Adverse Reaction (AR) 88
9.1.3 Serious Adverse Event (SAE) 88
9.1.4 Serious Adverse Reaction (SAR) 89
9.1.5 Suspected Unexpected Serious Adverse Reaction (SUSAR) 89
9.2 Expectedness 89
9.3 Foreseeable Adverse Reactions: 89
9.4 Adverse Events of Special Interest (AESI) 89
9.5 Causality 90
9.6 Reporting Procedures for All Adverse Events 91
9.7 SAE and AESI collection period 91
9.8 Assessment of severity 92
9.9 Reporting Procedures for Serious AEs 94

9.10 Reporting Procedures for SUSARS 95
9.11 Development Safety Update Report 95
9.12 Procedures to be followed in the event of abnormal findings 95
9.13 Interim Reviews 95
9.14 Data Safety Monitoring Board 96
9.15 Safety Group Holding Rules 96
9.15.1 Group holding rules 97
9.15.2 Individual stopping rules (will apply to prime-boost group only) 98
10 STATISTICS 100
10.1 Description of Statistical Methods 100
10.2 Primary efficacy 100
10.3 Safety & Reactogenicity 101
10.4 Immunogenicity 101
10.5 Subgroup analyses 102
10.6 Interim and primary analyses of the primary outcome 102
10.7 Final Analysis 103
10.8 Procedure for Accounting for Missing, Unused, and Spurious Data. 103
10.9 Inclusion in Analysis 103
10.10 Interim analysis for the combined DSMB 103
11 DATA MANAGEMENT 104
11.1 Data Handling 104
11.2 Record Keeping 104
11.3 Source Data and Case Report Forms (CRFs) 105
11.4 Data Protection 105
11.5 Data Quality 106
11.6 Archiving 106
12 QUALITY CONTROL AND QUALITY ASSURANCE PROCEDURES 107

12.1 Investigator procedures ... 107

12.2 Monitoring ... 107

12.3 Protocol deviation ... 107

12.4 Audit & inspection ... 107

13 SERIOUS BREACHES ... 108

14 ETHICS AND REGULATORY CONSIDERATIONS ... 109

14.1 Declaration of Helsinki ... 109

14.2 Guidelines for Good Clinical Practice ... 109

14.3 Ethical and Regulatory Approvals ... 109

14.4 Volunteer Confidentiality ... 109

15 FINANCING AND INSURANCE ... 111

15.1 Financing ... 111

15.2 Insurance ... 111

15.3 Compensation ... 111

16 Publication Policy ... 112

17 DEVELOPMENT OF A NEW PRODUCT/PROCESS OR THE GENERATION OF INTELLECTUAL PROPERTY ... 113

1 SYNOPSIS

**Title**	A phase I/II study to determine efficacy, safety and immunogenicity of the candidate Coronavirus Disease (COVID-19) vaccine ChAdOx1 nCoV-19 in UK healthy adult volunteers.
**Trial Identifier**	COV001
**Trial Registration**	EudraCT number: 2020-001072-15<br>REC Reference: 20/SC/0145<br>IRAS Reference: 281259
**Chief Investigator**	Professor Andrew Pollard
**Clinical Phase**	I/II
**Design**	Single-blinded, randomised, controlled, multi-centre
**Population**	Healthy adults aged 18-55 years
**Planned Sample Size**	Up to 1090

Group	W0	W4	W8 (-7/+14 days)	Booster	Late Vacc (LV)	Late Vacc Two (LVT)
1a (n=44) Intense Follow-up	ChAdOx1 nCoV-19 $5x10^{10}$vp	-	-	-	-	-
1b (n=44) Intense Follow-up	MenACWY	-	-	-	-	-

1c $ (n=up to 44 from group 1a)	ChAdOx1 nCoV-19 5x10^{10}vp	-	-	-	ChAdOx1 nCoV-19 0.5mL (3.5-6.5x10^{10}vp)	-
1d $ (n=up to 22 from group 1b)	MenACWY	-	-	-	ChAdOx1 nCoV-19 0.5mL (3.5-6.5x10^{10}vp)	ChAdOx1 nCoV-19 0.5mL (3.5-6.5x10^{10}vp)
2a* (n= up to 206)	ChAdOx1 nCoV-19 5x10^{10}vp	-	-	-	-	-
2b* (n= up to 206)	MenACWY	-	-	-	-	-
2c* Prime-boost (up to 20 volunteers from 2a)	ChAdOx1 nCoV-19 5x10^{10}vp	-	ChAdOx1 nCoV-19 5x10^{10}vp	-	-	-
2d* Prime-boost (up to 32 volunteers from 2a)	ChAdOx1 nCoV-19 5x10^{10}vp	-	ChAdOx1 nCoV-19 2.5x10^{10}vp	-	-	-
2e* Prime-boost (up to 10 volunteers from 2b)	MenACWY	-	MenACWY	-	-	-
2f* Prime-boost (remaining participants from 2a, n=up to 154)	ChAdOx1 nCoV-19 5x10^{10}vp	-	-	ChAdOx1 nCoV-19 0.5mL (3.5-6.5x10^{10}vp)	-	-
2g* Prime-boost	MenACWY	-	-	MenACWY	-	-

(remaining participants from 2b, n= up to 196)						
3a (n=10)<br>Prime-boost	ChAdOx1 nCoV-19<br>5x10^{10}vp	ChAdOx1 nCoV-19<br>5x10^{10}vp	-	-		-
3b (up to n=10 from 3a)<br>Prime-boost	ChAdOx1 nCoV-19<br>5x10^{10}vp	ChAdOx1 nCoV-19<br>5x10^{10}vp	-	-	ChAdOx1 nCoV-19<br>0.5mL (3.5-6.5x10^{10}vp)	
4a** (n= up to 290)	ChAdOx1 nCoV-19<br>5x10^{10} vp	-	-	-	-	-
4b** (n= up to 290)	MenACWY	-	-	-	-	-
4c Prime-boost (n= up to 290)	ChAdOx1 nCoV-19<br>5x10^{10} vp	-	-	ChAdOx1 nCoV-19<br>0.5mL (3.5-6.5x10^{10}vp)	-	-
4d Prime-boost (n= up to 290)	MenACWY	-	-	MenACWY	-	-
5a† (n= up to 80 groups 2c, 2f and 4c, with a ≤16 week dose interval)	2 x ChAdOx1 nCoV-19<br>≤ 16 week dose interval				ChAdOx1 nCoV -19<br>0.5mL (3.5-6.5x10^{10}vp)	-
5b† (n= up to 40 from groups 2e, 2g and 4d, with a ≤16	2 x MenACWY<br>≤ 16 week dose interval				ChAdOx1 nCoV-19<br>0.5mL (3.5-6.5x10^{10}vp)	ChAdOx1 nCoV-19<br>0.5mL (3.5-6.5x10^{10}vp)

week dose interval)			

$Group 1c will consist of up to 44 participants who were previously allocated to group 1a and group 1d will consist of up to 22 participants who were previously allocated to group 1b. The allocation procedure for these sub-groups is described in section 7.4.10.

* Group 2 will consist of an overall sample size of up to 412 volunteers, of which up to 62 (52 IMP and 10 controls) will receive a booster dose at 8 weeks (-7/+14 days). The remaining participants in Group 2 will be invited to receive a booster dose at the earliest opportunity (minimum 4 weeks).

**Group 4 will consist of an overall sample size of up to 580 volunteers, of which up to 112 will be given Paracetamol at D0 visit. All volunteers in Group 4 will be invited to receive a booster dose at the earliest opportunity (minimum 4 weeks).

†Group 5a will consist of up to 80 participants who were previously allocated to Group 2c, 2f and 4c and Group 5b will consist of up to 40 participants who were previously allocated to Group 2e, 2g and 4d. All group 5 participants will have had a first-to-second dose interval of ≤16 week. The allocation procedure for these sub-groups is described in section 7.4.10.

Visit Schedule: See schedule of attendances for different groups

**Planned Trial Duration**	12 months from last vaccination visit (approximately 15 months from enrolment for participants receiving 2 doses)	
	**Objective**	**Outcome Measure**
**Primary**	To assess efficacy of the candidate ChAdOx1 nCoV-19 against COVID-19	a) Virologically confirmed (PCR* positive) symptomatic cases of COVID-19
**Co-Primary**	To assess the safety of the candidate vaccine ChAdOx1 nCoV	a) occurrence of serious adverse events (SAEs) throughout the study until a cut-off date of 1st July 2021 or 6 months post late vaccination visit, whichever is latest

**Secondary**	To assess the safety, tolerability and reactogenicity profile of the candidate vaccine ChAdOx1 nCoV	a) occurrence of solicited local reactogenicity signs and symptoms for 7 days following vaccination;<br>b) occurrence of solicited systemic reactogenicity signs and symptoms for 7 days following vaccination;<br>c) occurrence of unsolicited adverse events (AEs) for 28 days following vaccination; (7 days following vaccination for groups 1c,1d,4e and 4f)<br>d) change from baseline for safety laboratory measures and;<br>e) Occurrence of disease enhancement episodes
	To assess efficacy of the candidate ChAdOx1 nCoV-19 against severe and non-severe COVID-19	a) Hospital admissions associated with COVID-19<br>b) Intensive care unit (ICU) admissions associated with COVID-19<br>c) Deaths associated with COVID-19<br>d) Severe COVID-19 disease (defined according to clinical severity scales).<br>e) Seroconversion against non-Spike SARS-CoV-2 antigens
	To assess cellular and humoral immunogenicity of ChAdOx1 nCoV-19	a) Interferon-gamma (IFN-γ) enzyme-linked immunospot (ELISpot) responses to SARS-CoV-2 spike protein;<br>b) Quantify antibodies against SARS-CoV-2 spike protein (seroconversion rates)
**Exploratory**	Exploratory Immunology	a) virus neutralising antibody (NAb) assays against live and/or pseudotype SARS-CoV-2 virus<br>b)Cell analysis by flow cytometry assays

		c) Functional antibody assays
	To assess safety, reactogenicity, immunogenicity and efficacy endpoints, for participants receiving prophylactic paracetamol	All safety, reactogenicity, immunogenicity and efficacy endpoints.
	To assess immunogenicity of ChAdOx1 nCoV-19 given as homologous prime-boost	Quantify antibodies against SARS-CoV-2 spike protein (seroconversion rates) post boost
	To assess immunogenicity of a delayed prime-boost ChAdOx1 nCoV-19 regimen	Quantify antibodies against SARS-CoV-2 spike protein (seroconversion rates) post boost
	To assess immunogenicity of a delayed three dose ChAdOx1 nCoV-19 regimen	Quantify antibodies against SARS-CoV-2 spike protein (seroconversion rates) post third dose
	To compare viral shedding on stool samples of SARS-CoV-2 PCR* positive individuals	Differences in viral shedding on stool at 7 days and beyond post SARS-CoV-2 positivity.
	To assess immunological correlates of protection in relation to occurrence of COVID-19 disease in ChAdOx1 nCoV-19 recipients	Immunological endpoints and COVID-19 disease endpoints in ChAdOx1 nCoV-19 recipients

* Or other nucleic acid amplification test (NAAT)

Sample analysis for the completion of exploratory endpoints may be performed under the OVC Biobank research tissue bank protocol (REC: 16/SC/0141).

**Investigational products**	a) ChAdOx1 nCoV-19, a replication-deficient simian adenoviral vector expressing the spike (S) protein of SARS-CoV-2<br>b) MenACWY, Meningococcal Group A, C, W-135 and Y conjugate vaccine

**Formulation**	ChAdOx1 nCoV-19: Liquid<br><br>MenACWY: powder and solvent for solution for injection
**Route of Administration**	IM<br><br>ChAdOx1 nCoV-19/MenACWY: Intramuscularly (IM) into the deltoid region of the arm
**Dose per Administration**	ChAdOx1 nCoV-19: $5x10^{10}$ vp<br><br>ChAdOx1 nCoV-19: $2.5x10^{10}$ vp<br><br>ChAdOx1 nCoV-19: 0.5mL ($3.5-6.5x10^{10}$vp)<br><br>MenACWY: 0.5mL

2 ABBREVIATIONS

**AdHu**	Human adenovirus
**AdHu5**	Human adenovirus serotype 5
**AE**	Adverse event
**AID**	Autoimmune Disease
**CCVTM**	Centre for Clinical Vaccinology and Tropical Medicine, Oxford
**CBF**	Clinical BioManufacturing Facility
**CEF**	Chick embryo fibroblast
**ChAd63**	Chimpanzee adenovirus 63
**CI**	Confidence interval
**COP**	Code of Practice
**CRF**	Case Report Form or Clinical Research Facility
**CTRG**	Clinical Trials & Research Governance Office, Oxford University
**CTL**	Cytotoxic T Lymphocyte
**DSUR**	Development Safety Update Report
**ELISPOT**	Enzyme-linked immunospot
**GCP**	Good Clinical Practice
**GMO**	Genetically modified organism
**GMT**	Geometric Mean Titre
**GP**	General Practitioner
**HCG**	Human Chorionic Gonadotrophin
**HBV**	Hepatitis B virus
**HEK**	Human embryonic kidney
**HCV**	Hepatitis C virus
**HIV**	Human immunodeficiency virus
**HLA**	Human leukocyte antigen
**HRA**	Health Research Authority
**HTLV**	Human T-Lymphotrophic Virus
**IB**	Investigator Brochure
**ICH**	*International Conference on Harmonisation*
ICMJE	*International Committee of Medical Journal Editors*
ICS	*Intracellular Cytokine Staining*
**ID**	Intradermal
**IFNγ**	Interferon gamma
**IM**	Intramuscular
**IMP**	Investigational Medicinal Product
**IMP-D**	Investigational Medicinal Product Dossier
**IV**	Intravenous
**JCVI**	Joint Committee on Vaccination and Immunisation
**MenACWY**	Quadrivalent capsular group A, C, W and Y meningococcal protein-polysaccharide conjugate vaccine
**MHRA**	Medicines and Healthcare Products Regulatory Agency
**MVA**	Modified vaccinia virus Ankara
**NAAT**	Nucleic acid amplification test
**NHS**	National Health Service
**NIH**	National Institutes of Health
**NIHR**	National Institute for Health Research
**PBMC**	*Peripheral blood mononuclear cell*
**PCR**	Polymerase chain reaction

**PI**	Principal Investigator
**QP**	Qualified Person
**qPCR**	Quantitative polymerase chain reaction
**REC**	Research Ethics Committee
**SAE**	Serious adverse event
**SC**	Subcutaneous
**SmPc**	Summary of Product characteristics
**SOP**	Standard Operating Procedure
**SUSAR**	Suspected unexpected serious adverse reaction
**µg**	microgram
**vp**	viral particle
**VV**	viral vector
**WHO**	World Health Organisation

3 BACKGROUND AND RATIONALE

3.1 Background

In December 2019, a cluster of patients with pneumonia of unknown cause was linked to a seafood wholesale market in Wuhan, China and were later confirmed to be infected with a novel coronavirus, known as 2019-nCoV [1]. The virus was subsequently renamed to SARS-CoV-2 because it is similar to the coronavirus responsible for severe acute respiratory syndrome (SARS-CoV), a lineage B betacoronavirus. SARS-CoV-2 shares more than 79% of its sequence with SARS-CoV, and 50% with the coronavirus responsible for Middle East respiratory syndrome (MERS-CoV), a member of the lineage C betacoronavirus [2]. COVID-19 is the infectious disease caused by SARS-CoV-2. By January 2020 there was increasing evidence of human to human transmission as the number of cases rapidly began to increase in China. Despite unprecedented containment measures adopted by the Chinese government, SARS-CoV-2 rapidly spread across the world. The WHO declared the COVID-19 outbreak a public health emergency of international concern on 30th January 2020. As of 10th March 2020, over 118,000 cases have been reported with more than 4200 deaths and 115 countries affected.

Coronaviruses (CoVs) are spherical, enveloped, large positive-sense single-stranded RNA genomes. One-fourth of their genome is responsible for coding structural proteins, such as the spike (S) glycoprotein, envelope (E), membrane (M) and nucleocapsid (N) proteins. E, M, and N are mainly responsible for virion assembly whilst the S protein is involved in receptor binding, mediating virus entry into host cells during CoVs infection via different receptors.[3] SARS-CoV-2 belongs to the phylogenetic lineage B of the genus *Betacoronavirus* and it recognises the angiotensin-converting enzyme 2 (ACE2) as the entry receptor [4]. It is the seventh CoV known to cause human infections and the third known to cause severe disease after SARS-CoV and MERS-CoV.

The spike protein is a type I, trimeric, transmembrane glycoprotein located at the surface of the viral envelope of CoVs, which can be divided into two functional subunits: the N-terminal S1 and the C-terminal S2. S1 and S2 are responsible for cellular receptor binding via the receptor binding domain (RBD) and fusion of virus and cell membranes respectively, thereby mediating the entry of SARS-CoV-2 into target cells.[3] The roles of S in receptor binding and membrane fusion make it an ideal target for vaccine and antiviral development, as it is the main target for neutralising antibodies.

ChAdOx1 nCoV-19 vaccine consists of the replication-deficient simian adenovirus vector ChAdOx1, containing the structural surface glycoprotein (Spike protein) antigen of the SARS CoV-2 (nCoV-19), with a leading tissue plasminogen activator (tPA) signal sequence. ChAdOx1 nCoV-19 expresses a codon-optimised coding sequence for the Spike protein from genome sequence accession GenBank:

MN908947. The tPA leader sequence has been shown to be beneficial in enhancing immunogenicity of another ChAdOx1 vectored CoV vaccine (ChAdOx1 MERS) [5].

3.2 Pre-Clinical Studies

Refer to the Investigator Brochure for most recent pre-clinical data update

3.2.1 Immunogenicity (Jenner Institute, unpublished)

Mice (balb/c and CD-1) were immunised with ChAdOx1 expressing SARS-CoV-2 Spike protein or green fluorescent protein (GFP). Spleens were harvested for assessment of IFY ELISpot responses and serum samples were taken for assessments of S1 and S2 antibody responses on ELISA at 9 or 10 days post vaccination. The results of this study show that a single dose of ChAdOx1 nCoV was immunogenic in mice.

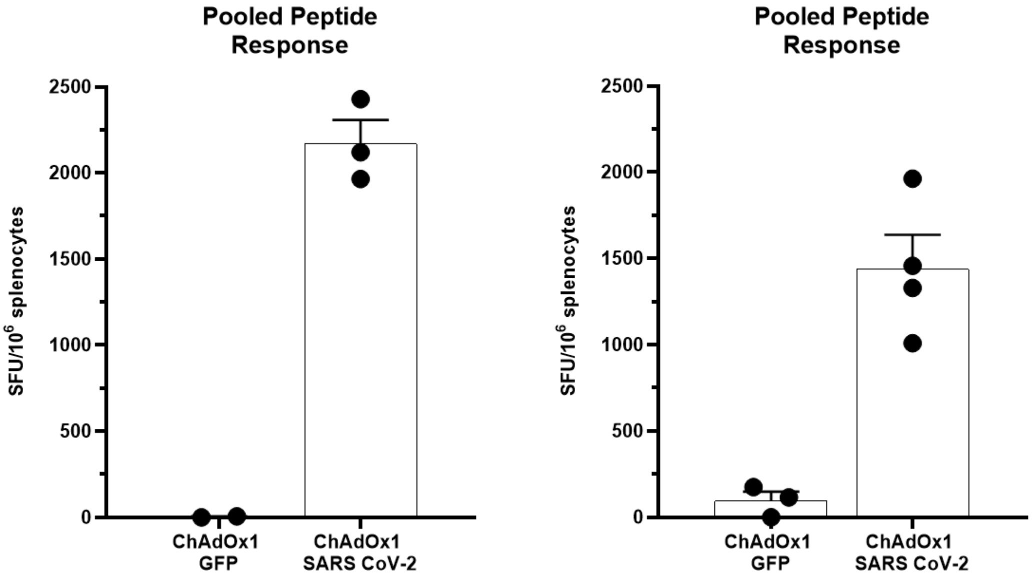

Figure 1. Summed splenic IFN-γ ELISpot responses of BALB/c (left panel) and CD-1 (right panel) mice, in response to peptides spanning the spike protein from SARS-CoV-2, nine or ten days post vaccination, with 1.7×10^{10} vp ChAdOx1 nCoV-19 or 8×10^{9} vp ChAdOx1 GFP. Mean with SEM are depicted

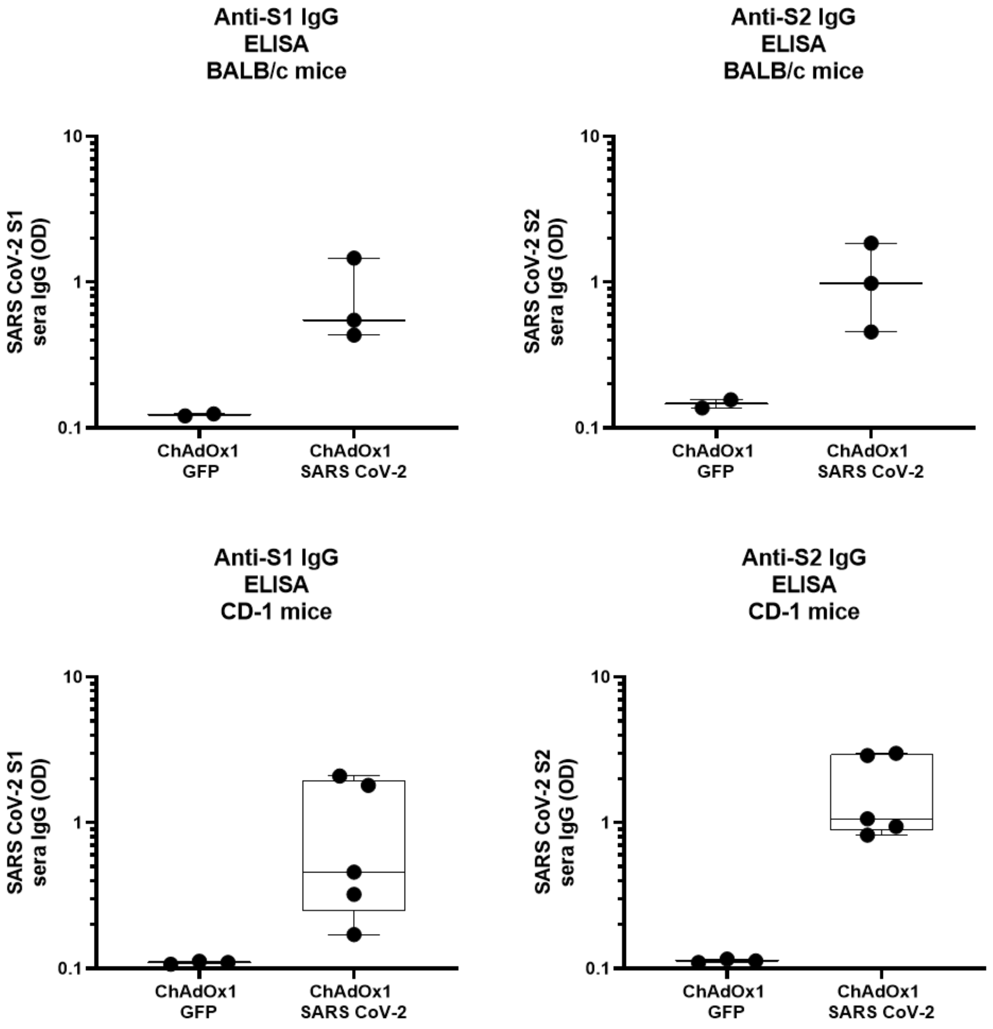

Figure 2. Box and whisker plot of the optical densities following ELISA analysis of BALB/C mouse sera (Top panel) incubated with purified protein spanning the S1 domain (left) or purified protein spanning the S2 domain (right) of the SARS-CoV-2 spike nine or ten days post vaccination, with 1.7×10^{10} vp ChAdOx1 nCoV-19 or 8×10^{9} vp ChAdOx1 GFP. Box and whisker plots of the optical densities following ELISA analysis of CD-1 mouse sera (Bottom panel) incubated with purified protein spanning the S1 domain (left) or purified protein spanning the S2 domain (right) of the SARS-CoV-2 spike.

3.2.2 Efficacy

Pre-clinical efficacy studies of ChAdOx1 nCoV-19 in ferrets and non-human primates are underway. Results will be included in the Investigator's Brochure when available.

3.3 Disease Enhancement

Safety concerns around the use of full length coronavirus Spike glycoproteins and other viral antigens (nucleoprotein) as a vaccine antigen have been raised following historical and limited reports of

immunopathology and antibody dependant enhancement (ADE) reported in vitro and post SARS-CoV challenge in mice, ferrets and non-human primates immunised with whole SARS-CoV inactivated or full-length S protein based vaccines, including a study using Modified Vaccinia Ankara as a vector.[6-8] To date, there has been one report of lung immunopathology following MERS-CoV challenge in mice immunised with an inactivated MERS-CoV candidate vaccine.[9] However, in preclinical studies of ChAdOx1 immunisation and MERS-CoV challenge, no ADE was observed in hDPP4 transgenic mice, dromedary camels or non-human primates (van Doremalen et al, manuscript submitted).[10, 11]

The risks of inducing lung immunopathology in the event of COVID-19 disease following ChAdOx1 nCoV-19 vaccination are unknown. Challenge studies on ferrets and NHPs are underway and these pre-clinical studies will report on presence or absence of lung pathology. Results will be reviewed as soon as they emerge and will inform discussions on risk/benefit to participants receiving the IMP. All pathology data arising from challenge studies of other SARS-CoV-2 vaccine candidates will also be taken into account.

3.4 Previous clinical experience

This will be the first-in-human study employing ChAdOx1 nCoV-19. However, ChAdOx1 vectored vaccines expressing different inserts have previously been used in over 320 healthy volunteers taking part in clinical trials conducted by or in partnership with the University of Oxford in the UK and overseas (table 1 and 2). Most importantly, a ChAdOx1 vectored vaccine expressing the full-length Spike protein from another Betacoronavirus, MERS-CoV, has been given to 31 participants to date as part of MERS001 and MERS002 trials. ChAdOx1 MERS was given at doses ranging from $5x10^9$ vp to $5x10^{10}$ vp (table 2) with no serious adverse reactions reported. Further safety and immunogenicity results on ChAdOx1 MERS can be found on the Investigator's Brochure for ChAdOx1 nCoV-19 for reference.

Clinical trials of ChAdOx1 vectored vaccines encoding antigens for Influenza (fusion protein NP+M1), Tuberculosis (85A), Prostate Cancer (5T4), Malaria (LS2), Chikungunya (structural polyprotein), Zika (prM and E), MERS-CoV (full-length Spike protein) and Meningitis B are listed below.

None of the below mentioned clinical trials reported serious adverse events associated with the administration of ChAdOx1, which was shown to have a good safety profile.

Table 1. Clinical experience with ChAdOx1 viral vector vaccines.

Country	Trial	Vaccine	Age	Route	Dose	Number of Volunteers (Received ChAdOx1)	Publication / Registration Number
UK	FLU004	ChAdOx1 NP+M1	18-50	IM	$5x10^{8}$ vp	3	Antrobus et al, 2014. Molecular Therapy. DOI: 10.1038/mt.2013.284 [12]
					$5x10^{9}$ vp	3	
					$2.5x10^{10}$ vp	3	
					$5x10^{10}$ vp	6	
UK	FLU005	ChAdOx1 NP+M1<br>MVA NP+M1 (week 8)	18-50	IM	$2.5x10^{10}$ vp	12	Coughlan et al, 2018. EBioMedicine DOI: 10.1016/j.ebiom.2018.02.011 DOI: 10.1016/j.ebiom.2018.05.001 [13]
		ChAdOx1 NP+M1<br>MVA NP+M1 (week 52)	18-50	IM	$2.5x10^{10}$ vp	12	
		MVA NP+M1<br>ChAdOx1 NP+M1 (week 8)	18-50	IM	$2.5x10^{10}$ vp	12	
		MVA NP+M1<br>ChAdOx1 NP+M1 (week 52)	18-50	IM	$2.5x10^{10}$ vp	9	
		ChAdOx1 NP+M1	>50	IM	$2.5x10^{10}$ vp	12	
		ChAdOx1 NP+M1<br>MVA NP+M1 (week 8)	>50	IM	$2.5x10^{10}$ vp	12	
UK	TB034	ChAdOx1 85A	18-50	IM	$5x10^{9}$ vp	6	Wilkie et al, 2020 Vaccine

Country	Trial	Vaccine	Age	Route	Dose	Number of Volunteers (Received ChAdOx1)	Publication / Registration Number
					$2.5x10^{10}$ vp	12	DOI: 10.1016/j.vaccine.2019.10.102 [14]
		ChAdOx1 85A MVA85A (week 8)	18-50	IM	$2.5x10^{10}$ vp	12	
		ChAdOx1 85A (x2, 4weeks apart) MVA85A (at 4 months)	18-50	IM	$2.5x10^{10}$ vp	12	
Switzerland	TB039 (ongoing)	ChAdOx1 85A	18-55	Aerosol	$1x10^{9}$ vp	3	Clinicaltrials.gov: NCT04121494
				Aerosol	$5x10^{9}$ vp	3	
				Aerosol	$1x10^{10}$ vp	11	
				Aerosol/IM	$1x10^{10}$ vp	15	
Uganda	TB042 (ongoing)	ChAdOx1 85A	18-49	IM	$5x10^{9}$ vp	6	Clinicaltrials.gov: NCT03681860
					$2.5\ x10^{10}$	6	
UK	VANCE01	ChAdOx1.5T4 MVA.5T4	18 – 75	IM	$2.5x10^{10}$ vp	34	Clinicaltrials.gov: NCT02390063
UK	ADVANCE	ChAdOx1.5T4	≥18	IM	$2.5x10^{10}$ vp	23 (as of Feb 20)	Clinicaltrials.gov:

Country	Trial	Vaccine	Age	Route	Dose	Number of Volunteers (Received ChAdOx1)	Publication / Registration Number
	(ongoing)	MVA.5T4					NCT03815942
UK	VAC067	ChAdOx1 LS2	18-45	IM	$5x10^9$ vp<br>$2.5x10^{10}$ vp	3<br>10	Clinicaltrials.gov:<br>NCT03203421
UK	VAMBOX	ChAdOx1 MenB.1	18-50	IM	$2.5x10^{10}$ vp<br>$5x10^{10}$ vp	3<br>26	ISRCTN46336916
UK	CHIK001	ChAdOx1 Chik	18-50	IM	$5x10^9$ vp<br>$2.5x10^{10}$ vp<br>$5x10^{10}$ vp	6<br>9<br>9	Clinicaltrials.gov:<br>NCT03590392<br>DOI: https://doi.org/10.4269/ajtmh.abstract2019<br>Abstract #59, page 19.
UK	ZIKA001<br>(ongoing)	ChAdOx1 Zika	18-50	IM	$5x10^9$ vp<br>$2.5x10^{10}$ vp<br>$5x10^{10}$ vp	6<br>3 (as of Feb 20)<br>-	Clinicaltrials.gov:<br>NCT04015648

Table 2. Clinical experience with ChAdOx1 MERS

**Country**	**Trial**	**Vaccine**	**Age**	**Route**	**Dose**	**Number of Volunteers (Received ChAdOx1)**	**Publication / Registration Number**
UK	MERS001 (ongoing)	ChAdOx1 MERS	18-50	IM	$5x10^9$ vp<br>$2.5x10^{10}$ vp<br>$5x10^{10}$ vp<br>$2.5x10^{10}$ vp (homologous prime-boost)	6<br>9<br>9<br>3	Clinicaltrials.gov: NCT03399578<br>DOI: https://doi.org/10.1016/S1473-3099(20)30160-2<br>Folegatti et.al. 2020, Lancet Infect.Dis. **[15]**
Saudi Arabia	MERS002 (ongoing)	ChAdOx1 MERS	18-50	IM	$5x10^9$ vp<br>$2.5x10^{10}$ vp<br>$5x10^{10}$ vp	4<br>3<br>-	Clinicaltrials.gov: NCT04170829

3.5 Rationale

The COVID-19 epidemic has caused major disruption to healthcare systems with significant socioeconomic impacts. Containment measures have failed to stop the spread of virus, which is now at pandemic levels. There are currently no specific treatments available against COVID-19 and accelerated vaccine development is urgently needed.

Live attenuated viruses have historically been among the most immunogenic platforms available, as they have the capacity to present multiple antigens across the viral life cycle in their native conformations. However, manufacturing live-attenuated viruses requires complex containment and biosafety measures. Furthermore, live-attenuated viruses carry the risks of inadequate attenuation causing disseminated disease, particularly in immunocompromised hosts. Given that severe disease and fatal COVID-19 disproportionally affect older adults with co-morbidities, making a live-attenuated virus vaccine is a less viable option. Replication competent viral vectors could pose a similar threat for disseminated disease in the immuno-suppressed. Replication deficient vectors, however, avoid that risk while maintaining the advantages of native antigen presentation, elicitation of T cell immunity and the ability to express multiple antigens [16]. Subunit vaccines usually require the use of adjuvants and whilst DNA and RNA vaccines can offer manufacturing advantages, they are often poorly immunogenic requiring multiple doses, which is highly undesirable in the context of a pandemic.

Chimpanzee adenovirus vaccine vectors have been safely administered to thousands of people using a wide range of infectious disease targets. ChAdOx1 vectored vaccines have been given to over 320 volunteers with no safety concerns and have been shown to be highly immunogenic at single dose administration. Of relevance, a single dose of a ChAdOx1 vectored vaccine expressing full-length spike protein from another betacoronavirus (MERS-CoV) has shown to induce neutralising antibodies in recent clinical trials.

Data generated in this study will be used to support further larger phase II/III efficacy studies, which will include target groups at higher risk of severe disease.

The use of an active comparator (MenACWY) will minimise the chances of accidental participant unblinding, decreasing bias in reactogenicity or safety reporting and/or health seeking behaviours once symptomatic for COVID-19. The use of prophylactic paracetamol reduces the incidence and severity of fever and other adverse events following immunisation, and it has been previously recommended following Meningococcal B vaccine administration without negatively impacting its immunogenicity profile (reference: Bexsero SmPC). A prophylactic paracetamol dose arm has been introduced in order to assess safety, reactogenicity, immunogenicity and efficacy of the co-administration of paracetamol and ChAdOx1 nCoV-19 as an exploratory objective.

Whilst a single-dose regimen is the preferred option in the context of a pandemic, a two-dose schedule is likely to boost seroconversion rates and increase neutralising antibody levels, although correlates of protection for COVID-19 are still unknown. Groups 2c, d, and e have been added in order to gather additional evidence on immunogenicity of ChAdOx1 nCoV-19 given as part of the two-dose schedule.

A booster dose has been added to participants in groups 2 and 4 following interim immunogenicity results on homologous prime-boost groups, showing improved neutralising antibody titres after 2 doses when compared to a 1 dose regimen.

Late / Yearly Booster Vaccination (Protocol v15.0)

An urgent public health question related to COVID-19 vaccination that remains unanswered is whether seasonal booster vaccinations may be required in order to further boost immune responses and enhance protection against the SARS-CoV-2 virus. However, the impact on transgene responses of an annual vaccination with the same adenoviral vector remains uncertain. Concerns exist that repeated doses of adenoviral vaccine vectors may generate anti-vector immunity which may impede responses to the pathogen transgene although we have previously shown that anti-ChAdOx1 vector antibodies do not impact on anti-spike antibody responses or spike specific T-cell responses{Ramasamy, 2020 #83} and also that individuals who have received a prior ChAdOx1-vectored vaccine for a non-spike transgene at least one year prior to receipt of ChAdOx1 nCoV-19 have similar binding antibody responses to spike protein compared to ChAdOx1-naïve individuals{Emary, 2021 #82}.

The addition of the subgroups 1c, 1d, 5a and 5b to the study will enable the assessment of annual boosting and late boosting on safety and immunogenicity outcomes. This will be achieved by reallocating participants into the following subgroups:

- A 2 x ChAdOx1 group with a long interval between the first and second dose. This will be achieved by boosting group 1a with ChAdOx1, creating a new subgroup “1c”.
- 3 x ChAdOx1 dose groups with a long interval between the second and third dose. This will be achieved by boosting groups 2c, 2f and 4c with ChAdOx1, creating a new subgroup “5a”. Group 3 (unblinded two-dose 4-week interval ChAdOx1 nCoV-19 group) will also be given a third dose creating group “3b”. The first-to-second dose intervals amongst groups 3 (4 weeks); 2c, 2e (8 weeks) and 2f, 2g, 4c, 4d (approx. 16 weeks) will enable an assessment of *third* dose outcomes in individuals matching the currently MHRA approved 4-12 week schedule.
- To allow for a comparison of a ChAdOx1 prime vaccination with both the two-dose and three-dose late boost groups, participants that were originally randomised to the corresponding MenACWY arms of the above groups (1b,2e, 2g and 4d) will now receive an ChAdOx1 vaccination at the late vaccination visit, creating subgroups 1d and 5b. (Additionally, in order to receive the currently

recommended 2-dose ChAdOx1 vaccine schedule, these participants will also be offered a ChAdOx1 boost at 12 weeks following their ChAdOx1 prime vaccination - the “Late Vacc Two” visit)

4 OBJECTIVES AND ENDPOINTS

Objectives	Outcome Measures	Time point(s) of evaluation of this outcome measure
**Primary Objective**<br>To assess efficacy of ChAdOx1 nCoV-19 against COVID-19	a) Virologically confirmed (PCR* positive) symptomatic cases of COVID-19	Throughout the study
**Co-primary Objective**<br>To assess the safety of the candidate vaccine ChAdOx1 nCoV	a) occurrence of serious adverse events (SAEs) throughout the study until a cutoff date of 1st July 2021 or 6 months post late vaccination visit, whichever is latest	Throughout the study until a cutoff date of 1st July 2021 or 6 months post late vaccination visit, whichever is latest
**Secondary Objectives**<br>To assess the safety, tolerability and reactogenicity profile of the candidate vaccine ChAdOx1 nCoV	a) occurrence of solicited local reactogenicity signs and symptoms for 7 days following vaccination<br><br>b) occurrence of solicited systemic reactogenicity signs and symptoms for 7 days following vaccination;<br><br>c) occurrence of unsolicited adverse events (AEs) for 28 days following vaccination; (7 days following vaccination for groups 1c,1d,5a and 5b)<br>d) change from baseline for safety laboratory measures and;<br><br>f) Occurrence of SAE of special interest: disease enhancement episodes	Day 0-7<br>Self-reported symptoms recorded using electronic diaries<br><br>Day 0-7<br>Self-reported symptoms recorded using electronic diaries<br><br>Day 0-28<br>Self-reported symptoms recorded using electronic diaries<br><br>Blood samples drawn at enrolment (before vaccination), day 3, 7 and 28<br><br>Throughout the study
To assess efficacy of ChAdOx1 nCoV-19 against COVID-19	a) Hospital admissions associated with COVID-19	Throughout the study<br><br>Throughout the study

	b) Intensive care unit (ICU) admissions associated with COVID-19<br>c) Deaths associated with COVID-19<br>d) Severe COVID-19 disease (defined according to clinical severity scales)<br><br>e) Seroconversion against non-Spike antigens	Throughout the study<br><br>Throughout the study<br><br>Blood samples drawn at D0, D28 , D182 and D364
To assess cellular and humoral immunogenicity of ChAdOx1 nCoV-19	a) Interferon-gamma (IFN-γ) enzyme-linked immunospot (ELISpot) responses to SARS-CoV-2 spike protein;<br><br>b) Quantify antibodies against SARS-CoV-2 spike protein (seroconversion rates)	See schedule of attendances
**Exploratory Objectives**<br>Exploratory Immunology	a) virus neutralising antibody (NAb) assays against live and/or pseudotype SARS-CoV-2 virus<br>b) Cell analysis by flow cytometry assays<br>c) Functional antibody assays	See schedule of attendances
To assess safety, reactogenicity, immunogenicity and efficacy endpoints, for participants receiving prophylactic paracetamol	All safety, reactogenicity, immunogenicity and efficacy endpoints.	Throughout the study
To assess immunogenicity of ChAdOx1 nCoV-19 given as homologous prime-boost	Quantify antibodies against SARS-CoV-2 spike protein (seroconversion rates) post boost	Blood samples drawn at D0, D28, D56, D70, D84, D182 and D364
To assess immunogenicity of a delayed prime-boost ChAdOx1 nCoV-19 regimen	Quantify antibodies against SARS-CoV-2 spike protein (seroconversion rates) post boost	Blood samples drawn at LV14, LV28 and LV182

To assess immunogenicity of a delayed three dose ChAdOx1 nCoV-19 regimen	Quantify antibodies against SARS-CoV-2 spike protein (seroconversion rates) post boost	Blood samples drawn at LV14, LV28 and LV182
To compare viral shedding on stool samples of SARS-CoV-2 PCR* positive individuals	Differences in viral shedding on stool between vaccine and comparator arms	At approximately 7 days and beyond post SARS-CoV-2 PCR* positivity.
To assess immunological correlates of protection in relation to occurrence of COVID-19 disease in ChAdOx1 nCoV-19 recipients	Immunological endpoints and COVID-19 disease endpoints in ChAdOx1 nCoV-19 recipients	Throughout the study

* Or other nucleic acid amplification test

Sample analysis for the completion of exploratory endpoints may be performed under the ethically approved OVC Biobank protocol.

5 TRIAL DESIGN

This is a Phase I/II, single-blinded, -controlled, individually randomised study in healthy adults aged 18-55 years recruited in the UK. ChAdOx1 nCoV-19 or active control (licensed MenACWY) will be administered via an intramuscular injection into the deltoid. The study will assess efficacy, safety and immunogenicity of ChAdOx1 nCoV-19. Additional steps may be taken to keep clinical investigators assessing the primary efficacy endpoint blinded to group allocation with an aim to minimise unblinding of participants, where this is possible and practical to do so.

There will be 4 study groups with up to 540 volunteers in each of the single dose vaccine arms (ChAdOx1 nCoV-19 or licensed MenACWY) in groups 1, 2 & 4 combined and 10 participants in group 3 with an overall sample size of up to 1090 (Table 3). Randomisation will take place at an intervention to control ratio of 1:1. Only participants enrolled in groups 1, 2 and 4 will be randomised. Participants in group 3 will not be randomised or blinded. Up to 112 participants in group 4 will be requested to take prophylactic paracetamol 1000mg every 6 hours for 24 hours from the time of vaccination to reduce the chance of fever post immunisation. Participants receiving a booster dose in groups 2f, 2g, 4c and 4d will be advised to take prophylactic paracetamol for 24h post booster vaccine.

Staggered enrolment will apply to the first volunteers receiving the IMP as described in section 7.4.2.2. Participants will be first recruited in groups 1 and 3. Once groups 1 and 3 are fully recruited, subsequent volunteers will be enrolled in groups 2 and 4.

Safety will be assessed in real time and interim reviews are scheduled after 1, 4, and up to 54 participants received the IMP. Randomisation blocks will ensure there is at least 1 control for each participant receiving the IMP, so these safety reviews will take place after 2, 8, and up to 98 participants are enrolled in the study overall (groups 1 and 3).

Up to 62 participants enrolled in group 2 (a and b) will be invited to receive a booster vaccine. Participants in groups 2c and 2d will be randomised to receive either a standard booster dose ($5x10^{10}$vp), or a lower booster dose ($2.5x10^{10}$vp) at approximately 8 weeks post prime. Up to 10 volunteers from 2b will be receive a second dose of MenACWY at the same interval. The remaining participants in groups 2 and 4 (those who have not already been boosted) will be invited to receive a booster dose of either ChAdOx1 nCoV-19: 0.5mL (3.5-6.5x1010vp) or MenACWY. Volunteers in groups 2f, 2g, 4c and 4d will be advised to take prophylactic paracetamol for 24h post booster dose.

The DSMB will periodically assess safety and efficacy data every 4-8 weeks and/or as required.

Participants will be followed over the duration of the study to record adverse events and episodes of virologically confirmed symptomatic COVID-19 cases. Participants will be tested for COVID-19 if they present with a new onset of fever (≥37.8 C) OR cough OR shortness of breath OR anosmia/ageusia.

Moderate and Severe COVID-19 disease will be defined using clinical criteria. Detailed clinical parameters will be collected from medical records and aligned with agreed definitions as they emerge. These are likely to include, but are not limited to, oxygen saturation, need for oxygen therapy, respiratory rate and other vital signs, need for ventilatory support, Xray and CT scan imaging and blood test results, amongst other clinically relevant parameters.

5.1 Study groups

Group	Number of Volunteers	Dose	Route
1a	44	ChAdOx1 nCoV-19<br>$5x10^{10}$ vp	IM
1b	44	MenACWY	IM
1c	Up to 44 from group 1a	Delayed homologous ChAdOx1 nCoV-19<br>Prime-Boost	IM
1d	Up to 22 from group 1b	Two-dose MenACWY, Two-dose ChAdOx1 nCoV-19	IM
2a*	Up to 206	ChAdOx1 nCoV-19<br>$5x10^{10}$ vp	IM
2b*	Up to 206	MenACWY	IM
2c*	Up to 20 volunteers from 2a	Homologous Prime-Boost<br>$5x10^{10}$ vp, 8 weeks apart (-7/+14 days)	IM
2d*	Up to 32 volunteers from 2a	Homologous Prime-Boost<br>$5x10^{10}$ vp (prime) and $2.5x10^{10}$ vp (boost) 8 weeks apart (-7/+14 days)	IM

2e*	Up to 10 volunteers from 2b	Two-dose MenACWY 8 weeks apart (-7/+14 days)	IM
2f*	Up to 154 volunteers from 2a	Homologous Prime-Boost<br>$5x10^{10}$ vp prime and 0.5mL ($3.5-6.5x10^{10}$vp) boost, at the earliest opportunity (minimum 4 weeks apart)	IM
2g*	Up to 196 volunteers from 2b	Two-dose MenACWY at the earliest opportunity (minimum 4 weeks apart)	IM
3a	10	Homologous Prime-Boost<br>$5x10^{10}$ vp	IM
3b	Up to 10 from 3a	Delayed three-dose ChAdOx1 nCoV-19	IM
4a **	Up to 290	ChAdOx1 nCoV-19<br>$5x10^{10}$ vp	IM
4b**	Up to 290	MenACWY	IM
4c**	Up to 290 from 4a	Homologous Prime-Boost<br>$5x10^{10}$ vp prime and 0.5mL ($3.5-6.5x10^{10}$vp) boost, at the earliest opportunity (minimum 4 weeks apart)	IM
4d**	Up to 290 from 4b	Two-dose MenACWY at the earliest opportunity (minimum 4 weeks apart)	IM
5a	Up to 80 from 2c, 2f, 4c	Delayed three-dose ChAdOx1 nCoV-19	IM
5b	Up to 40 from 2e, 2g and 4d	Two-dose MenACWY, Two-dose ChAdOx1 nCoV-19	IM

* Group 2 will consist of an overall sample size of up to 412 volunteers, of which up to 62 (52 IMP and 10 controls) will receive a booster dose at 8 weeks (-7/+14 days), and the remainder will be invited to receive a booster dose at the earliest opportunity (minimum 4 weeks apart).

**Group 4 will consist of an overall sample size of up to 580 volunteers, of which up to 112 will be given Paracetamol at D0 visit. All volunteers in Group 4 will be invited to receive a booster dose at the earliest opportunity (minimum 4 weeks apart).

5.2 Trial volunteers

Healthy adult volunteers aged 18-55 will be recruited into the study. Volunteers will be considered enrolled immediately following administration of first vaccination.

5.3 Definition of End of Trial

The end of the trial is the date of the last assay conducted on the last sample collected.

5.4 Duration of study

The total duration of the study will be 12 months from the day of the last vaccination dose (6 months post the Late Vaccination visit for groups 1c, 1d, 4e.)

5.5 Potential Risks for volunteers

The potential risks are those associated with phlebotomy, vaccination and disease enhancement

Venepuncture

Localised bruising and discomfort can occur at the site of venepuncture. Infrequently fainting may occur. These will not be documented as AEs if they occur. The total volume of blood drawn over a six month period will be 177.5-621.5mL (blood volumes may vary slightly for volunteers at different investigator sites due to use of different volume vacutainers, following local Trust SOPs). This should not compromise these otherwise healthy volunteers, as they would donate 470mL during a single blood donation for the National Blood transfusion Service over a 3-4 month period.

As of protocol v15.0, participants will be permitted to donate blood to transfusion services from 3 months after the date of their final planned study vaccination. This replaces the previous instruction that volunteers should refrain from blood donation for the duration of their involvement in the trial. 3 months has been selected to provide adequate spacing between study visits and blood donation in order to remain within the recommended limits for blood donation in the UK and to comply with the Joint United Kingdom (UK) Blood Transfusion and Tissue Transplantation Services Professional Advisory Committee requirement for a minimum

period of 7 days between ChAdOx1 nCov-19 vaccination and blood donation (Joint United Kingdom (UK) Blood Transfusion and Tissue Transplantation Services Professional Advisory Committee: Change Notification UK National Blood Services No. 73 - 2020).

Allergic reactions

Allergic reactions from mild to severe may occur in response to any constituent of a medicinal product's preparation. Anaphylaxis is extremely rare (about 1 in 1,000,000 vaccine doses) but can occur in response to any vaccine or medication.

Vaccination

Local reaction from IM vaccination

The typical local reaction as a result of IM injection is temporary pain, tenderness, redness, and swelling at the site of the injection.

Systemic reactions

Constitutional influenza-like symptoms such as fatigue, headache, malaise, feverishness, and muscle aches can occur with any vaccination and last for 2-3 days. Presyncopal and syncopal episodes may occur at the time of vaccination which rapidly resolve. For subset of participants in group 4, use of prophylactic paracetamol for 24 hours will be advised to alleviate potential fevers and flu-like symptoms. As with any other vaccine, temporary ascending paralysis (Guillain-Barré syndrome, GBS) or immune mediated reactions that can lead to organ damage may occur, but this should be extremely rare (1 in 100,000-1,000,000 vaccine doses).

Control participants will receive one or two doses of a licensed MenACWY vaccine, the risks of which are described in these vaccines SmPC.

Disease Enhancement

The risks of inducing disease enhancement and lung immunopathology in the event of COVID-19 disease following ChAdOx1 nCoV-19 vaccination are unknown as described above. Challenge studies on ferrets and NHPs are underway and results will be reviewed as they emerge. All pre-clinical data from challenge studies using ChAdOx1 nCoV-19 and other vaccine candidates (when available) will inform decisions on risk/benefit to participants receiving the IMP.

Emerging Thrombosis with Thrombocytopenia Association with vaccination

The MHRA and JCVI issued updated guidance regarding the use of ChAdOx1 nCoV-19 on 7th April 2021, following a review of extremely rare reports of cerebral venous sinus thrombosis (and thrombosis of other major veins) with concurrent thrombocytopenia that have occurred after vaccination in the national rollout

programme. This recommends that currently, in the UK setting, alternative vaccinations against COVID-19 should be preferentially offered to individuals aged 29 and under.

Prior to any further participant receiving ChAdOx1 nCoV-19 in this trial, they will be provided with up-to-date information from regulators on this finding via the updated participant information sheet. They will also be provided with other relevant documentation from regulators and/or public health authorities related to this association and possible risks of vaccination that is also being provided in vaccination centres. Participants will be advised to be aware of possible signs and symptoms of blood clots and to have a low threshold to contact trial teams if experiencing these or other symptoms.

Following this recommendation, no further ChAdOx1 nCoV-19 prime vaccination doses will be administered to participants aged 29 and under within this trial. This age restriction recommendation does not apply to booster vaccinations which may therefore continue to be administered within the trial to participants of any age.

5.6 Known Potential Benefits

Volunteers enrolled into the control groups will receive 1-2 doses of MenACWY, a licensed vaccine that has been administered to teenagers in the UK routine schedule since 2015 and is used as a travel vaccine for high risk areas. The majority of participants in this study will not have had this vaccine previously, and therefore will gain the benefit of protection against group A, C, W and Y meningococcus. Those participants who have previously had MenACWY vaccines will have their immunity against these organisms boosted. Recipients of ChAdOx1 nCoV-19 do not have any guaranteed benefit. However, it is hoped that the information gained from this study will contribute to the development of a safe and effective vaccine against COVID-19. The only benefits for participants would be information about their general health status.

6 RECRUITMENT AND WITHDRAWAL OF TRIAL VOLUNTEERS

6.1 Identification of Trial Volunteers

Healthy adults in the UK will be recruited by use of an advertisement +/- registration form formally approved by the ethics committee(s) and distributed or posted in the following places:

- In public places, including buses and trains, with the agreement of the owner / proprietor.
- In newspapers or other literature for circulation.
- On radio via announcements.
- On a website or social media site operated by our group or with the agreement of the owner or operator (including on-line recruitment through our web-site).
- By e-mail distribution to a group or list only with the express agreement of the network administrator or with equivalent authorisation.
- By email distribution to individuals who have already expressed an interest in taking part in any clinical trial at the Oxford Vaccine Centre and other trial sites.
- On stalls or stands at exhibitions or fairs.
- Via presentations (e.g. presentations at lectures or invited seminars).
- Direct mail-out: This will involve obtaining names and addresses of adults via the most recent Electoral Roll. The contact details of individuals who have indicated that they do not wish to receive postal mail-shots would be removed prior to the investigators being given this information. The company providing this service is registered under the General Data Protection Regulation 2016/679. Investigators would not be given dates of birth or ages of individuals but the list supplied would only contain names of those aged between 18-55 years (as per the inclusion criteria).
- Direct mail-out using National Health Service databases: These include the National Health Applications and Infrastructure Services (NHAIS) via a NHAIS data extract or equivalent. Initial contact to potential participants will not be made by the study team. Instead study invitation material will be sent out on our behalf by an external company, CFH Docmail Ltd, in order to preserve the confidentiality of potential participants. CFH Docmail Ltd is accredited as having exceeded standards under the NHS Digital Data Security and Protection Toolkit (ODS ID – 8HN70).
- Oxford Vaccine Centre databases and other trial sites databases: We may contact individuals from databases of groups within the CCVTM (including the Oxford Vaccine Centre database) and other trial

sites of previous trial participants who have expressed an interest in receiving information about all future studies for which they may be eligible.

6.2 Informed consent

All volunteers will sign and date the informed consent form before any study specific procedures are performed. The information sheet will be made available to the volunteer at least 24 hours prior to the screening visit. At the screening visit, a video presentation of the aims of the study and all tests to be carried out may be screened to an audience, or made available for them to access it remotely. Individually each volunteer will have the opportunity to question an appropriately trained and delegated researcher before signing the consent. At the screening visit, the volunteer will be fully informed of all aspects of the trial, the potential risks and their obligations. The following general principles will be emphasised:

- Participation in the study is entirely voluntary
- Refusal to participate involves no penalty or loss of medical benefits
- The volunteer may withdraw from the study at any time.
- The volunteer is free to ask questions at any time to allow him or her to understand the purpose of the study and the procedures involved
- The study involves research of an investigational vaccine
- There is no direct benefit to the volunteer from participating
- The volunteer's GP will be contacted to corroborate their medical history. Written or verbal information regarding the volunteer's medical history will be sought from the GP or other sources. This can either be via the study team accessing patient's electronic care summaries, GP and other medical records from local systems, by contacting the GP practice, or volunteers bringing their medical care summaries from the GP to the study clinicians. However, volunteers may be enrolled based on medical information obtained during screening only, at the physician's discretion.
- Blood samples taken as part of the study may be sent outside of the UK and Europe to laboratories in collaboration with the University of Oxford. These will be anonymised. Volunteers will be asked if they consent to indefinite storage of any leftover samples for use in other ethically approved research, this will be optional.
- The volunteer will be registered on the TOPS database (The Over volunteering Prevention System; www.tops.org.uk).

The aims of the study and all tests to be carried out will be explained. The volunteer will be given the opportunity to ask about details of the trial, and will then have time to consider whether or not to participate. If they do decide to participate, they, and the investigator will sign and date the consent form. However, in the current crisis, there may be occasions when it is necessary for the consent form to be signed by an appropriately trained and delegated research nurse instead of the investigator. The participant would always have the opportunity to discuss the study with a medically qualified investigator if they wish. The volunteer will then be provided with a copy of the consent form to take away and keep, with the original being stored in the case report form (CRF). Reconsent will be taken by appropriately trained and delegated members of the team.

Participants are required to consent to receive an additional swab should they develop symptoms of COVID-19. Updated information will be sent to participants and written re-consent requested at the earliest scheduled visit. If the earliest visit to occur is in the symptomatic pathway, the participant may consent using an electronic signature on a tablet for infection control purposes.

6.3 Inclusion and exclusion criteria

This study will be conducted in healthy adults, who meet the following inclusion and exclusion criteria:

6.3.1 Inclusion Criteria

The volunteer must satisfy all the following criteria to be eligible for the study:

- Healthy adults aged 18-55 years.
- Able and willing (in the Investigator's opinion) to comply with all study requirements (participants must not rely on public transport or taxis).
- Willing to allow the investigators to discuss the volunteer's medical history with their General Practitioner and access all medical records when relevant to study procedures.
- For females only, willingness to practice continuous effective contraception (see below) during the study and a negative pregnancy test on the day(s) of screening and vaccination.
- Agreement to refrain from blood donation during the course of the study.
- Provide written informed consent.

6.3.2 Exclusion Criteria

The volunteer may not enter the study if any of the following apply:

- Prior receipt of any vaccines (licensed or investigational) ≤30 days before enrolment

- Planned receipt of any vaccine other than the study intervention within 30 days before and after each study vaccination with the exception of the licensed seasonal influenza vaccination and the licensed pneumococcal vaccine. Participants will be encouraged to receive these vaccinations at least 7 days before or after their study vaccine.
- Prior receipt of an investigational or licensed vaccine likely to impact on interpretation of the trial data (e.g. Adenovirus vectored vaccines, any coronavirus vaccines)
- Administration of immunoglobulins and/or any blood products within the three months preceding the planned administration of the vaccine candidate.
- Any confirmed or suspected immunosuppressive or immunodeficient state, including HIV infection; asplenia; recurrent severe infections and use of immunosuppressant medication within the past 6 months, except topical steroids or short-term oral steroids (course lasting <14 days) .
- Any autoimmune conditions, except mild psoriasis, well-controlled autoimmune thyroid disease, vitiligo or stable coeliac disease not requiring immunosuppressive or immunomodulatory therapy.
- History of allergic disease or reactions likely to be exacerbated by any component of the ChAdOx1 nCoV-19 or MenACWY vaccines.
- Any history of angioedema.
- Any history of anaphylaxis.
- Pregnancy, lactation or willingness/intention to become pregnant during the study.
- History of cancer (except basal cell carcinoma of the skin and cervical carcinoma in situ).
- History of serious psychiatric condition likely to affect participation in the study (e.g. ongoing severe depression, history of admission to an in-patient psychiatric facility, recent suicidal ideation, history of suicide attempt, bipolar disorder, personality disorder, alcohol and drug dependency, severe eating disorder, psychosis, use of mood stabilisers or antipsychotic medication).
- Bleeding disorder (e.g. factor deficiency, coagulopathy or platelet disorder), or prior history of significant bleeding or bruising following IM injections or venepuncture.
- Any other serious chronic illness requiring hospital specialist supervision.
- Chronic respiratory diseases, including mild asthma (resolved childhood asthma is allowed)
- Chronic cardiovascular disease (including hypertension), gastrointestinal disease, liver disease (except Gilberts Syndrome), renal disease, endocrine disorder (including diabetes) and neurological illness (excluding migraine)

- Seriously overweight (BMI≥40 Kg/m^2) or underweight (BMI≤18 Kg/m^2)
- Suspected or known current alcohol abuse as defined by an alcohol intake of greater than 42 units every week.
- Suspected or known injecting drug abuse in the 5 years preceding enrolment.
- Any clinically significant abnormal finding on screening biochemistry, haematology blood tests or urinalysis.
- Any other significant disease, disorder or finding which may significantly increase the risk to the volunteer because of participation in the study, affect the ability of the volunteer to participate in the study or impair interpretation of the study data.
- History of laboratory confirmed COVID-19.
- New onset of fever or a cough or shortness of breath or anosmia/ageusia since February 2020. Should a reliable test become available, this exclusion criteria will be replaced with seropositivity for SARS-CoV-2 before enrolment.
- Those who have been at high risk of exposure before enrolment, including but not limited to: close contacts of confirmed COVID-19 cases, anyone who had to self-isolate as a result of a symptomatic household member, frontline healthcare professionals working in A&E, ICU and other higher risk areas. Should a reliable test become available, this exclusion criteria will be replaced with seropositivity for SARS-CoV-2 before enrolment.
- Living in the same household as any vulnerable groups at risk of severe COVID-19 disease (as per PHE guidance)

Additional exclusion criteria (subset of participants receiving Paracetamol in group 4 only)

- History of allergic disease or reactions likely to be exacerbated by Paracetamol

6.3.3 Re-vaccination exclusion criteria

The following AEs associated with any vaccine, or identified on or before the day of vaccination constitute absolute contraindications to further administration of an IMP to the volunteer in question. If any of these events occur during the study, the subject will not be eligible to receive a booster dose and will be followed up by the clinical team or their GP until resolution or stabilisation of the event:

- Anaphylactic reaction following administration of vaccine
- Pregnancy. An exception to this will be prior to receipt of a booster dose at extra visit B. If a pregnant woman has discussed vaccination with their usual clinician (e.g. GP) and chooses to receive a COVID-

19 vaccination, this may be administered by the trial team as part of extra visit B (Protocol V17.0 or as part of the provision of treatment to controls.

- Any AE that in the opinion of the Investigator may affect the safety of the participant or the interpretation of the study results

Participants who develop COVID-19 symptoms and have a positive NAAT test after the first vaccination can only receive a booster dose after a minimum 4 weeks interval from their first NAAT positive test, provided their symptoms have significantly improved. The decision to proceed with booster vaccinations in those cases will be at clinical discretion of the investigators. For participants who are asymptomatic and have a positive NAAT test, a minimum of 2 weeks from first NAAT positivity will be required before boosting.

6.3.4 Effective contraception for female volunteers

Female volunteers of childbearing potential are required to use an effective form of contraception at least during the first 3 months after their booster vaccination (groups 2-5) and the first 3 months after their single dose vaccine administration (group 1).

Acceptable forms of contraception for female volunteers include:

- Established use of oral, injected or implanted hormonal methods of contraception.
- Placement of an intrauterine device (IUD) or intrauterine system (IUS).
- Total abdominal hysterectomy.
- Bilateral tubal Occlusion
- Barrier methods of contraception (condom or occlusive cap with spermicide).
- Male sterilisation, if the vasectomised partner is the sole partner for the subject.
- True abstinence, when this is in line with the preferred and usual lifestyle of the subject. Periodic abstinence (e.g., calendar, ovulation, symptothermal, post-ovulation methods), declaration of abstinence for the duration of exposure to IMP, and withdrawal are not acceptable methods of contraception

6.3.4.1 Contraception for female volunteers following unblinding and provision of ChAdOx1 nCoV-19 vaccines (Protocol V17.0)

In keeping with UK national guidance and the product approvals, female participants will be advised that they do not need to continue to use effective contraception methods if they are receiving ChAdOx1 nCoV-19 vaccinations as part of either:

- The national vaccine rollout

- Provision of a second dose to single dose participants within the trial
- Provision of treatment to controls within the trial (Protocol V17.0)

6.3.5 Prevention of 'Over Volunteering'

Volunteers will be excluded from the study if they are concurrently involved in another trial where an IMP has been administered within 30 days prior to enrolment, or will be administered during the trial period. In order to ensure this, volunteers will be asked to provide their National Insurance or Passport number (if they are not entitled to a NI number) and will be registered on a national database of participants in clinical trials (*www.tops.org.uk*). They will not be enrolled if found to be actively registered on another trial until further information on IMP and bleeding schedule is obtained.

6.3.6 Withdrawal of Volunteers

In accordance with the principles of the current revision of the Declaration of Helsinki and any other applicable regulations, a volunteer has the right to withdraw from the study at any time and for any reason, and is not obliged to give his or her reasons for doing so. The Investigator may withdraw the volunteer at any time in the interests of the volunteer's health and well-being. In addition, the volunteer may withdraw/be withdrawn for any of the following reasons:

- Administrative decision by the Investigator.
- Ineligibility (either arising during the study or retrospectively, having been overlooked at screening).
- Significant protocol deviation.
- Volunteer non-compliance with study requirements.
- An AE, which requires discontinuation of the study involvement or results in inability to continue to comply with study procedures.

The reason for withdrawal will be recorded in the CRF. If withdrawal is due to an AE, appropriate follow-up visits or medical care will be arranged, with the agreement of the volunteer, until the AE has resolved, stabilised or a non-trial related causality has been assigned. The DSMB or DSMB chair may recommend withdrawal of volunteers.

Any volunteer who is withdrawn from the study may be replaced, if that is possible within the specified time frame.

If a volunteer withdraws from the study, data and blood samples collected before their withdrawal will still be used on the analysis. Storage of blood samples will continue unless the participant specifically requests otherwise.

In all cases of subject withdrawal, long-term safety data collection, including some procedures such as safety bloods, will continue as appropriate if subjects have received one or more vaccine doses, unless they decline any further follow-up.

Vaccination with an approved or licensed COVID-19 vaccine according to government policy will not be considered a withdrawal from the study. Participants who are offered and accept a COVID-19 vaccine will be unblinded to the vaccines they have received prior to receiving the approved COVID-19 vaccine and will be asked to continue in the study.

6.3.6.1 Discontinuation from Study Procedures

If a participant wishes to withdraw from the study because of the intensity of study visits or procedures and the commitment this requires, but are willing to remain in the study, they will be offered the option to discontinue some study procedures (e.g. booster dose in 2-dose groups, blood samples, vaccine diary, symptomatic pathway, e-diary, in-person symptomatic follow-up). This allows continued data collection on core study data for safety, immunogenicity and efficacy outcomes.

6.4 Pregnancy

Should a volunteer become pregnant during the trial, no further study IMP will be administered* (see below for exception). She will be followed up for clinical safety assessment with her ongoing consent and in addition will be followed until pregnancy outcome is determined. We would not routinely perform venepuncture in a pregnant volunteer unless there is clinical need. Given that no routine bloods will be drawn from pregnant volunteers, follow-up visits may be conducted over the phone/video consultation in order to minimise contact and exposure from SARS-CoV-2 in pregnant volunteers.

** The current UK national vaccination policy advice is that pregnant women may receive the vaccine as part of the national rollout of COVID-19 vaccines in certain circumstances[20]. The decision on whether COVID-19 vaccination should be recommended for any pregnant participants in the trial will not be taken by trial teams. However, if after discussion of the risk and benefits of COVID-19 vaccination with their usual clinician (e.g. GP), a pregnant participant chooses to have a COVID-19 vaccination during pregnancy, this may be administered by the trial team as part of extra visit B, although no blood samples will be drawn.*

7 TRIAL PROCEDURES

This section describes the trial procedures for evaluating study participants and follow-up after administration of study vaccine.

7.1 Schedule of Attendance

All volunteers in groups 1 will have the same schedule of clinic attendances and procedures as indicated in the schedules of attendance (Table 6). Group 2 will have clinic attendances and procedures as indicated in the schedules of attendances below (Table 7). Group 3 will have clinic attendances and procedures as indicated in the schedules of attendances below (Table 8). Group 4 will have clinic attendances and procedures as indicated in the schedules of attendances below (Table 9). Subjects will receive either the ChAdOx1 nCoV-19 vaccine or MenACWY control, and undergo follow-up for a total of 12 months from the last vaccination visit. The total volume of blood donated during the study will be 177.5 – 621.5mL depending on which group they are allocated to. Additional visits or procedures may be performed at the discretion of the investigators, e.g., further medical history and physical examination, urine microscopy in the event of positive urinalysis or additional blood tests if clinically relevant.

7.2 Observations

Pulse, blood pressure and temperature will be measured at the time-points indicated in the schedule of procedures and may also be measured as part of a physical examination if indicated at other time-points.

7.3 Blood tests, Nose/Throat Swab and urinalysis

Blood will be drawn for the following laboratory tests and processed at agreed NHS Trust laboratories using NHS standard procedures:

- **Haematology;** Full Blood Count
- **Biochemistry;** Sodium, Potassium, Urea, Creatinine, Albumin, Liver Function Tests (ALT, ALP, Bilirubin)
- **Diagnostic serology;** HBsAg, HCV antibodies, HIV antibodies (specific consent will be gained prior to testing blood for these blood-borne viruses)
- **Immunology;** Human Leukocyte Antigen (HLA) typing (groups 1 and 3 only)

A nose/throat swab will be conducted for COVID-19 NAAT

- **COVID-19** NAAT processing (nose/throat swabs)

Additional safety blood tests may be performed if clinically relevant at the discretion of the medically qualified investigators, including potential prognostic indicators or markers of severe COVID-19 disease.

At University of Oxford research laboratories:

- **Immunology**; Immunogenicity will be assessed by a variety of immunological assays. This may include antibodies to SARS-CoV-Spike and non-Spike antigens by ELISA, ex vivo ELISpot assays for interferon gamma and flow cytometry assays, neutralising and other functional antibody assays and B cell analyses. Other exploratory immunological assays including cytokine analysis and other antibody assays, DNA analysis of genetic polymorphisms potentially relevant to vaccine immunogenicity and gene expression studies amongst others may be performed at the discretion of the Investigators. SARS-CoV-2 serology to be conducted at screening on participants at high risk of COVID-19 exposure (healthcare workers will be prioritised), subject to test availability and lab capacity.
- **Urinalysis;** Urine will be tested for protein, blood and glucose at screening. For female volunteers only, urine will be tested for beta-human chorionic gonadotrophin (β-HCG) at screening and immediately prior to vaccination.
- **Stool samples**; SARS-CoV-2 NAAT, infectivity assays, calprotectin, and other exploratory immunology and microbiology assays may be conducted in a subset of participants, subject to site capacity, sample and test availability

Collaboration with other specialist laboratories in the UK, Europe and outside of Europe for further exploratory tests may occur. This would involve the transfer of serum, urine or plasma, PBMC and/or other study samples to these laboratories, but these would remain anonymised. Informed consent for this will be gained from volunteers. Samples collected for the purposes of COVID-19 diagnosis might be sent to reference labs in the UK alongside their personal data. This would be in line with the national guidance and policy for submitting samples for testing at reference labs.

Immunological assays will be conducted according to local SOPs.

Subjects will be informed that there may be leftover samples of their blood (after all testing for this study is completed), and that such samples may be stored indefinitely for possible future research (exploratory immunology), including genotypic testing of genetic polymorphisms potentially relevant to vaccine immunogenicity. Subjects will be able to decide if they will permit such future use of any leftover samples. With the volunteers' informed consent, any leftover cells, urine and serum/plasma will be frozen indefinitely for future analysis of COVID-19 and other coronaviruses related diseases or vaccine-related responses. If a subject elects not to permit this, all of that subject's leftover samples will be discarded after the required period of storage to meet Good Clinical Practice (GCP) and regulatory requirements.

Samples that are to be stored for future research will be transferred to the OVC Biobank (REC 16/SC/0141).

7.4 Study visits

The study visits and procedures will be undertaken by one of the clinical trials team. The procedures to be included in each visit are documented in the schedule of attendances (Tables6-9). Each visit is assigned a time-point and a window period, within which the visit will be conducted. In order to obtain core study data if a participant is unable to attend a study visit, the visit will be conducted by phone or video consultation and information recorded in the CRF.

7.4.1 Screening visit

Participants will be required to complete an online questionnaire as an initial confirmation of eligibility. All potential volunteers will have a screening visit, which may take place up to 90 days prior to vaccination. At the screening visit, a video presentation of the aims of the study and all tests to be carried out may be screened to an audience. Individually each volunteer will have the opportunity to question an appropriately trained and delegated researcher before signing the consent. Informed consent will be taken before screening, as described in section 6.2. If consent is obtained, the procedures indicated in the schedule of attendances will be undertaken including a medical history, physical examination, blood tests and height and weight. To avoid unnecessary additional venepuncture, if the appropriate blood test results for screening are available for the same volunteer from a screening visit for another study, these results may be used for assessing eligibility (provided the results date is within the 6 months preceding enrolment in COV001).

We will aim to contact the subject's general practitioner with the written permission of the subject after screening to corroborate medical history when possible and practical to do so. GPs will be notified that the subject has volunteered for the study. During the screening, the volunteers will be asked to provide their National Insurance or passport number so that this can be entered on to a national database which helps prevent volunteers from participating in more than one clinical trial simultaneously or over-volunteering for clinical trials (www.tops.org.uk).

Abnormal clinical findings from the urinalysis or blood tests at screening will be assessed by a medically qualified study member. Abnormal blood tests following screening will be assessed according to specific laboratory adverse event grading tables. Any abnormal test result deemed clinically significant may be repeated to ensure it is not a single occurrence. If an abnormal finding is deemed to be clinically significant, the volunteer will be informed and appropriate medical care arranged with the permission of the volunteer.

The eligibility of the volunteer will be reviewed at the end of the screening visit and again when all results from the screening visit have been considered. Decisions to exclude the volunteer from enrolling in the trial or to withdraw a volunteer from the trial will be at the discretion of the Investigator. If eligible, a day 0 visit will be scheduled for the volunteer to receive the vaccine and subsequent follow-up.

7.4.2 Day 0: Enrolment and vaccination visit

Volunteers will be considered enrolled in to the trial at the point of vaccination. Before vaccination/trial intervention, the eligibility of the volunteer will be reviewed. Pulse, blood pressure and temperature will be observed and if necessary, a medical history and physical examination may be undertaken to determine need to withdraw the participant. Participants with symptoms meeting the case definition for COVID-19 or likely recent exposure to COVID-19 will be excluded. Vaccinations will be administered as described below.

7.4.2.1 Vaccination

All vaccines will be administered intramuscularly according to specific SOPs. The injection site will be covered with a sterile dressing and the volunteer will stay in the trial site for observation, in case of immediate adverse events. Observations will be taken 60 minutes after vaccination (+/- 30 minutes) for the prime dose and from 15 minutes after booster vaccination. The sterile dressing will be removed and injection site inspected.

In all groups, volunteers will be given an oral thermometer, tape measure and diary card (paper or electronic), with instructions on use, along with the emergency 24 hour telephone number to contact the on-call study physician if needed. Volunteers will be instructed on how to self-assess the severity of these AEs. There will also be space on the diary card to self-document unsolicited AEs, and whether medication was taken to relieve the symptoms. Volunteers in groups 2f, 2g, 4c and 4d will not be asked to fill-out diary cards for their booster vaccines. All participants in groups 1c, 1d, 5a and 5b will be asked to complete (paper or electronic) diaries following their late boost vaccination visit. Diary cards will collect information on the timing and severity of the following solicited AEs:

Table 4. ***Solicited AEs as collected on post vaccination diary cards***

Local solicited AEs	Systemic solicited AEs
Pain	Fever
Tenderness	Feverishness
Redness	Chills
Warmth	Joint pains
Itch	Muscle pains
Swelling	Fatigue
Induration	Headache
	Malaise
	Nausea
	Vomiting

7.4.2.2 Sequence of Enrolment and Vaccination of Volunteers

Prior to initiation of the study, any newly available safety data will be reviewed from animal studies or clinical trials of coronavirus vaccines being tested elsewhere, and discussed with the DSMB and/or MHRA as necessary. For safety reasons, the first volunteer to receive the IMP will be vaccinated ahead of any other participants and the profile of adverse events will be reviewed after 24 hours (+24h) post vaccination. Provided there are no safety concerns, as assessed by a medically qualified investigator and/or chair of DSMB, another 3 volunteers will be vaccinated with the IMP after at least 48 hours (±24h) has elapsed following first vaccination and at least 1 hour apart from each other. The profile of AEs will be assessed by a medically qualified investigator in real time and after 24 hours (+24h) of the first 4 participants receiving the IMP, further vaccinations will proceed provided there are no safety concerns. Relevant investigators and chair of DSMB will be asked to provide a decision on whether further vaccinations can go ahead after the first 4 participants received the IMP. A full DSMB may also be consulted should safety concerns arise at this point.

A review will be conducted based on accumulated safety data of the first up to 54 participants receiving the IMP. Enrolment of the remaining participants will only proceed if the CI, and/or other designated relevant investigators and the chair of DSMB assess the data as indicating that it is safe to do so. At this point, any new immunopathology data from pre-clinical challenge studies in ferrets and non-human primates will be assessed by the CI and/or other designated relevant investigators and the DSMB prior to enrolment of the remaining participants.

A second review will be conducted based on accumulated safety data once the trial is fully recruited. The table below provides an estimate of the sequence of recruitment

7.4.3 Subsequent visits:

Follow-up visits will take place as per the schedule of attendances described in tables 6-9 with their respective windows. Volunteers will be assessed for local and systemic adverse events, interim history, physical examination, review of diary cards (paper or electronic) and blood tests at these time points as detailed in the schedule of attendances. Blood will also be taken for immunology purposes.

If volunteers experience adverse events (laboratory or clinical), which the investigator (physician), CI and/or DSMB chair determine necessary for further close observation, the volunteer may be admitted to an NHS hospital for observation and further medical management under the care of the Consultant on call.

Table 5. Expected recruitment schedule

By Day	0	3	5	6 onwards
Single Dose IMP arms (up to)	1	3	40	496
Control arms (up to)	1	3	40	496
Prime-Boost Group (up to)			10	
Total per Day<br>(up to)	2	6	90	Approximately 120 per day until trial fully recruited
Cumulative IMP	1	4	54	Up to 540
Cumulative<br>Total	2	8	98	Up to 1090
Safety Review	Real time<br>Review of pre-clinical data	Real time	Real time<br>Review of first 54 participants receiving IMP before enrolling the remainder	Review of Immunopathology data (pre-clinical studies)<br>Review of accumulated safety data once trial fully recruited

Table 6 Schedule of attendances for participants in group 1a,b. (prime vaccine only) Note: Participants in group 1a and b may be reallocated 10 months post-enrolment to subgroups 1c or 1d respectively (see table 12 and 13) . Protocol V17: Unblinding / provision of treatment procedures will occur concurrently (Table 14) to these procedures.

Attendance Number	1^{S}	2	3	4	5	6	7	8	9	10	COVID-19 Testing	COVD-19 NAAT positive + 7 days (remote)	COVID-19 Follow-up	Extra Visit A	Extra Visit B
														Optional	Optional
Timeline** (days)	≤ 90	0	1	3	7	14	28	56	182	364	As required	7 days post NAAT positive	As required	At time of early unblinding before receipt of deployed vaccine	At earliest possibility
Time window (days)				±1	±2	±3	±7	±7	±14	±30	N/A	-2, + 7	N/A	N/A	≥4 weeks post prime
Informed Consent	X														
Review contraindications, inclusion and exclusion criteria	X	X													
Vaccination		X													X
Vital signs^	X	X		X	X	X	X	X	X	X	X				
Telephone/Video call			X									X	As required		

Attendance Number	1^{S}	2	3	4	5	6	7	8	9	10	COVID-19 Testing	COVD-19 NAAT positive + 7 days (remote)	COVID-19 Follow-up	Extra Visit A	Extra Visit B
														Optional	Optional
Ascertainment of adverse events		X	X	X	X	X	X	X	X	X	X	X	X	X	X
Diary cards provided		X													
Diary cards collected							X								
Weekly household exposure questionnaire															
Medical History, Physical Examination	X	(X)		(X)	(X)	(X)	(X)	(X)	(X)	(X)	(X)	(X)			
Biochemistry, Haematology (mL)	5	5		5	5		5								
Exploratory immunology (mL)	(5)*	50			50	50	50	50	50	50	up to 50			up to 50	up to 50
PAXgenes (mL)		2.5									2.5				
Nose/Throat Swab											X				
Stool sample [a,b]												(X)	(X)		
Urinalysis	X														
Urinary bHCG (women only)	X	X													

Attendance Number	1S	2	3	4	5	6	7	8	9	10	COVID-19 Testing	COVD-19 NAAT positive + 7 days (remote)	COVID-19 Follow-up	Extra Visit A	Extra Visit B
														Optional	Optional
HLA typing (mL)		4													
HBsAg, HCV Ab, HIV serology (mL)	5														
Blood volume per visit	15	61.5		5	55	50	55	50	50	50	up to 52.5			up to 50	up to 50
Cumulative blood volume%*	15	76.5		81.5	136.5	186.5	241.5	291.5	341.5	391.5	444			494	494

S = screening visit; (X) = if considered necessary ^ = Vital signs includes pulse, blood pressure and temperature; ** Timeline is approximate only. Exact timings of visits relate to the day on enrolment, ie, each visit must occur at indicated number of days after enrolment ± time window. % Cumulative blood volume for volunteers if blood taken as per schedule, and excluding any repeat safety blood test that may be necessary. Blood volumes may vary according to local site equipment and practices. *SARS-CoV-2 serology to be conducted at screening on participants at high risk of COVID-19 exposure (healthcare workers will be prioritised), subject to test availability and lab capacity. An extra 5mls should be added to cumulative blood volumes if extra COVID-19 serology is required at screening. [a] Subject to site capacity, sample and test availability. [b] Optional Stool sample at approximately 7 days after onset of symptoms for those who have a positive SARS-CoV-2 NAAT test result and possibly again at 14 days after symptom onset if necessary.

Table 7 Schedule of attendances for participants in group 2a and 2b. Protocol V17: Unblinding / provision of treatment procedures will occur concurrently (Table 14) to these procedures.

Attendance Number	1[S]	2	3	4	5	COVID-19 Testing	COVD-19 NAAT positive + 7 days (remote)	COVID-19 follow-up	Extra Visit A	Extra Visit B
									Optional	Optional
Timeline** (days)	≤ 90	0	28	182	364	As required	7 days post NAAT positive	As required	At time of early unblinding before receipt of deployed vaccine	At earliest possibility
Time window (days)			±7	±14	±30	N/A	-2, +7	N/A	N/A	≥4 weeks post prime
Informed Consent	X									
Review contraindications, inclusion and exclusion criteria	X	X								
Vaccination		X								
Vital signs^	X	X	X	X	X	X				
Telephone/Video call							X	As required		
Ascertainment of adverse events		X	X	X	X	X	X	X	X	X
Diary cards provided		X								
Diary cards collected			X							
Weekly household exposure questionnaire	Ongoing									

Attendance Number	1[S]	2	3	4	5	COVID-19 Testing	COVD-19 NAAT positive + 7 days (remote)	COVID-19 follow-up	Extra Visit A	Extra Visit B
									Optional	Optional
Medical History, Physical Examination	X	(X)	(X)	(X)	(X)	(X)	(X)			
Biochemistry, Haematology (mL)	5	5	5							
Exploratory immunology (mL)	(5)*	50	50	50	50	up to 50			up to 50	Up to 50
PAXgenes (mL)		2.5				2.5				
Nose/Throat Swab						X				
Stool sample [a,b]							(X)	(X)		
Urinalysis	X									
Urinary bHCG (women only)	X	X								
HBsAg, HCV Ab, HIV serology (mL)	5									
Blood volume per visit	15	57.5	55	50	50	up to 57.5			up to 50	up to 50
Cumulative blood volume[%]	15	72.5	127.5	177.5	227.5	280	280		330	380

S = screening visit; (X) = if considered necessary ^ = Vital signs includes pulse, blood pressure and temperature; ** Timeline is approximate only. Exact timings of visits relate to the day on enrolment, ie, each visit must occur at indicated number of days after enrolment ± time window.% Cumulative blood volume for volunteers if blood taken as per schedule, and excluding any repeat safety blood test that may be necessary. Blood volumes may vary according to local site equipment and practices. *SARS-CoV-2 serology to be conducted at screening on participants at high risk of COVID-19 exposure (healthcare workers will be prioritised), subject to test availability and lab capacity. An extra 5mls should be added to cumulative blood volumes if extra COVID-19 serology is required at screening. [a] Subject to site capacity, sample and test availability. [b] Optional Stool sample at approximately 7 days after onset of symptoms for those who have a positive SARS-CoV-2 NAAT test result and possibly again at 14 days after symptom onset if necessary.

Table 8 Schedule of attendances for participants in group 2c, 2d and 2e. Note: Participants in group 2c and 2e may be reallocated approx. 10 months post-enrolment to subgroups 5a or 5b respectively (see table 12 and 13). Protocol V17: Unblinding / provision of treatment procedures will occur concurrently (Table 14) to these procedures.

Attendance Number	1[S]	2	3	4	5	6	7	8	COVID-19 Testing	COVD-19 NAAT positive + 7 days (remote)	COVID-19 follow-up	Extra Visit A
Timeline** (days)	≤ 90	0	28	56	14 days post boost	28 days post boost	182 days post boost	364 days post boost	As required	7 days post NAAT positive	As required	At time of early unblinding before receipt of deployed vaccine
Time window (days)			±7	-7/+14	±7	±7	±14	±30	N/A	-2, +7	N/A	N/A
Informed Consent	X											
Review contraindications, inclusion and exclusion criteria	X	X										
Vaccination		X		X								
Vital signs^	X	X	X				X	X	X			
Telephone/Video call										X	As required	
Ascertainment of adverse events		X	X				X	X	X	X	X	X
Diary cards provided		X		X								
Diary cards collected			X			X						
Weekly household exposure questionnaire	Ongoing											
Medical History, Physical Examination	X	(X)	(X)				(X)	(X)	(X)	(X)		
Biochemistry, Haematology (mL)	5	5	5	5	5	5						

Attendance Number	1[S]	2	3	4	5	6	7	8	COVID-19 Testing	COVD-19 NAAT positive + 7 days (remote)	COVID-19 follow-up	Extra Visit A
Exploratory immunology (mL)	(5)*	50	50	Up to 50	Up to 50	Up to 50	Up to 50	Up to 50	up to 50			up to 50
PAXgenes (mL)		2.5							2.5			
Nose/Throat Swab									X			
Stool sample [a,b]										(X)	(X)	
Urinalysis	X											
Urinary bHCG (women only)	X	X		X								
HBsAg, HCV Ab, HIV serology (mL)	5											
Blood volume per visit	15	57.5	55	55	55	55	50	50	up to 52.5			up to 50
Cumulative blood volume[%]	15	72.5	127.5	182.5	237.5	292.5	342.5	392.5	445	445		495

S = screening visit; (X) = if considered necessary ^ = Vital signs includes pulse, blood pressure and temperature; ** Timeline is approximate only. Exact timings of visits relate to the day on enrolment, ie, each visit must occur at indicated number of days after enrolment ± time window.% Cumulative blood volume for volunteers if blood taken as per schedule, and excluding any repeat safety blood test that may be necessary. Blood volumes may vary according to local site equipment and practices. *SARS-CoV-2 serology to be conducted at screening on participants at high risk of COVID-19 exposure (healthcare workers will be prioritised), subject to test availability and lab capacity. An extra 5mls should be added to cumulative blood volumes if extra COVID-19 serology is required at screening. [a] Subject to site capacity, sample and test availability. [b] Optional Stool sample at approximately 7 days after onset of symptoms for those who have a positive SARS-CoV-2 NAAT test result and possibly again at 14 days after symptom onset if necessary.

Table 9 Schedule of attendances for participants in group 3. Protocol V17: Unblinding / provision of treatment procedures will occur concurrently (Table 14) to these procedures.

Attendance Number	1[S]	2(V1)	3	4	5	6	7 (V2)	8	9	10	11	12	13	COVID-19 Testing	COVD-19 NAAT positive + 7 days (remote)	COVID-19 Follow-up
Timeline** (days)	≤ 90	0	1	3	7	14	28	31	35	42	56	182	364	As required	7 days post NAAT positive	As required
Time window (days)			+1	±1	±3	±3	±7	±1	±2	±3	±3	±14	±30	N/A	-2, +7	N/A
Informed Consent	X															
Review contraindications, inclusion and exclusion criteria	X	X					X									
Vaccination		X					X									
Vital signs^	X	X		X	X	X	X	X	X	X	X	X	X	X		
Telephone/Video call			X												X	As required
Ascertainment of adverse events		X	X	X	X	X	X	X	X	X	X	X	X	X	X	
Diary cards provided		X					X									
Diary cards collected							X				X					
Weekly household	Ongoing															

exposure questionnaire																
Medical History, Physical Examination	X	(X)		(X)	(X)	(X)	(X)	(X)	(X)	(X)	(X)	(X)	(X)	(X)	(X)	
Biochemistry[$], Haematology (mL)	5	5		5	5		5	5	5		5					
Exploratory immunology[£] (mL)	(5)*	50			50	50	50		50	50	50	50	50	up to 50		
PAXgenes (mL)		2.5												2.5		
Nasal/Throat Swab														X		
Stool sample [a,b]															(X)	(X)
Urinalysis	X															
Urinary bHCG (women only)	X	X					X									
HLA typing (mL)		4														
HBsAg, HCV Ab, HIV serology (mL)	5															
Blood volume per visit	15	61.5		5	55	50	55	5	55	50	55	50	50	up to 52.5		
Cumulative blood volume[%]	15	76.5		81.5	136.5	186.5	241.5	246.5	301.5	351.5	406.5	456.5	506.5	559	559	

S = screening visit; (X) = if considered necessary ^ = Vital signs includes pulse, blood pressure and temperature; ** Timeline is approximate only. Exact timings of visits relate to the day on enrolment, ie, each visit must occur at indicated number of days after enrolment ± time window.% Cumulative blood volume for volunteers if blood taken as per schedule, and excluding any repeat safety blood test that may be necessary. Blood volumes may vary according to local site equipment and practices. *SARS-CoV-2 serology to be conducted at screening on participants at high risk of COVID-19 exposure (healthcare workers will be prioritised), subject to test availability and lab capacity. An extra 5mls should be added to cumulative blood volumes if extra COVID-19 serology is required at screening. [a] Subject to site capacity, sample and test availability. [b] Optional Stool sample at approximately 7 days after onset of symptoms for those who have a positive SARS-CoV-2 NAAT test result and possibly again at 14 days after symptom onset if necessary.

Table 10 schedule of attendances for participants in group 4a,b (prime vaccine only). Protocol V17: Unblinding / provision of treatment procedures will occur concurrently (Table 14) to these procedures.

Attendance Number	1[S]	2	3	4	5	COVID-19 Testing	COVD-19 Positive NAAT + 7 days (remote)	COVID-19 Follow-up	Extra Visit A	Extra Visit B
									Optional	Optional
Timeline** (days)	≤ 90	0	28	182	364	As required	7 days post NAAT postive	As required	At time of early unblinding before receipt of deployed vaccine	At earliest possibility
Time window (days)			±7	±14	±30	N/A	-2, +7	N/A	N/A	≥4 weeks post prime
Informed Consent	X									
Review contraindications, inclusion and exclusion criteria	X	X								
Vaccination		X								
Prophylactic Paracetamol for 24h[p]		X								
Vital signs^	X	X	X	X	X	X				
Telephone/Video call							X	As required		
Ascertainment of adverse events		X	X	X	X	X	X	X	X	X
Diary cards provided		X								
Diary cards collected			X							
Weekly household exposure questionnaire	Ongoing									

Attendance Number	1[S]	2	3	4	5	COVID-19 Testing	COVD-19 Positive NAAT + 7 days (remote)	COVID-19 Follow-up	Extra Visit A	Extra Visit B
									Optional	Optional
Medical History, Physical Examination	X	(X)	(X)	(X)	(X)	(X)	(X)			
Biochemistry, Haematology (mL)	5	5	5							
Exploratory immunology (mL)	(5)*	10	10	10	10	up to 50			up to 50	up to 50
PAXgenes (mL)		2.5				2.5				
Nose/Throat Swab						X				
Stool sample [a,b]							(X)	(X)		
Urinalysis	X									
Urinary bHCG (women only)	X	X								
HBsAg, HCV Ab, HIV serology (mL)	5									
Blood volume per visit	15	17.5	15	10	10	up to 52.5			up to 50	up to 50
Cumulative blood volume[%]	15	32.5	47.5	57.5	67.5	120	120		170	170

S = screening visit; (X) = if considered necessary ^ = Vital signs includes pulse, blood pressure and temperature; ** Timeline is approximate only. Exact timings of visits relate to the day on enrolment, ie, each visit must occur at indicated number of days after enrolment ± time window.% Cumulative blood volume for volunteers if blood taken as per schedule, and excluding any repeat safety blood test that may be necessary. Blood volumes may vary according to local site equipment and practices. *SARS-CoV-2 serology to be conducted at screening on participants at high risk of COVID-19 exposure (healthcare workers will be prioritised), subject to test availability and lab capacity. An extra 5mls should be added to cumulative blood volumes if extra COVID-19 serology is required at screening.P = prophylactic paracetamol over the first 24h post immunisation in a subset of participants in group 4 only. [a] Subject to site capacity, sample and test availability. [b] Optional Stool sample at approximately 7 days after onset of symptoms for those who have a positive SARS-CoV-2 NAAT test result and possibly again at 14 days after symptom onset if necessary.

Table 11 Schedule of attendances for participants in group 2f, 2g, 4c and 4d (booster) Note: Participants in group 2f,4c and 2g,4d may be reallocated approx. 10 months post-enrolment to subgroups 5a or 5b respectively (see table 12 and 13). Protocol V17: Unblinding / provision of treatment procedures will occur concurrently (Table 14) to these procedures.

Attendance Number (boost)	1 V2 (booster)	2	3	4	8	COVID-19 Testing	COVD-19 Positive NAAT + 7 days (remote)	COVID-19 Follow-up	Extra Visit A
Timeline** (days)	Minimum 4weeks post prime	28 days post boost	90 days post boost	182 days post boost	364 days post boost	As required	7 days post positive NAAT result		At time of early unblinding before receipt of deployed vaccine
Time window (days)	+14	±7	±14	±14	±30	N/A	-2, +7		N/A
Informed Consent	X								
Review contraindications, inclusion and exclusion criteria	X								
Vaccination	X								
Prophylactic Paracetamol for 24h[p]	X								
Vital signs^	(X)	(X)	(X)	(X)	(X)	X			
Telephone/Video call							X	As required	
Ascertainment of adverse events	X	X	X	X	X	X	X	X	X
Weekly household exposure questionnaire	Ongoing								

Attendance Number (boost)	1 V2 (booster)	2	3	4	8	COVID-19 Testing	COVD-19 Positive NAAT + 7 days (remote)	COVID-19 Follow-up	Extra Visit A
Medical History	(X)	(X)	(X)	(X)	(X)	(X)	(X)		
Physical Examination (if necessary)	(X)	(X)	(X)	(X)	(X)	(X)	(X)		
Biochemistry, Haematology (mL)									
Exploratory immunology (mL)	up to 50	up to 50	up to 50	up to 50	up to 50	up to 50[d]			up to 50
PAXgenes (mL)						2.5			
Nose/Throat Swab						X			X
Stool sample [a,b]							(X)	(X)	
Urinary bHCG (women of childbearing potential only)	X								
Blood volume per visit	50	50	50	50	50	up to 52.5			up to 50
Cumulative blood volume[%]	50	100	150	200	250	302.5	302.5		352.5

S = screening visit; (X) = if considered necessary; ** Timeline is approximate only. Exact timings of visits relate to the day on enrolment, ie, each visit must occur at indicated number of days after enrolment ± time window. ^Only temperature will be routinely measured at subsequent follow-up visits. At COVID-19 testing visits a full set observations will be taken, including respiratory rate and oxygen saturation. % Cumulative blood volume for volunteers if blood taken as per schedule, and excluding any repeat safety blood test that may be necessary. Blood volumes may vary according to local site equipment and practices. [a] Subject to site capacity, sample and test availability. [d] Optional and subject to site capacity. [b] Optional Stool sample at approximately 7 days after onset of symptoms for those who have a positive SARS-CoV-2 NAAT test result and possibly again at 14 days after symptom onset if necessary. [P] = prophylactic paracetamol over the first 24h post immunisation

Table 12 Schedule of attendances for participants in group 1c and 3b and 5a

Attendance Number (boost)	1 Late Vacc (LV)	LV8 (remote)	2 (LV14)	3 (LV28)	5 (LV182)	COVID-19 Testing	COVD-19 Positive NAAT + 7 days (remote)	COVID-19 Follow-up
Timeline** (days)	10 months post prime	8 days post LV	14 days post LV	28 days post LV	182 days post LV	As required	7 days post positive NAAT result	
Time window (days)	±56 days	+7	±7	±14	±14	N/A	-2, +7	
Informed Consent	X							
Review contraindications, inclusion and exclusion criteria	X							
ChAdOx1 nCov-19 Vaccination	X							
Vital signs^	(X)		(X)	(X)	(X)	(X)		
Telephone/Video call							X	As required
Ascertainment of adverse events	X		X	X	X	(X)	(X)	X
Diary cards provided (7 days)	X							
Participant unblinding		X						
Diary cards collected			x					
Weekly household exposure questionnaire	ongoing							
Medical History [M]	(X)		(X)	(X)	(X)	(X)	(X)	
Physical Examination (if necessary)	(X)		(X)	(X)	(X)	(X)	(X)	

Attendance Number (boost)	1 Late Vacc (LV)	LV8 (remote)	2 (LV14)	3 (LV28)	5 (LV182)	COVID-19 Testing	COVD-19 Positive NAAT + 7 days (remote)	COVID-19 Follow-up
Exploratory immunology (mL)	up to 50		up to 50	up to 50	up to 50	up to 50[d]		
PAXgenes (mL)						2.5		
Nose/Throat Swab						X		
Stool sample [a,b]							(X)	(X)
Urinary bHCG (women of childbearing potential only)	X							
Blood volume per visit	50		50	50	50	up to 52.5		
Cumulative blood volume[%]	50		100	150	200	252.5	252.5	

S = screening visit; (X) = if considered necessary; ** Timeline is approximate only. Exact timings of visits relate to the day on enrolment, ie, each visit must occur at indicated number of days after enrolment ± time window. ^Only temperature will be routinely measured at subsequent follow-up visits. At COVID-19 testing visits a full set observations will be taken, including respiratory rate and oxygen saturation. % Cumulative blood volume for volunteers if blood taken as per schedule, and excluding any repeat safety blood test that may be necessary. Blood volumes may vary according to local site equipment and practices. [a] Subject to site capacity, sample and test availability. [d] Optional and subject to site capacity. [b] Optional Stool sample at approximately 7 days after onset of symptoms for those who have a positive SARS-CoV-2 NAAT test result and possibly again at 14 days after symptom onset if necessary [M] Females will be questioned about menstrual cycle prior to receiving boost vaccination.

Table 13 Schedule of attendances for participants in group 1d and 5b

Attendance Number (boost)	1 Late Vacc (LV)	LV8 (remote)	2 (LV14)	3 (LV28)	4 Late Vacc Two (LVT)	5 (LV182)	COVID-19 Testing	COVD-19 Positive NAAT + 7 days (remote)	COVID-19 Follow-up
Timeline** (days)	10 months post prime	8 days post LV	14 days post LV	28 days post LV	84 days post LV (Must occur after LV28 visit procedures)	182 days post LV	As required	7 days post positive NAAT result	
Time window (days)	±56 days	+7	±7	±14	-56	±14	N/A	-2, +7	
Informed Consent	X								
Review contraindications, inclusion and exclusion criteria	X								
ChAdOx1 ncov-19 Vaccination	X				X				
Vital signs^	(X)		(X)	(X)		(X)	X		
Telephone/Video call								X	As required
Ascertainment of adverse events	X		X	X	X	X	X	X	X
Diary cards provided (7 days)	X								
Diary cards collected			x						
Participant Unblinding		X							

Attendance Number (boost)	1 Late Vacc (LV)	LV8 (remote)	2 (LV14)	3 (LV28)	4 Late Vacc Two (LVT)	5 (LV182)	COVID-19 Testing	COVD-19 Positive NAAT + 7 days (remote)	COVID-19 Follow-up
Weekly household exposure questionnaire	Ongoing								
Medical History [M]	(X)		(X)	(X)	(X)	(X)	(X)	(X)	
Physical Examination (if necessary)	(X)		(X)	(X)	(X)	(X)	(X)	(X)	
Exploratory immunology (mL)	up to 50		up to 50	up to 50		up to 50	up to 50[d]		
PAXgenes (mL)							2.5		
Nose/Throat Swab							X		
Stool sample [a,b]								(X)	(X)
Urinary bHCG (women of childbearing potential only)	X				X				
Blood volume per visit	50		50	50		50	up to 52.5		
Cumulative blood volume[%]	50		100	150		200	252.5	252.5	

S = screening visit; (X) = if considered necessary; ** Timeline is approximate only. Exact timings of visits relate to the day on enrolment, ie, each visit must occur at indicated number of days after enrolment ± time window. ^Only temperature will be routinely measured at subsequent follow-up visits. At COVID-19 testing visits a full set observations will be taken, including respiratory rate and oxygen saturation. % Cumulative blood volume for volunteers if blood taken as per schedule, and excluding any repeat safety blood test that may be necessary. Blood volumes may vary according to local site equipment and practices. [a] Subject to site capacity, sample and test availability. [d] Optional and subject to site capacity. [b] Optional Stool sample at approximately 7 days after onset of symptoms for those who have a positive SARS-CoV-2 NAAT test result and possibly again at 14 days after symptom onset if necessary. [M] Females will be questioned about menstrual cycle prior to receiving boost vaccination.

Table 14 Unblinding and provision of treatment to controls (Protocol V17.0)

Attendance Number (boost)	Unblinding - This may occur remotely, electronically or as a trial visit (all remaining blinded participants)	Prime (controls only)	Boost (controls only)
Timeline** (days)	Following approval of protocol V17.0	Any point following unblinding	4 to 12 weeks post prime
Informed Consent		X	X
Review contraindications to ChAdOx1 nCoV-19 against the current UK Greenbook COVID-19 vaccination guidance		X	X
ChAdOx1 nCoV-19 Vaccination		X	X
Ascertainment of adverse events (SAE or AESIs only)		X	X
Participant Unblinding	X		
Exploratory immunology (mL)		up to 50 (depending on site capacity)	
Blood volume per visit	-	(50)	-

All participants (that remain blinded) will be unblinded following approval of protocol V17.0. Control participants aged 30 and above (only) will be invited to receive the MHRA approved 4-to-12 week ChAdOx1 nCoV-19 prime-boost schedule as part of provision of treatment to controls efforts. The visits in this table will happen ***concurrently*** with the remaining follow up visits and study procedures that are listed for each group (as per tables 6 to 13 above) which should remain unchanged following unblinding and provision of treatment. Contraindications to vaccination will be reviewed against the current UK Greenbook guidance on COVID-19 vaccination https://www.gov.uk/government/collections/immunisation-against-infectious-disease-the-green-book. Pregnancy testing will not be required prior to vaccination as this is not advised by the UK Greenbook.

7.4.4 Symptomatic volunteers

Participants who become symptomatic during follow-up will be instructed to call the study team who will then advise on how to proceed with clinical testing for COVID-19 if necessary, as per the trial working instructions. Participants will get weekly reminders (email or text messages) to get in touch with the study team if they present with a new onset of fever or cough or shortness of breath or anosmia/ageusia and if they are admitted to hospital for any reason.

7.4.4.1 Diagnostic SARS-CoV-2 NAAT testing outside of the trial

In a (primary endpoint symptom) symptomatic illness episode, if a participant has already received a negative SARS-CoV-2 test result via a validated NAAT assay that was performed after symptom onset and where this test was carried out outside of the study, they will now (as of protocol V15.0) be considered to be a negative case without requiring further NAAT testing by the study team. In such instances, documentation relating to the result should be acquired by investigators and filed as part of the individual participant record. Additionally, if investigators believe a symptomatic participant can obtain their initial diagnostic SARS-CoV-2 NAAT test more rapidly outside of the study (for logistical or other reasons) they may advise participants be tested via that route e.g. by being advised to arrange a test through a local NHS testing clinic or similar service. Where symptomatic participants report a SARS-CoV-2 test outside of the trial but are unable to provide details of the assay and/or documentation of the test result, they will be asked to attend the trial site for a COVID-19 testing visit.

7.4.4.2 COVID-19 testing visit

Participants will be invited for a COVID-19 testing visit in two circumstances:

- They report primary endpoint symptoms AND have not already had a documented negative SARS-CoV-2 NAAT test result (including tests performed outside of the trial) since their symptom onset
- They report primary endpoint symptoms and have had a positive SARS-CoV-2 NAAT outside of the trial since symptom onset

At the COVID-19 testing visit, a nose/throat swab and immunology bloods (paxgenes, cytokine profile, PBMCs, serum and others) will be acquired. Clinical assessments may be performed at COVID-19 testing visits at the discretion of investigators. Symptomatic volunteers may be regularly reviewed over the phone or via video call using a smartphone or computer app if clinically appropriate. Participants who have a positive NAAT will be reviewed by phone for symptom severity data at approximately 7 days post positive swab. Closer follow-up and safety monitoring may be carried out by local trial teams if felt this is clinically indicated. Symptomatic participants with a positive NAAT test outside of the trial will be asked to attend for a COVID-19 testing visit in

order to take a further swab to facilitate SARS-CoV-2 sequencing and additionally to acquire immunology bloods.

7.4.4.3 COVID-19 Testing plus 7 day phone call

For symptomatic participants with a positive SARS-CoV-2 NAAT test, a remote follow up (via phone or other appropriate means) will take place at approximately 7 days (see schedule of procedure tables for window, section 7.3) from the date of their first positive NAAT test in that illness episode. The purpose of this remote follow up is to capture further symptom severity data.

7.4.4.4 Positive SARS-CoV-2 NAAT test and vaccination

Participants who develop COVID-19 symptoms and have a positive NAAT test after the first vaccination can only receive a booster dose after a minimum 4 weeks interval from their first NAAT positive test, provided their symptoms have significantly improved. The decision to proceed with booster vaccinations in those cases will be at clinical discretion of the investigators. For participants who are asymptomatic and have a positive NAAT test (e.g. done outside the study), a minimum of 2 weeks from first NAAT positivity will be required before boosting.

7.4.5 Household Weekly Questionnaire (optional)

Participants will be asked to record information on a weekly basis about illnesses amongst household contacts and friends, their contact with the general public, and infection control procedures. This will be optional.

Volunteers will be asked to enter data in a diary from baseline to the end of the follow-up period. This will be recorded via a web-based electronic diary to which participants will be provided access at baseline.

7.4.6 Stool samples (optional)

Those participants who have a SARS-CoV-2 positive NAAT test result, may be asked to provide a stool sample at approximately 7 days after symptom onset and 14 days after the first sample if necessary, as per trial specific instructions. Samples will be processed to look at differences in viral shedding between the investigational vaccine and control arms, and to measure calprotectin levels as a marker of gastrointestinal inflammation. These samples will be collected and processed depending on test availability, laboratory capacity, and will not be compulsory to the volunteers. Further exploratory immunology and microbiology tests may be conducted at the investigators' discretion.

7.4.7 Medical notes review

With the participants consent, the study team will request access to medical notes or submit a data collection form for completion by attending clinical staff on any medically attended COVID-19 episodes. Any data which are relevant to ascertainment of efficacy endpoints and disease enhancement (AESI) will be collected. These are likely to include, but not limited to, information on ICU admissions, clinical parameters such as oxygen saturation, respiratory rates and vital signs, need for oxygen therapy, need for ventilatory support, imaging and blood tests results, amongst others.

7.4.8 Randomisation, blinding and code-breaking

Participants will be randomised to investigational vaccine or MenACWY in a 1:1 allocation, using block randomisation. Block sizes will reflect the numbers to be recruited at each stage of the study. The first block will be a block of 2 participants, followed by a block of 6, then blocks of 4 as required to meet the totals for randomisation for each day.

Participants enrolled in groups 1, 2 and 4 will be blinded to the arm they have been allocated to, whether investigational vaccine or control. The trial staff administering the vaccine will not be blinded. Vaccines will be prepared out of sight of the participant and syringes will be covered with an opaque object/material until ready for administration to ensure blinding.

Additional steps may be taken to keep clinical investigators assessing primary endpoints blinded to group allocation, where this is possible and practical to do so. A designated member of the clinical team may be unblinded for the purposes of safety reporting procedures.

If the clinical condition of a participant necessitates breaking the code, this will be undertaken according to a trial specific working instruction and group allocation sent to the attending physician, if unblinding is thought to be relevant and likely to change clinical management.

Participants enrolled in group 3 will not be randomised or blinded

In the event of other SARS-CoV-2 vaccines becoming approved for use by the MHRA, participants will be supported to access SARS-CoV-2 vaccines as early as possible, particularly the control group. To allow for a baseline consideration prior to receipt of a different vaccine, all participants who are unblinded and wish to receive an approved or authorised vaccine in line with government prioritisation will be asked to attend an extra visit before receiving the deployed vaccine where practical to do so. This is to ensure that they have a complete status before they become unevaluable for the trial arm they were in.

Those who are eligible (as per government prioritisation strategy) and have been invited to receive an approved or licensed SARS-CoV-2 vaccine can request to be unblinded, all participants will be kept in the study regardless of the combination of COVID-19 vaccines received.

Participants will be asked about the vaccine they received and date of administration which will be recorded along with other vaccines received in a specific externally administered vaccination case report form. Follow up visits will continue as per the participants previous schedule and their data will contribute to ongoing safety monitoring according to the vaccine combinations they have received. Participants will be censored in the analysis of efficacy endpoints at the time of their unblinding and vaccination, but will contribute to exploratory immunological analyses which are descriptive or observational according to the vaccines they have received. When participants have been unblinded they and the GP will receive a letter detailing the vaccines received in the study.

Further details of blinding of participants in groups 1c, 1d, 5a and 5b are described in section 7.4.10

7.4.8.1 Unblinding of all trial participants (protocol V17.0)

All trial participants will be unblinded following the approval of protocol V17.0. This follows the earlier completion of the primary efficacy analysis, the significant level of unblinding that has occurred due to the national vaccine rollout already within the trial and the fall in transmission of SARS-CoV-2 within the UK. Following unblinding and provision of COVID-19 vaccination (in or outside the study), participants will continue to follow all other study procedures and should continue to complete the visit schedule for their group. This will enable further analysis of immunogenicity, safety and immunological correlate of protection analysis.

Provision of treatment for controls is described in section 8.12. Following unblinding exploratory COVID-19 disease endpoints will be assessed by two trial team following pre-specified criteria, rather than independent blinded assessors.

7.4.9 Unblinded participants who are eligible to receive an approved or licensed SARS-CoV-2 vaccine

For those who choose to receive a deployed vaccine:

An extra visit may be arranged (where possible and practical to do so) for those who are offered and are willing to accept a SARS-CoV-2 approved or licensed vaccine where a blood sample for immunogenicity assessment (up to 50mL) may be drawn, regardless of which arm of the study they have been allocated to (ChAdOx1 nCoV-19 or MenACWY) or their COVID-19 status. SAEs and AESIs will be collected at this point. Participants will then follow their normal schedule of attendances according to the group they were previously randomised or allocated to.

For those who have received a single dose of the ChAdOx1 nCov-19 vaccine:

Those who are eligible to receive an approved or licensed SARS-CoV-2 vaccine, and who after unblinding are found to have received only a single dose of ChAdOx1 nCoV-19 will be given an option to receive a booster standard dose (ChAdOx1 nCoV-19 3.5-6.5x10^{10} vp) at an extra visit B, instead of a deployed vaccine, as per

national policy. The safety and benefit of mixed vaccine schedules is unknown. The extra visit B will take place ≥4 weeks post prime and should be conducted at the earliest possibility. The participant will be counselled on the timings of the planned vaccination and will be able to decide which vaccine to choose. SAEs and AESIs will be collected at this point. A blood sample for immunogenicity assessment (up to 50mL) may be drawn and participants will then follow their normal schedule of attendances according to the group they were previously randomised or allocated to. When participants have been unblinded they and the GP will receive a letter detailing the vaccines received in the study.

7.4.10 Late Vaccination sub-groups

The rationale for these groups is listed in section 3.5 and the process of allocating these participants is described below.

Allocation of participants to late vaccination sub-groups (1c and 1d, 5a and 5b):

Allocation of group 1 and 4 participants to late vaccination sub-groups (1c, 1d, 5a and 5b) will be carried out locally by unblinded study team members. An overall 2:1 allocation ratio of participants previously randomised to ChAdOx1 nCoV-19 versus MenACWY recipients will be used. Late vaccination timepoints will occur 10 months +/-56 days from enrolment.

Individual participants from groups 1a and 1b (previous single dose groups) will be contacted and offered re-allocation into 1c (the long interval two-dose ChAdOx1 nCoV-19 group) and 1d (a newly dosed ChAdOx1 nCoV-19 group, assigned from participants receiving single dose MenACWY earlier in the trial) respectively.

Participants from groups 2c, 2f, 4c, 2e, 2g and 4d (the previous two-dose groups) will be offered reallocation into group 5. Those previously randomised to ChAdOx1 nCoV-19 (2c, 2f and 4c) will become group 5a (the long interval three-dose ChAdOx1 nCoV-19 group) and those previously randomised to MenACWY (2e, 2g and 4d) will become group 5b (a newly dosed ChAdOx1 nCoV-19 group, assigned from participants receiving two-dose MenACWY earlier in the trial).

Participants who agree to the reallocation will be re-consented prior to study procedures. Participants may decline to be reallocated and in this circumstance will remain within their previous subgroup.

Specific criteria for reallocation to groups 1c, 1d, 5a and 5b sub-groups are as follows (and figure 3):

Group 1c, 1d, 5a and 5b participants **must**:

- Not have received SARS-CoV-2 vaccines outside of the study OR as part of “Extra Visit B”.

Additionally, where possible, allocation to group 5a and 5b will preferentially include participants with the following characteristics:

- Negative baseline SARS-CoV-2 serology (if carried out)

- No positive COVID-19 NAAT tests during the preceding study period
- Remain blinded to their preceding study vaccinations. Due to the small group size of group 2c (n=20), all participants in that group will be offered reallocation into 5a regardless of blinding status.
- **Must** have a ≤ 16 week interval between their initial first and second vaccine doses within the trial

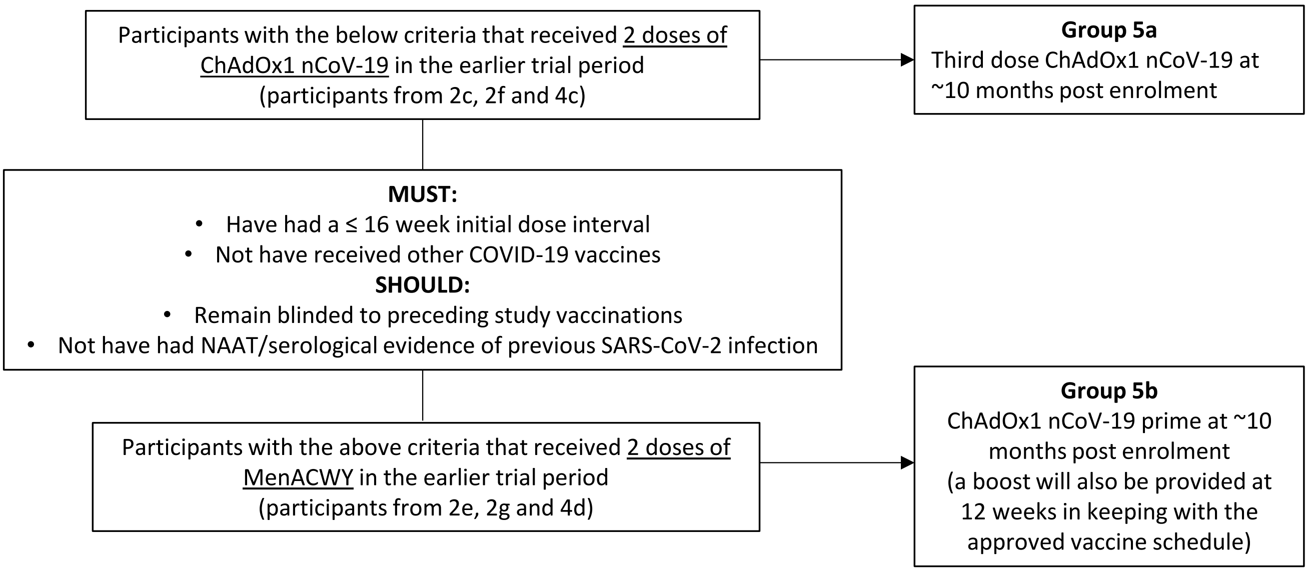

Figure 3: Allocation procedures for group 5: Third ChAdOx1 nCoV-19 dose (5a) and newly ChAdOx1 nCoV-19 dosed (5b)

Blinding and unblinding (1c, d, 5a and 5b)

All participants in sub-groups 1c, 1d, 5a and 5b will receive an unblinded ChAdOx1 vaccine at the "late vaccination" visit (introduced in protocol v15.0). They will remain blinded to the vaccination(s) they received earlier in the trial until they have completed at least 7 days of follow up following the late vaccination visit. After this period, participants in these subgroups will be unblinded to their earlier vaccine(s) in order to arrange the "Late vaccination 2" booster visit for participants in groups 1d and 5b.

Allocation of participants to late vaccination sub-groups (3b)

All group 3 participants (4 week interval unblinded two-dose group) will be offered a third ChAdOx1 nCoV-19 dose (group 3b). Participants who agree to the reallocation will be re-consented prior to study procedures. Participants may decline to be reallocated and in this circumstance will remain within their previous subgroup (now renamed group 3a).

8 INVESTIGATIONAL PRODUCT AND TRIAL INTERVENTIONS

8.1 Manufacturing and presentation

8.1.1 Description of ChAdOx1 nCoV-19

ChAdOx1 nCoV-19 vaccine consists of the replication-deficient simian adenovirus vector ChAdOx1, containing the structural surface glycoprotein (Spike protein) antigens of SARS-CoV-2.

8.2 Supply

ChAdOx1 nCoV-19 has been formulated and vialed at the Clinical BioManufacturing Facility (CBF), University of Oxford or Advent Srl, Italy. At the CBF the vaccine will be certified and labelled for the trial by a Qualified Person (QP) before transfer to the clinical site.

ChAdOx1 nCoV-19 (AZD1222) has been formulated at Cobra Biologics Ltd, vialed at Symbiosis Pharmaceutical Services, and labelled and packaged at Thermo Fisher Scientific (Hertfordshire, United Kingdom). It will be certified by a Qualified Person (QP) at the MedImmune Pharma, BV (Nijmegen, The Netherlands) or MedImmune Ltd (Cambridge, United Kingdom) before release and transfer to the clinical site.

8.3 Storage

The vaccine manufactured at CBF or Advent Srl is stored at nominal -80°C in a secure freezer, at the clinical site. The vaccine manufactured by Cobra Biologics Ltd is stored at 2-8°C in a secure fridge, at the clinical site. All movements of the study vaccines will be documented in accordance with existing standard operating procedure (SOP). Vaccine accountability, storage, shipment and handling will be in accordance with relevant SOPs and forms. To allow for large number of participants to receive the vaccine in a short time period additional clinic locations may be used. In this instance vaccines will be transported in accordance with local SOP's and approvals as required.

8.4 Administration

For Advent manufactured vaccine: On vaccination day, ChAdOx1 nCoV-19 will be allowed to thaw to room temperature and will be administered within 1 hour of removal from storage.

For Cobra manufactured vaccine: The vaccine manufactured by Cobra Biologics is a multi-dose vial which is stored at 2-8 degrees and does not require thawing. If the vaccine is stored outside of 2-8 it must be used within 6 hours. If stored at 2-8°C after the first vial puncture, it can be used within 48 hours.

The vaccine will be administered intramuscularly into the deltoid of the non-dominant arm (preferably). Prior to protocol V17.0: All volunteers will be observed in the unit for a minimum of 1 hour (±30 minutes) after vaccination for the prime dose and a minimum of 15 minutes after the booster vaccination. Following protocol V17.0 approval: In line with UK Greenbook recommendations, participants do not need to be routinely observed following either prime (1st) or boost (2nd) dose of ChAdOx1 nCoV-19. During administration of the investigational products, Advanced Life Support drugs and resuscitation equipment will be immediately available for the management of anaphylaxis. Vaccination will be performed and the IMPs handled according to the relevant SOPs.

8.5 Rationale for selected dose

The dose to be administered in this trial have been selected on the basis of clinical experience with the ChAdOx1 adenovirus vector expressing different inserts and other similar adenovirus vectored vaccines (eg. ChAd63).

A first-in-man dose escalation study using the ChAdOx1 vector encoding an influenza antigen (FLU004), safely administered ChAdOx1 NP+M1 at doses ranging from 5 x 10^{8} to 5 x 10^{10} vp. Subsequent review of the data identified an optimal dose of 2.5 x 10^{10} vp balancing immunogenicity and reactogenicity. This dose has subsequently been given to over hundreds of volunteers in numerous larger phase 1 studies at the Jenner Institute. ChAdOx1 vectored vaccines have thus far demonstrated to be very well tolerated. The vast majority of AEs have been mild-moderate and there have been no SARs until this date.

Another simian adenovirus vector (ChAd63) has been safely administered at doses up to 2 x 10^{11} vp with an optimal dose of 5 x 10^{10} vp, balancing immunogenicity and reactogenicity.

MERS001 was the first clinical trial of a ChAdOx1 vectored expressing the full-length Spike protein from a separate, but related betacoronavirus. ChAdOx1 MERS has been given to 31 participants to date at doses ranging from 5x10^{9} vp to 5x10^{10} vp. Despite higher reactogeniticy observed at the 5x10^{10} vp, this dose was safe, with self-limiting AEs and no SARs recorded. The 5x10^{10} vp was the most immunogenic, in terms of inducing neutralising antibodies against MERS-CoV using a live virus assay (Folegatti et al. Lancet Infect Dis, 2020, in press). Given the immunology findings and safety profile observed with a ChAdOx1 vectored vaccine against MERS-CoV, the 5x10^{10} vp dose was chosen for ChAdOx1 nCoV-19.

As this is a first-in-human assessment of the SARS-CoV-2 S antigenic insert, a staggered enrolment will apply for the first volunteers enrolled in the study. The same procedure will apply, should other batches of ChAdOx1 nCoV-19 become available. Safety of ChAdOx1 nCoV-19 will be monitored in real time. and should unacceptable adverse events or safety concerns arise, doses will be decreased via an amendment.

An analytical comparability assessment of ChAdOx1 nCoV-19 (AZD1222) manufactured by CBF, Advent and Cobra Biologics was conducted using a comprehensive set of physiochemical and biological release and characterization tests. In order to support the analytical comparability assessment, A260 testing of Advent's process (K.0007, K.0008, and K.0009 lots) was performed, where corrections to the absorbance due to excess polysorbate 80 were made to compensate for polysorbate 80 concentrations above the formulation target of 0.1% (w/v).

Differences in strength related attributes (ie, virus particle concentration, virus genome concentration, and infectious virus concentration) are noted. These differences in strength is further examined for potential impact on clinical dosing. The target clinical dosage of CBF's product is 5×10^{10} viral particles per dose based on vp/mL concentration determined by UV spectroscopy (A260), whereas that of Advent's product is 5×10^{10} viral genome copies per dose based on vg/mL concentration determined by qPCR. The target clinical dosage of Symbiosis' product is $3.5 – 6.5 \times 10^{10}$ viral particles per dose based on the vp/mL concentration determined by A260, with a 0.5 mL dosing volume. This dosing range is based on a target 5×10^{10} viral particles per dose and a ± 30% range to take into account process and method variabilities. When the planned clinical dosage of Symbiosis' product is compared to that of CBF and Advent products, the resulting Symbiosis' product dosage at 0.5 mL for lot 20481A is somewhat lower in total viral particle per dose (20% from the lower range limit), slightly higher in total viral genome copies per dose (12% from the higher range limit), and slightly lower in total infectious particle per dose (8% from the lower range limit). These differences are considered to be comparable to or within the variabilities from the analytical methods used in concentration determination (A260, qPCR, and infectivity) and the dosing volumes during clinical administration. In summary, with a 0.5 mL dosing volume for Symbiosis' product, the strength difference from CBF and Advent products is not expected to have significant clinical impact in terms of reactogenicity and immunogenicity/efficacy.

Table 12 Clinical Strengths of ChAdOx1 nCoV-19 (AZD1222) Drug Product

Strength Attribute	CBF		Advent			Cobra
	Lot 02P20-01	Lot 02P20-02	Lot K.0007	Lot K.0008	Lot K.0009	Lot 20481A
**Concentration**						
Virus particle concentration (A_{260}) (vp/mL)	1.49×10^{11}	1.22×10^{11}	3.12×10^{11}	3.16×10^{11}	2.45×10^{11}	0.8×10^{11}
Virus genome concentration (qPCR) (vg/mL)	1.7×10^{11}	Not tested	1.7×10^{11}	2.1×10^{11}	1.4×10^{11}	1.3×10^{11}
Infectious particle concentration (ifu/mL) [a]	2.6×10^{9}	Not tested	2.9×10^{9}	3.0×10^{9}	2.4×10^{9}	1.3×10^{9}
**Target Clinical Dosage**						

Table 12 Clinical Strengths of ChAdOx1 nCoV-19 (AZD1222) Drug Product

Strength Attribute	CBF		Advent			Cobra
	Lot 02P20-01	Lot 02P20-02	Lot K.0007	Lot K.0008	Lot K.0009	Lot 20481A
Equivalent DP volume per dose (mL)	0.34	0.41	0.294	0.235	0.356	0.50
Dosing of virus particle (vp/dose)	5.1×10^{10}	5.0×10^{10}	9.2×10^{10}	7.4×10^{10}	8.7×10^{10}	4.0×10^{10}
Dosing of viral genome (vg/dose)	5.8×10^{10}	NA	5.0×10^{10}	4.9×10^{10}	5.0×10^{10}	6.5×10^{10}
Dosing of infectious particle (ifu/dose)	8.8×10^{8}	NA	8.5×10^{8}	7.1×10^{8}	8.5×10^{8}	6.5×10^{8}

ifu = infectious units; NA = not applicable; vp = virus particle; vg = virus genome

[a] Testing performed using the Advent infectivity assay.

8.6 Minimising environmental contamination with genetically modified organisms (GMO)

The study will be performed in accordance with the current version of the UK Genetically Modified Organisms (Contained Use) Regulations. Approved SOPs will be followed to minimise dissemination of the recombinant vectored vaccine virus into the environment. GMO waste will be inactivated according to approved SOPs.

8.7 Control Vaccine

Participants who are allocated to the control groups will receive one or two injections of MenACWY vaccine instead of ChAdOx1 nCoV-19. Either of the two licensed quadrivalent protein-polysaccharide conjugate vaccine MenACWY vaccines will be used, i.e.:

- Nimenrix (Pfizer). The licensed posology of this vaccine for those over 6 months of age is a single (0.5ml) intramuscular dose, containing 5mcg each of *Neisseria meningitidis* group A, C, W and Y polysaccharide, each conjugated to 44 mcg tetanus toxoid carrier protein.
- Menveo (Glaxosmithkline). The licensed posology of this vaccine for those 2 years of age and over is a single (0.5ml) intramuscular dose, containing
    - 10 mcg meningococcal group A polysaccharide, conjugated to 16.7 to 33.3 mcg *Corynebacterium diphtheriae* CRM_{197} protein
    - 5mcg meningococcal group C polysaccharide, conjugated to 7.1 to 12.5 mcg *C. diphtheriae* CRM_{197} protein
    - 5mcg meningococcal group W polysaccharide, conjugated to 3.3 to 8.3 mcg *C. diphtheriae* CRM_{197} protein
    - 5mcg meningococcal group Y polysaccharide, conjugated to 5.6 to 10.0 mcg *C. diphtheriae* CRM_{197} protein

The summary of product characteristics for both vaccines allows for administration of a booster dose if indicated by ongoing risk, therefore allows for the two doses administered to a subset of participants in this study. Similarly, previous receipt of either vaccine (or a plain polysaccharide quadrivalent meningococccal A, C, W and Y vaccine) will not be a contraindication to receiving a further vaccine in this study.

Participants will be blinded as to which intervention they are receiving. A vaccine accountability log of MenACWY will be maintained at each trial site. There will be no additional labelling of these vaccines beyond their licensed packaging.

MenACWY will be stored in a locked (or access controlled) refrigerator (2°C – 8°C) at the sites, as per SmPC.

8.8 Compliance with Trial Treatment

All vaccinations will be administered by the research team and recorded in the CRF. The study medication will be at no time in the possession of the participant and compliance will not, therefore, be an issue.

8.9 Accountability of the Trial Treatment

Accountability of the IMP and control vaccine will be conducted in accordance with the relevant SOPs.

8.10 Paracetamol (non-IMP)

Paracetamol will be provided to a subset of participants in group 4 to be taken at vaccination day for 24hours.

Participants in groups 2f, 2g, 4c, and 4d will be advised to take prophylactic paracetamol for 24h post booster vaccine.

8.11 Concomitant Medication

As set out by the exclusion criteria, volunteers may not enter the study if they have received: any vaccine in the 30 days prior to enrolment or there is planned receipt of any other vaccine within 30 days of each vaccination, any investigational product within 30 days prior to enrolment or if receipt is planned during the study period, or if there is any use of immunosuppressant medication within 6 months prior to enrolment or if receipt is planned at any time during the study period (except topical steroids and short course of low dose steroids < 14 day). Participants who become eligible for a SARS-CoV-2 vaccine according to government guidance will be able to receive the vaccine as recommended. We will advise participants to allow a minimum period of 2 weeks between vaccination with MenACWY and the SARS-CoV-2 vaccine for those in the control group and 21 days between vaccination with ChAdOx1 nCoV-19 and the SARS-CoV-2 vaccine. Participants will be asked to record the date and type of vaccine received.

8.12 Provision of Treatment for Controls

If this vaccine is proven to be efficacious following analysis of the primary endpoint and if the DSMB agrees, participants allocated to MenACWY control group may be offered the IMP, should extra doses become available.

(Protocol V17.0) Following unblinding, all control participants will be offered ChAdOx1 nCoV-19 in the approved MHRA 4-to-12 week, 2-dose schedule (table 14). Participants will remain enrolled in the study and continue the follow up visit schedule listed for their sub-group *concurrently* with the provision of treatment prime and boost visits.

Due to the updated guidance relating to the emerging association of thrombosis with thrombocytopenia and ChAdOx1 nCoV-19, trial participants aged 29 and under will not be offered (prime) vaccinations with ChAdOx1 nCoV-19. These individuals will instead be advised to await vaccination under the national rollout program.

9 ASSESSMENT OF SAFETY

Safety will be assessed by the frequency, incidence and nature of AEs and SAEs arising during the study.

9.1 Definitions

9.1.1 Adverse Event (AE)

An AE is any untoward medical occurrence in a volunteer, which may occur during or after administration of an IMP and does not necessarily have a causal relationship with the intervention. An AE can therefore be any unfavourable and unintended sign (including any clinically significant abnormal laboratory finding or change from baseline), symptom or disease temporally associated with the study intervention, whether or not considered related to the study intervention.

9.1.2 Adverse Reaction (AR)

An AR is any untoward or unintended response to an IMP. This means that a causal relationship between the IMP and an AE is at least a reasonable possibility, i.e., the relationship cannot be ruled out. All cases judged by the reporting medical Investigator as having a reasonable suspected causal relationship to an IMP (i.e. possibly, probably or definitely related to an IMP) will qualify as AR.

Adverse events that may be related to the IMP are listed in the Investigator's Brochure for each product.

9.1.3 Serious Adverse Event (SAE)

An SAE is an AE that results in any of the following outcomes, whether or not considered related to the study intervention.

- Death
- Life-threatening event (i.e., the volunteer was, in the view of the Investigator, at immediate risk of death from the event that occurred). This does not include an AE that, if it occurred in a more severe form, might have caused death.
- Persistent or significant disability or incapacity (i.e., substantial disruption of one's ability to carry out normal life functions).
- Hospitalisation or prolongation of existing hospitalisation, regardless of length of stay, even if it is a precautionary measure for continued observation. Hospitalisation (including inpatient or outpatient hospitalisation for an elective procedure) for a pre-existing condition that has not worsened unexpectedly does not constitute a serious AE.
- An important medical event (that may not cause death, be life threatening, or require hospitalisation) that may, based upon appropriate medical judgment, jeopardise the volunteer and/or require medical or surgical intervention to prevent one of the outcomes listed above. Examples of such medical events

include allergic reaction requiring intensive treatment in an emergency room or clinic, blood dyscrasias, or convulsions that do not result in inpatient hospitalisation.

- Congenital anomaly or birth defect.

9.1.4 Serious Adverse Reaction (SAR)

An AE that is both serious and, in the opinion of the reporting Investigator or Sponsors, believed to be possibly, probably or definitely due to an IMP or any other study treatments, based on the information provided.

Reporting of SARs will also apply to concomitant medications, including any other approved or licensed SARS-CoV-2 vaccine, where there is the possibility of an interaction between study interventions and those concomitant medications.

9.1.5 Suspected Unexpected Serious Adverse Reaction (SUSAR)

A SAR, the nature and severity of which is not consistent with the information about the medicinal product in question set out in the IB.

9.2 Expectedness

No IMP related SAEs are expected in this study. All SARs will therefore be reported as SUSARs.

9.3 Foreseeable Adverse Reactions:

The foreseeable ARs following vaccination with ChAdOx1 nCoV-19 include injection site pain, tenderness, erythema, warmth, swelling, induration, pruritus, myalgia, arthralgia, headache, fatigue, fever, feverishness, chills, malaise and nausea.

9.4 Adverse Events of Special Interest (AESI)

- Disease enhancement following vaccination with ChAdOx1 nCoV-19 will be monitored. Severe COVID-19 disease will be defined using clinical criteria. Detailed clinical parameters will be collected from medical records and aligned with agreed definitions as they emerge. These are likely to include, but are not limited to, oxygen saturation, need for oxygen therapy, respiratory rate, need for ventilatory support, imaging and blood test results, amongst other clinically relevant parameters.
- Acute respiratory distress, pneumonitis, acute cardiac injury, arrhythmia, septic-shock like syndrome and acute kidney injury related with COVID-19 disease will be monitored from medical records review of hospitalised participants.
- Neurological events of >Grade 2 severity

- Eosinophilia as a marker skewed Th2 responses will be routinely monitored in participants attending their COVID-19 testing and follow-up visits. Marked eosinophilia of ≥ 1.5 $x10^9$/L will be reported as SAEs.
- AESI relevant to vaccination in general will also be monitored such as: generalised convulsion, Guillain-Barre Syndrome (GBS), Acute Disseminated Encephalomyelitis (ADEM), Thrombocytopenia, Anaphylaxis, Vasculitides in addition to serious solicited AEs will be monitored.
- (Protocol V18.0) The following conditions will be recorded as AESIs:
  - Cerebral Venous Sinus Thrombosis
  - Heparin-Induced Thrombocytopenia
  - Major thrombosis with concurrent thrombocytopenia

9.5 Causality

For every AE, an assessment of the relationship of the event to the administration of the vaccine will be undertaken by the CI-delegated clinician. An interpretation of the causal relationship of the intervention to the AE in question will be made, based on the type of event; the relationship of the event to the time of vaccine administration; and the known biology of the vaccine therapy (Table 11). Alternative causes of the AE, such as the natural history of pre-existing medical conditions, concomitant therapy, other risk factors and the temporal relationship of the event to vaccination will be considered and investigated. Causality assessment will take place during planned safety reviews, interim analyses (e.g. if a holding or stopping rule is activated) and at the final safety analysis, except for SAEs, which should be assigned by the reporting investigator, immediately, as described in SOP OVC005 Safety Reporting for CTIMPs.

0	**No Relationship**	No temporal relationship to study product ***and***<br>Alternate aetiology (clinical state, environmental or other interventions); ***and***<br>Does not follow known pattern of response to study product
1	**Unlikely**	Unlikely temporal relationship to study product ***and***<br>Alternate aetiology likely (clinical state, environmental or other interventions) ***and***<br>Does not follow known typical or plausible pattern of response to study product.

2	**Possible**	Reasonable temporal relationship to study product; ***or***<br>Event not readily produced by clinical state, environmental or other interventions; ***or***<br>Similar pattern of response to that seen with other vaccines
3	**Probable**	Reasonable temporal relationship to study product; ***and***<br>Event not readily produced by clinical state, environment, or other interventions ***or***<br>Known pattern of response seen with other vaccines
4	**Definite**	Reasonable temporal relationship to study product; ***and***<br>Event not readily produced by clinical state, environment, or other interventions; ***and***<br>Known pattern of response seen with other vaccines

Table 11. *Guidelines for assessing the relationship of vaccine administration to an AE.*

9.6 Reporting Procedures for All Adverse Events

All local and systemic AEs occurring in the 28 days following each study vaccination (except "late vaccination visit" and "Extra Visit B" vaccines, see below) observed by the Investigator or reported by the volunteer, whether or not attributed to study medication, will be recorded in electronic diaries or study database. AEs occurring following vaccination at Extra Visit B will not be recorded, unless meeting criteria for AESI or SAE. Following late vaccination visit timepoints (groups 1c, 1d,5a and 5b) all local and systemic AEs will be collected for a period of 7 days. Only SAEs and AESI will be recorded for participants who are unblinded and receive an approved/licensed COVID-19 vaccine either as part of the national roll-out strategy OR vaccines administered as part of provision of treatment to controls (introduced in protocol V17.0). All AEs that result in a volunteer's withdrawal from the study will be followed up until a satisfactory resolution occurs (if the volunteer consents to this), or until a non-study related causality is assigned.

9.7 SAE and AESI collection period

(As of protocol V16.0) SAEs and Adverse Events of Special Interest will be actively collected from enrolment until 1st July 2021 except where participants have received a "late boost" vaccination in the trial (groups 1c, 1d, 3a, 3b, 5a and 5b), in which case SAEs and AESIs will instead be actively collected until the LV182 visit (i.e. 6 months after the late boost vaccination visit).

Where participants spontaneously report SAEs and AESIs following the end of the active collection period defined above these will still be recorded and reported to relevant parties.

9.8 Assessment of severity

The severity of clinical and laboratory adverse events will be assessed according to scales based on FDA toxicity grading scales for healthy and adolescent volunteers enrolled in preventive vaccine clinical trials, listed in the study specific working instructions and tables 11-13 below,

Adverse Event	Grade	Intensity
Pain at injection site	1	Pain that is easily tolerated
	2	Pain that interferes with daily activity
	3	Pain that prevents daily activity
	4	A&E visit or hospitalization
Tenderness	1	Mild discomfort to touch
	2	Discomfort with movement
	3	Significant discomfort at rest
	4	A&E visit or hospitalization
Erythema at injection site*	1	2.5 - 5 cm
	2	5.1 - 10 cm
	3	>10 cm
	4	Necrosis or exfoliative dermatitis
Induration/Swelling at injection site	1	2.5 – 5 cm and does not interfere with activity
	2	5.1 - 10 cm or interferes with activity
	3	>10 cm or prevents daily activity
	4	Necrosis

Table 12. Severity grading criteria for local adverse events *erythema ≤2.5cm is an expected consequence of skin puncture and will therefore not be considered an adverse event

Vital Signs	Grade 1 (mild)	Grade 2 (moderate)	Grade 3 (severe)	Grade 4 Potentially Life threatening
Fever (oral)	38.0°C - 38.4°C	38.5°C – 38.9°C	39.0°C - 40°C	> 40°C
Tachycardia (bpm)*	101 - 115	116 – 130	>130	A&E visit or hospitalisation for arrhythmia
Bradycardia (bpm)**	50 – 54	45 – 49	<45	A&E visit or hospitalisation for arrhythmia
Systolic hypertension (mmHg)	141 - 150	151 – 155	≥155	A&E visit or hospitalization for malignant hypertension
Diastolic hypertension (mmHg)	91 - 95	96 – 100	>100	A&E visit or hospitalization for malignant hypertension
Systolic hypotension (mmHg)***	85 - 89	80 – 84	<80	A&E visit or hospitalization for hypotensive shock
Respiratory Rate –breaths per minute	17 - 20	21-25	>25	Intubation

Table 13. ***Severity grading criteria for physical observations.*** **Taken after ≥10 minutes at rest **When resting heart rate is between 60 – 100 beats per minute. Use clinical judgement when characterising bradycardia among some healthy subject populations, for example, conditioned athletes. ***Only if symptomatic (e.g. dizzy/ light-headed)*

**GRADE 0**	None
**GRADE 1**	Mild: Transient or mild discomfort (< 48 hours); No interference with activity; No medical intervention/therapy required
**GRADE 2**	Moderate: Mild to moderate limitation in activity – some assistance may be needed; no or minimal medical intervention/therapy required
**GRADE 3**	Severe: Marked limitation in activity, some assistance usually required; medical intervention/therapy required.
**GRADE 4**	Potentially Life-threatening: requires assessment in A&E or hospitalisation

Table 14. ***Severity grading criteria for local and systemic AEs.***

9.9 Reporting Procedures for Serious AEs

In order to comply with current regulations on SAE reporting to regulatory authorities, the event will be documented accurately and notification deadlines respected. SAEs will be reported on the SAE forms to members of the study team immediately after the Investigators become aware of their occurrence, as described in SOP OVC005 Safety Reporting for CTIMPs. Copies of all reports will be forwarded for review to the Chief Investigator (as the Sponsor's representative) within 24 hours of the Investigator being aware of the suspected SAE. The DSMB will be notified of SAEs that are deemed possibly, probably or definitely related to study interventions; the chair of DSMB will be notified immediately (within 24 hours) of the Sponsor being aware of their occurrence. SAEs will not normally be reported immediately to the ethical committee(s) unless there is a clinically important increase in occurrence rate, an unexpected outcome, or a new event that is likely to affect safety of trial volunteers, at the discretion of the Chief Investigator and/or DSMB. In addition to the expedited reporting above, the Investigator shall include all SAEs in the annual Development Safety Update Report (DSUR) report.

Cases falling under the Hy's Law should be reported as SAEs. A Hy's Law Case is defined by FDA Guidance for Industry "Drug-Induced Liver Injury: Premarketing Clinical Evaluation" (2009). Any study subject with an increase in Aspartate Aminotransferase (AST) or **Alanine Aminotransferase (ALT) ≥ 3x Upper Limit of Normal (ULN) together with Total Bilirubin ≥2xULN**, **where no other reason can be found to explain the combination of increases**, e.g., elevated serum alkaline phosphatase (ALP) indicating cholestasis, viral hepatitis A, B or C, or another drug capable of causing the observed injury.

9.10 Reporting Procedures for SUSARS

All SUSARs (including SUSARs related to the non-IMP where there is a possibility of an interaction between the non-IMP and IMP) will be reported by the sponsor delegate to the relevant Competent Authority and to the REC and other parties as applicable. For fatal and life-threatening SUSARS, this will be done no later than 7 calendar days after the Sponsor or delegate is first aware of the reaction. Any additional relevant information will be reported within 8 calendar days of the initial report. All other SUSARs will be reported within 15 calendar days.

Principal Investigators will be informed of all SUSARs for the relevant IMP for all studies with the same Sponsor, whether or not the event occurred in the current trial.

9.11 Development Safety Update Report

A Development Safety Update Report (DSUR) will be prepared annually, within 60 days of the anniversary of the first approval date from the regulatory authority for each IMP. The DSUR will be submitted by the CI to the Competent Authority, Ethics Committee, HRA (where required), Host NHS Trust and Sponsor.

9.12 Procedures to be followed in the event of abnormal findings

Eligibility for enrolment in the trial in terms of laboratory findings will be assessed by clinically qualified staff. Abnormal clinical findings from medical history, examination or blood tests will be assessed as to their clinical significance throughout the trial. Laboratory AEs will be assessed using specific toxicity grading scales adapted from the FDA Toxicity Grading Scale for Healthy Adult and Adolescent Volunteers Enrolled in Preventive Vaccine Clinical Trials. If a test is deemed clinically significant, it may be repeated, to ensure it is not a single occurrence. If a test remains clinically significant, the volunteer will be informed and appropriate medical care arranged as appropriate and with the permission of the volunteer. Decisions to exclude the volunteer from enrolling in the trial or to withdraw a volunteer from the trial will be at the discretion of the Investigator.

9.13 Interim Reviews

The safety profile will be assessed on an on-going basis by the Investigators. The CI and relevant Investigators (as per the trial delegation log) will also review safety issues and SAEs as they arise.

Interim safety reviews are planned after the first volunteer in the intervention arm and after the first 4 participants have been given a dose of the IMP.

Data from pre-clinical studies will be assessed by the CI, relevant investigators and the DSMB a soon as they are available and before up to 100 volunteers receive a dose of the IMP.

Safety data available from the first up to 54 volunteers receiving a dose of the IMP will be reviewed by the CI, relevant investigators and the chair of DSMB before proceeding with vaccination in the remaining volunteers.

The DSMB will review safety data accumulated when the study is fully recruited.

The DSMB will evaluate frequency of events, safety and efficacy data every 4-8 weeks and/or as required. The DSMB will make recommendations concerning the conduct, continuation or modification of the study.

9.14 Data Safety Monitoring Board

A Data Safety Monitoring Board will be appointed to

a) periodically review and evaluate the accumulated study data for participant safety, study conduct, progress, and efficacy.

b) make recommendations concerning the continuation, modification, or termination of the trial.

There will be a minimum of three appropriately qualified committee members of whom one will be the designated chair. The DSMB will operate in accordance with the trial specific charter, which will be established before recruitment starts.

The chair of the DSMB may be contacted for advice and independent review by the Investigator or trial Sponsor in the following situations:

- Following any SAE deemed to be possibly, probably or definitively related to a study intervention.
- Any other situation where the Investigator or trial Sponsor feels independent advice or review is important.

The DSMB will review SAEs deemed possibly, probably or definitively related to study interventions. The DSMB will be notified within 24 hours of the Investigators' being aware of their occurrence. The DSMB has the power to place the study on hold if deemed necessary following a study intervention-related SAE.

9.15 Safety Group Holding Rules

Safety holding rules have been developed considering the fact that this is a first-in-human study. Safety holding rules apply to participants receiving ChAdOx1 nCoV-19 only.

Solicited AEs are those listed as foreseeable ARs in section 9.3 of the protocol, occurring within the first 7 days after vaccination (day of vaccination and six subsequent days). 'Unsolicited adverse events' are adverse events other than the foreseeable ARs occurring within the first 7 days, or any AEs occurring after the first 7 days after vaccination

9.15.1 Group holding rules

For safety reasons, the first volunteer to receive a new vaccine will be vaccinated alone and the trial Investigators will wait 48 hours (±24 hours) before vaccinating subsequent volunteers. Three further volunteers may be vaccinated 48 hours (±24 hours) after the first and then at least another 48 hours (±24 hours) gap will be left before vaccinating the rest of the volunteers. Group holding rules mentioned below will apply to all study Groups

- **Solicited local adverse events:**
  - If more than 25% of doses of the vaccine at a given time point (e.g. Day 0, Day 28) in a study group are followed by the same Grade 3 solicited local adverse event beginning within 2 days after vaccination (day of vaccination and one subsequent day) and persisting at Grade 3 for >72 hrs
- **Solicited systemic adverse events:**
  - If more than 25% of doses of the vaccine at a given time point (e.g. Day 0, Day 28) in a study group are followed by the same Grade 3 solicited systemic adverse event beginning within 2 days after vaccination (day of vaccination and one subsequent day) and persisting at Grade 3 for >72 hrs
- **Unsolicited adverse events:**
  - If more than 25% of doses of the vaccine at a given time point (e.g. Day 0, Day 28) in a study group are followed by the same Grade 3 unsolicited adverse event beginning within 2 days after vaccination (day of vaccination and one subsequent day) and persisting at Grade 3 for >72 hrs
- **Laboratory adverse event:**
  - If more than 25% of doses of the vaccine at a given time point (e.g. Day 0, Day 28) in a study group are followed by the same Grade 3 laboratory adverse event beginning within 3 days after vaccination and persisting at Grade 3 for >72 hrs
- **A serious adverse event considered possibly, probably or definitely related to vaccination occurs**
  - If an SAE occurs in any one individual, which is possibly, probably or definitely related to vaccination this would trigger a holding rule. There are two exemptions from this rule, which would not activate a holding rule. These include:

- COVID-19 related hospital admissions considered to be at least possibly related to ChAdOx1 nCoV-19 (e.g. if considered to be a clinical presentation of a disease enhancement episode). COVID-19 related SAEs will be regularly reviewed by the DSMB, and a single event will not trigger a holding rule.
- SAEs reported under the Hy's Law requirement will not necessarily trigger a holding rule. These cases will also be reviewed by the DSMB

If any of the above holding rules are activated, then further vaccinations in any group will not occur until a safety review by the DSMB, study sponsor and the chief investigator has been conducted and it is deemed appropriate to restart dosing. The Regulatory Authority will be informed and a request to restart dosing with pertinent data will be submitted as a substantial amendment. The safety review will consider:

- The relationship of the AE or SAE to the vaccine.
- The relationship of the AE or SAE to the vaccine dose, or other possible causes of the event.
- If appropriate, additional screening or laboratory testing for other volunteers to identify those who may develop similar symptoms and alterations to the current Participant Information Sheet (PIS) are discussed.
- New, relevant safety information from ongoing research programs on the various components of the vaccine.

The local ethics committee and vaccine manufacturers will also be notified if a holding rule is activated or released.

All vaccinated volunteers will be followed for safety until resolution or stabilisation (if determined to be chronic sequelae) of their AEs.

Since the approval of ChAdOx1 nCoV-19 for emergency use by the MHRA and its subsequent national roll-out, group holding rules will no longer apply. Any SUSARs occurring in the trial from the time of approval onwards will be notified to and discussed with the DSMB and the MHRA, but will no longer automatically trigger a holding rule.

9.15.2 Individual stopping rules (will apply to prime-boost group only)

In addition to the above stated group holding rules, stopping rules for individual volunteers will apply (i.e., indications to withdraw individuals from further vaccinations). Study participants who present with at least one of the following stopping rules will be withdrawn from further vaccination in the study:

- **Local reactions:** Injection site ulceration, abscess or necrosis

- **Laboratory AEs:**

the volunteer develops a Grade 3 laboratory AE considered possibly, probably or definitely related within 7 days after vaccination and persisting continuously at Grade 3 for > 72hrs.

- **Systemic solicited adverse events:**
  - the volunteer develops a Grade 3 systemic solicited AE considered possibly, probably or definitely related within 2 days after vaccination (day of vaccination and one subsequent day) and persisting continuously at Grade 3 for > 72hrs.

- **Unsolicited adverse events:**
  - the volunteer has a Grade 3 adverse event, considered possibly, probably or definitely related to vaccination, persisting continuously at Grade 3 for >72hrs.
  - the volunteer has a SAE considered possibly, probably or definitely related to vaccination.
  - the volunteer has an acute allergic reaction or anaphylactic shock following the administration of vaccine investigational product.

If a volunteer has an acute respiratory illness (moderate or severe illness with or without fever) or a fever (oral temperature greater than 37.8°C) at the scheduled time of administration of investigational product/control, the volunteer will not be enrolled and will be withdrawn from the study.

All vaccinated volunteers will be followed for safety until the end of their planned participation in the study or until resolution or stabilisation (if determined to be chronic sequelae) of their AEs, providing they consent to this.

Participants who met individual holding rules or were advised not to receive a booster dose as a result of an AE and have been offered a COVID-19 approved/licensed vaccine will be given an opportunity to discuss potential safety implications if they were to accept the offered vaccine with clinically qualified investigators.

In addition to these pre-defined criteria, the study can be put on hold upon advice of the DSMB, Chief Investigator, Study Sponsor, regulatory authority, Ethical Committee(s), for any single event or combination of multiple events which, in their professional opinion, jeopardise the safety of the volunteers or the reliability of the data.

10 STATISTICS

10.1 Description of Statistical Methods

Both a fully detailed study level statistical analysis plan (SAP) as well as a separate Statistical Analysis Plan for the Marketing Authorisation Application (MAA SAP) will be written and signed off before any interim data analyses are conducted.

The data from this study will be included in prospective pooled analyses of studies for efficacy and safety of ChAdOx1 nCoV-19 to provide greater precision of both efficacy and safety outcomes.

10.1.1 Efficacy Outcomes

The primary efficacy endpoint is PCR* positive symptomatic COVID-19.

This is defined as a participant with a PCR+* swab and at least one of the following symptoms: cough, fever ≥ 37.8, shortness of breath, anosmia, or ageusia.

Where possible, sensitivity analyses will be conducted using common alternative definitions of virologically-confirmed COVID-19 disease, including those in use in other phase 3 protocols (including but not limited to: USA AstraZeneca phase 3 trial, South Africa COV005 trial, WHO solidarity trial, CEPI definition). This will aid in comparisons between various studies and meta-analyses. These alternative definitions will be detailed in the statistical analysis plan as exploratory analyses.

* Or other nucleic acid amplification test

10.2 Primary efficacy

The primary and secondary analyses will be conducted on participants who are seronegative at baseline. A sensitivity analysis will be conducted including all participants regardless of baseline serostatus.

Analysis of the primary endpoint will be computed as follows:

1. **Efficacy of two doses of vaccine.** Only cases occurring more than 14 days after the second vaccine will be included.

Secondary analysis
2. **Efficacy of at least one standard-dose** of any ChAdOx1 nCoV-19. Cases occurring more than 21 days after the first vaccination

Proportions will be compared between ChAdOx1 nCoV-19 and MenACWY groups using a Poisson regression model with robust variance [17]. The model will contain terms including treatment group, and age group at randomization if there is a sufficient sample size within each age category. The logarithm of the period at risk for primary endpoint will be used as an offset variable in the model to adjust for volunteers having different follow up times during which the events occur. Vaccine efficacy (VE) will be calculated as (1 – RR) x 100%, where RR is the relative risk of symptomatic infection (ChADOx1 nCoV-19: Control) and 95% confidence intervals will be presented.

If the Poisson regression model with robust variance fails to converge, the exact conditional method for stratified poisson regression will be used.

Cumulative incidence of symptomatic infections will be presented using the Kaplan-Meier method.

Secondary efficacy endpoints will be analysed in the same way as the primary efficacy endpoint.

Analyses will conducted for all adults combined as well as conducting analyses stratified by age cohorts.

All data from participants with PCR*-positive swabs will be assessed for inclusion in the efficacy analyses by two blinded assessors who will independently review each case according to pre-specified criteria as detailed in the statistical analysis plan, to classify each for inclusion in the primary and secondary outcomes. A separate CRF will be designed for this purpose.

All PCR*-positive results will be assessed for the primary outcome, including those with symptoms swabbed by trial staff and other potential sources of information such as health-care workers who are tested at their workplace as either a routine test procedure or due to developing symptoms.

PCR+* swabs from outside the trial (for example, a workplace routine swab result in a healthcare worker) will be reviewed by blinded staff and only included as a potential endpoint if the test was conducted in 1) a medical laboratory with ISO 15189 accreditation (provided by UKAS in UK) AND 2) an assay that is either CE marked or that has a derogation authorisation from the MHRA.

* Or other nucleic acid amplification test

10.3 Safety & Reactogenicity

Counts and percentages of each local and systemic solicited adverse reaction from diary cards, and all unsolicited AEs and SAEs will be presented for each group.

10.4 Immunogenicity

Highly skewed antibody data will be log-transformed prior to analysis. The geometric mean concentration and associated 95% confidence interval will be summarised for each group at each timepoint, by computing the anti-log of the mean difference of the log-transformed data.

The geometric mean concentration at day 28 and the proportion of participants seroconverting to the S-spike protein from day 0 to day 28 will be computed. Comparisons between ChAdOx1 nCoV-19 vaccine and MenACWY groups will be made using a Mann Whitney U test due to the low titres expected in the control group which will cause a non-normal distribution.

Spike-specific T cell responses (ELISpot) will be presented as means and confidence intervals, or medians and interquartile ranges if non-normally distributed at all post vaccination time points. Comparisons between ChAdOx1 nCoV-19 vaccine and MenACWY groups will be made using a Mann Whitney U test due to the low responses expected in the control group which will cause a non-normal distribution.

Exploratory immunogenicity of booster doses – groups 1c, 1d, 5a and 5b

Antibody responses in those who received a third dose boost (3b and 5a), a late boost (1c) or those in the control groups combined (1d and 5b) will be summarised descriptively at each timepoint. Exploratory comparisons with groups boosted at other timepoints (day 28 and day 56) may also be presented.

10.5 Subgroup analyses

Subgroup comparisons of efficacy, and safety will be conducted by incorporating vaccine-group by subgroup interaction terms into appropriate regression models. Subgroup comparisons will only be conducted if there are at least 5 cases in all subgroups.

Comparisons will include:

1. Males vs females

2. Age (18 to 55 years vs 56-<70 years vs 70+ years)

3. Seropositive to S-spike or non-spike proteins at baseline vs not seropositive

4. Health care workers and highly-exposed participants versus others

10.6 Interim and primary analyses of the primary outcome

It is planned that the primary evidence of efficacy and safety for the ChADOx1 nCoV-19 vaccine will be based on global analyses utilizing studies COV001 (the UK P1/2 study), COV002 (the UK P2/3 study), COV003 (the Brazil P3 study) and COV005 (the South Africa P1/2 study) including a pooled analysis across the studies. As such the interim and primary analyses for the primary outcome will be based on cases accumulated across multiple studies, details of which will be specified within the MAA SAP rather than for each individual study. Interim and primary data cuts from this study will therefore be carried out to support the pooled analysis.

The global MAA SAP allows for interim and primary analyses to be conducted once sufficient eligible cases have accumulated, where the overall type 1 error is controlled at the 5% level using a flexible alpha–spending approach that accounts for the incorporation of data from this study into pooled interim analyses under the global MAA SAP.

Evidence of efficacy will be determined if the lower bound of the multiplicity adjusted confidence interval is greater than a 20% threshold. The primary analysis will have approximately 90% power assuming a vaccine efficacy of 60%. A flexible alpha spending approach will be implemented to allow an earlier primary analysis in the situation where accumulation of eligible cases were lower than expected.

Evidence of efficacy at an interim or primary analysis of pooled data will not be considered a reason to stop the trial, but instead will be interpreted as early evidence of efficacy. However if an interim analysis demonstrates evidence of efficacy then a study level analysis according to the study SAP may be used to support study level evidence of efficacy.

Participants who received other vaccines during the trial will have their vaccination data recorded. If a participant receives an approved COVID-19 vaccine outside of the trial procedures (such as through their workplace) this information will be recorded. Participants will be censored in the analysis at the time of their vaccination with the alternative COVID-19 vaccine.

If a large proportion of trial volunteers are vaccinated in this way then an exploratory, non-randomised analysis of efficacy according to vaccine(s) received will be undertaken if data are sufficient.

10.7 Final Analysis

A final analysis will be conducted at the end of the study. The final study-specific analysis will incorporate all data from the study, including data that has previously contributed to global efficacy estimates under the pooled analysis strategy. The final analysis will be considered a supportive analysis to the global efficacy analysis. Alpha at the final study-specific analysis will be adjusted to incorporate the number of previous global analyses to which the study contributed data in order to control the overall study level type 1 error at 5%. Details will be specified in the study level SAP.

10.8 Procedure for Accounting for Missing, Unused, and Spurious Data.

All available data will be included in the analysis

10.9 Inclusion in Analysis

All vaccinated participants will be included in the analysis unless otherwise specified in the SAP.

10.10 Interim analysis for the combined DSMB

The independent DSMB will meet regularly to review safety data from all available studies of ChAdOx1 nCoV-19 and will assess whether the assumptions underlying the sample size calculation are in line with the observed cases. Additionally the independent DSMB will make recommendations based on the interim analyses to assess evidence of efficacy.

11 DATA MANAGEMENT

11.1 Data Handling

The Chief Investigator will be responsible for all data that accrues from the study.

All study data including participant diary will be recorded directly into an Electronic Data Capture (EDC) system (e.g. OpenClinica, REDCap, or similar) or onto a paper source document for later entry into EDC if direct entry is not available or is not practical at site. This includes safety data, laboratory data and outcome data. Any additional information that needs recording but is not relevant for the CRF (such as signed consent forms etc.) will be recorded on a separate paper source document. All documents will be stored safely and securely in confidential conditions.

All adverse event data (both solicited and unsolicited) reported by the volunteer will be entered onto a volunteer's electronic diary card (eDiary) for a maximum of 28 days following administration of the IMP. The eDiary provides a full audit trail of edits and will be reviewed at each review time-point indicated in the schedule of events. Any adverse event continuing beyond the period of the diary will be copied into the eCRF and followed to resolution, if there is a causal relationship to the IMP, or to the end of the study if there is no causal relationship.

The participants will be identified by a unique trial specific number and code in any database. The name and any other identifying detail will NOT be included in any trial data electronic file.

The EDC system (CRF data) uses a relational database (MySQL/ PostgreSQL) via a secure web interface with data checks applied during data entry to ensure data quality. The database includes a complete suite of features which are compliant with GCP, EU and UK regulations and Sponsor security policies, including a full audit trail, user-based privileges, and integration with the institutional LDAP server. The MySQL and PostgreSQL database and the webserver will both be housed on secure servers maintained by the University of Oxford IT personal. The servers are in a physically secure location in Europe. Backups will be stored in accordance with the IT department schedule daily, weekly, monthly, and are retained for one month, three months, and six months, respectively. The IT servers provide a stable, secure, well-maintained, and high capacity data storage environment. REDCap and OpenClinica are widely-used, powerful, reliable, well-supported systems. Access to the study's database will be restricted to the members of the study team by username and password.

11.2 Record Keeping

The Investigators will maintain appropriate medical and research records for this trial, in compliance with GCP and regulatory and institutional requirements for the protection of confidentiality of volunteers. The Chief

Investigator, co-Investigators and clinical research nurses will have access to records. The Investigators will permit authorised representatives of the Sponsor(s), as well as ethical and regulatory agencies to examine (and when required by applicable law, to copy) clinical records for the purposes of quality assurance reviews, audits and evaluation of the study safety and progress.

All trial records will be stored for a minimum of 5 years after the end of the trial at a secure archiving facility . If volunteers consent to be contacted for future research, information about their consent form will be recorded, retained and stored securely and separately from the research data. If volunteers consent to have their samples stored and used in future research, information about their consent form will be recorded, retained and stored securely as per Biobanking procedures and SOP.

11.3 Source Data and Case Report Forms (CRFs)

All protocol-required information will be collected in CRFs designed by the Investigator. All source documents will be filed in the CRF. Source documents are original documents, data, and records from which the volunteer's CRF data are obtained. For this study, these will include, but are not limited to, volunteer consent form, blood results, GP response letters, laboratory records, diaries, medical records and correspondence. In the majority of cases, CRF entries will be considered source data as the CRF is the site of the original recording (i.e. there is no other written or electronic record of data). In this study this will include, but is not limited to medical history, medication records, vital signs, physical examination records, urine assessments, blood results, adverse event data and details of vaccinations. All source data and volunteer CRFs will be stored securely.

Source data verification requirements will be defined in the trial risk assessment and monitoring plan.

To prevent withdrawal of a participant due to relocation, if there is a nearby participating site and with the consent of the participant, copies of relevant participant research records (such as ICF, paper source documents) will be transferred to the local site using secure email addresses such as nhs.net or by password protected sheets. The electronic research data stored on REDCap will also be transferred to the new site. The original records will be retained by the recruiting site. Consent to transfer information could be completed electronically.

11.4 Data Protection

The study protocol, documentation, data and all other information generated will be held in strict confidence. No information concerning the study or the data will be released to any unauthorised third party, without prior written approval of the sponsor.

11.5 Data Quality

Data collection tools will undergo appropriate validation to ensure that data are collected accurately and completely. Datasets provided for analysis will be subject to quality control processes to ensure analysed data is a true reflection of the source data.

Trial data will be managed in compliance with local data management SOPs. If additional, study specific processes are required, an approved Data Management Plan will be implemented

11.6 Archiving

Study data may be stored electronically on a secure server, and paper notes will be kept in a key-locked filing cabinet at the site. All essential documents will be retained for a minimum of 5 years after the study has finished. The need to store study data for longer in relation to licensing of the vaccine will be subject to ongoing review. For effective vaccines that may be licensed, we may store research data securely at the site at least 15 years after the end of the study, subject to adjustments in clinical trials regulations. Participants' bank details will be stored for 7 years in line with the site financial policy.

General archiving procedures will be conducted in compliance to SOP OVC020 Archiving.

12 QUALITY CONTROL AND QUALITY ASSURANCE PROCEDURES

12.1 Investigator procedures

Approved site-specific standard operating procedures (SOPs) will be used at all clinical and laboratory sites.

12.2 Monitoring

Regular monitoring will be performed according to GCP by the monitor. Following written SOPs and an approved, risk based monitoring plan, the monitor will verify that the clinical trial is conducted and data are generated, documented and reported in compliance with the protocol, GCP and the applicable regulatory requirements. The site will provide direct access to all trial related source data/documents and reports for the purpose of monitoring and auditing by the Sponsor and inspection by local and regulatory authorities.

12.3 Protocol deviation

Any deviations from the protocol will be documented in a protocol deviation form and filed in the trial master file. Each deviation will be assessed as to its impact on volunteer safety and study conduct. Significant protocol deviations will be listed in the end of study report.

12.4 Audit & inspection

The QA manager conducts systems based internal audits to check that trials are being conducted according to local procedures and in compliance with study protocols, departmental SOPs, GCP and applicable regulations.

The Sponsor, trial sites, and ethical committee(s) may carry out audit to ensure compliance with the protocol, GCP and appropriate regulations.

GCP inspections may also be undertaken by the MHRA to ensure compliance with protocol and the Medicines for Human Use (Clinical Trials) Regulations 2004, as amended. The Sponsor will assist in any inspections and will support the response to the MHRA as part of the inspection procedure.

13 SERIOUS BREACHES

The Medicines for Human Use (Clinical Trials) Regulations contain a requirement for the notification of "serious breaches" to the MHRA within 7 days of the Sponsor becoming aware of the breach.

A serious breach is defined as “A breach of GCP or the trial protocol which is likely to effect to a significant degree

(a) the safety or physical or mental integrity of the subjects of the trial; or

(b) the scientific value of the trial”.

In the event that a potential serious breach is suspected the Sponsor will be informed as soon as possible, to allow preliminary assessment of the breach and reporting to the MHRA within the required timelines.

14 ETHICS AND REGULATORY CONSIDERATIONS

14.1 Declaration of Helsinki

The Investigators will ensure that this study is conducted according to the principles of the current revision of the Declaration of Helsinki.

14.2 Guidelines for Good Clinical Practice

The Investigator will ensure that this trial is conducted in accordance with relevant regulations and with Good Clinical Practice.

14.3 Ethical and Regulatory Approvals

Following Sponsor approval the protocol, informed consent form, participant information sheet and any proposed advertising material will be submitted to an appropriate Research Ethics Committee (REC), HRA (where required), regulatory authorities (MHRA in the UK), and host institution(s) for written approval. No amendments to this protocol will be made without consultation with, and agreement of, the Sponsor.

The Investigator is responsible for ensuring that changes to an approved trial, during the period for which regulatory and ethical committee(s) approval has already been given, are not initiated without regulatory and ethical committee(s)' review and approval except to eliminate apparent immediate hazards to the subject (i.e as an Urgent Safety Measure).

14.4 Volunteer Confidentiality

The study will comply with the General Data Protection Regulation (GDPR) and Data Protection Act 2018, which require data to be de-identified as soon as it is practical to do so. The processing of personal data of participants will be minimised by making use of a unique participant study number only on all study documents and any electronic database(s), with the exception of informed consent forms and participant ID logs. All documents will be stored securely and only accessible by study staff and authorised personnel. The study staff will safeguard the privacy of participants' personal data. A separate confidential file containing identifiable information will be stored in a secured location in accordance with the current data protection legislation. Photographs taken of vaccination sites (if required, with the volunteer's written, informed consent) will not include the volunteer's face and will be identified by the date, trial code and subject's unique identifier. Once developed, photographs will be stored as confidential records, as above. This material may be shown to other professional staff, used for educational purposes, or included in a scientific publication.

If participants have a positive swab result for COVID-19 during the course of the study then the Public Health Authority will be notified as COVID-19 is a "notifiable disease" and this is legal requirement in the UK. This

may mean participants personal information from their health records will be shared with Public Health either by the processing lab or the study site. Participants may also be contacted by the NHS Test and Trace service.

Samples collected using home swab kits may be processed at laboratories within and outside the UK, as determined by the community testing programme. These laboratories provide a test result for the barcode to NPEx (National Pathology Exchange) and this result is then recombined with participant identifiable information by NHS Digital. NHS Digital provide lab results to the Sponsor (University of Oxford) who will match this with personal data including identifying contact information sent to them by the site in order to centralise the processing of results.

Participants will be required to separately consent to the terms and conditions of the national community swabbing programme, each time they perform a self-swab. This is available at: https://www.gov.uk/government/publications/coronavirus-covid-19-testing-privacy-information/testing-for-coronavirus-privacy-information

15 FINANCING AND INSURANCE

15.1 Financing

The study is funded through UK Government

15.2 Insurance

The University has a specialist insurance policy in place which would operate in the event of any participant suffering harm as a result of their involvement in the research (Newline Underwriting Management Ltd, at Lloyd's of London). NHS indemnity operates in respect of the clinical treatment which is provided.

15.3 Compensation

Volunteers will be compensated for their time, the inconvenience of having blood tests and procedures, and their travel expenses. The total amount compensated will be approximately **£190-625** depending on the exact number of visits, and whether any repeat or additional visits are necessary. They will be compensated £25 for attending the screening visit. For all other trial visits as outlined in Tables 6-8, compensation will be calculated according to the following:

- Travel expenses: £15 per visit
- Inconvenience of blood tests: £10 per blood donation
- Time required for visit: £20 per hour

Should the volunteer decide to withdraw from the trial before it is completed, payment will be pro rata

16 Publication Policy

The Investigators will be involved in reviewing drafts of the manuscripts, abstracts, press releases and any other publications arising from the study. Data from the study may also be used as part of a thesis for a PhD or MD.

17 DEVELOPMENT OF A NEW PRODUCT/PROCESS OR THE GENERATION OF INTELLECTUAL PROPERTY

Ownership of IP generated by employees of the University vests in the University. The protection and exploitation of any new IP is managed by the University's technology transfer office, Oxford University Innovations. Investigators in this study may benefit from the royalty sharing policy of the University if new intellectual property is generated from the trial. Several investigators are applicants or co-inventors on previous patent filings or patents related to ChAdOx1 vaccines. The University of Oxford, which is partnered with the Oxford University Hospitals NHS Foundation Trust in the NIHR Oxford Biomedical Research Centre, is committed to the translational progress and commercial development of healthcare products potentially meeting medical and global health needs, and does and will work with commercial partners towards these goals.

18. References

1. Zhu, N., et al., *A Novel Coronavirus from Patients with Pneumonia in China, 2019.* New England Journal of Medicine, 2020. **382**(8): p. 727-733.
2. Lu, R., et al., *Genomic characterisation and epidemiology of 2019 novel coronavirus: implications for virus origins and receptor binding.* Lancet (London, England), 2020. **395**(10224): p. 565-574.
3. Li, F., *Structure, Function, and Evolution of Coronavirus Spike Proteins.* Annual review of virology, 2016. **3**(1): p. 237-261.
4. Zhou, P., et al., *A pneumonia outbreak associated with a new coronavirus of probable bat origin.* Nature, 2020.
5. Alharbi, N.K., et al., *ChAdOx1 and MVA based vaccine candidates against MERS-CoV elicit neutralising antibodies and cellular immune responses in mice.* Vaccine, 2017. **35**(30): p. 3780-3788.
6. Liu, L., et al., *Anti–spike IgG causes severe acute lung injury by skewing macrophage responses during acute SARS-CoV infection.* JCI insight, 2019. **4**(4).
7. Tseng, C.T., et al., *Immunization with SARS coronavirus vaccines leads to pulmonary immunopathology on challenge with the SARS virus.* PLoS One, 2012. **7**(4): p. e35421.
8. Weingartl, H., et al., *Immunization with modified vaccinia virus Ankara-based recombinant vaccine against severe acute respiratory syndrome is associated with enhanced hepatitis in ferrets.* J Virol, 2004. **78**(22): p. 12672-6.
9. Agrawal, A.S., et al., *Immunization with inactivated Middle East Respiratory Syndrome coronavirus vaccine leads to lung immunopathology on challenge with live virus.* Hum Vaccin Immunother, 2016. **12**(9): p. 2351-6.
10. Alharbi, N.K., et al., *Humoral Immunogenicity and Efficacy of a Single Dose of ChAdOx1 MERS Vaccine Candidate in Dromedary Camels.* Scientific reports, 2019. **9**(1): p. 1-11.
11. Munster, V.J., et al., *Protective efficacy of a novel simian adenovirus vaccine against lethal MERS-CoV challenge in a transgenic human DPP4 mouse model.* npj Vaccines, 2017. **2**(1): p. 1-4.
12. Antrobus, R.D., et al., *Clinical assessment of a novel recombinant simian adenovirus ChAdOx1 as a vectored vaccine expressing conserved Influenza A antigens.* Mol Ther, 2014. **22**(3): p. 668-74.
13. Coughlan, L., et al., *Heterologous Two-Dose Vaccination with Simian Adenovirus and Poxvirus Vectors Elicits Long-Lasting Cellular Immunity to Influenza Virus A in Healthy Adults.* EBioMedicine, 2018.
14. Wilkie, M., et al., *A phase I trial evaluating the safety and immunogenicity of a candidate tuberculosis vaccination regimen, ChAdOx1 85A prime - MVA85A boost in healthy UK adults.* Vaccine, 2020. **38**(4): p. 779-789.
15. Folegatti, P.M., et al., *Safety and immunogenicity of a candidate Middle East respiratory syndrome coronavirus viral-vectored vaccine: a dose-escalation, open-label, non-randomised, uncontrolled, phase 1 trial.* The Lancet Infectious Diseases, 2020.
16. Modjarrad, K., *MERS-CoV vaccine candidates in development: The current landscape.* Vaccine, 2016. **34**(26): p. 2982-7.
17. Zou, G., *A modified poisson regression approach to prospective studies with binary data.* Am J Epidemiol, 2004. **159**(7): p. 702-6.

APPENDIX A: AMENDMENT HISTORY

Amendment No.	Protocol Version No.	Date issued	Author(s) of changes	Details of Changes made
N/A	1.0	13 Mar 2020	Pedro Folegatti, Daniel Jenkin, Sarah Gilbert, Andrew Pollard, Adrian Hill, Merryn Voysey	N/A
N/A	2.0	18 Mar 2020	Pedro Folegatti	Neutralising antibodies listed as exploratory endpoints instead of secondary ones; Added blood samples for safety and immunogenicity at COVID-19 testing visit, in addition to nose/throat swabs; Day 2 visit replaced with Day 3; Replaced references to Oxford site with 'the site'. COI statement updated for Prof Adrian Hill
SA01	3.0	23 Mar 2020	Pedro Folegatti	Replaced saline placebo with active comparator (MenACWY). Added Dr Maheshi Ramasamy and Prof Matthew Snape as investigators; Expanded list of exclusion criteria, adding all vulnerable groups considered at risk of severe COVID-19 disease; Changed procedures for swabbing volunteers when symptomatic to include ECDC case definition; Removed reference to separate consent procedure for Biobank (this is already covered in the consent form); Increased blood volumes for exploratory immunology at D3 from 10 to 50mL; Added COVID-19 Testing +7 visit; Added PAXgenes; Consent to be taken by

Amendment No.	Protocol Version No.	Date issued	Author(s) of changes	Details of Changes made
				both clinicians and appropriately trained and delegated research nurses when required.
SA02	4.0	20 Apr 2020	Pedro Folegatti, Merryn Voysey, Emma Plested	Increased sample size with added group 4; Added phone/video call review at D1 for groups 1 and 3; Added phone/video call review as required during COVID-19 follow-up; updated statistical analysis section; clarifications to exclusion criteria; expanded list of AESI and instruction to report eosinophilia greater than 1.5x10^9/L as SAE. Addition of sites. Addition of monitoring roles..; a reduced dose has been introduced for the booster doses in group 3 (2.5x10^10 vp) as this will allow for an assessment of dose sparing, optimising the available vials; re-consent to be taken by appropriately trained and delegated members of the team; added household weekly questionnaires as a measure of exposure to COVID-19; added lab AE grading scale as an appendix to the protocol.
SA03	5.0	21 Apr 2020	Pedro Folegatti	Clarification to screening procedures for high risk COVID-19 exposure participants.
SA05	6.0	06 May 2020	Pedro Folegatti, Merryn Voysey	Added Dr Angela Minassian as an investigator; Added prophylactic paracetamol use to a subset of participants in group 4; Added an exploratory objective to describe safety, reactogenicity, immunogenicity and efficacy amongst those receiving paracetamol for 24h; Clarifications to IMP

Amendment No.	Protocol Version No.	Date issued	Author(s) of changes	Details of Changes made
				storage locations; Updated sample size to reflect the actual number of vials available to run the study.; Corrected lab AE stopping rule to reflect timing of safety bloods taken.
SA06	7.0	19 May 2020	Pedro Folegatti	Booster dose changed back to $5x10^{10}$vp for consistency with COV002; clarification that holding rules apply to ChAdOx1 nCoV-19 only and not the licensed comparator; clarification to notification of DSMB chair on SAEs deemed at least possibly related which should happen within 24h of the Sponsor becoming aware of the event; additional steps taken to avoid unblinding of participants when attending for COVID-19 swabbing visits; Confirmation that additional steps may be taken to manage the blinding of staff when assessing endpoints or managing the unblinding of staff when managing safety reporting; correction of formatting and typographical errors
SA08	8.0	22 Jun 2020	Pedro Folegatti	Added booster vaccination on a subset of participants enrolled in Group 2.
SA09	9.0	30 Jul 2020	Pedro Folegatti, Maheshi Ramasamy, Merryn Voysey, Hannah Robinson	Added booster vaccination on all participants in Groups 2 and 4; Added Hy's law criteria to be reported as SAE; Clarifications to holding rules; Added information on Cobra material to be used in booster doses; added stool samples on PCR positive participants, added an observation time for post booster vaccinations; changed the D364 visit from

**Amendment No.**	**Protocol Version No.**	**Date issued**	**Author(s) of changes**	**Details of Changes made**
				optional.; changed swabbing pathway (S7 to be conducted only on positive cases, added S3-5 visit for second swab or home testing); updated statistical analysis section; Re-consent may be collected with electronic signatures if required for infection control purposes; an update to the 'Planned receipt of any vaccine other than the study intervention within 30 days before and after each study vaccination' exclusion criteria to allow an exception for the seasonal flu vaccine; clarifications made to visit time points.
SA12	10	16 Sep 2020	Pedro Folegatti, Merryn Voysey	Clarification to efficacy objectives to include efficacy against severe disease; Clarification to exclusion criteria where only licensed seasonal influenza vaccines will be allowed within 7 days of vaccine administration; Clarification to requirements for contraception (at least 3 months post last vaccination); Clarifications to the statistical analysis section on primary, secondary and exploratory analysis; Clarifications to symptomatic pathway; Correction of formatting and typographical errors; Addition of transfer possibility if participants are relocating to an area with a participating site; Update to the vaccine expiry time
SA14	11	21 Oct 2020	Hannah Robinson	Exclusion criteria updated to allow administration of licensed pneumococcal vaccines within 7 days of study vaccine administration; Clarification that home

Amendment No.	Protocol Version No.	Date issued	Author(s) of changes	Details of Changes made
				swabs may be processed outside of the UK; Clarification of the flow of information from home swabbing results to sponsor; Correction of blood sample volumes in table 11; statistician inserted, statistical 'section 10' updated.
SA15	12	13 Nov 2020	Maheshi Ramasamy	Clarification that nucleic acid amplification assays (NAAT) will be used to confirm viral infection; Typographical correction to amendment reference for V11.0 protocol change summary
SA16	13.1	11 Dec 2020	Hannah Robinson Pedro Folegatti<br>Emma Plested	Addition of >Grade 2 neurological events as AESI.<br>Addition of plan and management for unblinding of participants to allow administration of a SARS-CoV-2 vaccine according to government guidance.<br>Those who received an approved or licensed COVID-19 will not be withdrawn from the study and will be followed-up as per their schedule of attendances.<br>Exploratory, non-randomised analysis of efficacy according to vaccine(s) received will be undertaken if data are sufficient on other COVID-19 vaccines.<br>Extra visits A & B included for collection of samples or administration of additional study vaccine following early unblinding.<br>Added the 'extra visit A' and' Extra visit B' in the schedule of events<br>Safety section updated to consider assessment of causality of deployed vaccines.

Amendment No.	Protocol Version No.	Date issued	Author(s) of changes	Details of Changes made
SA19	14.0		Hannah Robinson Pedro Folegatti	Only AESI and SAEs will be collected following Extra Visit B and vaccination as part of the NHS roll out<br>Clarification that visits can be conducted over the phone for pregnant participants and those unable to attend in person<br>Outline discontinuation processes that prevent withdrawal<br>Change in IMP storage instructions to use within 48 hours of vial piercing<br>Holding rules will no longer apply following approval for emergency use by the UK regulator and subsequent national roll-out.
SA20	15.0		Daniel Jenkin	Changes to symptomatic participant follow up:<br>If negative NAAT test outside of the trial during a symptomatic episode then no need to bring participant in for repeat swab<br>If positive NAAT test outside + symptoms then still bring in for repeat swab and immunology bloods (no safety bloods, observations or examination needed)<br>No repeat home swab (A single negative NAAT test in or out of the trial is now sufficient to declare it a negative case)<br>Replace S7 physical visit with phone call<br>Removal of symptomatic ediary

Amendment No.	Protocol Version No.	Date issued	Author(s) of changes	Details of Changes made
				Removal of S3-5 repeat swab/visit<br><br>Addition of late vac groups:<br><br>Exploratory objective added to assess immunogenicity of a delayed prime-boost and three dose ChAdOx1 nCoV-19 regimen<br><br>Group 1c (delayed homologous prime-boost of group 1a)<br><br>Group 4e (delayed 3rd ChAdOx1 dose)<br><br>Group 1b and 4f (ChAdOx1 prime boost to enable comparison of prime with delayed prime boost and delayed 3rd dose groups)<br><br>7 day local and systemic AE follow up following late vacc visit<br><br>Prophylactic paracetamol will not be advised at the late vaccination visits<br><br>Miscellanous<br><br>Blood donation now permitted 3 months following receipt of final planned study vaccine<br><br>Update to extra visit B vaccination for pregnancy: Vaccination now permissible at extra visit B if participant has discussed with her usual clinician (e.g. GP) and chosen to receive a covid-19 vaccination<br><br>Details of menstrual cycle will be collected prior to receipt of boost vaccination for female participants in group 1c, 1d, 4e and 4f<br><br>Clarification that a letter will be sent to the participant and their GP detailing the

Amendment No.	Protocol Version No.	Date issued	Author(s) of changes	Details of Changes made
				vaccinations received in the study, once participant has been unblinded.
SA21	V16.0		Daniel Jenkin	Groups 4e and 4f now called group 5a and 5b. These groups will now consist of volunteers previously in 2c, 2f, 4c, 2e, 2g and 4d to draw from a pool of volunteers that more closely match the 4-12 week MHRA approved schedule. Group 3 also to receive a third dose for the same reason.<br><br>SAE collection period defined: (For "late vaccination groups" SAEs will be actively solicited for 6 months from the first "late vaccination". Otherwise SAEs will be actively solicited up to 01/07/2021.)<br><br>Primary safety endpoint amended to reflect SAE active collection period cutoff date
SA22	V17.0		Daniel Jenkin	Unblinding and provision of ChAdOx1 nCoV-19 to all participants<br><br>Removal of 1 hour post prime and 15 minute post boost observation period<br><br>Contraception advice following vaccination now harmonised with UK Greenbook guidance<br><br>Immunological correlate of protection endpoint added
SA23	V18.0		Parvinder Aley	Update to risks and AESIs sections regarding the MHRA & JCVI recommendations regarding reports of thrombosis and thrombocytopenia.

**Amendment No.**	**Protocol Version No.**	**Date issued**	**Author(s) of changes**	**Details of Changes made**
				Clarification that unblinded exploratory COVID-19 disease endpoints may be determined by trained trial team members (rather than blinded independent endpoint assessors)

List details of all protocol amendments here whenever a new version of the protocol is produced.

Appendix. Toxicity grading scale for Lab AEs

**Haematology**			**Lab Range**	**Grade 1**	**Grade 2**	**Grade 3**	**Grade 4**
Haemoglobin Absolute	**Male**	g/l	130 - 170	115-125	100-114	85-99	<85
Haemoglobin Absolute	**Female**		120 - 150	105-113	90-104	80-89	<80
Haemoglobin Change from Baseline (Decrease)			n/a	10-15	16-20	21-50	>50
White Blood Cells	**Elevated**	x109/l	11	11.5-15.00	15.01-20	20.01-25	>25
White Blood Cells	**Low**		4.0	2.5-3.5	1.5-2.49	1.0-1.49	<1.0
Platelets	**Low**		150-400	125-140	100-124	25-99	<25
Neutrophils	**Low**		2.0-7.0	1.5-1.99	1.0-1.49	0.5-0.99	<0.50
Lymphocytes	**Low**		1.0-4.0	0.75-0.99	0.5-0.74	0.25-0.49	<0.25
Eosinophils	**Elevated**	x109/l	0.02 - 0.5	0.65-1.5	1.51-5.00	>5.00	Hypereosinophilia
**Biochemistry**							
Sodium	**Elevated**	mmol/l	145	146-147	148-149	150-155	>155
Sodium	**Low**		135	132-134	130-131	125-129	<125
Potassium	**Elevated**	mmol/l	5	5.1-5.2	5.3-5.4	5.5-6.5	>6.5
Potassium	**Low**		3.5	3.2-3.3	3.1	2.5-3.0	<2.5
Urea	**Elevated**	mmol/l	2.5 - 7.4	8.2-9.3	9.4-11.0	>11.0	Requires dialysis
Creatinine	**Elevated**	µmol/l	49 - 104	1.1-1.5xULN<br>114-156	>1.5-3.0xULN<br>157-312	>3.0xULN<br>>312	Requires dialysis
Bilirubin	**Normal LFTs**	µmol/l	0-21	1.1-1.5xULN<br>23-32	>1.5-2xULN<br>33-42	>2-3xULN<br>43-63	>3xULN<br>≥64
Bilirubin	**Abnormal LFTs**	µmol/l	0 - 21	1.1-1.25xULN<br>23-26	>1.25-1.5xULN<br>27-32	>1.5-1.75xULN<br>33-37	>1.75xULN<br>>37
ALT		IU/l	10 - 45	1.1-2.5xULN<br>49-112	>2.-5xULN<br>113-225	>5-10xULN<br>226-450	>10xUPN<br>>450
Alk Phosphatase	**Elevated**	IU/l	30 -130	1.1-2xULN<br>143-260	>2.-3xULN<br>261-390	>3-10xULN<br>391-1300	>10xULN<br>>1300
Albumin		g/l	32-50	28-31	25-27	<25	-

Normal lab ranges may vary between sites and should be adapted accordingly
