# Supplementary material for: Reactogenicity and immunogenicity after a late second dose or a third dose of ChAdOx1 nCoV-19 in the UK: a substudy of two randomised controlled trials (COV001 and COV002)
Source: Lancet. 2021 Sep 11;398(10304):981–90. doi: 10.1016/S0140-6736(21)01699-8 (PMC8409975; doi:10.1016/S0140-6736(21)01699-8)
Supplement: Supplementary appendix 2 [file mmc2.pdf]

THE LANCET

Supplementary appendix

This appendix formed part of the original submission and has been peer reviewed. We post it as supplied by the authors.

Supplement to: Flaxman A, Marchevsky NG, Jenkin D, et al. Reactogenicity and immunogenicity after a late second dose or a third dose of ChAdOx1 nCoV-19 in the UK: a substudy of two randomised controlled trials (COV001 and COV002). *Lancet* 2021; published online Sept 1. http://dx.doi.org/10.1016/S0140-6736(21)01699-8.

Trial Title: A phase 2/3 study to determine the efficacy, safety and immunogenicity of the candidate Coronavirus Disease (COVID-19) vaccine ChAdOx1 nCoV-19

Short title Investigating a Vaccine Against COVID-19

Study Reference: (COV002)

Protocol Version: 20.0

Date: 2 June 2021

EudraCT number: 2020-001228-32

REC Reference: **20/SC/0179**

IRAS Reference: 281904

Chief Investigator: Prof Andrew Pollard

Sponsor: University of Oxford

Funder: **NIHR**

Key Trial Contacts

**Chief Investigator**	Prof Andrew Pollard
**Address**	Centre for Clinical Vaccinology and Tropical Medicine<br>University of Oxford,<br>Churchill Hospital, Old Road, Headington<br>Oxford, OX3 7LE<br>Email: andrew.pollard@paediatrics.ox.ac.uk
**Clinical Trials Unit**	Primary Care and Vaccines Collaborative CTU
**Investigators**	Prof Brian Angus<br>Centre for Clinical Vaccinology and Tropical Medicine<br>Churchill Hospital, Old Road, Headington<br>Oxford, OX3 7LE<br>Email: brian.angus@ndm.ox.ac.uk<br><br>Prof Adrian Hill<br>The Jenner Institute, University of Oxford<br>Old Road Campus Research Building (ORCRB)<br>Roosevelt Drive Oxford OX3 7DQ<br>Email: adrian.hill@ndm.ox.ac.uk<br><br>Prof Sarah Gilbert<br>The Jenner Institute, University of Oxford<br>Old Road Campus Research Building (ORCRB)<br>Roosevelt Drive Oxford OX3 7DQ<br>Email: sarah.gilbert@ndm.ox.ac.uk<br><br>Dr Pedro Folegatti<br>Centre for Clinical Vaccinology and Tropical Medicine<br>Churchill Hospital, Old Road, Headington<br>Oxford, OX3 7LE<br>Email: pedro.folegatti@ndm.ox.ac.uk<br><br>Dr Alexander Douglas<br>The Jenner Institute<br>Wellcome Centre for Human Genetics<br>Roosevelt Drive<br>Oxford, OX3 7BN<br>Email: sandy.douglas@ndm.ox.ac.uk<br><br>Prof Matthew Snape<br>Oxford Vaccine Group<br>Centre for Clinical Vaccinology and Tropical Medicine<br>University of Oxford,<br>Churchill Hospital, Old Road, Headington<br>Oxford, OX3 7LE<br>Email : matthew.snape@paediatrics.ox.ac.uk

Dr Maheshi Ramasamy
Oxford Vaccine Group
Centre for Clinical Vaccinology and Tropical Medicine
University of Oxford,
Churchill Hospital, Old Road, Headington
Oxford, OX3 7LE
Email : maheshi.ramasamy@paediatrics.ox.ac.uk

Prof Teresa Lambe
The Jenner Institute, University of Oxford
Old Road Campus Research Building (ORCRB)
Roosevelt Drive Oxford OX3 7DQ
Email: teresa.lambe@ndm.ox.ac.uk

Prof Katie Ewer
The Jenner Institute, University of Oxford
Old Road Campus Research Building (ORCRB)
Roosevelt Drive Oxford OX3 7DQ
Email: katie.ewer@ndm.ox.ac.uk

Dr Angela Minassian
Centre for Clinical Vaccinology and Tropical Medicine
University of Oxford,
Churchill Hospital, Old Road, Headington
Oxford, OX3 7LE
Email : angela.minassian@ndm.ox.ac.uk

Dr Paola Cicconi
Centre for Clinical Vaccinology and Tropical Medicine
University of Oxford,
Churchill Hospital, Old Road, Headington
Oxford, OX3 7LE
Email: paola.cicconi@ndm.ox.ac.uk

Trial Sites

Centre for Clinical Vaccinology & Tropical Medicine
University of Oxford
Churchill Hospital, Old Road, Headington, Oxford, OX3 7LE
PI at Site: Professor Adrian Hill
Email: adrian.hill@ndm.ox.ac.uk

NIHR WTCRF
University Hospital Southampton NHS Foundation Trust
Southampton, SO16 6YD
PI at Site: Professor Saul Faust
Email: s.faust@soton.ac.uk

IHR Imperial CRF
NIHR Imperial Clinical Research Facility Imperial College
Hammersmith Hospital, Imperial College NHS Trust
150 Du Cane Road, London, W12 0HS
PI at Site: Dr Katrina M. Pollock
Email: k.pollock@imperial.ac.uk

St Georges University Hospital NHS Foundation Trust
Blackshaw Road, Tooting, London
SW17 0QT
PI at site: Professor Paul Heath
Email: pheath@sgul.ac.uk

University Hospitals Bristol and Weston NHS Foundation Trust
Marlborough Street, Bristol, Avon
BS1 3NU
PI at site: Professor Adam Finn
Email: Adam.Finn@bristol.ac.uk

North Bristol NHS Trust
Southmead Hospital
Southmead Road
Westbury-on-Trym
Bristol, BS10 5NB
PI at site: Dr Rajeka Lazarus
Email: rajeka.lazarus@uhbw.nhs.uk

Oxford University Hospital Foundation Trust
John Radcliffe Hospital
Headley Way, Headington
Oxford
OX3 9DU
PI at site: Dr Maheshi Ramasamy
Email: maheshi.ramasamy@paediatrics.ox.ac.uk

University of Nottingham Health Service
Cripps Health Centre, University Park
Nottingham, NG7 2QW
PI at site: Dr David Turner
Email: david.turner@nottingham.ac.uk

Nottingham University Hospitals NHS Trust
C Floor, South Block
Queen's Medical Centre Campus
Derby Road Nottingham NG7 2UH
PI at site: Dr David Turner
Email: david.turner@nottingham.ac.uk

Sheffield Teaching Hospitals NHS Foundation Trust
Royal Hallamshire Hospital, Glossop Road
Sheffield S10 2RX
PI at site: Dr. Thomas Darton
Email: t.darton@sheffield.ac.uk

University Hospitals Birmingham NHS Foundation Trust (UHB)
Queen Elizabeth Hospital Birmingham
Mindelsohn Way
Birmingham B15 2TH
PI at site: Dr Christopher Green
Email: cgreen16@nhs.net

Wales (Public Health Wales)
Aneurin Bevan Local Health Board of Aneurin Bevan Local Health Board Headquarters,
St. Cadoc's Hospital,
Lodge Road,
Caerleon,
Newport,
NP18 3XQPI at site: Dr Chris J Williams
Email: Christopher.williams25@wales.nhs.uk

Hull University Teaching Hospitals NHS Trust (HUTH).
Hull Royal Infirmary
Anlaby Road, Hull
HU3 2JZ
PI at site: Dr Patrick Lillie
Email: patrick.lillie@hey.nhs.uk

Greater Glasgow and Clyde NHS Board
NHS Greater Glasgow and Clyde Corporate HQ
J B Russell House
Gartnavel Royal Hospital Campus
1055 Great Western Road
GLASGOW
G12 0XH
PI at site: Professor Emma Thomson (QEUH)
Co Investigator: Prof Andrew Smith (GRI)
PI Email: Emma.Thomson@glasgow.ac.uk
Co-Investigator Email: Andrew.Smith@glasgow.ac.uk

Guy's and St Thomas' NHS Foundation Trust
Department of Infection, St Thomas Hospital
Westminster Bridge Road, London SE1 7EH
PI at site: Dr Anna Goodman

Email: Anna.goodman@gstt.nhs.uk

Liverpool School of Tropical Medicine (LSTM)
Liverpool School of Tropical Medicine
Pembroke Place
Liverpool L3 5QA
PI at site: Dr Andrea Collins
Email: Andrea.collins@lstmed.ac.uk

The Newcastle upon Tyne Hospitals NHS Foundation Trust
Royal Victoria Infirmary
Newcastle upon Tyne
NE1 4LP
PI at site: Dr Christopher Duncan
Email: christopherduncan@nhs.net

UCLH
250 Euston Road, London
NW1 2PG
PI at site: Prof Vincenzo Libri
Email: vincenzo.libri@ucl.ac.uk

Lothian NHS Board
Waverleygate
2-4 Waterloo Place
Edinburgh
EH1 3EGPI at site: Dr Rebecca Sutherland
Email: Rebecca.sutherland@nhslothian.scot.nhs.uk

Cambridge
Cambridge Clinical Research Facility
Cambridge Biomedical Campus, Hills Road
Cambridge CB2 0QQ
PI at site: Dr Mark Toshner
Email: mrt34@medschl.cam.ac.uk

Northwick Park Hospital
London North West University Healthcare Trust, Northwick Park Hospital, Watford Road, Harrow, HA1 3UJ.
PI at site: Dr Alastair McGregor
Email: alastair.mcgregor1@nhs.net

Sponsoring Institution

University of Oxford
Clinical Trials and Research Governance
Joint Research Office,
Boundary Brook House
Churchill Drive, Headington

Oxford OX3 7GB
Email: ctrg@admin.ox.ac.uk

Funder **NIHR**

Lead Monitor

Oxford Vaccine Centre
CCVTM
Churchill Hospital
Headington
OXON
OX3 7LE
Email: covid19@ndm.ox.ac.uk

DSMC Chair

Professor Robert Heyderman (Chair)
University College London

Statistician

Merryn Voysey
Oxford Vaccine Centre
CCVTM, Churchill Hospital
Headington, OXON
OX3 7LE
merryn.voysey@paediatrics.ox.ac.uk

Signature:

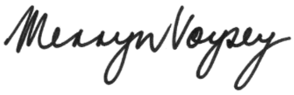

Confidentiality Statement

This document contains confidential information that must not be disclosed to anyone other than the Sponsor, the Investigator Team, HRA, host organisation, and members of the Research Ethics Committee and other regulatory bodies. This information cannot be used for any purpose other than the evaluation or conduct of the clinical investigation without the prior written consent of Prof Andrew Pollard.

Statement of Compliance

The trial will be conducted in compliance with the protocol, the principles of Good Clinical Practice, Medicines for Human Use (Clinical Trial) Regulations 2004 (as amended) and all other applicable regulatory requirements.

Investigator Agreement and Notification of Conflict of Interest

Details can be found in Appendix 1

Table of Contents

1 SYNOPSIS 13
2 ABBREVIATIONS 25
3 BACKGROUND AND RATIONALE 27
3.1 Background 27
3.2 Preclinical studies 28
3.2.1 Immunogenicity (Jenner Institute, unpublished) 28
3.2.2 Non-human primate efficacy and immunogenicity – NIH (pre-print) 32
3.2.3 Antibody Dependant Enhancement and Immunopathology 37
3.3 Previous clinical experience 38
3.4 Rationale 44
3.4.1 Rationale for including older age groups 45
3.4.2 Phase I/II study - COV001 46
3.4.3 Rationale for including HIV Infected persons 46
4 OBJECTIVES AND ENDPOINTS 47
5 TRIAL DESIGN 55
5.1 Study groups 56
5.2 Trial volunteers 61
5.3 Definition of End of Trial 61
5.4 Potential Risks for volunteers 61
5.5 Known Potential Benefits 64
6 RECRUITMENT AND WITHDRAWAL OF TRIAL VOLUNTEERS 64
6.1 Identification of Trial Volunteers 64
6.2 Informed consent 65
6.3 Inclusion and exclusion criteria 67
6.3.1 Inclusion Criteria 67

6.3.2 Exclusion Criteria....68

6.3.3 Re-vaccination exclusion criteria (two-dose groups only)....70

6.3.4 Effective contraception for female volunteers....71

6.3.5 Withdrawal of Volunteers....71

6.3.5.1 Discontinuation from Study Procedures....72

6.4 Pregnancy....73

7 CLINICAL PROCEDURES....74

7.1 Schedule of Attendance....74

7.2 Observations, medical history and physical examination....74

7.3 Study visits....77

7.3.1 Screening visit....77

7.3.2 Day 0: Enrolment and vaccination visit....78

7.3.2.1 Vaccination....79

7.3.2.2 Sequence of Enrolment and Vaccination of Volunteers....80

7.3.3 Subsequent visits....80

7.3.4 Symptomatic volunteers....110

7.3.4.1 Diagnostic SARS-CoV-2 NAAT testing outside of the trial....110

7.3.4.2 COVID-19 testing visit....110

7.3.4.3 COVID-19 Testing plus 7 day phone call....111

7.3.4.4 Positive SARS-CoV-2 NAAT test and vaccination....111

7.3.5 Weekly PCR samples....111

7.3.6 Stool samples....112

7.4 Household Weekly Questionnaire (optional)....112

7.4.1 Medical notes review....112

7.4.2 Randomisation, blinding and code-breaking....113

7.4.3 Unblinded participants who are eligible to receive an approved or licensed SARS-CoV-2 vaccine....114

8 INVESTIGATIONAL PRODUCT....115

8.1 Description of ChAdOx1 nCoV-19 115
8.2 Supply 115
8.3 Storage 116
8.4 Administration 116
8.5 Rationale for selected dose 117
8.6 Minimising environmental contamination with genetically modified organisms (GMO) 121
8.7 Control vaccine 121
8.8 Compliance with Trial Treatment 122
8.9 Accountability of the Trial Treatment 122
8.10 Concomitant Medication 122
8.11 Provision of Treatment for Controls 123
9 ASSESSMENT OF SAFETY 123
9.1 Definitions 123
9.1.1 Adverse Event (AE) 123
9.1.2 Adverse Reaction (AR) 124
9.1.3 Serious Adverse Event (SAE) 124
9.1.4 Serious Adverse Reaction (SAR) 125
9.1.5 Suspected Unexpected Serious Adverse Reaction (SUSAR) 125
9.2 Expectedness 125
9.3 Foreseeable Adverse Reactions: 125
9.4 Adverse Events of Special Interest 125
9.5 Causality 126
9.6 Reporting Procedures for All Adverse Events 127
9.7 Assessment of severity 129

9.8 Reporting Procedures for Serious AEs 131

9.9 Reporting Procedures for SUSARS 132

9.10 Development Safety Update Report 132

9.11 Procedures to be followed in the event of abnormal findings 132

9.12 Interim Safety Reviews 133

9.13 Data Safety Monitoring Board 133

9.14 Safety Group Holding Rules 134

9.14.1 Individual stopping rules (will apply to prime-boost groups only) 136

10 STATISTICS 138

9.1 Description of Statistical Methods 138

9.2 Primary efficacy 138

9.3 Safety & Reactogenicity 140

9.4 Immunogenicity 141

9.5 Subgroup analyses 141

9.6 Interim and primary analyses of the primary outcome 142

9.7 Final Analysis 143

9.8 Procedure for Accounting for Missing, Unused, and Spurious Data. 143

9.9 Inclusion in Analysis 143

9.10 Interim analysis for the combined DSMB 143

11 DATA MANAGEMENT 145

11.1 Data Handling 145

11.2 Record Keeping 146

11.3 Source Data and Case Report Forms (CRFs) 146

11.4 Data Protection 147

11.5 Data Quality 147

11.6 Archiving 147

12 QUALITY CONTROL AND QUALITY ASSURANCE PROCEDURES 148

12.1 Investigator procedures 148

12.2 Trial Steering Committee 148

The trial steering committee will meet regularly and as required. 148

12.3 Monitoring 148

12.4 Protocol deviation 149

12.5 Audit & inspection 149

13 SERIOUS BREACHES 149

14 ETHICS AND REGULATORY CONSIDERATIONS 150

14.1 Declaration of Helsinki 150

14.2 Guidelines for Good Clinical Practice 150

14.3 Ethical and Regulatory Approvals 150

14.4 Volunteer Confidentiality 150

15 FINANCING AND INSURANCE 151

15.1 Financing 151

15.2 Insurance 151

15.3 Compensation 152

15.4 Publication Policy 152

16 DEVELOPMENT OF A NEW PRODUCT/ PROCESS OR THE GENERATION OF INTELLECTUAL PROPERTY 152

17 Appendix 169

1 SYNOPSIS

**Title**	A phase 2/3 study to determine the efficacy, safety and immunogenicity of the candidate Coronavirus Disease (COVID-19) vaccine ChAdOx1 nCoV-19
**Trial Identifier**	COV002
**Trial Registration**	EudraCT number: 2020-001228-32<br><br>REC Reference: 20/SC/0179<br><br>IRAS: 281904
**Clinical Phase**	2/3
**Design**	A single-blind, randomised safety and efficacy study, with immunogenicity sub studies in older and younger age groups
**Population**	Main efficacy trial: Healthy adults aged ≥18 years.<br><br>Sequential age escalation/de-escalation immunogenicity sub studies:<br><br>1. Healthy adults aged between 56 – <70 years<br>2. Healthy adults aged 70 years or older<br>3. Healthy adults aged 18 – 55 years.<br>4. HIV positive adults aged 18 – 55 years.
**Planned Sample Size**	Total number to enrol: up to 12,390 participants.<br><br>**Sequential age escalation/de-escalation groups:**<br><br>**Group 1: Adults aged between 56 – 69 years**<br><br>a1) Single dose ChAdOx1 nCoV-19 $5x10^{10}$vp (Abs 260)*, N=30, OR<br><br>a2) Single dose MenACWY N=10, OR<br><br>a3) Two-dose ChAdOx1 nCoV-19 $5x10^{10}$vp (Abs 260) prime and 0.5mL (3.5 – 6.5 × 10^{10} vp, Abs 260, corrected for PS80) boost*, N=up to 30 participants

recruited from group 1a1 will be invited to receive a booster dose at the earliest available opportunity (minimum 4 weeks from prime), OR

a4) Two-dose MenACWY, N=up to 10 participants recruited from group 1a2 will be invited to receive a booster dose at the earliest available opportunity (minimum 4 weeks from prime).

b1) Two-dose ChAdOx1 nCoV-19 $5x10^{10}$vp (Abs 260) prime and 2.2 $x10^{10}$vp (qPCR) boost * (4-6 weeks apart), N=30, OR

b2) Two-dose MenACWY(4-6 weeks apart) , N=10

Group 2: Adults aged 70 years and above

a1) Single dose ChAdOx1-nCoV-19 $5x10^{10}$vp (Abs 260)*, N=50, OR

a2) Single dose MenACWY N=10, OR

a3) Two-dose ChAdOx1 nCoV-19 $5x10^{10}$vp (Abs 260) prime and 0.5mL (3.5 – 6.5 × 10^{10} vp, Abs 260, corrected for PS80) boost*, N=up to 50 participants recruited from group 2a1 will be invited to receive a booster dose at the earliest available opportunity (minimum 4 weeks from prime), OR

a4) Two-dose MenACWY, N=up to 10 participants recruited from group 2a2 will be invited to receive a booster dose at the earliest available opportunity (minimum 4 weeks from prime).

b1) Two-dose ChAdOx1 nCoV-19 $5x10^{10}$vp (Abs 260) prime and 2.2 $x10^{10}$vp (qPCR) boost * (4-6 weeks apart), N=50, OR

b2) Two-dose MenACWY (4-6 weeks apart), N=10

Group 4: Adults aged 18-55 (n=up to 3550)

a1) Single dose ChAdOx1 nCoV-19 $5x10^{10}$vp (Abs 260)*, N= up to 1775, OR

a2) MenACWY, N= up to 1775 OR

b1) Two-dose ChAdOx1 nCoV-19 $5x10^{10}$vp (Abs 260) prime and $2.2x10^{10}$vp (qPCR) boost*, (4-6 weeks apart), N= up to 50, OR

b2) Two-dose MenACWY, (4-6 weeks apart), N=up to 50

NB: A subset of up to 100 participants in group 4a will be invited to receive a booster dose in 4b, keeping the overall sample size in group 4 the same.

c1) Two-dose ChAdOx1 nCoV-19 $5x10^{10}$vp (Abs 260) prime and 0.5mL (3.5 – 6.5 × 10^{10} vp, Abs 260, corrected for PS80) boost* OR ChAdOx1 nCoV-19 $5x10^{10}$vp (qPCR) boost, (at least 4 weeks apart), N= up to 1725, OR

c2) Two-dose MenACWY, (at least 4 weeks apart), N=up to 1725

NB: Participants in group 4a, excluding those already in 4b, will be invited to receive a booster dose in 4c, keeping the overall sample size in group 4 the same.

Group 5: Adults aged 18-55 years

a1) ChAdOx1 nCoV-19 $5x10^{10}$vp (Abs 260)*, N= 50 OR

a2) MenACWY, N= 50, OR

a3) Two-dose ChAdOx1 nCoV-19 $5x10^{10}$vp (Abs 260) prime and 0.5mL (3.5 – 6.5 × 10^{10} vp, Abs 260, corrected for PS80) boost*, N=up to 50 participants recruited from group 5a1 will be invited to receive a booster dose at the earliest available opportunity (minimum 4 weeks from prime),

a4) Two-dose MenACWY, N=up to 50 participants recruited from group 5a2 will be invited to receive a booster dose at the earliest available opportunity (minimum 4 weeks from prime).

b1) ChAdOx1 nCoV-19 $5x10^{10}$vp (qPCR)*, N= 25 OR

b2) MenACWY, N= 25

(B cell immunology only)

c1) ChAdOx1 nCoV-19 $5x10^{10}$vp (qPCR)*, N= 25 OR

c2) MenACWY, N= 25

(B and T-cell immunology)

d1) Two dose ChAdOx1 nCoV-19 0.5mL (3.5 – 6.5 × 10^{10} vp Abs 260, corrected for PS80)*, (4-6 weeks apart) N=50, OR

d2) Two dose MenACWY, N= 10

(B cell immunology and T-cell in a subset)

e) Two dose ChAdOx1 nCoV-19 0.5mL (Covishield 0.9 x 10^11 vp/mL), 4-6 weeks apart N=15

f) Two dose ChAdOx1 nCoV-19 (Covishield 0.9 x 10^11 vp/mL), 0.25mL prime and 0.5mL boost 4-6 weeks apart N=15

Group 6: Adults aged 18-55 years (n= up to 6000)

a1) ChAdOx1 nCoV-19 $5x10^{10}$vp (qPCR), N= up to 3000

a2) MenACWY, N= up to 3000

b1) Two-dose ChAdOx1 nCoV-19 $5x10^{10}$vp (qPCR) prime and 0.5mL (3.5 – 6.5 × 10^{10} vp Abs 260, corrected for PS80) boost* OR ChAdOx1 nCoV-19 $5x10^{10}$vp (qPCR) boost, (at least 4 weeks apart), N= up to 3000, OR

b2) Two-dose MenACWY, (at least 4 apart), N=up to 3000

NB: Participants in group 6a, will be invited to receive a booster dose in 6b, keeping the overall sample size in group 6 the same.

Group 7: Adults aged between 56 – 69 years (n=80):

a1) Single dose ChAdOx1-nCoV-19 $5x10^{10}$vp (qPCR)*, N=30, OR

a2) Single dose MenACWY N=10, OR

b1) Two-dose ChAdOx1 nCoV-19 $5x10^{10}$vp (qPCR)* (4-6 weeks apart), N=30, OR

b2) Two-dose MenACWY(4-6 weeks apart) , N=10

Group 8: Adults aged 70 years and above (n=120):

a1) Single dose ChAdOx1-nCoV-19 $5x10^{10}$vp (qPCR)*, N=50, OR

a2) Single dose MenACWY N=10, OR

b1) Two-dose ChAdOx1 nCoV-19 $5x10^{10}$vp (qPCR) prime and 0.5mL (3.5 – 6.5 × 10^{10} vp, Abs 260, corrected for PS80) boost* OR ChAdOx1 nCoV-19 $5x10^{10}$vp (qPCR) boost (4-6 weeks apart), N=50, OR

b2) Two-dose MenACWY (4-6 weeks apart), N=10

Group 9: Adults aged 56-69 (n=1000, +/- 10%)

a1) Two dose ChAdOx1 nCoV-19 0.5mL (3.5 – 6.5 × 10^{10} vp, Abs 260, corrected for PS80)*, (4-6 weeks apart) N=500, OR

a2) Two dose MenACWY (4-6 weeks apart), N= 500

Group 10: Adults aged 70 years and above (n=1000, +/- 10%)

a1) Two dose ChAdOx1 nCoV-19 0.5mL (3.5 – 6.5 × 10^{10} vp, Abs 260, corrected for PS80)*, (4-6 weeks apart) N=500, OR

a2) Two dose MenACWY (4-6 weeks apart), N= 500

Group 11: Adults aged 18-55 who previously received a ChAdOx1 vectored vaccine (n=up to 60)

a1) Two dose ChAdOx1 nCoV-19 0.5mL (3.5 – 6.5 × 10^{10} vp, Abs 260, corrected for PS80)*, (4-6 weeks apart) N=up to 60

Group 12: HIV positive adults aged 18-55 (n=up to 60)

a1) Two dose ChAdOx1 nCoV-19 0.5mL (3.5 – 6.5 × 10^{10} vp, Abs 260, corrected for PS80)*, (4-6 weeks apart) N= up to 60

* See section 8.5 for further information on dosing

Visit Schedule : See schedule of attendances tables in section 7.3.3

Planned Trial Duration 12 months post last vaccination per participant

	Objective	Outcome Measure

**Primary**	To assess efficacy of the candidate ChAdOx1 nCoV-19 against COVID-19 in adults aged 18 years and older.	Virologically confirmed (PCR* positive) symptomatic cases of COVID-19
**Co-Primary**	To assess the safety of the candidate vaccine ChAdOx1 nCoV-19 in adults .	Occurrence of serious adverse events (SAEs) throughout the study duration.
**Secondary**	To assess the safety, tolerability and reactogenicity profile of the candidate vaccine ChAdOx1 nCoV-19	a) occurrence of solicited local reactogenicity signs and symptoms for 7 days following vaccination;<br>b) occurrence of solicited systemic reactogenicity signs and symptoms for 7 days following vaccination;<br>c) occurrence of unsolicited adverse events (AEs) for 28 days following vaccination ;<br>d) change from baseline for safety laboratory measures (except groups 4, 6, 9 and 10);<br>e) Occurrence of disease enhancement episodes
	To assess efficacy of the candidate ChAdOx1 nCoV-19 against severe and non-severe COVID-19	a) Hospital admissions associated with COVID-19

		b) Intensive care unit (ICU) admissions associated with COVID-19<br>c) Deaths associated with COVID-19<br>d) Seroconversion against non-Spike SARS-CoV-2 antigens<br>e) Severe COVID-19 disease (defined according to clinical severity scales)
	To assess humoral immunogenicity of ChAdOx1 nCoV-19	a) Antibodies against SARS-CoV-2 spike protein (seroconversion rates) at Day 28 post-vaccination.<br>b) Proportion of seroconversion to antibodies against SARS-CoV-2 spike protein at Day 28 post-vaccination.
	To assess cellular immunity of ChAdOx1 nCoV-19 in older adults (groups 1, 2, 7 and 8 only)	a) Interferon-gamma (IFN-γ) enzyme-linked immunospot (ELISpot) responses to SARS-CoV-2 spike protein;
	To assess the safety and immunogenicity of a booster dose of ChAdOx1 nCoV-19 in older adults aged 56 years or older (two-dose schedules for groups 1, 2, 7 and 8 only)	a) occurrence of solicited local reactogenicity signs and symptoms for 7 days following booster vaccination;<br>b) occurrence of solicited systemic reactogenicity signs and symptoms for 7 days following booster vaccination;

		c) occurrence of unsolicited adverse events (AEs) for 28 days following booster vaccination;<br>d) change from baseline and change from pre-booster for safety laboratory measures and;<br>e) Occurrence of disease enhancement episodes<br>f) Antibodies against SARS-CoV-2 spike protein at Day 56 post-vaccination.<br>g) Proportion of seroconversion to antibodies against SARS-CoV-2 spike protein at Day 56 post-vaccination
Tertiary	Exploratory Immunology	a) virus neutralising antibody (NAb) assays against live and/or pseudotype SARS-CoV-2 virus<br>b) Cell analysis by flow cytometry assays<br>c) Functional antibody assays<br>d) Anti-vector immunity induced by 1 or 2 doses of ChAdOx1 nCoV-19
	Measure exposure to COVID-19	Reported by weekly survey to collect information about cases amongst household contacts and friends, contact with the general public, infection control procedures

	Exploratory efficacy against infection<br>• To assess efficacy of the candidate ChAdOx1 nCoV-19 against SARS-CoV-2 infection	a) PCR* positive SARS-CoV-2 infection<br>b) Differences in viral loads between those with severe, mild, and asymptomatic PCR+* SARS-CoV-2 infections
	Compare safety, reactogenicity and immunogenicity between different manufacturing batches of ChAdOx1 nCoV-19 used in COV001 and COV002	a) Differences in safety, reactogenicity and immunogenicity profiles between Group 1 in COV001 and Group 5 in COV002 (proportion of Grade 3 solicited AEs, occurrence of fevers, seroconversion rates at D28, neutralising antibody titres and differences in T-cell responses at D14).
	Compare safety, reactogenicity and immunogenicity between different methods for measuring doses (Abs260, Abs 260 corrected for PS80 and qPCR) of ChAdOx1 nCoV-19	a) Differences in safety, reactogenicity and immunogenicity profiles between Groups 1, 2, and 5A compared with Groups, 7, 8, and 5B, C and D respectively (proportion of Grade 3 solicited AEs, occurrence of fevers, seroconversion rates at D28, neutralising antibody titres and differences in T-cell responses at D14).
	To assess vaccine induced mucosal immunity	Nasal mucosa IgA levels at D0 and D28 in a subset of individuals

	To compare viral shedding on stool samples of SARS-CoV-2 PCR* positive individuals	Differences in viral shedding on stool at 7 days and beyond post SARS-CoV-2 positivity.
	To compare immunogenicity of ChAdOx1 nCoV-19 in participants receiving 1 or 2 doses in groups 1, 2, 7 and 8	a) Differences in antibody titres (ELISA and Neutralising antibodies) in participants who received 1 or 2 doses of ChAdOx1 nCoV-19 (groups 1, 2, 7 and 8)<br>b) Longevity of immune responses in participants who received 1 or 2 doses of ChAdOx1 nCoV-19
	To describe the impact of previous vaccination with other ChAdOx1 vectored vaccines on safety and immune responses to ChAdOx1 nCoV-19	Differences reactogenicity profile, antibody titres and T-cell responses between groups 5d and 11 and their relationship with anti-vector neutralising antibody titres.
	To assess the cell-mediated and humoral immunogenicity profile of ChAdOx1 nCoV-19 vaccine in HIV infected adults	Cell-mediated and humoral responses against SARS-Cov-2 These will be measured by the following:<br>a) Proportion of seroconversion to antibodies (Ab) against SARS-CoV-2 spike protein measured by ELISA.<br>b) Interferon-gamma enzyme linked immunospot (ELISpot) responses to SARS-CoV-2 spike protein

		c) Intracellular Cytokine analyses of CD4 and CD8-specific SARS-CoV-2 spike protein responses<br>d) Further exploratory immunology
	To assess whether increasing age and or CD4 nadir are associated with a lack of immune response in HIV infected adults	a) relationship between nadir CD4 count and vaccine immune responses<br>b) relationship between age at enrolment and vaccine immune response<br>c) Immune responses to ChAdOx1 nCoV-19 (assessed as described above)
	To assess the safety of the candidate vaccine ChAdOx1 nCoV-19 in HIV infected adults	a) Occurrence of serious adverse events (SAEs) throughout the study duration<br>b) occurrence of solicited local reactogenicity signs and symptoms for 7 days following vaccination<br>c) occurrence of solicited systemic signs and symptoms for 7 days following each vaccination<br>d) occurrence of unsolicited AEs for 28 days following each vaccination
	To assess Impact of vaccination on HIV reservoirs	Change in Total HIV DNA copies per million CD4 T cells

	To assess immunological correlates of protection in relation to occurrence of COVID-19 disease in ChAdOx1 nCoV-19 recipients	Immunological endpoints and COVID-19 disease endpoints in ChAdOx1 nCoV-19 recipients
**Investigational products**	a) ChAdOx1 nCoV-19, a replication-deficient simian adenoviral vector expressing the spike (S) protein of SARS-CoV-2<br>b) MenACWY, Meningococcal Group A, C, W-135 and Y conjugate vaccine	

*or other nucleic acid amplification test (NAAT)

**Formulation**	ChAdOx1 nCoV-19: Aqueous solution for injection<br><br>MenACWY: powder and solvent for solution for injection
**Route of Administration**	Intramuscular (IM)
**Dose per Administration**	ChAdOx1 nCoV-19*:<br>- 2.2×10^{10} vp (qPCR)<br>- 2.5×10^{10} vp (qPCR)<br>- 5×10^{10} VP (Abs 260)<br>- 5×10^{10} VP (qPCR)<br>- 0.5mL ($3.5 – 6.5 \times 10^{10}$ vp, Abs 260, corrected for PS80)*<br>- 0.5mL (Covishield 0.9×10^{11} vp/mL)<br>- 0.25mL (Covishield 0.9×10^{11} vp/mL)<br><br>Men ACWY: 0.5 mL

* See section 8.5 for further information on dosing

2 ABBREVIATIONS

**Abs 260**	Absorbance 260 nm
**AdHu**	Human adenovirus
**AdHu5**	Human adenovirus serotype 5
**AE**	Adverse event
**AID**	Autoimmune Disease
**CCVTM**	Centre for Clinical Vaccinology and Tropical Medicine, Oxford
**CBF**	Clinical BioManufacturing Facility
**CEF**	Chick embryo fibroblast
**ChAdOx**	Chimpanzee adenovirus 1
**CI**	Confidence interval
**COP**	Code of Practice
**CRF**	Case Report Form or Clinical Research Facility
**CTRG**	Clinical Trials & Research Governance Office, Oxford University
**CTL**	Cytotoxic T Lymphocyte
**DSUR**	Development Safety Update Report
**ELISPOT**	Enzyme-linked immunospot
**GCP**	Good Clinical Practice
**GMO**	Genetically modified organism
**GMT**	Geometric Mean Titre
**GP**	General Practitioner
**HCG**	Human Chorionic Gonadotrophin
**HEK**	Human embryonic kidney
**HIV**	Human Immunodeficiency virus
**HLA**	Human leukocyte antigen
**HRA**	Health Research Authority
**IB**	Investigator Brochure
**ICH**	*International Council for Harmonisation*
ICMJE	*International Committee of Medical Journal Editors*
ICS	*Intracellular Cytokine Staining*
**ID**	Intradermal
**IFNγ**	Interferon gamma
**IM**	Intramuscular
**IMP**	Investigational Medicinal Product
**IMP-D**	Investigational Medicinal Product Dossier
**IV**	Intravenous
**NAAT**	Nucleic acid amplification assay
**MenACWY**	Quadrivalent capsular group A, C, W and Y meningococcal protein-polysaccharide conjugate vaccine
**MHRA**	Medicines and Healthcare Products Regulatory Agency
**MVA**	Modified vaccinia virus Ankara
**NHS**	National Health Service
**NIH**	National Institutes of Health
**NIHR**	National Institute for Health Research
**PBMC**	*Peripheral blood mononuclear cell*

**PCR**	Polymerase chain reaction
**PI**	Principal Investigator
**PS80**	Polysorbate 80
**QP**	Qualified Person
**qPCR**	Quantitative polymerase chain reaction
**REC**	Research Ethics Committee
**SAE**	Serious adverse event
**SC**	Subcutaneous
**SmPc**	Summary of Product characteristics
**SOP**	Standard Operating Procedure
**SUSAR**	Suspected unexpected serious adverse reaction
**µg**	microgram
**Vp**	viral particle
**VV**	viral vector
**WHO**	World Health Organisation

3 BACKGROUND AND RATIONALE

3.1 Background

In December 2019, a cluster of patients with pneumonia of unknown cause was linked to a seafood wholesale market in Wuhan, China and were later confirmed to be infected with a novel coronavirus, known as 2019-nCoV [1]. The virus was subsequently renamed to SARS-CoV-2 because it is similar to the coronavirus responsible for severe acute respiratory syndrome (SARS-CoV), a lineage B betacoronavirus. SARS-CoV-2 shares more than 79% of its sequence with SARS-CoV, and 50% with the coronavirus responsible for Middle East respiratory syndrome (MERS-CoV), a member of the lineage C betacoronavirus[2]. COVID-19 is the infectious disease caused by SARS-CoV-2. By January 2020 there was increasing evidence of human to human transmission as the number of cases rapidly began to increase in China. Despite unprecedented containment measures adopted by the Chinese government, SARS-CoV-2 rapidly spread across the world. The WHO declared the COVID-19 outbreak a public health emergency of international concern on 30th January 2020. As of 26th May 2020, over 5, 584,091 cases have been reported with more than 349,894 deaths and 188 countries affected.

Coronaviruses (CoVs) are spherical, enveloped, large positive-sense single-stranded RNA genomes. One-fourth of their genome is responsible for coding structural proteins, such as the spike (S) glycoprotein, envelope (E), membrane (M) and nucleocapsid (N) proteins. E, M, and N are mainly responsible for virion assembly whilst the S protein is involved in receptor binding, mediating virus entry into host cells during CoVs infection via different receptors.[3] SARS-CoV-2 belongs to the phylogenetic lineage B of the genus *Betacoronavirus* and it recognises the angiotensin-converting enzyme 2 (ACE2) as the entry receptor [4]. It is the seventh CoV known to cause human infections and the third known to cause severe disease after SARS-CoV and MERS-CoV.

The spike protein is a type I, trimeric, transmembrane glycoprotein located at the surface of the viral envelope of CoVs, which can be divided into two functional subunits: the N-terminal S1 and the C-terminal S2. S1 and S2 are responsible for cellular receptor binding via the receptor binding domain (RBD) and fusion of virus and cell membranes respectively, thereby mediating the entry of SARS-CoV-2 into target cells.[3] The roles of S in receptor binding and

membrane fusion make it an ideal target for vaccine and antiviral development, as it is the main target for neutralising antibodies.

ChAdOx1 nCoV-19 vaccine consists of the replication-deficient simian adenovirus vector ChAdOx1, containing the structural surface glycoprotein (Spike protein) antigen of the SARS CoV-2 (nCoV-19), with a leading tissue plasminogen activator (tPA) signal sequence. ChAdOx1 nCoV-19 expresses a codon-optimised coding sequence for the Spike protein from genome sequence accession GenBank: MN908947. The tPA leader sequence has been shown to be beneficial in enhancing immunogenicity of another ChAdOx1 vectored CoV vaccine (ChAdOx1 MERS) [5].

3.2 Preclinical studies

Refer to the Investigator Brochure for most recent pre-clinical data update

3.2.1 Immunogenicity (Jenner Institute, unpublished)

Mice (balb/c and CD-1) were immunised with ChAdOx1 expressing SARS-CoV-2 Spike protein or green fluorescent protein (GFP). Spleens were harvested for assessment of IFY ELISpot responses and serum samples were taken for assessments of S1 and S2 antibody responses on ELISA at 9 or 10 days post vaccination. The results of this study show that a single dose of ChAdOx1 nCoV was immunogenic in mice.

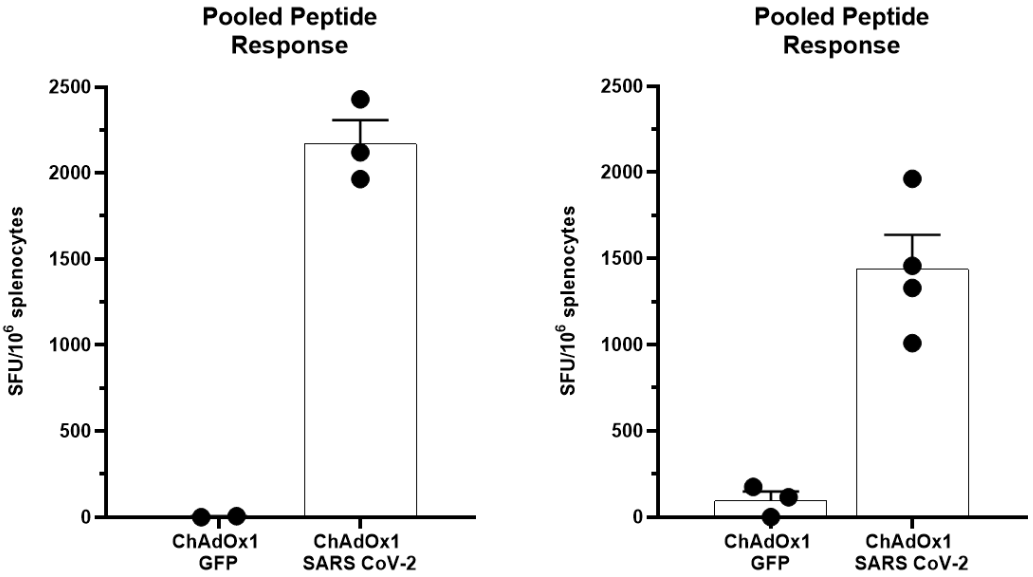

Figure 1. Summed splenic IFN-γ ELISpot responses of BALB/c (left panel) and CD-1 (right panel) mice, in response to peptides spanning the spike protein from SARS-CoV-2, nine or ten days post vaccination, with 1.7×10^{10} vp ChAdOx1 nCoV-19 or 8×10^{9} vp ChAdOx1 GFP. Mean with SEM are depicted

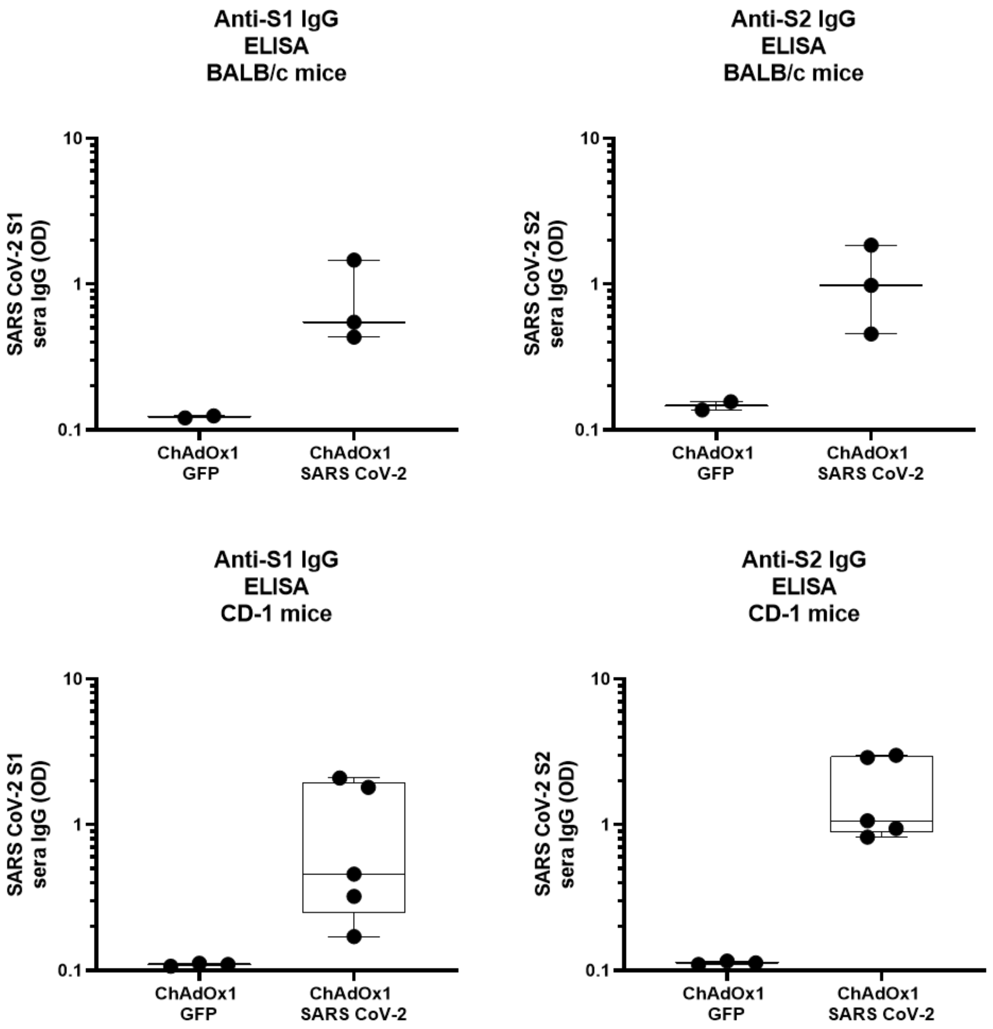

Figure 2. Box and whisker plot of the optical densities following ELISA analysis of BALB/C mouse sera (Top panel) incubated with purified protein spanning the S1 domain (left) or purified protein spanning the S2 domain (right) of the SARS-CoV-2 spike nine or ten days post vaccination, with 1.7×10^{10} vp ChAdOx1 nCoV-19 or 8×10^{9} vp ChAdOx1 GFP. Box and whisker plots of the optical densities following ELISA analysis of CD-1 mouse sera (Bottom panel) incubated with purified protein spanning the S1 domain (left) or purified protein spanning the S2 domain (right) of the SARS-CoV-2 spike.

Two mouse strains (BALB/c, N=5 and outbred CD1, N=8) were vaccinated intramuscularly (IM) with ChAdOx1 nCoV-19 or ChAdOx1 GFP, a control vaccine expressing green fluorescent protein. Humoral and cellular immunity were studied 9-14 days later. Total IgG titers were detected against spike protein subunits S1 and S2 in all vaccinated mice (Figure 3a). Profiling of the IgG subclasses showed a predominantly Th1 response post vaccination (Figure 4a). Virus-specific neutralising antibodies were detected in all mice vaccinated with ChAdOx1 nCoV-19, whereas no neutralisation was detected in serum from mice vaccinated with ChAdOx1 GFP (Figure 5b). Splenic T-cell responses measured by IFN-γ ELISpot and intracellular cytokine staining (ICS) were detected against peptides spanning the full length of the spike construct (Figure 3c). Again, a strong Th1-type response was detected post vaccination as supported by high levels of IFN-γ and TNF-α, and low levels of IL-4 and IL-10 (Figure 3d & Figure 4b-c).

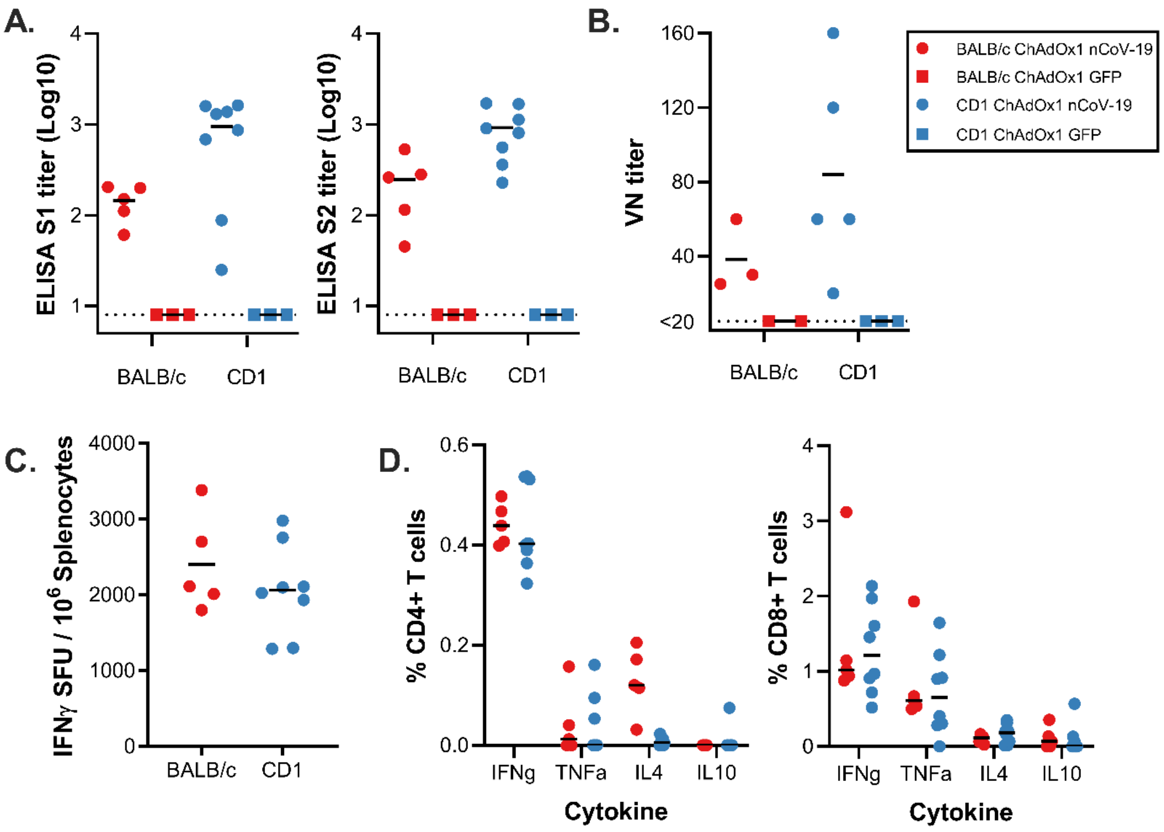

Figure 3: Humoral and cellular immune responses to ChAdOx1 58 nCoV-19 vaccination in mice. A). End point titer of serum IgG detected against S1 or S2 protein. Control mice were below the limit of detection. B). Virus neutralizing titer in serum. C). Summed IFN-γ ELISpot responses in splenocytes toward peptides spanning the spike protein. Control mice had low

(<100 SFU) or no detectable response. D). Summed frequency of spike-specific cytokine positive CD4+ or CD8+ T cells. BALB/c = red; CD1 = blue; vaccinated = circle; control = square; dotted line = limit of detection; line = mean; SFU = spot-forming units.

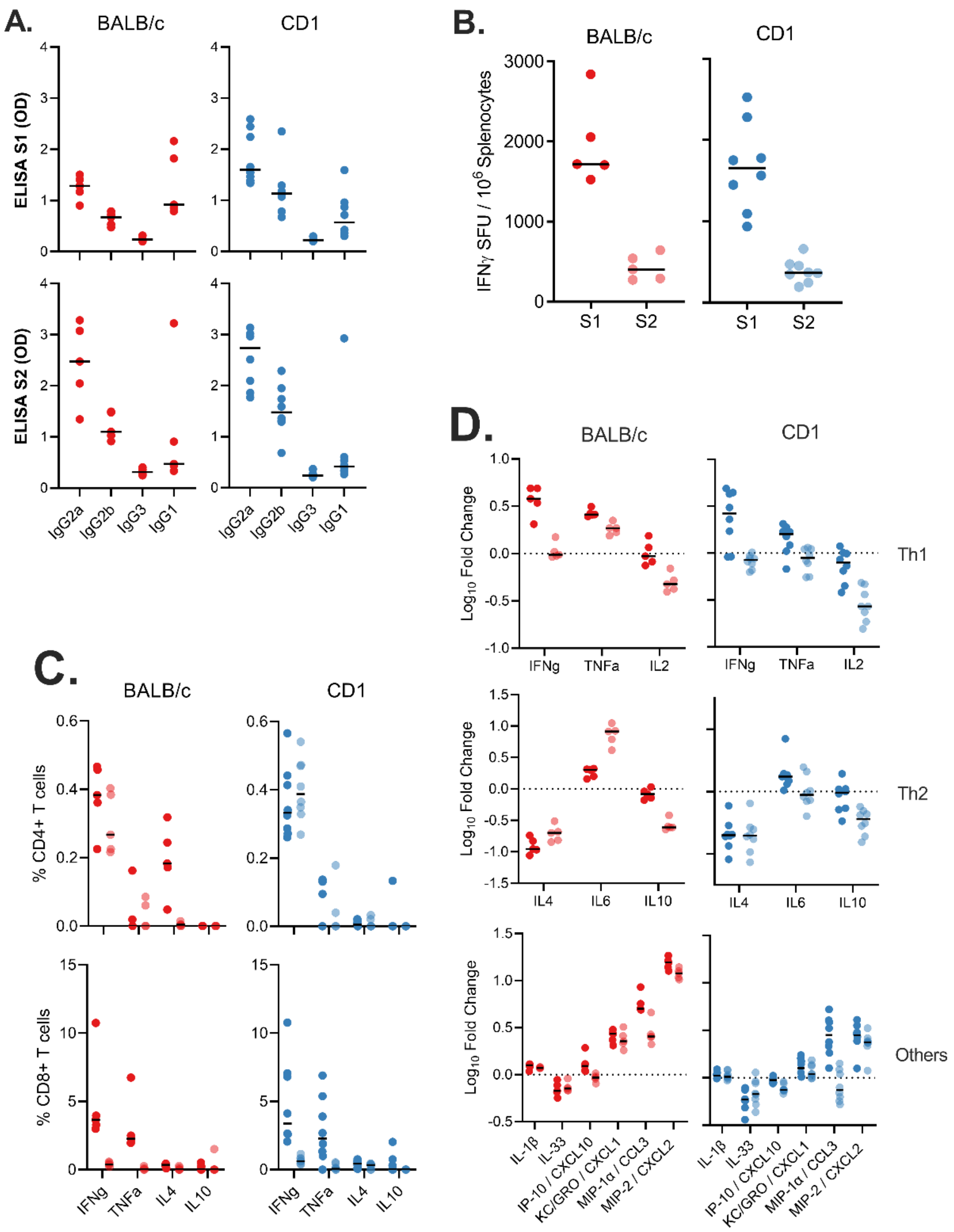

Figure 4. Antigen specific responses following ChAdOx1 nCov19 vaccination. A). IgG subclass antibodies detected against S1 or S2 protein in sera of BALB/c or CD1 mice. B). Frequency of cytokine positive CD4+ or CD8+ T cells following stimulation of splenocytes with S1 pool (dark) or S2 pool (transparent) peptides in BALB/c (red) and CD1 (blue) mice. C) Percentage of CD4+

or CD8+ T cells in BALB/c or CD1. D) Log10 fold change in cytokine levels in supernatant from S1 (dark) and S2 (transparent) stimulated splenocytes when compared to corresponding unstimulated 407 splenocyte sample for BALB/c and CD1 mice.

3.2.2 Non-human primate efficacy and immunogenicity – NIH (pre-print)

Details of this experiment are available at: https://www.biorxiv.org/content/10.1101/2020.05.13.093195v1

doi: https://doi.org/10.1101/2020.05.13.093195. In this study, two groups of rhesus macaques were utilized. Animals were adults, vaccinated group contained six animals, control group contained three animals. Group 1 was vaccinated with ChAdOx1 nCoV-19 at a dose of 2.5×10^{10} vp/animal at 28 days before challenge. Group 2 (control) was vaccinated with ChAdOx1 GFP at a dose of 2.5×10^{10} vp/animal at 28 days before challenge. The dose is half that which is planned for humans.

Animals were challenged with 2.6×10^{6} TCID¬50/animal of SARS-CoV-2 using 4 routes: intranasal (0.5ml per nostril), intratracheal (4ml), oral (1ml), and ocular (0.25ml per eye) of a 4×10^{5} TCID50/ml virus dilution in sterile DMEM.

Animals were examined on 1, 3, 5, and 7 days post challenge and will be euthanized at 7 days post challenge.

Humoral response

Antibodies in serum against SARS-CoV-2 spike protein were measured by ELISA. An increase in ELISA titer (Figure 5B) and neutralizing antibodies (Figure 5C) was found when comparing serum obtained before initial vaccination (-28), and at day of challenge (0).

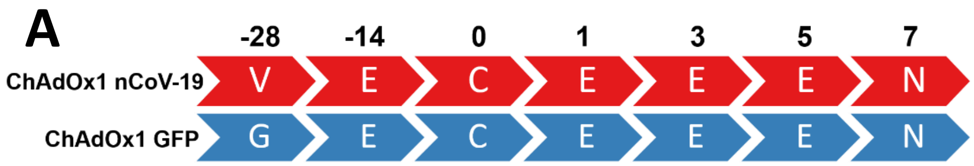

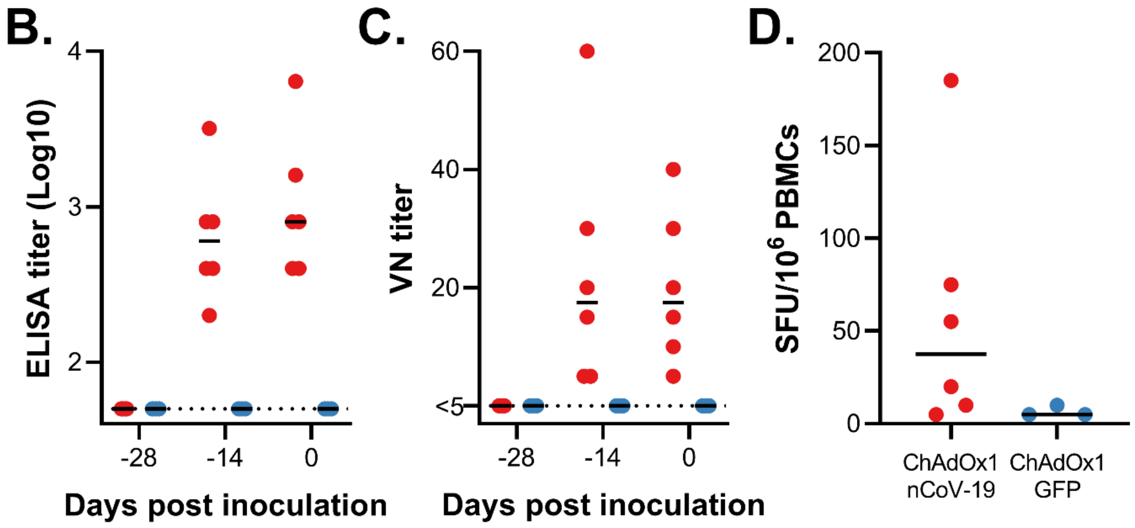

Figure 5. Humoral and cellular response to ChAdOx1 nCoV-19 vaccination in rhesus macaques. A. Study schedule for NHPs. V = vaccination with ChAdOx1 nCoV-19; G = vaccination with ChAdOx1 GFP; E = exam; N = necropsy. B. End point titre of serum IgG detected against S protein via ELISA at -28, -14 and 0 DPI. C. Two-fold serial-diluted serum samples were tested for neutralizing antibodies against SARS CoV-2 in VeroE6 cells at -28, -14 and 0 DPI. D. Summed S protein specific IFN-γ ELISpot responses. Vaccinated animals = red; control animals = blue; dotted line = limit of detection.

Cytokine response

Cytokines in serum were analysed after challenge to monitor immune responses. We observed an upregulation in IFN-γ at 1 DPI in ChAdOx1 nCoV-19 vaccinated animals, but not in control animals. No significant differences were observed between ChAdOx1 nCoV-19 and control animals for TNF-α, IL-2, IL-4, IL-6, and IL-10 (Figure 6).

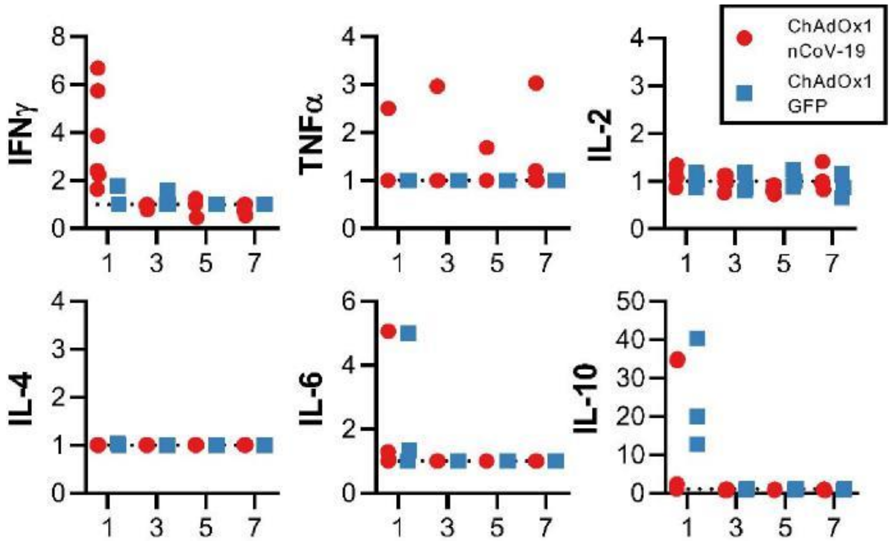

Figure 6. Serum cytokines in rhesus macaques challenged with SARS-CoV-2. Fold increase in cytokines in serum compared to 0 DPI values.

<u>Shedding of virus</u>

Viral gRNA load was high in lung tissue of control animals and viral sgRNA was detected in 2 out of 3 control animals (Figure 7d). In contrast, the viral gRNA load was significantly lower in lung tissue obtained from vaccinated animals as determined via Mann-Whitney's rank test and below limits of detection in two vaccinated animals. Viral sgRNA was detected in lung tissue obtained from 1 out of 6 vaccinated animals ($p<0.0001$, Figure 11d). Viral gRNA could be detected in other tissues but was low in both groups (Figure 8).

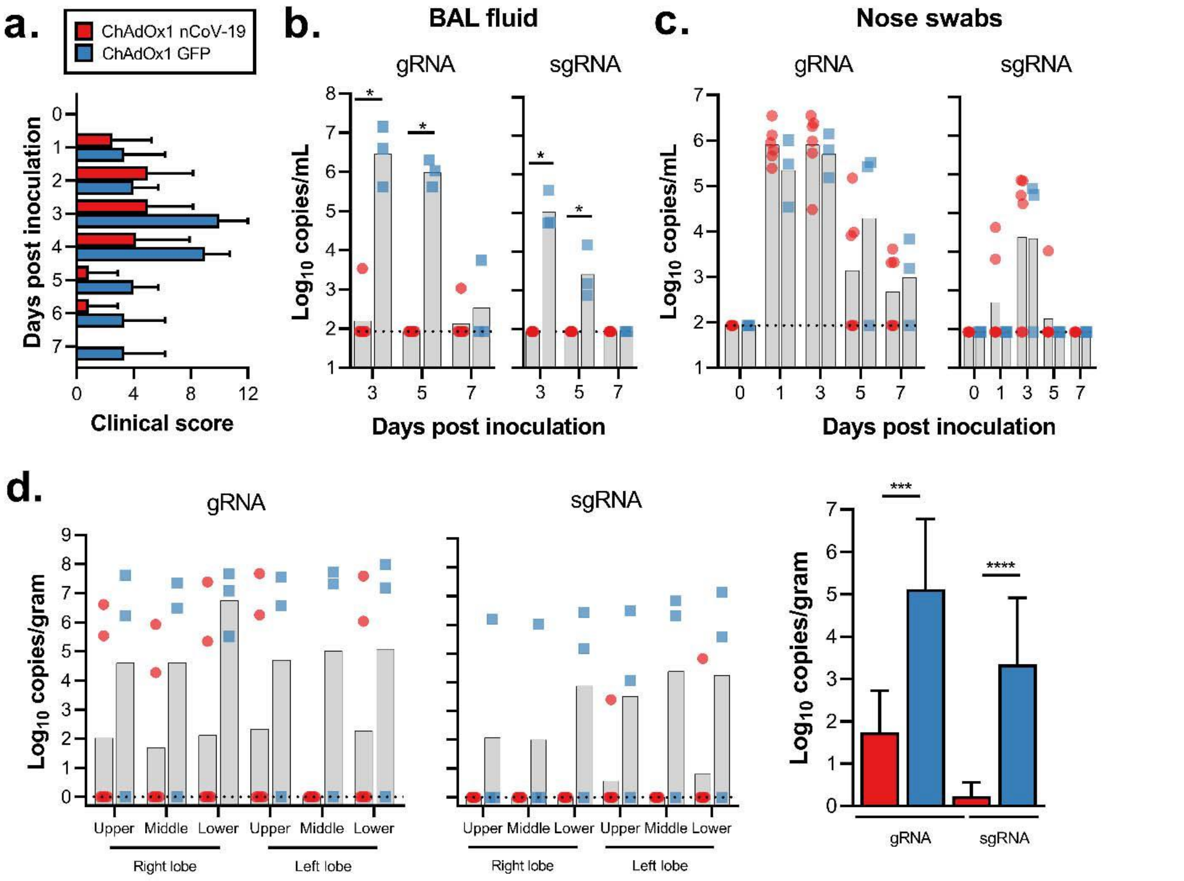

Figure 7. Clinical signs and viral load in rhesus macaques inoculated with SARS-CoV-2 after vaccination with ChAdOx1 nCoV-19. a. Mean clinical score with standard deviation in NHPs. Any scoring associated with food was removed from final score. b. Viral load in BAL fluid obtained from rhesus macaques, bar at geometric mean. *=p-value<0.0166. c. Viral load in nose swabs obtained from rhesus macaques, bar at geometric mean. d. Viral load in tissues at 7 DPI. Pictured are individual values with geometric mean bars (left panels) and geometric mean of all lung lobes per group (right panel). ***=p-value<0.001; ****=p-value<0.0001. Vaccinated animals = red circles; control animals = blue squares; dotted line = limit of detection.

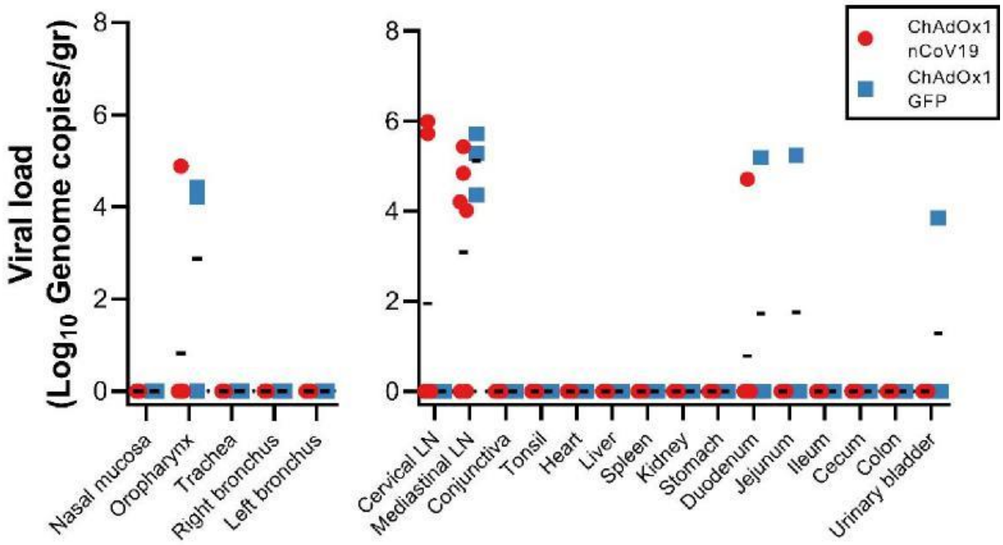

Figure 8. Viral load in rhesus macaques challenged with SARS-CoV-2. Viral genomic RNA in respiratory tissues excluding lung tissue (left panel) and other tissues (right panel). A two-tailed Mann-Whitney's rank test was performed to investigate statistical significance. Bonferroni correction was applied, and thus statistical significance was reached at p>0.0125.

Pulmonary pathology

At 7 days post inoculation, all animals were euthanized, and tissues were collected. None of the vaccinated monkeys developed pulmonary pathology after inoculation with SARS-CoV-2. All lungs were histologically normal and no evidence of viral pneumonia nor immune-enhanced inflammatory disease was observed. In addition, no SARS-CoV-2 antigen was detected by immunohistochemistry in the lungs of any of the vaccinated animals. Two out of 3 control animals developed some degree of viral interstitial pneumonia. Lesions were widely separated and characterized by thickening of alveolar septae by small amounts of edema fluid and few macrophages and lymphocytes. Alveoli contained small numbers of pulmonary macrophages and, rarely, edema. Type II pneumocyte hyperplasia was observed. Multifocally, perivascular infiltrates of small numbers of lymphocytes forming perivascular cuffs were observed. Immunohistochemistry demonstrated viral antigen in type I and II pneumocytes, as well as in alveolar macrophages (Figure 9.

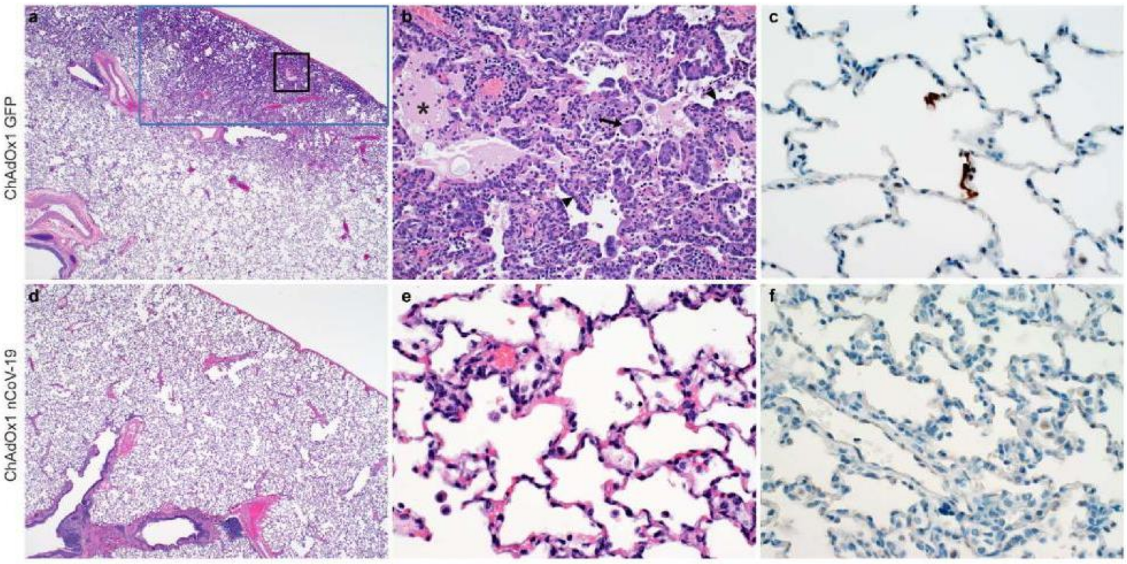

Figure 9 Histological changes in lungs of rhesus macaques on 7 dpi. a) Focal interstitial pneumonia in lungs of a control animal (blue box). The area in the black box is magnified in panel b. b) Interstitial pneumonia with edema (asterisk), type II pneumocyte hyperplasia (arrowhead) and syncytial cells (arrow) in control animals. c) SARS-CoV-2 antigen (visible as red-brown staining) was detected by immunohistochemistry in type I and type II pneumocytes in the lungs of control animals. d) No histological changes were observed in the lungs of ChadOx1 nCoV-19-vaccinated animals. e) Higher magnification of lung tissue in panel d. No evidence of pneumonia or immune-enhanced inflammation is observed. f) No SARS-CoV-2 antigen was detected by immunohistochemistry in the lungs of vaccinated animals. Magnification: panels a, d 40x; panels b, c, e, f 400x.

Further pre-clinical efficacy studies of ChAdOx1 nCoV-19 in ferrets and non-human primates are in progress. Results will be included in the Investigator's Brochure when available

3.2.3 Antibody Dependant Enhancement and Immunopathology

Safety concerns around the use of full length coronavirus Spike glycoproteins and other viral antigens (nucleoprotein) as a vaccine antigen have been raised following historical and limited reports of immunopathology and antibody dependant enhancement (ADE) reported *in vitro* and post SARS-CoV challenge in mice, ferrets and non-human primates immunised with whole SARS-CoV inactivated or full-length S protein based vaccines, including a study using Modified Vaccinia Ankara as a vector.[6-8] To date, there has been one report of lung immunopathology

following MERS-CoV challenge in mice immunised with an inactivated MERS-CoV candidate vaccine.[9] However, in preclinical studies of ChAdOx1 immunisation and MERS-CoV challenge, no ADE was observed in hDPP4 transgenic mice, dromedary camels or non-human primates (van Doremalen et al, manuscript submitted).[10,11]

The risks of inducing lung immunopathology in the event of COVID-19 disease following ChAdOx1 nCoV-19 vaccination are unknown. The NHP study conducted by NIH described above showed no evidence of immune-enhanced inflammation in ChAdOx1 nCoV-19 vaccinated animals who underwent SARS-CoV-2 challenge 4 weeks post immunisation, at 7 days post challenge. Results from a separate challenge study conducted on a purified inactivated SARS-CoV-2 vaccine also corroborate with NIH findings where no ADE has been detected in vaccinated animals [12]. However, the negative findings on ADE and lung immunopathology from both reports should be interpreted with caution, as challenged animals were sacrificed and examined shortly after challenge (7 days post inoculation). Further challenge studies on ChAdOx1 nCoV-19 vaccinated ferrets and NHPs with observation periods greater than 7 days after challenge are underway. These pre-clinical studies will report on presence or absence of lung pathology. Results will be reviewed as soon as they emerge and will inform discussions on risk/benefit to participants receiving the IMP. All pathology data arising from challenge studies of other SARS-CoV-2 vaccine candidates will also be taken into account.

3.3 Previous clinical experience

ChAdOx1 vectored vaccines expressing different inserts have previously been used in over 320 healthy volunteers taking part in clinical trials conducted by or in partnership with the University of Oxford in the UK and overseas (table 1 and 2). Most importantly, a ChAdOx1 vectored vaccine expressing the full-length Spike protein from another Betacoronavirus, MERS-CoV, has been given to 31 participants to date as part of MERS001 and MERS002 trials. ChAdOx1 MERS was given at doses ranging from $5x10^9$ vp to $5x10^{10}$ vp (table 2) with no serious adverse reactions reported. Further safety and immunogenicity results on ChAdOx1 MERS can be found on the Investigator’s Brochure for ChAdOx1 nCoV-19 for reference.

Clinical trials of ChAdOx1 vectored vaccines encoding antigens for Influenza (fusion protein NP+M1), Tuberculosis (85A), Prostate Cancer (5T4), Malaria (LS2), Chikungunya (structural

polyprotein), Zika (prM and E), MERS-CoV (full-length Spike protein) and Meningitis B are listed below.

None of the below mentioned clinical trials reported serious adverse events associated with the administration of ChAdOx1, which was shown to have a good safety profile.

Table 1. Clinical experience with ChAdOx1 viral vector vaccines.

Country	Trial	Vaccine	Age	Route	Dose	Number of Volunteers (Received ChAdOx1)	Publication / Registration Number
UK	FLU004	ChAdOx1 NP+M1	18-50	IM	$5x10^8$ vp	3	Antrobus et al, 2014. Molecular Therapy. DOI: 10.1038/mt.2013.284 [13]
					$5x10^9$ vp	3	
					$2.5x10^{10}$ vp	3	
					$5x10^{10}$ vp	6	
UK	FLU005	ChAdOx1 NP+M1<br>MVA NP+M1 (week 8)	18-50	IM	$2.5x10^{10}$ vp	12	Coughlan et al, 2018. EBioMedicine DOI: 10.1016/j.ebiom.2018.02.011 DOI: 10.1016/j.ebiom.2018.05.001 [14]
		ChAdOx1 NP+M1<br>MVA NP+M1 (week 52)	18-50	IM	$2.5x10^{10}$ vp	12	
		MVA NP+M1<br>ChAdOx1 NP+M1 (week 8)	18-50	IM	$2.5x10^{10}$ vp	12	
		MVA NP+M1<br>ChAdOx1 NP+M1 (week 52)	18-50	IM	$2.5x10^{10}$ vp	9	
		ChAdOx1 NP+M1	>50	IM	$2.5x10^{10}$ vp	12	
		ChAdOx1 NP+M1<br>MVA NP+M1 (week 8)	>50	IM	$2.5x10^{10}$ vp	12	
UK	TB034	ChAdOx1 85A	18-50	IM	$5x10^9$ vp	6	Wilkie et al, 2020 Vaccine

Country	Trial	Vaccine	Age	Route	Dose	Number of Volunteers (Received ChAdOx1)	Publication / Registration Number
					$2.5x10^{10}$ vp	12	DOI: 10.1016/j.vaccine.2019.10.102 [15]
		ChAdOx1 85A<br>MVA85A (week 8)	18-50	IM	$2.5x10^{10}$ vp	12	
		ChAdOx1 85A<br>(x2, 4weeks apart)<br>MVA85A (at 4 months)	18-50	IM	$2.5x10^{10}$ vp	12	
Switzerland	TB039<br>(ongoing)	ChAdOx1 85A	18-55	Aerosol	$1x10^{9}$ vp	3	Clinicaltrials.gov:<br>NCT04121494
				Aerosol	$5x10^{9}$ vp	3	
				Aerosol	$1x10^{10}$ vp	11	
				Aerosol/IM	$1x10^{10}$ vp	15	
Uganda	TB042<br>(ongoing)	ChAdOx1 85A	18-49	IM	$5x10^{9}$ vp	6	Clinicaltrials.gov:<br>NCT03681860
					$2.5 x10^{10}$	6	
UK	VANCE01	ChAdOx1.5T4<br>MVA.5T4	18 – 75	IM	$2.5x10^{10}$ vp	34	Clinicaltrials.gov:<br>NCT02390063
UK	ADVANCE	ChAdOx1.5T4	≥18	IM	$2.5x10^{10}$ vp	23 (as of Feb 20)	Clinicaltrials.gov:

Country	Trial	Vaccine	Age	Route	Dose	Number of Volunteers (Received ChAdOx1)	Publication / Registration Number
	(ongoing)	MVA.5T4					NCT03815942
UK	VAC067	ChAdOx1 LS2	18-45	IM	$5x10^9$ vp<br>$2.5x10^{10}$ vp	3<br>10	Clinicaltrials.gov:<br>NCT03203421
UK	VAMBOX	ChAdOx1 MenB.1	18-50	IM	$2.5x10^{10}$ vp<br>$5x10^{10}$ vp	3<br>26	ISRCTN46336916
UK	CHIK001	ChAdOx1 Chik	18-50	IM	$5x10^9$ vp<br>$2.5x10^{10}$ vp<br>$5x10^{10}$ vp	6<br>9<br>9	Clinicaltrials.gov:<br>NCT03590392<br>DOI: https://doi.org/10.4269/ajtmh.abstract2019<br>Abstract #59, page 19.
UK	ZIKA001<br>(ongoing)	ChAdOx1 Zika	18-50	IM	$5x10^9$ vp<br>$2.5x10^{10}$ vp<br>$5x10^{10}$ vp	6<br>3 (as of Feb 20)<br>-	Clinicaltrials.gov:<br>NCT04015648

Table 2. Clinical experience with ChAdOx1 MERS

Country	Trial	Vaccine	Age	Route	Dose	Number of Volunteers (Received ChAdOx1)	Publication / Registration Number
UK	MERS001 (ongoing)	ChAdOx1 MERS	18-50	IM	5×10^{9} vp<br>2.5×10^{10} vp<br>5×10^{10} vp<br>2.5×10^{10} vp (homologous prime-boost)	6<br>9<br>9<br>3	Clinicaltrials.gov: NCT03399578<br>Folegatti et.al. 2020, Lancet Infect.Dis<br>DOI: https://doi.org/10.1016/S1473-3099(20)30160-2 [16]
Saudi Arabia	MERS002 (ongoing)	ChAdOx1 MERS	18-50	IM	5×10^{9} vp<br>2.5×10^{10} vp<br>5×10^{10} vp	4<br>3<br>-	Clinicaltrials.gov: NCT04170829

3.4 Rationale

The COVID-19 epidemic has caused major disruption to healthcare systems with significant socioeconomic impacts. Containment measures have failed to stop the spread of virus, which has reached pandemic levels. There are currently no specific treatments available against COVID-19 and accelerated vaccine development is urgently needed.

Live attenuated viruses have historically been among the most immunogenic platforms available, as they have the capacity to present multiple antigens across the viral life cycle in their native conformations. However, manufacturing live-attenuated viruses requires complex containment and biosafety measures. Furthermore, live-attenuated viruses carry the risks of inadequate attenuation causing disseminated disease, particularly in immunocompromised hosts. Given that severe disease and fatal COVID-19 disproportionally affect older adults with co-morbidities, making a live-attenuated virus vaccine is a less viable option. Replication competent viral vectors could pose a similar threat for disseminated disease in the immuno-suppressed. Replication deficient vectors, however, avoid that risk while maintaining the advantages of native antigen presentation, elicitation of T cell immunity and the ability to express multiple antigens [17]. Subunit vaccines usually require the use of adjuvants and whilst DNA and RNA vaccines can offer manufacturing advantages, they are often poorly immunogenic requiring multiple doses, which is highly undesirable in the context of a pandemic.

Chimpanzee adenovirus vaccine vectors have been safely administered to thousands of people using a wide range of infectious disease targets. ChAdOx1 vectored vaccines have been given to over 320 volunteers with no safety concerns and have been shown to be highly immunogenic at single dose administration. Of relevance, a single dose of a ChAdOx1 vectored vaccine expressing full-length spike protein from another betacoronavirus (MERS-CoV) has shown to induce neutralising antibodies in recent clinical trials.

The use of an active comparator (MenACWY) will minimise the chances of accidental participant unblinding, decreasing bias in reactogenicity or safety reporting and/or health seeking behaviours once symptomatic for COVID-19.

The use of prophylactic paracetamol reduces the incidence and severity of fever and other adverse events following immunisation (AEFI). It has been previously recommended following Meningococcal B vaccine administration without negatively impacting its immunogenicity profile (reference: Bexsero

SmPC). Given the potential higher reactogenicity profile of ChAdOx1 nCoV-19 at $5x10^{10}$vp doses, a prophylactic paracetamol dose has been introduced in order to minimise severity of commonly observed local and systemic AEFI.

A batch comparison group (Group 5) has been included to assess potential differences in safety, reactogenicity and immunogenicity profiles across different ChAdOx1 nCoV-19 vaccine manufacturers.

Group 6 has been added to provide a comparison between efficacy at $5x10^{10}$vp dose on Abs260 and $5x10^{10}$ vp qPCR methods from different vaccine manufacturers.

Groups 7 and 8 have been added to provide safety, reactogenicity and immunogenicity data in older age groups receiving a $5x10^{10}$vp dose on qPCR, and replicate the study design in groups 1 and 2.

Group 4b has been added to provide immunogenicity data on homologous prime-boost at $5x10^{10}$vp (Abs260) prime and $2.2x10^{10}$vp (qPCR) boost, where up to 100 volunteers aged 18-55 initially recruited into group 4a will receive a booster dose of the vaccine 4-6 weeks apart.

Groups 4c and 6b have been added following interim immunogenicity results on homologous prime-boost groups showing improved neutralising antibody titres after 2 doses when compared to 1 dose regimen.

Groups 9 and 10 have been added as part of main safety and efficacy assessments in older age groups (56 – 69 years and 70 years and over) and removed from groups 4 and 6, as no vaccinations have been given to these age groups at the time of these group additions.

Group 11 has been added as an open-label and not randomised group to investigate the impact of previous ChAdOx1 vectored vaccines in immune responses elicited by ChAdOx1 nCoV-19.

Group 12 has been added as an open-label and not randomised group to investigate the safety and immunogenicity of ChAdOx1 nCoV-19 in people living with HIV.

3.4.1 Rationale for including older age groups

Deaths from COVID-19 infections are more common in adults aged 70 or older, and in those with pre-existing co-morbidities such as cardiovascular disease, diabetes, chronic respiratory disease, hypertension and cancer. SARS-CoV-2 infects children as well as adults and the elderly. However, COVID-19 infections in children are less severe and rarely result in death. It is the oldest age group

that is most at risk of death following natural infection, and in whom the vaccine would most likely be used first if deployed in a future public health campaign.

This study will recruit volunteers aged 56 to 70 years and those aged over 70 years. Simultaneously we will proceed to enrol a further up to 10,000 participants aged 18+ for a wide assessment of efficacy, with those over 55 years included in this larger cohort only as safety data become available from Group 1 and 2 cohorts.

3.4.2 Phase I/II study - COV001

The phase I/II study of efficacy, safety and immunogenicity of the ChAdOx1 nCoV19 vaccine (COV001, EudraCT 2020-001072-15) is the first evaluation of the vaccine in healthy adults aged 18-55 years in the UK started in April 2020. Over 1000 participants were enrolled and received either the investigational vaccine or a licensed MenACWY vaccine.

The two clinical studies are aligned in terms of study procedures and endpoints to allow data to be compared and combined across the two studies. The safety data from animal studies and from COV001 will be reviewed prior to vaccinating the first participant in COV002, and at each time point prior to expansion into additional age groups. See section 5 for further details.

3.4.3 Rationale for including HIV Infected persons

People living with HIV may have less functional immunity and have more associated co-morbidities than the general population. Indeed the chronic immune activation and inflammation observed in HIV-infected patients has been associated with poor antibody (Ab) responses to vaccines against influenza and HAV/HBV [18,19]. Evaluating immunological outcomes to the ChAdOx1 nCoV-19 vaccine allows us to assess whether responses are the same as in a matched HIV negative cohort, facilitating global policy on vaccine implementation in areas of high HIV prevalence

4 OBJECTIVES AND ENDPOINTS

	Objective	Outcome Measure	Timepoint of evaluation
**Primary**	To assess efficacy of the candidate ChAdOx1 nCoV-19 against COVID-19 in adults aged 18 years and older.	Virologically confirmed (PCR* positive) symptomatic cases of COVID-19	Throughout the study
**Co-Primary**	To assess the safety of the candidate vaccine ChAdOx1 nCoV-19 in adults .	Occurrence of serious adverse events (SAEs) throughout the study duration.	Throughout the study
**Secondary**	To assess the safety, tolerability and reactogenicity profile of the candidate vaccine ChAdOx1 nCoV-19	a) occurrence of solicited local reactogenicity signs and symptoms for 7 days following vaccination;<br>b) occurrence of solicited systemic reactogenicity signs and symptoms for 7 days following vaccination;<br>c) occurrence of unsolicited adverse events (AEs) for 28 days following vaccination ;<br>d) change from baseline for safety laboratory measures and (except groups 4, 6, 9 and 10);<br>e) Occurrence of disease enhancement episodes	a) Day 0-7 Self-reported symptoms recorded using electronic diaries<br>b) Day 0-7 Self-reported symptoms recorded using electronic diaries<br>c) Day 0-28 Self-reported symptoms recorded using electronic diaries<br>d) See schedule of attendances<br>e) Throughout the study

	To assess efficacy of the candidate ChAdOx1 nCoV-19 against severe and non-severe COVID-19	a) Hospital admissions associated with COVID-19<br>b) Intensive care unit (ICU) admissions associated with COVID-19<br>c) Deaths associated with COVID-19<br>d) Seroconversion against non-Spike SARS-CoV-2 antigens<br>e) Severe COVID-19 disease (defined according to clinical severity scales)	Throughout the study<br><br>See schedule of attendances<br><br>Throughout the study
	To assess humoral immunogenicity of ChAdOx1 nCoV-19	a) Antibodies against SARS-CoV-2 spike protein at Day 28 post-vaccination.<br>b) Proportion of seroconversion to antibodies against SARS-CoV-2 spike protein measured by ELISA at Day 28 post-vaccination.	Blood samples drawn at Day 0 and Day 28 post-vaccination
	To assess cellular immunity of ChAdOx1 nCoV-19 in older adults (groups 1, 2, 7 and 8 only)	a) Interferon-gamma (IFN-γ) enzyme-linked immunospot (ELISpot) responses to SARS-CoV-2 spike protein;	See schedule of attendances
	To assess the safety and immunogenicity of a booster dose of ChAdOx1 nCoV-19 in older adults aged 56 years or older (two-dose	a) occurrence of solicited local reactogenicity signs and symptoms for 7 days following booster vaccination;	a) Day 28-35 Self-reported symptoms recorded using electronic diaries

	schedules for groups 1, 2, 7 and 8 only)	b) occurrence of solicited systemic reactogenicity signs and symptoms for 7 days following booster vaccination;<br>c) occurrence of unsolicited adverse events (AEs) for 28 days following booster vaccination;<br>d) change from pre-booster for safety laboratory measures and;<br>e) Occurrence of disease enhancement episodes<br>f) Antibodies against SARS-CoV-2 spike protein at Day 56 post-vaccination.<br>g) Proportion of seroconversion to antibodies against SARS-CoV-2 spike protein from baseline at Day 56 post-vaccination.	b) Day 28-35 Self-reported symptoms recorded using electronic diaries<br>c) Day 28-56 Self-reported symptoms recorded using electronic diaries<br>d) See schedule of attendances<br>e) Throughout the study<br>f) Blood samples drawn at day 0, 28 and at day 56.<br>g) Blood samples drawn at day 0, 28 and at day 56.
Tertiary	Exploratory Immunology	a) virus neutralising antibody (NAb) assays against live and/or pseudotype SARS-CoV-2 virus<br>b) Cell analysis by flow cytometry assays<br>c) Functional antibody assays<br>d) Anti-vector immunity induced by 1 or 2 doses of ChAdOx1 nCoV-19	See schedule of attendances

	Exploratory efficacy against infection<br>• To assess efficacy of the candidate ChAdOx1 nCoV-19 against SARS-CoV-2 infection	a) PCR* positive SARS-CoV-2 asymptomatic infection<br>b) Differences in viral loads between those with severe, mild, and asymptomatic PCR+* SARS-CoV-2 infections.	Throughout the study
	Measure exposure to COVID-19	Reported by weekly survey to collect information about cases amongst household contacts and friends, contact with the general public, infection control procedures	Weekly throughout the study
	Compare safety, reactogenicity and immunogenicity between different manufacturing batches of ChAdOx1 nCoV-19 used in COV001 and COV002	Differences in safety, reactogenicity and immunogenicity profiles between Group 1 in COV001 and Group 5 in COV002 (proportion of Grade 3 solicited AEs, occurrence of fevers, seroconversion rates, neutralising antibody titres and differences in T-cell responses.	Day 0-7 for solicited AEs<br><br>D28 for seroconversion rates and neutralising antibodies<br><br>D14 for T-cell immunology readouts
	Compare safety, reactogenicity and immunogenicity between different methods for measuring doses (Abs260, Abs 260 corrected for PS80, and qPCR) of ChAdOx1 nCoV-19	Differences in safety, reactogenicity and immunogenicity profiles between Groups 1, 2, and 5A compared with and Groups, 7, 8 and 5B, C and D respectively (proportion of Grade 3 solicited AEs, occurrence of fevers, seroconversion	Day 0-7 for solicited AEs<br><br>D28 for seroconversion rates and neutralising antibodies

		rates at D28, neutralising antibody titres and differences in T-cell responses at D14).	D14 for T-cell immunology readouts
	To assess vaccine induced mucosal immunity	Differences in IgA levels in nasal mucosa in a subset of individuals	at D0 and D28 post vaccination
	To compare viral shedding on stool samples of SARS-CoV-2 PCR* positive individuals	Differences in viral shedding on stool between vaccine and comparator arms	At approximately 7 days and beyond post SARS-CoV-2 PCR* positivity.
	To compare immunogenicity of ChAdOx1 nCoV-19 in participants receiving 1 or 2 doses (groups 1, 2, 7 and 8)	a) Differences in antibody titres (ELISA and Neutralising antibodies) in participants who received 1 or 2 doses of ChAdOx1 nCoV-19 (groups 1, 2, 7 and 8)<br>b) Longevity of immune responses in participants who received 1 or 2 doses of ChAdOx1 nCoV-19 (groups 1, 2, 7 and 8)	a) At 28 days post prime and 28 days post boost<br>b) At 6 and 12 months post prime (prime only) and 6 and 12 months post boost
	To describe the impact of previous vaccination with other ChAdOx1 vectored vaccines in immune responses to ChAdOx1 nCoV-19	Differences in antibody titres and T-cell responses between groups 5d and 11 and their relationship with anti-vector neutralising antibody titres.	At day 14 post ChAdOx1 nCoV-19 prime (T-cell responses), day 28 post prime and day 28 post boost.

	To assess the cell-mediated and humoral immunogenicity profile of ChAdOx1 nCoV-19 vaccine in HIV infected adults	Cell-mediated and humoral responses against SARS-Cov-2 These will be measured by the following:<br>a) Proportion of seroconversion to antibodies (Ab) against SARS-CoV-2 spike protein measured by ELISA.<br>b) Interferon-gamma enzyme linked immunospot (ELISpot) responses to SARS-CoV-2 spike protein<br>c) Intracellular Cytokine analyses of CD4 and CD8-specific SARS-CoV-2 spike protein responses<br>d) Further exploratory immunology	a) at all exploratory immunology timepoints described in the schedule of attendances<br>b) at all exploratory immunology timepoints described in the schedule of attendances<br>c) at all exploratory immunology timepoints described in the schedule of attendances<br>d) at all exploratory immunology timepoints described in the schedule of attendances

	To assess whether increasing age and or CD4 nadir are associated with a lack of immune response in HIV infected adults	a) Nadir CD4 count<br>b) Age at enrolment<br>c) Immune responses to ChAdOx1 nCoV-19 (assessed as described above)	a) at all exploratory immunology timepoints described in the schedule of attendances
	To assess the safety of the candidate vaccine ChAdOx1 nCoV-19 in HIV infected adults	a) Occurrence of serious adverse events (SAEs) throughout the study duration<br>b) occurrence of solicited local reactogenicity signs and symptoms for 7 days following vaccination<br>c) occurrence of solicited systemic signs and symptoms for 7 days following each vaccination<br>d) occurrence of unsolicited AEs for 28 days following each vaccination	a) Throughout the study<br>b) Day 0-7 post prime and boost<br>c) Day 0-7 post prime and boost<br>d) Day 0-28 post prime and boost
	To assess Impact of vaccination HIV reservoirs	Change in Total HIV DNA copies per million CD4 T cells	a) Throughout the study

	To assess immunological correlates of protection in relation to occurrence of COVID-19 disease in ChAdOx1 nCoV-19 recipients	Immunological endpoints and COVID-19 disease endpoints in ChAdOx1 nCoV-19 recipients	Throughout the study

*or other nucleic acid amplification test

Sample analysis for the completion of exploratory endpoints may be performed under the ethically approved OVC Biobank protocol.

5 TRIAL DESIGN

This is a Phase 2/3, participant-blinded individually randomised controlled trial in adults in the UK, administering either a single dose or two-doses of ChAdOx1 nCoV-19 or licensed MenACWY vaccine via IM injection. Additional steps may be taken to keep clinical investigators assessing the primary efficacy endpoint blinded to group allocation, where this is possible and practical to do so. All data from participants with PCR (or other nucleic acid amplification test) -positive swabs will be assessed for inclusion in the primary efficacy analysis by two blinded assessors who will independently review each case according to pre-specified criteria as detailed in the statistical analysis plan, to classify each for inclusion in the primary and secondary outcomes. *Protocol V19.0:* Exploratory COVID-19 disease endpoints may be assessed by trained study site staff following pre-specified criteria, rather than independent blinded assessors.

After review of all available data from animal studies and at least 4 weeks safety and immunogenicity data from the first 54 participants receiving ChAdOx1 nCoV-19 in COV001, following DSMB review, enrolment into Groups 1, 4, 5 & 6 will commence. A minimum of 7 days safety data from group 1 will be reviewed by the DSMB prior to enrolment of participants into group 2. Participants will be randomised to ChAdOx1 nCoV-19/MenACWY on a 3:1:3:1 ratio in groups 1 and 7, and 5:1:5:1 ratio in groups 2 and 8, 1:1 in groups 4, 5a, 5b, 5c, 6, 9 and 10, and 5:1 in group 5d. Groups 5e and 5f will be open label and therefore, volunteers will not be randomised. Participants in groups 4, 6, 9 and 10 will be advised to take prophylactic paracetamol for 24 hours (1000 mg every 4-6 hours) from the time of vaccination to reduce the likelihood of fever. The sequence of enrolment of participants over the age of 56 years is outlined in section 7.3.2.2. Up to 100 volunteers in group 4 will be invited to receive a booster dose of $2.2x10^{10}$vp (qPCR) 4-6 weeks after prime. All remaining volunteers in group 4 and all participants in group 6 will be invited to receive a booster dose of 0.5mL ($3.5 – 6.5 \times 10^{10}$ vp, Abs 260, corrected for PS80) at least 4 weeks after prime. Participants who were originally randomised to receive a single dose in groups 1 (a1 and a2), 2 (a1 and a2) and 5 (a1 and a2) will be invited to receive a booster dose of 0.5mL ($3.5 – 6.5 \times 10^{10}$ vp, Abs 260, corrected for PS80) at the earliest available opportunity, with a minimum 4 weeks interval from prime.

Safety will be assessed in real time. The DSMB will periodically assess safety and efficacy data every 4-8 weeks and/or as required.

Participants will be followed over the duration of the study to record adverse events and episodes of virologically confirmed symptomatic COVID-19 cases. Participants will be tested for COVID-19 if they present with a new onset of fever (≥37.8 C) OR cough OR shortness of breath OR anosmia/ageusia.

Weekly testing for PCR+ infection with SARS-CoV-2 using home test kits will also be undertaken in partnership with the Department of Health and Social care national community testing programme, subject to testing resource availability.

Moderate and Severe COVID-19 disease will be defined using clinical criteria. Detailed clinical parameters will be collected from medical records and aligned with agreed definitions as they emerge. These are likely to include, but are not limited to, oxygen saturation, need for oxygen therapy, respiratory rate and other vital signs, need for ventilatory support, Xray and CT scan imaging and blood test results, amongst other clinically relevant parameters.

Accumulated safety data from COV001 will be reviewed before commencing enrolment.

To account for the multisite recruitment activity, it is recognised that the number of volunteers enrolled into each group 9 and 10 will be 1000 +/-10%.

<u>HIV -Group 12: open-label</u>

This is a single arm group whereby up to 60 HIV infected individuals who are stable on antiretroviral therapy (ARV) will be recruited and receive ChAdOx1 nCoV-19 vaccination according to the schedule of attendance described in table 14.

5.1 Study groups

**Group**	**Vaccine**	**Number of Volunteers**	**Age group of volunteers**
**Randomised groups**			
Group 1 ****	a1) Single dose ChAdOx1 nCOV19 vaccine, 5x1010vp (Abs 260)*, OR<br>a2) Single dose MenACWY<br>a3) Two-dose ChAdOx1 nCoV-19 5x1010vp (Abs 260) prime and 0.5mL	N=30<br><br>N=10<br>N= up to 30 from 1a1	Adults aged 56 – 69 years

	(3.5 – 6.5 × 1010 vp, Abs 260, corrected for PS80) boost*, OR<br>a4) Two-dose MenACWY,<br><br>b1) Two dose ChAdOx1 nCOV19 vaccine, 5x1010vp (Abs 260) prime and 2.2x1010vp (qPCR) boost* (4-6 weeks apart), OR<br>b2) Two-dose MenACWY (4-6 weeks apart)	N= up to 10 from 1a2<br>N=30<br><br>N=10	
Group 2****	a1) Single dose ChAdOx1 nCOV19 vaccine, 5x1010vp (Abs 260)*, OR<br>a2) Single dose MenACWY (4-6 weeks apart)<br>a3) Two-dose ChAdOx1 nCoV-19 5x1010vp (Abs 260) prime and 0.5mL (3.5 – 6.5 × 1010 vp, Abs 260, corrected for PS80) boost*, OR<br>a4) Two-dose MenACWY<br>b1) Two dose ChAdOx1 nCOV19 vaccine, 5x1010vp (Abs 260) prime and 2.2x1010vp (qPCR) boost * (4-6 weeks apart), OR<br>b2) Two-dose MenACWY	N=50<br>N=10<br>N= up to 50 from 2a1<br>N= up to 10 from 2a2<br>N=50<br>N=10	Adults aged 70 years or older
Group 4**<br>(n= up to 3550)	a1) Single dose ChAdOx1 nCoV19 vaccine, 5x10^{10}vp (Abs 260)*OR<br>a2) MenACWY<br><br>b1) Two dose ChAdOx1 nCOV19 vaccine, 5x10^{10}vp (Abs260) prime and 2.2x10^{10}vp (qPCR) boost* (4-6 weeks apart) OR	N=up to 1775<br>N=up to 1775<br><br>N= up to 50 (from 4a1)	Adults aged 18 – 55 years

	b2) Two dose MenACWY<br><br>c1) Two dose ChAdOx1 nCOV19 vaccine, 5x10^{10}vp (Abs260) prime and 0.5mL (3.5 – 6.5 × 10^{10} vp, Abs 260, corrected for PS80) boost* OR 5x10^{10}vp (qPCR) boost (at least 4 weeks apart) OR<br><br>c2) Two dose MenACWY	N= up to 50 (from 4a2)<br><br>N= up to 1725 (from 4a1)<br><br>N= up to 1725 (from 4a2)	
Group 5****	a1) Single dose ChAdOx1 nCoV19 vaccine, 5x10^{10}vp, (Abs 260)* OR<br>a2) MenACWY<br><br>a3) Two-dose ChAdOx1 nCoV-19 5x1010vp (Abs 260) prime and 0.5mL (3.5 – 6.5 × 1010 vp, Abs 260, corrected for PS80) boost*<br><br>a4) Two-dose MenACWY<br><br>b1) Single dose ChAdOx1 nCoV19 vaccine, 5x10^{10}vp, (qPCR)* OR<br><br>b2) Men ACWY MenACWY<br>(B-cell immunology only)<br><br>c1) Single dose ChAdOx1 nCoV19 vaccine, 5x10^{10}vp, (qPCR)* OR<br><br>c2) MenACWY<br>(B and T-cell immunology)<br><br>d1) Two-dose ChAdOx1 nCoV19 vaccine, 0.5mL (3.5 – 6.5 × 10^{10} vp, Abs 260,	N= 50<br><br>N=50<br><br>N = up to 50 from 5a1<br><br>N = up to 50 from 5a2<br><br>N= up to 25<br><br>N= up to 25<br><br>N= up to 25<br><br>N= up to 25<br><br>N= up to 50	Adults aged 18-55 years

	corrected for PS80)*, (4-6 weeks apart) OR<br>d2) Men ACWY<br><br>e) Two-dose ChAdOx1 nCoV-19 vaccine (Covishield 0.9 x 10^11 vp/mL), 0.5mL (4-6 weeks apart)<br>f) Two-dose ChAdOx1 nCoV-19 vaccine (Covishield 0.9 x 10^11 vp/mL), 0.25mL prime and 0.5mL boost (4-6 weeks apart)	N= up to 10<br><br>N= up to 15<br><br>N= up to 15	
Group 6***<br>(n= up to 6000)	a1) ChAdOx1 nCoV19 vaccine, $5x10^{10}$vp (qPCR)* OR<br>a2) MenACWY<br>b1) Two dose ChAdOx1 nCoV-19 vaccine, $5x10^{10}$vp (qPCR) prime and 0.5mL (3.5 – 6.5 × 10^{10} vp, Abs 260, corrected for PS80) boost* OR $5x10^{10}$vp (qPCR) boost* (at least 4 weeks apart) OR<br>b2) Two dose MenACWY	N = up to 3000<br><br>N = up to 3000<br><br>N = up to 3000 (from 6a1)<br><br>N = up to 3000 (from 6a2)	Adults aged 18 – 55 years
Group 7	a1) Single dose ChAdOx1nCOV19 vaccine, $5x10^{10}$vp (qPCR)*, OR<br>a2) Single dose MenACWY<br>b1) Two dose ChAdOx1nCOV19 vaccine, $5x10^{10}$vp (qPCR)* (4-6 weeks apart), OR<br>b2) Two-dose MenACWY (4-6 weeks apart)	N=30<br><br>N=10<br><br>N=30<br><br>N=10	Adults aged 56 – 69 years

Group 8	a1) Single dose ChAdOx1nCOV19 vaccine, $5x10^{10}$vp (qPCR)*, OR<br>a2) Single dose MenACWY<br>b1) Two dose ChAdOx1nCOV19 vaccine, $5x10^{10}$vp (qPCR) prime and 0.5mL (3.5 – 6.5 × 10^{10} vp, Abs 260, corrected for PS80) boost* OR $5x10^{10}$vp (qPCR) boost (4-6 weeks apart), OR<br>b2) Two-dose MenACWY (4-6 weeks apart)	N=50<br>N=10<br>N=50<br>N=10	Adults aged 70 years or older
Group 9	a1)Two dose ChAdOx1 nCOV19 vaccine, 0.5mL (3.5 – 6.5 × 10^{10} vp, Abs 260, corrected for PS80)* (4-6 weeks apart) OR<br>a2) Two dose MenACWY	N= approx. 500<br>N= approx. 500	Adults aged 56 – 69 years
Group 10	a1)Two dose ChAdOx1 nCOV19 vaccine, 0.5mL (3.5 – 6.5 × 10^{10} vp, Abs 260, corrected for PS80)* (4-6 weeks apart) OR<br>a2) Two dose MenACWY	N= approx. 500<br>N= approx. 500	Adults aged 70 years or older
Group 11	Two dose ChAdOx1 nCOV19 vaccine, 0.5mL (3.5 – 6.5 × 10^{10} vp, Abs 260, corrected for PS80)* (4-6 weeks apart)	N=up to 60	Adults aged 18-55 who previously received a ChAdOx1 vectored vaccine.
Group 12	Two dose ChAdOx1 nCOV19 vaccine, 0.5mL (3.5 – 6.5 × 10^{10} vp, Abs 260, corrected for PS80)* (4-6 weeks apart)	N=up to 60	HIV positive adults aged 18-55

* See section 8.5 for further information on dosing

** A subset of up to 100 participants in group 4a will be invited to receive a booster dose in 4b, keeping the overall sample size in group 4 the same. All remaining participants in group 4a will be invited to receive a booster dose in 4c, keeping the overall sample size in group 4 the same.

*** Participants in group 6a will be invited to receive a booster dose in 6b, keeping the overall sample size in group 6 the same

**** Participants in groups 1a (a1 and a2), 2a (a1 and a2) and 5a (a1 and a2) will be invited to receive a booster dose in the respective a3 and a4 groups, keeping the overall sample size in group 1a, 2a and 5a the same.

5.2 Trial volunteers

Adult volunteers aged at least 18 years, will be recruited into the study. Volunteers will be considered enrolled immediately following administration of the vaccine.

5.3 Definition of End of Trial

The end of the trial is the date of the last assay conducted on the last sample collected.

5.4 Potential Risks for volunteers

The potential risks are those associated with phlebotomy, vaccination and disease enhancement

Venepuncture

Adult Groups

Localised bruising and discomfort can occur at the site of venepuncture. Infrequently fainting may occur. These will not be documented as AEs if they occur. The total volume of blood drawn over a 12 month period will be 105-687.5mL in the adult groups (blood volumes may vary slightly for volunteers at different investigator sites due to use of different volume vacutainers, following local Trust SOPs). The total volume of blood drawn over a 12 month period in the HIV group will be 1077.5mL. This should not compromise these otherwise healthy volunteers, as they would donate 470mL during a single blood donation for the National Blood transfusion Service over a 3-4 month period.

As of SA23 (protocol V18.0), participants will be permitted to donate blood to transfusion services from 3 months after the date of their final planned study vaccination. This replaces the previous instruction that volunteers should refrain from blood donation for the duration of their involvement in the trial. 3 months has been selected to provide adequate spacing between study visits and blood

donation in order to remain within the recommended limits for blood donation in the UK and to comply with the Joint United Kingdom (UK) Blood Transfusion and Tissue Transplantation Services Professional Advisory Committee requirement for a minimum period of 7 days between ChAdOx1 nCov-19 vaccination and blood donation (Joint United Kingdom (UK) Blood Transfusion and Tissue Transplantation Services Professional Advisory Committee: Change Notification UK National Blood Services No. 73 - 2020).

Allergic reactions

Allergic reactions from mild to severe may occur in response to any constituent of a medicinal product's preparation. Anaphylaxis is extremely rare (about 1 in 1,000,000 vaccine doses) but can occur in response to any vaccine or medication.

Vaccination

Local reaction from IM vaccination

The typical local reaction as a result of IM injection is temporary pain, tenderness, redness, and swelling at the site of the injection.

Systemic reactions

Constitutional influenza-like symptoms such as fatigue, headache, malaise, feverishness, and muscle aches can occur with any vaccination and last for approximately 2-3 days. In the phase 1 COV001 study, approximately 30-40% of participants not taking prophylactic paracetamol felt feverishness, or had chills, muscle ache, malaise, fatigue, or headache which they rated as moderate to severe. (See the investigator brochure for further details). Presyncopal and syncopal episodes may occur at the time of vaccination which rapidly resolve. As with many vaccines, temporary ascending paralysis (Guillain-Barré syndrome, GBS) or immune mediated reactions that can lead to organ damage may occur, but this should be extremely rare (1 in 100,000-1,000,000 vaccine doses).

Transient neutropenia, lymphopenia and thrombocytopenia has been described following immunization with other adenoviral-vectored vaccines, and is not perceived to be of clinical significance.

Control participants will receive one or two doses of a licensed MenACWY vaccine, the risks of which are described in these vaccines SmPC.

Disease Enhancement

The risks of inducing disease enhancement and lung immunopathology in the event of COVID-19 disease following ChAdOx1 nCoV-19 vaccination are unknown as described above. Two NHP challenge studies have shown no evidence of disease enhancement from immunisation with ChAdOx1 nCoV-19 or inactivated SARS-CoV-2 virus, but caution should be taken when interpreting these negative findings. All pre-clinical data from challenge studies using ChAdOx1 nCoV-19 and other vaccine candidates (when available) will inform decisions on risk/benefit to participants receiving the IMP. Any safety signals associated with disease enhancement potentially observed in COV001 will also inform these decisions.

Emerging Thrombosis with Thrombocytopenia Association with vaccination

The MHRA and JCVI issued updated guidance regarding the use of ChAdOx1 nCoV-19 on 7th April 2021, following a review of extremely rare reports of cerebral venous sinus thrombosis (and thrombosis of other major veins) with concurrent thrombocytopenia that have occurred after vaccination in the national rollout programme. This recommends that currently, in the UK setting, alternative vaccinations against COVID-19 should be preferentially offered to individuals aged 29 and under.

Prior to any further participant receiving ChAdOx1 nCoV-19 in this trial, they will be provided with up-to-date information from regulators on this finding via the updated participant information sheet. They will also be provided with other relevant documentation from regulators and/or public health authorities related to this association and possible risks of vaccination that is also being provided in vaccination centres. Participants will be advised to be aware of possible signs and symptoms of blood clots and to have a low threshold to contact trial teams if experiencing these or other symptoms.Following this recommendation, no further ChAdOx1 nCoV-19 prime vaccination doses will be administered to participants aged 29 and under within this trial. This age restriction recommendation does not apply to booster vaccinations which may therefore continue to be administered within the trial to participants of any age.

5.5 Known Potential Benefits

Volunteers enrolled into the control groups will receive 1 or 2 doses of MenACWY, a licensed vaccine that has been administered to teenagers in the UK routine schedule since 2015 and is used as a travel vaccine for high risk areas. The majority of participants in this study will not have had this vaccine previously, and therefore will gain the benefit of protection against group A, C, W and Y meningococcus. Those participants who have previously had MenACWY vaccines will have their immunity against these organisms boosted. Recipients of ChAdOx1 nCoV-19 do not have any guaranteed benefit, however it is hoped that the information gained from this study will contribute to the development of a safe and effective vaccine against COVID-19.

6 RECRUITMENT AND WITHDRAWAL OF TRIAL VOLUNTEERS

6.1 Identification of Trial Volunteers

Volunteers will be recruited by use of an advertisement +/- registration form formally approved by the ethics committee(s) and distributed or posted in the following places:

- In public places, including buses and trains, with the agreement of the owner / proprietor.
- In newspapers or other literature for circulation.
- On radio via announcements.
- On a website or social media site operated by our group or with the agreement of the owner or operator (including on-line recruitment through our website).
- By e-mail distribution to a group or list only with the express agreement of the network administrator or with equivalent authorisation.
- By email distribution to individuals who have already expressed an interest in taking part in any clinical trial at the Oxford Vaccine Centre and at trial sites.
- On stalls or stands at exhibitions or fairs.
- Via presentations (e.g. presentations at lectures or invited seminars).
- Direct mail-out: This will involve obtaining names and addresses of adults via the most recent Electoral Roll. The contact details of individuals who have indicated that they do not wish to

receive postal mail-shots would be removed prior to the investigators being given this information. The company providing this service is registered under the General Data Protection Regulation 2016/679. Investigators would not be given dates of birth or ages of individuals but the list supplied would only contain names of those aged ≥18 years (as per the inclusion criteria).

- Direct mail-out using National Health Service databases: These include the National Health Applications and Infrastructure Services (NHAIS) via a NHAIS data extract or equivalent. Initial contact to potential participants will not be made by the study team. Instead study invitation material will be sent out on our behalf by an external company, CFH Docmail Ltd, in order to preserve the confidentiality of potential participants. CFH Docmail Ltd is accredited as having exceeded standards under the NHS Digital Data Security and Protection Toolkit (ODS ID – 8HN70).
- Oxford Vaccine Centre databases and study site databases: We may contact individuals from databases of groups within the CCVTM (including the Oxford Vaccine Centre database) and other study sites of previous trial participants who have expressed an interest in receiving information about all future studies for which they may be eligible.
- Using local GP practices or Trusts as Participant Identification Centres (PICs)

Recruitment of those with likely higher exposure to SARS-CoV-2 will be prioritised, in order to increase the likelihood of obtaining efficacy endpoints in the context of a waning epidemic. These priority groups will mainly consist of, but are not limited to, COVID-19 patient facing frontline healthcare workers (e.g. those working in ICU, A&E, COVID-19 wards, Paramedics, Care Homes, GP COVID-19 hubs, dentists, COVID-19 testing centres), non-healthcare staff working in COVID-19 clinical areas (e.g. hospital porters, receptionists, cleaners, other hospital workers), and other public facing keyworkers with no access to personal protective equipment, amongst others.

6.2 Informed consent

Participants will personally sign and date the latest approved version of the Informed Consent form. A written version and verbal explanation of the Study Information leaflet and Informed Consent will be presented to the participant/parent/legal guardian of the participant detailing:

- the exact nature of the study

• what it will involve for the participant

• the implications and constraints of the protocol

• the known side effects and any risks involved in taking part

• sample handling – participants will be informed that anonymised samples taken during the course of the study may be shared with study collaborators.

• Individual results will not be shared with participants

The Study Information leaflet will be made available to the participant and/or parent/legal guardian for an appropriate amount of time (where possible this will a minimum of 24 hours) prior to consent being obtained. A video presentation of the Study Information leaflet may be screened to an audience, or made available for them to access it remotely. However, participants will have the opportunity to individually question an appropriately trained and delegated researcher before signing consent.

The following general principles will be emphasised:

- Participation in the study is entirely voluntary
- Refusal to participate involves no penalty or loss of medical benefits
- The volunteer may withdraw from the study at any time.
- The volunteer is free to ask questions at any time to allow him or her to understand the purpose of the study and the procedures involved
- The study involves research of an investigational vaccine
- There is no direct benefit to the volunteer from participating
- The volunteer's GP will be contacted to corroborate their medical history (Groups 1, 2 , 7 and 8 only, except group 12 where GPs can be replaced by their HIV consultant). Written or verbal information regarding the volunteer's medical history will be sought from the GP or other sources. This can either be via the study team accessing patient's electronic care summaries from local systems, by contacting the GP practice, or volunteers bringing their medical care summaries from the GP to the study clinicians. However, volunteers in all remaining groups may be enrolled based on medical information obtained during screening and/or enrolment visit, at the physician's discretion.

- Blood samples taken as part of the study may be sent outside of the UK and Europe to laboratories in collaboration with the University of Oxford. These will be de-identified. Volunteers will be asked if they consent to indefinite storage of any leftover samples for use in other ethically approved research, this will be optional.

Participants will be allowed as much time as wish to consider the information, and the opportunity to question the Investigator, their GP or other independent parties to decide whether they will participate in the study. Written informed consent will then be obtained by means of the adult participant or the parent/legal guardian of the participant dated signature, and dated signature of the person who presented and obtained the Informed consent. The person who obtained the consent must be suitably qualified and experienced, and have been authorised to do so by the Chief/Principal Investigator and listed on the delegation log. A copy of the signed informed consent will be given to the participant. The original signed form will be retained at the research study site, in the case report form (CRF).

Updated information that require volunteers to be re-consented will be sent to participants and written re-consent requested at the earliest scheduled visit. If the earliest visit to occur is in the symptomatic pathway, the participant may consent using an electronic signature for infection control purposes. Where appropriate, and when re-consenting in person is not possible (e.g. participants in self-isolation), volunteers may be contacted over the phone and an appropriately trained and delegated researcher will obtain re-consent. In this instance the re-consent discussion will be documented by the researcher, the participant will sign the form (electronic or paper) and a copy will be signed by the researcher. The dates of signature may be different and a fully signed copy will be provided to the participant at the next scheduled visit. The participant may re-consent using an electronic signature.

6.3 Inclusion and exclusion criteria

This study will be conducted in adults who meet the following inclusion and exclusion criteria:

6.3.1 Inclusion Criteria

The volunteer must satisfy all the following criteria to be eligible for the study:

- Adults aged 18 - 55 years (groups 4, 5, 6 and 11)
- Adults aged 56-69 years (groups 1, 7, and 9)

- Adults aged 70 years and older (groups 2, 8, and 10)
- Able and willing (in the Investigator's opinion) to comply with all study requirements.
- Willing to allow the investigators to discuss the volunteer's medical history with their General Practitioner and access all medical records when relevant to study procedures.
- For females of childbearing potential only, willingness to practice continuous effective contraception (see below) during the study and a negative pregnancy test on the day(s) of screening and vaccination.
- Agreement to refrain from blood donation during the course of the study.
- Provide written informed consent.

Additional Inclusion criteria to Group 12 (HIV sub-study):

- HIV positive
- Receiving antiretroviral therapy
- Undetectable HIV viral load
- CD4>350 cells/mL

6.3.2 Exclusion Criteria

The volunteer may not enter the study if any of the following apply:

- Participation in COVID-19 prophylactic drug trials for the duration of the study.

  Note: Participation in COVID-19 treatment trials is allowed in the event of hospitalisation due to COVID-19. The COV002 study team should be informed as soon as possible.

- Participation in SARS-CoV-2 serological surveys where participants are informed of their serostatus for the duration of the study.

  Note: Disclosure of serostatus post enrolment may accidently unblind participants to group allocation. Participation in COV002 can only be allowed if volunteers are kept blinded to their serology results from local/national serological surveys

- Receipt of any vaccine (licensed or investigational) other than the study intervention within 30 days before and after each study vaccination, with the exception of the licensed seasonal

influenza vaccination and the licensed pneumococcal vaccination. Participants will be encouraged to receive these vaccinations at least 7 days before or after their study vaccine.

- Prior or planned receipt of an investigational or licensed vaccine or product likely to impact on interpretation of the trial data (e.g. Adenovirus vectored vaccines, any coronavirus vaccines). This exclusion criteria will not apply to group 11, as recruitment will be targeted at those volunteers who previously received a ChAdOx1 vectored vaccine.
- Administration of immunoglobulins and/or any blood products within the three months preceding the planned administration of the vaccine candidate.
- Any confirmed or suspected immunosuppressive or immunodeficient state (except group 12, where HIV infected participants are allowed); asplenia; recurrent severe infections and use of immunosuppressant medication within the past 6 months, except topical steroids or short-term oral steroids (course lasting ≤14 days)
- History of allergic disease or reactions likely to be exacerbated by any component of ChAdOx1 nCoV-19 or MenACWY
- Any history of angioedema.
- Any history of anaphylaxis.
- Pregnancy, lactation or willingness/intention to become pregnant during the study.
- Current diagnosis of or treatment for cancer (except basal cell carcinoma of the skin and cervical carcinoma in situ).
- History of serious psychiatric condition likely to affect participation in the study.
- Bleeding disorder (e.g. factor deficiency, coagulopathy or platelet disorder), or prior history of significant bleeding or bruising following IM injections or venepuncture.
- Continuous use of anticoagulants, such as coumarins and related anticoagulants (i.e. warfarin) or novel oral anticoagulants (i.e. apixaban, rivaroxaban, dabigatran and edoxaban)
- Suspected or known current alcohol or drug dependency.
- Any other significant disease, disorder or finding which may significantly increase the risk to the volunteer because of participation in the study, affect the ability of the volunteer to participate in the study or impair interpretation of the study data.
- Severe and/or uncontrolled cardiovascular disease, respiratory disease, gastrointestinal disease, liver disease, renal disease, endocrine disorder and neurological illness (mild/moderate well controlled comorbidities are allowed)

- History of laboratory confirmed COVID-19 (except groups 5d, 5e, 5f, 9, 10 and 11).
  - Seropositivity to SARS-CoV-2 before enrolment (except groups 5d, 5e, 5f, 9, 10 and 11)
  - NB: volunteers with previous NAAT positive results are also allowed in groups 9, 10 and 11

Additional Exclusion criteria to Groups 4, 6, 9 and 10

- History of allergic disease or reactions likely to be exacerbated by Paracetamol
  - Note: Caution should be taken when recommending paracetamol to adults who already take paracetamol chronically

6.3.3 Re-vaccination exclusion criteria

The following AEs associated with any vaccine, or identified on or before the day of vaccination constitute absolute contraindications to further administration of an IMP to the volunteer in question. If any of these events occur during the study, the subject will not be eligible to receive a booster dose and will be followed up by the clinical team or their GP until resolution or stabilisation of the event:

- Anaphylactic reaction following administration of vaccine
- Pregnancy. An exception to this will be prior to receipt of a booster dose at extra visit B. If a pregnant woman has discussed vaccination with their usual clinician (e.g. GP) and chooses to receive a COVID-19 vaccination, this may be administered by the trial team as part of extra visit B (*Protocol 19.0:*) or as part of the provision of treatment to controls.
- Any AE that in the opinion of the Investigator may affect the safety of the participant or the interpretation of the study results

Participants who develop COVID-19 symptoms and have a positive NAAT test after the first vaccination can only receive a booster dose after a minimum 4 weeks interval from their first NAAT positive test, provided their symptoms have significantly improved. The decision to proceed with booster vaccinations in those cases will be at clinical discretion of the investigators. For participants who are asymptomatic and have a positive NAAT test, a minimum of 2 weeks from first NAAT positivity will be required before boosting.

6.3.4 Effective contraception for female volunteers

Female volunteers of childbearing potential are required to use an effective form of contraception during the course of the study.

Acceptable forms of contraception for female volunteers include:

- Established use of oral, injected or implanted hormonal methods of contraception.
- Placement of an intrauterine device (IUD) or intrauterine system (IUS).
- Total hysterectomy.
- Bilateral Tubal Occlusion
- Barrier methods of contraception (condom or occlusive cap with spermicide).
- Male sterilisation, if the vasectomised partner is the sole partner for the subject.
- True abstinence, when this is in line with the preferred and usual lifestyle of the subject (Periodic abstinence and withdrawal are not acceptable methods of contraception).

6.3.4.1 (Protocol V19.0) Contraception for female volunteers following unblinding and provision of ChAdOx1 nCoV-19 vaccines

In keeping with UK national guidance and the product approvals, female participants will be advised that they do not need to continue to use effective contraception methods if they are receiving ChAdOx1 nCoV-19 vaccinations as part of either:

- The national vaccine rollout
- Provision of a second dose to single dose participants within the trial
- Provision of treatment to controls within the trial (Protocol V19.0)

6.3.5 Withdrawal of Volunteers

In accordance with the principles of the current revision of the Declaration of Helsinki and any other applicable regulations, a volunteer has the right to withdraw from the study at any time and for any reason, and is not obliged to give his or her reasons for doing so. The Investigator may withdraw the volunteer at any time in the interests of the volunteer's health and well-being. In addition, the volunteer may withdraw/be withdrawn for any of the following reasons:

- Administrative decision by the Investigator.

- Ineligibility (either arising during the study or retrospectively, having been overlooked at screening).
- Significant protocol deviation.
- Volunteer non-compliance with study requirements.
- An AE, which requires discontinuation of the study involvement or results in inability to continue to comply with study procedures.
- Enrolment into a separate COVID-19 vaccine booster trial (section 6.3.6)

The reason for withdrawal will be recorded in the CRF. If withdrawal is due to an AE, appropriate follow-up visits or medical care will be arranged, with the agreement of the volunteer, until the AE has resolved, stabilised or a non-trial related causality has been assigned. Any volunteer who is withdrawn from the study may be replaced, if that is possible within the specified time frame. The DSMB or DSMB chair may recommend withdrawal of volunteers.

Vaccination with an approved or licensed COVID-19 vaccine according to government policy will not be considered a withdrawal from the study. Participants who are offered and accept a COVID-19 vaccine will be unblinded to the vaccines they have received prior to receiving the approved COVID-19 vaccine and will be asked to continue in the study.

If a volunteer withdraws from the study, data and blood samples collected before their withdrawal will still be used on the analysis. Storage of blood samples will continue unless the participant specifically requests otherwise.

In all cases of subject withdrawal, long-term safety data collection, including some procedures such as safety bloods, will continue as appropriate.

6.3.5.1 Discontinuation from Study Procedures

If a participant wishes to withdraw from the study because of the intensity of study visits or procedures and the commitment this requires, but are willing to remain in the study, they will be offered the option to discontinue some study procedures (e.g. booster dose in 2-dose groups, blood samples, vaccine diary, symptomatic pathway, e-diary, in-person symptomatic follow-up). This allows continued data collection on core study data for safety, immunogenicity and efficacy outcomes.

6.3.6 Recruitment of COV002 participants into separate COVID-19 booster vaccine trials (protocol V20.0)

6.3.6.1 Targeted Recruitment of COV002 participants for separate COVID-19 booster vaccine trials

COV002 participants received ChAdOx1 nCoV-19 vaccines earlier than individuals vaccinated as part of the national rollout of vaccines. They are therefore amongst the only individuals available to answer urgent public health questions around the need for additional booster vaccines prior to anticipated future waves of COVID-19. Where appropriate, participants enrolled in COV002 may therefore be targeted for recruitment into a separate COVID-19 booster vaccine trial.

6.3.6.2 Withdrawal of COV002 participants enrolled into other trials

If participants choose to enroll into separate ChAdOx1 COVID-19 vaccine booster trials they will be withdrawn from further follow up within COV002. Withdrawal from COV002 will occur once enrolment into the new trial has been confirmed. As safety and immunology follow up will continue under the new trial protocol, these participants will not continue with any further COV002 study procedures (including follow up visits). It is anticipated that less than 20% of individuals will be withdrawn for this purpose and the effects on long term follow up endpoints will therefore be minor.

6.4 COV009 follow on safety and immunogenicity study

Following completion of COV002, participants that have not been withdrawn and that have consented to be approached for future research will be approached for recruitment into COV009, a safety and immunogenicity follow on study under a separate protocol.

6.5 Pregnancy

Should a volunteer become pregnant during the trial, no further study IMP will be administered* (see below for exception). She will be followed up for clinical safety assessment with her ongoing consent and in addition will be followed until pregnancy outcome is determined. We would not routinely perform venepuncture in a pregnant volunteer unless there is clinical need. Given that no routine bloods will be drawn from pregnant volunteers, follow-up visits may be conducted over the phone/video consultation in order to minimise contact and exposure from SARS-CoV-2 in pregnant volunteers

* The current UK national vaccination policy advice is that pregnant women may receive the vaccine as part of the national rollout of COVID-19 vaccines in certain circumstances[20]. The decision on

whether COVID-19 vaccination should be recommended for any pregnant participants in the trial will not be taken by trial teams. However, if after discussion of the risk and benefits of COVID-19 vaccination with their usual clinician (e.g. GP), a pregnant participant chooses to have a COVID-19 vaccination during pregnancy, this may be administered by the trial team as part of extra visit B, although no blood samples will be drawn.

7 CLINICAL PROCEDURES

This section describes the clinical procedures for evaluating study participants and follow-up after administration of study vaccine.

7.1 Schedule of Attendance

All volunteers will have clinic attendances and procedures as indicated in the schedule of attendances below (tables 5-12). Subjects will receive either the ChAdOx1 nCoV-19 vaccine or MenACWY, and undergo follow-up for a total of 12 months from the last vaccination visit. Additional visits or procedures may be performed at the discretion of the investigators, e.g., further medical history and physical examination, or additional blood tests and other investigations if clinically relevant.

7.2 Observations, medical history and physical examination

Temperature will be routinely measured at the time-points indicated in the schedule of procedures. Respiratory rate, oxygen saturation, pulse, blood pressure and temperature will be measured at the COVID-19 testing visits and if clinically required. All subjects will undergo medical history and a targeted physical examination if considered necessary at screening or pre-enrolment on D0. The purpose of this examination is to assess and document the subject's baseline health status so that any later change can be determined. Vital signs (temperature, heart rate, respiratory rate, blood pressure +/- oxygen saturation) will be measured at screening or pre-enrolment on D0 as part of baseline assessments. Further medical history, physical examination and observations may be done throughout the study based on clinical discretion. A targeted physical examination, including neurological assessment, must be conducted, when appropriate, in the event of a SAE.

Blood tests, Nose/Throat Swabs, Saliva samples and urinalysis

Blood will be drawn for the following laboratory tests and processed at contractually agreed NHS Trust laboratories using NHS standard procedures:

- **Haematology;** Full Blood Count
- **Biochemistry;** Sodium, Potassium, Urea, Creatinine, Albumin, Liver Function Tests (ALT, ALP, Bilirubin)
- **Diagnostic serology;** HBsAg, HCV antibodies, HIV antibodies in groups 1, 2, 5a, 5b, 5c, 5d, and 5e, 5f, 7 and 8 only (specific consent will be gained prior to testing blood for these blood-borne viruses). HBsAg and HCV antibodies will be in group 12 with HIV antibodies only done at the investigators discretion.
- **Immunology;** Human Leukocyte Antigen (HLA) typing (groups 5a, 5b, 5c only)
- **COVID-19;** A nose/throat swab and/or saliva sample will be taken for COVID-19 NAAT testing.
- **CD4 count and HIV viral load;** volunteers in group 12 only, before enrolment.

Additional safety blood tests may be performed if clinically relevant at the discretion of the medically qualified investigators, including potential prognostic indicators or markers of severe COVID-19 disease

At University of Oxford research laboratories or at designated specialist laboratories:

- **Immunology**; Immunogenicity will be assessed by a variety of immunological assays. This may include antibodies to SARS-CoV-Spike and non-Spike antigens by ELISA, ex vivo ELISpot assays for interferon gamma and flow cytometry assays, neutralising and other functional antibody assays and B cell analyses, virus neutralising Ab (NAb) assays against live and/or pseudotype SARS-CoV-2 virus. Other exploratory immunological assays including cytokine analysis and other antibody assays, DNA analysis of genetic polymorphisms potentially relevant to vaccine immunogenicity and gene expression studies amongst others may be performed at the discretion of the Investigators. Further exploratory immunology assays may be performed at the discretion of the Investigators on HIV and non-HIV cohorts, including, but not limited to: T cell Proliferative responses to SARS-CoV-2 antigen; T cell cross-reactivity to circulating common cold coronaviruses; Multiparameter immunophenotyping by CyTOF; BCR and TCR repertoire analysis; Serum analysis by Luminex (including inflammatory, anti-inflammatory and adaptive cytokines, chemokines, growth factors and antimicrobial proteins); HIV viral reservoir; amongst others.

- **Stool samples**; SARS-CoV-2 NAAT, infectivity assays, calprotectin, and other exploratory immunology and microbiology assays may be conducted in a subset of participants, subject to site capacity, sample and test availability
- **Mucosal Immunity Swabs (Synthetic Absorptive Matrix [SAM])**; an assessment of mucosal immunity will be conducted in a subset of participants, subject to site capacity, sample and test availability.
- **SARS-CoV-2 weekly PCR sample**; weekly nose/throat swabs will be processed via the Department of Health and Social Care's community testing programme.

At each site:

- **Urinalysis;** For female volunteers of child bearing potential only, urine will be tested for beta-human chorionic gonadotrophin (β-HCG) at screening (when applicable) and immediately prior to vaccination. Where local policies require, a serum β-HCG may replace urinary test.
- **Serum;** Samples may be centrifuged at local sites and shipped to University of Oxford laboratories or elsewhere for analysis.

SARS-CoV-2 serology will be conducted at screening (except in groups 5d, 5e, 5f, 9, 10 and 11). These may be conducted at appropriate university research or NHS trust laboratories facilities at sites. SARS-CoV-2 screening serology samples and or COVID-19 related immunology samples taken, may also be shipped from sites to a central laboratory facility at the University of Oxford or elsewhere.

Collaboration with other specialist laboratories in the UK, Europe and outside of Europe for further exploratory tests may occur. This would involve the transfer of serum, urine, stool or plasma, PBMC and/or other study samples to these laboratories, but these would remain anonymised. Informed consent for this will be gained from volunteers. Samples collected for the purposes of COVID-19 diagnosis might be sent to reference labs in the UK alongside their personal data. This would be in line with the national guidance and policy for submitting samples for testing at reference labs.

Immunological assays will be conducted according to local SOPs.

Subjects will be informed that there may be leftover samples of their blood (after all testing for this study is completed), and that such samples may be stored indefinitely for possible future research (exploratory immunology), including genotypic testing of genetic polymorphisms potentially relevant to vaccine immunogenicity. Subjects will be able to decide if they will permit such future use of any

leftover samples. With the volunteers' informed consent, any leftover cells, urine, stool and serum/plasma will be frozen indefinitely for future analysis of COVID-19 and other coronaviruses related diseases or vaccine-related responses. If a subject elects not to permit this, all of that subject's leftover samples will be discarded after the required period of storage to meet Good Clinical Practice (GCP) and regulatory requirements.

Samples that are to be stored for future research will be transferred to the OVC Biobank (REC 16/SC/0141).

7.3 Study visits

The study visits and procedures will be undertaken by one of the clinical trials team. The procedures to be included in each visit are documented in the schedule of attendances (tables 5-12). Each visit is assigned a time-point and a window period, within which the visit will be conducted. In order to obtain core study data if a participant is unable to attend a study visit, the visit will be conducted by phone or video consultation and information recorded in the CRF.

7.3.1 Screening visit

Participants will be required to complete an online questionnaire as an initial confirmation of eligibility.

In order to minimise the risks of COVID-19 exposure in clinic, participants may be asked to provide verbal permission or electronic consent to collect and record details of their medical history over the phone, ahead of their screening visit (for the purpose of the eligibility assessment and if enrolled the recording of baseline health records). This will be recorded on their Pre-screening questionnaire (either directly completed by the volunteer or on behalf of the volunteer by a member of the study team with the volunteers verbal consent) or on the reply slip. This will reduce the amount of time participants have with the clinical team during their screening procedures.

All potential volunteers will have a screening visit, which may take place up to 90 days prior to vaccination. At the screening visit, a video presentation of the aims of the study and all tests to be carried out may be screened to an audience or accessed remotely. Individually each volunteer will have the opportunity to question an appropriately trained and delegated researcher before signing the consent. Informed consent will be taken before screening/enrolment, as described in section 6.2.

If written consent is obtained, the procedures indicated in the schedule of attendances will be undertaken including a medical history (if not already collected by phone), physical examination (if required), height and weight and blood tests including a SARS-CoV-2 screening test (all groups except 4c, 6b, 5d, 5e, 5f, 9, 10 and 11) and safety bloods (groups 1, 2, 5, 7 and 8) will be done. To avoid unnecessary additional venepuncture, if the appropriate blood test results for screening are available for the same volunteer from a previous screening visit or from another study, these results may be used for assessing eligibility (provided the results date is within the 6 months preceding enrolment in COV002).

We will aim to contact the subject's general practitioner with the permission of the subject after screening to corroborate medical history when possible and practical to do so (Groups 1, 2, 7 and 8 only, except group 12 where GPs can be replaced by their HIV consultant). GPs will be notified that the subject has volunteered for the study (all study groups,).

Abnormal clinical findings from blood tests at screening (Groups 1, 2, 5, 7 and 8 only) will be assessed by a medically qualified study member. Abnormal blood tests following screening will be assessed according to site-specific laboratory adverse event grading tables. Any abnormal test result deemed clinically significant may be repeated to ensure it is not a single occurrence. If an abnormal finding is deemed to be clinically significant, the volunteer will be informed and appropriate medical care arranged with the permission of the volunteer.

The eligibility of the volunteer will be reviewed at the end of the screening visit and again when all results from the screening visit have been considered. Decisions to exclude the volunteer from enrolling in the trial or to withdraw a volunteer from the trial will be at the discretion of the Investigator. If eligible, a day 0 visit will be scheduled for the volunteer to receive the vaccine and subsequent follow-up.

7.3.2 Day 0: Enrolment and vaccination visit

Participants in all groups will have informed consent taken as per section 6.2. Volunteers will be considered enrolled in to the trial at the point of vaccination. Before vaccination/trial intervention, the eligibility of the volunteer will be reviewed. Temperature will be observed and if necessary, a medical history and physical examination maybe undertaken to determine need to postpone vaccination or withdraw the participant. Vaccinations/trial intervention will be administered as described below.

7.3.2.1 Vaccination

All vaccines will be administered intramuscularly according to specific SOPs. The injection site will be covered with a sterile dressing and the volunteer will stay in the trial site for observation for a minimum of 15 minutes (+15 minutes), in case of immediate adverse events. The sterile dressing will be removed and injection site inspected.

In groups 1-3, 5, 7, 8, 11 and 12 and in a subset of volunteers in groups 4, 6, 9 and 10 (n=up to 1000, in each groups 4 and 6 and approximately 500 in each of groups 9 and 10), participants will be given an oral thermometer, tape measure and diary card (paper or electronic), with instructions on use. The approximate 3000 participants in groups 4, 6, 9 and 10 that are required to complete diaries will be allocated according to site. The allocation will ensure distribution of ages. All participants will be given the emergency 24 hour telephone number to contact the on-call study physician if needed. Volunteers will be instructed on how to self-assess the severity of these AEs. There will also be space on the diary card to self-document unsolicited AEs, and whether medication was taken to relieve the symptoms. Participants in groups 4, 6, 9 and 10 will be advised to take prophylactic paracetamol for 24 hours after vaccination and will record this in the e-diary (up to 1,000 participants in each of groups 4 and 6, and approximately 500 in each of groups 9 and 10 only). Participants in groups 1-3 and 5, 7, 8 and 11 will be asked to report on solicited AEs for 7 days and unsolicited AEs for 28 days. The subset of approximately 3000 participants in groups 4, 6, 9, and 10 will be asked to report solicited and unsolicited AEs for 7 days only. Participants in group 12 will be asked to report on solicited local and systemic AEs for 7 days and unsolicited AEs for 28 days.

Diary cards will collect information on the timing and severity of the following solicited AEs:

Table 3**. Solicited AEs as collected on post vaccination diary cards**

**Local solicited AEs**	**Systemic solicited AEs**
Pain	Fever
Tenderness	Feverishness
Redness	Chills
Warmth	Joint pains
Itch	Muscle pains
Swelling	Fatigue
Induration	Headache
	Malaise
	Nausea
	Vomiting

7.3.2.2 <u>Sequence of Enrolment and Vaccination of Volunteers</u>

Prior to initiation of the study, any newly available safety data will be reviewed from animal studies or clinical trials of coronavirus vaccines being tested in the UK (COV001) or elsewhere, and discussed with the DSMB and/or MHRA as necessary. Recruitment of groups 1, 4, 5, 6, 11 and 12 may occur simultaneously. However, older adults aged 56 and above will only be recruited into groups 4, 6, 7 and 8 following a safety review of participants enrolled in groups 1 and 2. This review will include the profile of AEs observed following a single dose of ChAdOx1 nCoV-19. Adults aged 56 and above will only be recruited into group 9 following safety review of groups 1 and 7, and into group 10 following safety review of groups 2 and 8.

7.3.3 Subsequent visits

Follow-up visits will take place as per the schedule of attendances described in tables 5-12 with their respective windows. Volunteers in groups 1-3 and 5, 7 and 8 will be assessed for local and systemic adverse events, interim history, physical examination, review of diary cards (paper or electronic) and blood tests at these time points as detailed in the schedule of attendances. Blood will also be taken for immunology purposes.

If volunteers experience adverse events (laboratory or clinical), which the investigator (physician), CI and/or DSMB chair determine necessary for further close observation, the volunteer may be admitted to an NHS hospital for observation and further medical management under the care of the Consultant on call.

Table 4 Schedule of attendances for participants in groups 1a, 2a, 7a and 8a (single dose). *Protocol V19:* Unblinding / provision of treatment procedures will occur concurrently (Table 14) to these procedures.

Attendance Number	1[S]	2	3	4	5	6	7	8	9	COVID-19 Testing	COVD-19 NAAT positive + 7 days (remote)	COVID-19 Follow-up	Extra Visit A	Extra Visit B
													Optional	Optional
Timeline**(days)	≤ 90	0	3	7	14	28	56	182	364	As required	7 days post NAAT positive		At time of early unblinding before receipt of deployed vaccine	At earliest possibility
Time window (days)			±1	±2	±3	±7	±7	±14	±30	N/A	-2,+7		N/A	≥4 weeks post prime
Verbal Consent to discuss medical history over the phone	(X)													
Informed Consent	X													
Review contraindications, inclusion and exclusion criteria	X	X												
Vaccination		X												X
Vital signs^	X	X	X	X	X	X	X	X	X	X				
Telephone/Video call											X	As required		
Ascertainment of adverse events		X	X	X	X	X	X	X	X	X	X	X	X	X
Diary cards provided		X												

Attendance Number	1[S]	2	3	4	5	6	7	8	9	COVID-19 Testing	COVD-19 NAAT positive + 7 days (remote)	COVID-19 Follow-up	Extra Visit A	Extra Visit B
													Optional	Optional
Diary cards collected						X								
Weekly household exposure questionnaire	ongoing													
Medical History, Physical Examination	X	(X)	(X)	(X)	(X)	(X)	(X)	(X)	(X)	(X)	(X)			
Biochemistry, Haematology (mL)	5	(5)*	5	5		5								
SARS-CoV-2 Serology	5													
Exploratory immunology (mL)		up to 55		up to 50	up to 50	up to 50	up to 50	up to 50	up to 50	up to 50			(up to 50)	(Up to 50)
PAXgenes		2.5								2.5				
Nose/Throat Swab and/or Saliva Sample										X			X	
Stool sample[a,b]											(X)	(X)		
Weekly PCR sample[a]	ongoing													
Urinary bHCG (women of childbearing potential only)	X	X												X
HBsAg, HCV Ab, HIV serology (mL)	5													
Blood volume per visit	Up to 15	Up to 57.5	Up to 5	Up to 55	Up to 50	Up to 55	Up to 50	Up to 50	Up to 50	up to 52.5			Up to 50	Up to 50
Cumulative blood volume[%]	15	72.5	77.5	132.5	182.5	237.5	287.5	337.5	387.5	440	440		490	490

S = screening visit; (X) = if considered necessary; ** Timeline is approximate only. Exact timings of visits relate to the day on enrolment, ie, each visit must occur at indicated number of days after enrolment ± time window. ^Vital signs at screening or pre-enrolment assessment on D0 include pulse, blood pressure, temperature, respiratory rate +/- oxygen saturation. Only temperature will be routinely measured at subsequent follow-up visits. At COVID-19 testing visits a full set of observations will be taken, including respiratory rate and oxygen saturation. % Cumulative blood volume for volunteers if blood taken as per schedule, and excluding any repeat safety blood test that may be necessary. Blood volumes may vary according to local site equipment and practices. *Safety bloods should only be repeated at vaccination day if there is a period greater than 2 weeks between screening and vaccination visit; an extra 5mL of blood should be added to the overall cumulative blood volume. [a] Subject to site capacity, sample and test availability. [b] Optional Stool sample at approximately 7 days after onset of symptoms for those who have a positive SARS-CoV-2 NAAT test result and possibly again at 14 days after symptom onset if necessary.

Table 5 Schedule of attendances for participants in groups 1b, 2b, 5d, 7b and 8b (two dose). *Protocol V19:* Unblinding / provision of treatment procedures will occur concurrently (Table 14) to these procedures.

Attendance Number	1^S	2 (V1)	3	4	5	6 (V2)	7	8	9	10	11	12	COVID-19 Testing	COVID-19 NAAT positive +7 days (Remote)	COVID-19 Follow-up	Extra Visit A	Extra Visit B***
																Optional	Optional if not had boost
Timeline** (days)	≤ 90	0	3	7	14	28	31 (3 days post boost)	35 (7 days post boost)	42 (14 days post boost)	56 (28 days post boost	182	364	As required	7 days post NAAT positive		At time of early unblinding before receipt of deployed vaccine	At earliest possibility
Time window (days)			±1	±3	±3	+14	±1	±2	±3	±7	±14	±30	N/A	-2, +7		N/A	≥4 weeks post prime
Verbal Consent to discuss medical history over the phone	(X)																
Informed Consent	X																
Review contraindications, inclusion and exclusion criteria	X	X				X											
Vaccination		X				X											X
Vital signs^	X	X	X	X	X	X	X	X	X	X	X	X	X				
Telephone/Video call														X	As required		
Ascertainment of adverse events		X	X	X	X	X	X	X	X	X	X	X	X	X	X	X	X
Diary cards provided		X				X											
Diary cards collected						X				X							

Attendance Number	1[S]	2 (V1)	3	4	5	6 (V2)	7	8	9	10	11	12	COVID-19 Testing	COVD-19 NAAT positive +7 days (Remote)	COVID-19 Follow-up	Extra Visit A	Extra Visit B***
																Optional	Optional if not had boost
Weekly household exposure questionnaire			ongoing														
Medical History, Physical Examination	X[c]	(X)	(X)	(X)	(X)	(X)	(X)	(X)	(X)	(X)	(X)	(X)	(X)	(X)			
Biochemistry[$], Haematology (mL)	5	(5)*	5	5		5	5	5		5							
SARS-CoV-2 Serology (mL)	(5)																
Exploratory immunology[£] (mL)		up to 55		up to 50	up to 50	up to 50		up to 50	up to 50	up to 50	up to 50	up to 50	up to 50			(up to 50)	(up to 50)
PAXgenes		2.5											2.5				
Nasal/Throat Swab, and/or Saliva sample													X				
Stool sample [a,b]														(X)	(X)		
Weekly PCR sample							ongoing										
Urinary bHCG (women of childbearing potential only)	X	X				X											X
HBsAg, HCV Ab, HIV serology (mL)	5																
Blood volume per visit	Up to 15	Up to 57.5	Up to 5	Up to 55	Up to 50	Up to 55	Up to 5	Up to 55	Up to 50	Up to 55	Up to 50	Up to 50	up to 52.5			Up to 50	Up to 50
Cumulative blood volume[%]	15	72.5	77.5	132.5	182.5	237.5	242.5	297.5	347.5	402.5	452.5	502.5	555	555		605	605

S = screening visit; (X) = if considered necessary; ** Timeline is approximate only. Exact timings of visits relate to the day on enrolment, ie, each visit must occur at indicated number of days after enrolment ± time window. Where a second dose is administered, the window will apply to the time their

last vaccination took place ^Vital signs at screening or pre-enrolment assessment on D0 include pulse, blood pressure, temperature, respiratory rate +/- oxygen saturation. Only temperature will be routinely measured at subsequent follow-up visits. At COVID-19 testing visits a full set observations will be taken, including respiratory rate and oxygen saturation. % Cumulative blood volume for volunteers if blood taken as per schedule, and excluding any repeat safety blood test that may be necessary. Blood volumes may vary according to local site equipment and practices. *Safety bloods should only be repeated at vaccination day if there is a period greater than 2 weeks between screening and vaccination visit; an extra 5mL of blood should be added to the overall cumulative blood volume. [a] Subject to site capacity, sample and test availability. [b] Optional Stool sample at approximately 7 days after onset of symptoms for those who have a positive SARS-CoV-2 NAAT test result and possibly again at 14 days after symptom onset if necessary. [c] Targeted physical examination if considered necessary for group 5d. *** For participants who previously declined to have a second dose of vaccine but now wish to complete study vaccine following unblinding.

Table 6**:** Schedule of attendances for participants in groups 5e and 5f (two dose). *Protocol V19:* Unblinding / provision of treatment procedures will occur concurrently (Table 14) to these procedures.

Attendance Number	1[S]	2 (V1)	3	4	5	6 (V2)	7	8	9	10	11	12	COVID-19 Testing	COVD-19 NAAT positive +7 days (Remote)	COVID-19 Follow-up
Timeline** (days)	≤ 90	0	3	7	14	28	31 (3 days post boost)	35 (7 days post boost)	42 (14 days post boost)	56 (28 days post boost	182	364	As required	7 days post NAAT positive	
Time window (days)			±1	±3	±3	+14	±1	±2	±3	±7	±14	±30	N/A	-2,+7	
Verbal Consent to discuss medical history over the phone	(X)														
Informed Consent	X														
Review contraindications, inclusion and exclusion criteria	X	X				X									
Vaccination		X				X									
Vital signs^	X	X	X	X	X	X	X	X	X	X	X	X	X		
Telephone/Video call														X	As required
Ascertainment of adverse events		X	X	X	X	X	X	X	X	X	X	X	X	X	X
Diary cards provided		X				X									
Diary cards collected						X				X					
Weekly household exposure questionnaire			ongoing												
Medical History, Physical Examination	X[c]	(X)	(X)	(X)	(X)	(X)	(X)	(X)	(X)	(X)	(X)	(X)	(X)	(X)	
Biochemistry[$], Haematology (mL)	5[d]	(5)*	5	5		5	5	5		5					
Exploratory immunology[£] (mL)		up to 60		up to 60	up to 60	up to 60		up to 60	up to 60	up to 60	up to 50	up to 50	up to 50		

Attendance Number	1[S]	2 (V1)	3	4	5	6 (V2)	7	8	9	10	11	12	COVID-19 Testing	COVD-19 NAAT positive +7 days (Remote)	COVID-19 Follow-up
PAXgenes		2.5											2.5		
Nasal/Throat Swab, and/or Saliva sample													X		
Stool sample [a,b]														(X)	(X)
Weekly PCR sample			ongoing												
Urinary bHCG (women of childbearing potential only)	X	X				X									
HBsAg, HCV Ab, HIV serology (mL)	5[d]														
Blood volume per visit	Up to 15	Up to 67.5	Up to 5	Up to 65	Up to 60	Up to 65	Up to 5	Up to 65	Up to 60	Up to 65	Up to 50	Up to 50	up to 52.5		
Cumulative blood volume[%]	15	82.5	87.5	152.5	212.5	277.5	282.5	347.5	407.5	472.5	522.5	572.5	625	625	

S = screening visit; (X) = if considered necessary; ** Timeline is approximate only. Exact timings of visits relate to the day on enrolment, ie, each visit must occur at indicated number of days after enrolment ± time window. Where a second dose is administered, the window will apply to the time their last vaccination took place ^Vital signs at screening or pre-enrolment assessment on D0 include pulse, blood pressure, temperature, respiratory rate +/- oxygen saturation. Only temperature will be routinely measured at subsequent follow-up visits. At COVID-19 testing visits a full set observations will be taken, including respiratory rate and oxygen saturation. % Cumulative blood volume for volunteers if blood taken as per schedule, and excluding any repeat safety blood test that may be necessary. Blood volumes may vary according to local site equipment and practices. *Safety bloods should only be repeated at vaccination day if there is a period greater than 2 weeks between screening and vaccination visit; an extra 5mL of blood should be added to the overall cumulative blood volume. [a] Subject to site capacity, sample and test availability. [b] Optional Stool sample at approximately 7 days after onset of symptoms for those who have a positive SARS-CoV-2 NAAT test result and possibly again at 14 days after symptom onset if necessary. [c] Targeted physical examination if considered necessary for groups 5e and 5f.[d] If volunteer has had bloods taken in previous 6 months (for COV002 or another study) these do not need repeating at screening, but safety bloods will be taken prior to vaccination.

Table 7 Schedule of attendances for participants in group 4a and 6a. *Protocol V19:* Unblinding / provision of treatment procedures will occur concurrently (Table 14) to these procedures.

Attendance Number	1	2	3	4	5	6	COVID-19 Testing	COVD-19 NAAT positive +7 days (remote)	COVID-19 Follow-up	Extra Visit A	Extra Visit B
										Optional	Optional
Timeline** (days)		0	28	90	182	364	As required	7 days post NAAT positive		At time of early unblinding before receipt of deployed vaccine	At earliest possibility
Time window (days)			-7/+14	±14	±14	±30	N/A	-2,+7		N/A	≥4 weeks post prime
Informed Consent	X	X									
Review contraindications, inclusion and exclusion criteria	X	X									
Vaccination		X									X
Vital signs^	X	X	(X)	(X)	(X)	(X)	X				
Telephone/Video call								X	As required		
Ascertainment of adverse events		X	X	X	X	X	X	X	X	X	X
Diary Cards$		X									
Weekly household exposure questionnaire			ongoing								
Medical History (required at 1 timepoint prior to enrolment),	X	X	(X)	(X)	(X)	(X)	(X)	(X)			
Physical Examination (if necessary)	(X)	(X)	(X)	(X)	(X)	(X)	(X)	(X)			
Biochemistry, Haematology (mL)											

Attendance Number	1	2	3	4	5	6	COVID-19 Testing	COVD-19 NAAT positive +7 days (remote)	COVID-19 Follow-up	Extra Visit A	Extra Visit B
										Optional	Optional
Exploratory immunology (mL)		up to 50	up to 50	up to 50	up to 50	up to 50	up to 50[d]			(up to 50)	(up to 50)
Mucosal Immunity[a,c]		(X)	(X)								
PAXgenes						(2.5)[P]	2.5				
SARS-CoV-2 Serology	5										
Nose/Throat Swab and/or saliva sample							X				
Stool sample [a,b]								(X)	(X)		
Weekly PCR sample[a]			ongoing								
Urinary bHCG (women of childbearing potential only)	X	X									X
Blood volume per visit	5	50	50	50	50	50	up to 55.5			up to 50	up to 50
Cumulative blood volume[%]	5	55	105	155	205	255	307.5	307.5		357.5	357.5

S = screening visit; (X) = if considered necessary; ** Timeline is approximate only. Exact timings of visits relate to the day on enrolment, ie, each visit must occur at indicated number of days after enrolment ± time window. ^Vital signs at screening or pre-enrolment assessment on D0 include pulse, blood pressure, temperature, respiratory rate +/- oxygen saturation. Only temperature will be routinely measured at subsequent follow-up visits. At COVID-19 testing visits a full set observations will be taken, including respiratory rate and oxygen saturation. % Cumulative blood volume for volunteers if blood taken as per schedule, and excluding any repeat safety blood test that may be necessary. Blood volumes may vary according to local site equipment and practices. [a] Subject to site capacity, sample and test availability. [d] Optional and subject to site capacity. [$] A subset of up to 1000 volunteers will be asked to fill an e-diary with reactogenicity symptoms for 7 days only in groups 4, 6, 9 and 10. [P] Pax genes sample at D364 to be done only on that have attended at least one COVID-19 testing visit within the trial, an extra 2.5mL should be added to the cumulative blood volume when this applies. [b] Optional Stool sample at approximately 7 days after onset of symptoms for those who have a positive SARS-CoV-2 NAAT test result and possibly again at 14 days after symptom onset if necessary. [c] Mucosal immunity assessments to be done in a subset of group 6 individuals only.

Table 8**:** Schedule of attendances for participants in group 4b. Protocol V19: Unblinding / provision of treatment procedures will occur concurrently (Table 14) to these procedures.

Attendance Number	1	2	3	4	5	6	7	8	COVID-19 Testing	COVD-19 NAAT positive +7 days (Remote)	COVID-19 Follow-up	Extra Visit A	
												Optional	
Timeline** (days)		0	28	42 (14 days post boost)	56 (28 days post boost)	118 (90 days post boost)	210 (182 days post boost)	392 (364 days post boost)	As required	7 days post NAAT positive		At time of early unblinding before receipt of deployed vaccine	
Time window (days)			+14	±7	±7	±14	±14	±30	N/A	-2,+7		N/A	
Informed Consent	X	X											
Review contraindications, inclusion and exclusion criteria	X	X											
Vaccination		X	X										
Vital signs^	X	X	(X)	(X)	(X)	(X)	(X)	(X)	X				
Telephone/Video call										X	As required		
Ascertainment of adverse events		X	X	X	X	X	X	X	X	X	X	X	
Diary Cards[$]		X	X										
Weekly household exposure questionnaire				ongoing									
Medical History (required at 1 timepoint prior to enrolment),	X	X	(X)	(X)		(X)	(X)	(X)	(X)	(X)			

Attendance Number	1	2	3	4	5	6	7	8	COVID-19 Testing	COVD-19 NAAT positive +7 days (Remote)	COVID-19 Follow-up	Extra Visit A	
												Optional	
Physical Examination (if necessary)	(X)	(X)	(X)	(X)		(X)	(X)	(X)	(X)	(X)			
Biochemistry, Haematology (mL)													
Exploratory immunology (mL)		up to 50	up to 50	up to 50	up to 50	up to 50	up to 50	up to 50	up to 50[d]			up to 50	
PAXgenes								(2.5)[P]	2.5				
SARS-CoV-2 Serology	5												
Nose/Throat Swab and/or saliva sample									X				
Stool sample [a,b]										(X)	(X)		
Weekly PCR sample[a]			ongoing										
Urinary bHCG (women of childbearing potential only)	X	X											
Blood volume per visit	5	50	50	50	50	50	50	50	up to 52.5			up to 50	
Cumulative blood volume[%]	5	55	105	155	205	255	305	355	407.5	407.5		457.5	

S = screening visit; (X) = if considered necessary; ** Timeline is approximate only. Exact timings of visits relate to the day. Where a second dose is administered, the window will apply to the time their last vaccination took place . ^Vital signs at screening or pre-enrolment assessment on D0 include pulse, blood pressure, temperature, respiratory rate +/- oxygen saturation. Only temperature will be routinely measured at subsequent follow-up visits. At COVID-19 testing visits a full set observations will be taken, including respiratory rate and oxygen saturation. % Cumulative blood volume for volunteers if blood taken as per schedule, and excluding any repeat safety blood test that may be necessary. Blood volumes may vary according to local site equipment and practices. [a] Subject to site capacity, sample and test availability. [d] Optional and subject to site capacity. [$] A subset of up to 1000 volunteers will be asked to fill an e-diary with reactogenicity symptoms for 7 days only in groups 4, 6, 9 and 10. [P] Pax genes sample at D364 to be done only on participants that have attended at least one COVID-19 testing visits within the trial testing visits, an extra 2.5mL should be added to the cumulative blood

volume when this applies. [b] Optional Stool sample at approximately 7 days after onset of symptoms for those who have a positive SARS-CoV-2 NAAT test result and possibly again at 14 days after symptom onset if necessary.

Table 9 Schedule of attendances for participants in group 5a1,5a2, 5b and 5c. Protocol V19: Unblinding / provision of treatment procedures will occur concurrently (Table 14) to these procedures.

Attendance Number	1[S]	2	3	4	5	6	7	8	9	COVID-19 Testing	COVD-19 NAAT positive + 7 days	COVID-19 Follow-up	Extra Visit A	Extra Visit B
													Optional	Optional
Timeline**(days)	≤ 90	0	3	7	14	28	56	182	364	As required	7 days post NAAT positive		At time of early unblinding before receipt of deployed vaccine	At earliest possibility
Time window (days)			±1	±2	±3	±7	±7	±14	±30	N/A	-2,+7		N/A	≥4 weeks post prime
Informed Consent	X													
Review contraindications, inclusion and exclusion criteria	X	X												
Vaccination		X												X
Vital signs	X	X	(X)	(X)	(X)	(X)	(X)	(X)	(X)	X				
Telephone/Video call											X	As required		
Ascertainment of adverse events		X	X	X	X	X	X	X	X	X	X	X	X	X
Diary cards provided		X												
Diary cards collected						X								
Weekly household exposure questionnaire			ongoing											

Attendance Number	1[S]	2	3	4	5	6	7	8	9	COVID-19 Testing	COVD-19 NAAT positive + 7 days	COVID-19 Follow-up	Extra Visit A	Extra Visit B
													Optional	Optional
Medical History, Physical Examination	(X)	(X)	(X)	(X)	(X)	(X)	(X)	(X)	(X)	(X)	(X)			
Biochemistry, Haematology (mL)	5	(5)*	5	5		5								
Hep B, C and HIV serology	5													
Exploratory immunology (mL)[e]		up to 55		up to 50	up to 50	up to 50	up to 50	up to 50	up to 50	up to 50			up to 50	up to 50
SARS-CoV-2 Serology	5													
PAXgenes		2.5								2.5				
Nose/Throat Swab and/or Saliva Sample										X				
Stool sample [a,b]											(X)	(X)		
Weekly PCR sample			ongoing											
Urinary bHCG (women of childbearing potential only)	X	X												X
HLA typing (mL)		4												
Blood volume per visit	15	61.5	5	55	50	55	50	50	50	up to 52.5			up to 50	up to 50
Cumulative blood volume[%]	15	76.5	81.5	136.5	186.5	241.5	291.5	341.5	391.5	444	444		494	494

S = screening visit; (X) = if considered necessary; ** Timeline is approximate only. Exact timings of visits relate to the day on enrolment, ie, each visit must occur at indicated number of days after enrolment ± time window. ^Vital signs at screening or pre-enrolment assessment on D0 include pulse, blood pressure, temperature, respiratory rate +/- oxygen saturation. Only temperature will be routinely measured at subsequent follow-up visits. At COVID-19 testing visits a full set observations will be taken, including respiratory rate and oxygen saturation. % Cumulative blood volume for volunteers if blood taken as per schedule, and excluding any repeat safety blood test that may be necessary. Blood volumes may vary according to local site equipment and practices. *Safety bloods should only be repeated at vaccination day if there is a period greater than 2 weeks between screening and vaccination visit;

an extra 5mL of blood should be added to the overall cumulative blood volume. [a] Subject to site capacity, sample and test availability. [b] Optional Stool sample at approximately 7 days after onset of symptoms for those who have a positive SARS-CoV-2 NAAT test result and possibly again at 14 days after symptom onset if necessary. [e] Participants enrolled in group 5b will only have B-cell immunology assessments whereas those in group 5c will have both B and T-cell immunology assessments.

Table 10 Schedule of attendances for participants in group 4c, 1a3, 1a4, 2a3, 2a4, 5a3, 5a4 and 6b (booster) *Protocol V19:* Unblinding / provision of treatment procedures will occur concurrently (Table 14) to these procedures.

Attendance Number (boost)	1 V2 (booster)	2	3	4	5	COVID-19 Testing	COVD-19 NAAT positive + 7 days	COVID-19 Follow-up	Extra Visit
Timeline** (days)	> 4 weeks post prime	28 post boost	90 post boost	182 post boost	364 post boost	As required	7 days post NAAT positive		At time of early unblinding before receipt of deployed vaccine
Time window (days)	+14	±7	±14	±14	±30	N/A	-2, +7		N/A
Informed Consent	X								
Review contraindications, inclusion and exclusion criteria	X								
Vaccination	X								
Vital signs^	(X)	(X)	(X)	(X)	(X)	X			
Telephone/Video call							X	As required	
Ascertainment of adverse events	X	X	X	X	X	X	X	X	X
Diary Cards[$]	X								

Attendance Number (boost)	1 V2 (booster)	2	3	4	5	COVID-19 Testing	COVD-19 NAAT positive + 7 days	COVID-19 Follow-up	Extra Visit
Weekly household exposure questionnaire		ongoing							
Medical History	(X)	(X)	(X)	(X)	(X)	(X)	(X)		
Physical Examination (if necessary)	(X)	(X)	(X)	(X)	(X)	(X)	(X)		
Biochemistry, Haematology (mL)									
Exploratory immunology (mL)	up to 50	up to 50	up to 50	up to 50	up to 50	up to 50[d]			(up to 50)
PAXgenes					(2.5)[P]	2.5			
Nose/Throat Swab and/or saliva sample						X			
Stool sample [a,b]							(X)	(X)	
Weekly PCR sample[a]		ongoing							
Urinary bHCG (women of childbearing potential only)	X								
Blood volume per visit	50	50	50	50	50	up to 52.5			up to 50
Cumulative blood volume[%]	50	100	150	200	250	302.5	302.5		352.5

S = screening visit; (X) = if considered necessary; ** Timeline is approximate only. Exact timings of visits relate to the day on enrolment, ie, each visit must occur at indicated number of days after enrolment ± time window. Where a second dose is administered, the window will apply to the time their last vaccination took place . ^Only temperature will be routinely measured at subsequent follow-up visits. At COVID-19 testing visits a full set observations will be taken, including respiratory rate and oxygen saturation. % Cumulative blood volume for volunteers if blood taken as per schedule,

and excluding any repeat safety blood test that may be necessary. Blood volumes may vary according to local site equipment and practices. [a] Subject to site capacity, sample and test availability. [d] Optional and subject to site capacity. [$] A subset of up to 1000 volunteers will be asked to fill an e-diary with reactogenicity symptoms for 7 days only in groups 4, 6, 9 and 10. [P] Pax genes sample at D364 to be done only on participants that have attended at least one COVID-19 testing visits within the trial, an extra 2.5mL should be added to the cumulative blood volume when this applies. [b] Optional Stool sample at approximately 7 days after onset of symptoms for those who have a positive SARS-CoV-2 NAAT test result and possibly again at 14 days after symptom onset if necessary.

Table 11. Schedule of attendances for participants in groups 9 and 10. Protocol V19: Unblinding / provision of treatment procedures will occur concurrently (Table 14) to these procedures.

Attendance Number	1	2 V1	3 V2	4	5	6	7	COVID-19 Testing	COVD-19 NAAT positive+ 7 days (remote)	COVID-19 Follow-up	Extra Visit A	Extra Visit B***
											Optional	Optional if not had boost
Timeline** (days)		0	28	56 (28 post boost)	118 (90 post boost	210 (182 post boost)	392 (364 post boost)	As required	7 days post NAAT positive		At time of early unblinding before receipt of deployed vaccine	≥4 weeks post prime
Time window (days)			+14	±7	±14	±14	±30	N/A	-2, +7		N/A	N/A
Informed Consent	X	X										
Review contraindications, inclusion and exclusion criteria	X	X										
Vaccination		X	X									X
Vital signs^	X	X	(X)	(X)	(X)	(X)	(X)	X				

Attendance Number	1	2 V1	3 V2	4	5	6	7	COVID-19 Testing	COVD-19 NAAT positive+ 7 days (remote)	COVID-19 Follow-up	Extra Visit A	Extra Visit B***
											Optional	Optional if not had boost
Telephone/Video call									X	As required		
Ascertainment of adverse events		X	X	X	X	X	X	X	X	X	X	X
Diary Cards[$]		X	X									
Weekly household exposure questionnaire				ongoing								
Medical History (required at 1 timepoint prior to enrolment),	X	X	(X)		(X)	(X)	(X)	(X)	(X)			
Physical Examination (if necessary)	(X)	(X)	(X)		(X)	(X)	(X)	(X)	(X)			
Biochemistry, Haematology (mL)												
Exploratory immunology (mL)		up to 50	up to 50	up to 50	up to 50	up to 50	up to 50	up to 50[d]			(up to 50)_	(up to 50)
PAXgenes							(2.5)[P]	2.5				
Nose/Throat Swab and/or saliva sample								X				
Stool sample [a,b]									(X)	(X)		
Weekly PCR sample[a]				ongoing								
Urinary bHCG (women of childbearing potential only)	X	X										
Blood volume per visit		50	50	50	50	50	50	up to 52.5			up to 50	up to 50

Attendance Number	1	2 V1	3 V2	4	5	6	7	COVID-19 Testing	COVD-19 NAAT positive+ 7 days (remote)	COVID-19 Follow-up	Extra Visit A	Extra Visit B***
											Optional	Optional if not had boost
Cumulative blood volume%		50	100	150	200	250	300	352.5	352.5		402.5	402.5

S = screening visit; (X) = if considered necessary; ** Timeline is approximate only. Exact timings of visits relate to the day on enrolment, ie, each visit must occur at indicated number of days after enrolment ± time window. Where a second dose is administered, the window will apply to the time their last vaccination took place. ^Only temperature will be routinely measured at subsequent follow-up visits. At COVID-19 testing visits a full set observations will be taken, including respiratory rate and oxygen saturation. % Cumulative blood volume for volunteers if blood taken as per schedule, and excluding any repeat safety blood test that may be necessary. Blood volumes may vary according to local site equipment and practices. [a] Subject to site capacity, sample and test availability. [d] Optional and subject to site capacity. [$] A subset of up to 1000 volunteers will be asked to fill an e-diary with reactogenicity symptoms for 7 days only in groups 4, 6, 9 and 10. [P] Pax genes sample at D364 to be done only on participants that have attended at least one COVID-19 testing visits within the trial, an extra 2.5mL should be added to the cumulative blood volume when this applies. [b] Optional Stool sample at approximately 7 days after onset of symptoms for those who have a positive SARS-CoV-2 NAAT test result and possibly again at 14 days after symptom onset if necessary. *** For participants who previously declined to have a second dose of vaccine but now wish to complete study vaccine following unblinding

Table 12 Schedule of attendances for participants in group 11.

Attendance Number	1	2 V1	3	4 V2	5	6	7	8	COVID-19 Testing	COVD-19 NAAT positive +7 days (Remote)	COVID-19 Follow-up
Timeline** (days)		0	14	28	56 (28 days post boost)	118 (90 days post boost	210 (182 days post boost)	392 (364 days post boost)	As required	7 days post NAAT positive	
Time window (days)			±3	+14	±7	±14	±14	±30	N/A	-2, +7	
Informed Consent	X	X									
Review contraindications, inclusion and exclusion criteria	X	X									
Vaccination		X		X							
Vital signs^	X	X		(X)	(X)	(X)	(X)	(X)	X		
Telephone/Video call										X	As required
Ascertainment of adverse events		X		X	X	X	X	X	X	X	X
Diary Cards		(X)		(X)							
Weekly household exposure questionnaire		ongoing									

Attendance Number	1	2 V1	3	4 V2	5	6	7	8	COVID-19 Testing	COVD-19 NAAT positive +7 days (Remote)	COVID-19 Follow-up
Medical History (required at 1 timepoint prior to enrolment),	X	X		(X)		(X)	(X)	(X)	(X)	(X)	
Physical Examination (if necessary)	(X)	(X)		(X)		(X)	(X)	(X)	(X)	(X)	
Biochemistry, Haematology (mL)											
Exploratory immunology (mL)		up to 50	up to 50	up to 50	up to 50	up to 50	up to 50	up to 50	up to 50[d]		
PAXgenes								(2.5)[P]	2.5		
Nose/Throat Swab and/or saliva sample									X		
Stool sample [a,b]										(X)	(X)
Weekly PCR sample[a]			Ongoing								
Urinary bHCG (women of childbearing potential only)	X	X									
Blood volume per visit		50	50	50	50	50	50	52.5	up to 52.5		
Cumulative blood volume[%]	0	50	100	150	200	250	300	352.5	405	405	405

S = screening visit; (X) = if considered necessary; ** Timeline is approximate only. Exact timings of visits relate to the day on enrolment, ie, each visit must occur at indicated number of days after enrolment ± time window. Where a second dose is administered, the window will apply to the time their last vaccination took place. ^Only temperature will be routinely measured at subsequent follow-up visits. At COVID-19 testing visits a full set observations

will be taken, including respiratory rate and oxygen saturation. % Cumulative blood volume for volunteers if blood taken as per schedule, and excluding any repeat safety blood test that may be necessary. Blood volumes may vary according to local site equipment and practices. [a] Subject to site capacity, sample and test availability. [d] Optional and subject to site capacity. [P] Pax genes sample at D364 to be done only on participants that have attended at least one COVID-19 testing visits within the trial, an extra 2.5mL should be added to the cumulative blood volume when this applies. [b] Optional Stool sample at approximately 7 days after onset of symptoms for those who have a positive SARS-CoV-2 NAAT test result and possibly again at 14 days after symptom onset if necessary.

Table 13 Schedule of attendances for participants in group 12 (two dose)

Attendance Number	1[S]	2 (V1)	3	4	5	6 (V2)	7	8	9	10	11	12	COVID-19 Testing	COVD-19 NAAT positive +7 days	COVID-19 Follow-up
Timeline** (days)	≤ 90	0	3	7	14	28	31 (3 days post boost)	35 (7 days post boost)	42 (14 days post boost)	56 (28 days post boost)	182	364	As required	7 days post NAAT positive	
Time window (days)			±1	±3	±3	+14	±1	±2	±3	±7	±14	±30	N/A	±2	
Verbal Consent to discuss medical history over the phone	(X)														
Informed Consent	X														
Review contraindications, inclusion and exclusion criteria	X	X				X									
Vaccination		X				X									
Vital signs^	X	X	X	X	X	X	X	X	X	X	X	X	X	X	
Telephone/Video call															As required
Ascertainment of adverse events		X	X	X	X	X	X	X	X	X	X	X	X	X	X
Diary cards provided		X				X									
Diary cards collected						X				X					
Weekly household															

Attendance Number	1[$]	2 (V1)	3	4	5	6 (V2)	7	8	9	10	11	12	COVID-19 Testing	COVD-19 NAAT positive +7 days	COVID-19 Follow-up
exposure questionnaire															
Medical History, Physical Examination	X	(X)	(X)	(X)	(X)	(X)	(X)	(X)	(X)	(X)	(X)	(X)	(X)	(X)	
SARS-CoV-2 Ab	5														
Biochemistry[$], Haematology (mL)	5	(5)*	5	5		5	5	5		5			5	5	
Exploratory immunology[£] (mL)		up to 95		up to 95	up to 95	up to 95		up to 95	up to 95	up to 95	up to 95	up to 95	up to 95	up to 95	
PAXgenes		2.5											2.5	2.5	
Nasal/Throat Swab, and/or Saliva sample													X	(X)	
Stool sample [a,b]														(X)	(X)
Weekly PCR sample			ongoing												
Urinary bHCG (women of childbearing potential only)	X	X				X									
HBsAg, HCV Ab, (mL)	5														
HIV serology (at investigators discretion)	(X)														
CD4 count and Viral Load	Up to 20mL														
Blood volume per visit	Up to 35	Up to 102.5	Up to 5	Up to 100	Up to 95	Up to 100	Up to 5	Up to 100	Up to 95	Up to 100	Up to 95	Up to 95	up to 102.5	up to 107.5	

Attendance Number	1[S]	2 (V1)	3	4	5	6 (V2)	7	8	9	10	11	12	COVID-19 Testing	COVD-19 NAAT positive +7 days	COVID-19 Follow-up
Cumulative blood volume[%]	35	137.5	142.5	242.5	337.5	437.5	442.5	542.5	637.5	737.5	832.5	927.5	1030	1137.5	

S = screening visit; (X) = if considered necessary; ** Timeline is approximate only. Exact timings of visits relate to the day on enrolment, ie, each visit must occur at indicated number of days after enrolment ± time window. ^Vital signs at screening or pre-enrolment assessment on D0 include pulse, blood pressure, temperature, respiratory rate +/- oxygen saturation. Only temperature will be routinely measured at subsequent follow-up visits. At COVID-19 testing visits a full set observations will be taken, including respiratory rate and oxygen saturation. % Cumulative blood volume for volunteers if blood taken as per schedule, and excluding any repeat safety blood test that may be necessary. Blood volumes may vary according to local site equipment and practices. *Safety bloods should only be repeated at vaccination day if there is a period greater than 2 weeks between screening and vaccination visit; an extra 5mL of blood should be added to the overall cumulative blood volume. [a] Subject to site capacity, sample and test availability. [b] Optional Stool sample at approximately 7 days after onset of symptoms for those who have a positive SARS-CoV-2 NAAT test result and possibly again at 14 days after symptom onset if necessary.

Table 14 Unblinding and provision of treatment to controls (Protocol V19.0)

Attendance Number (boost)	Unblinding - This may occur remotely, electronically or as a trial visit (all remaining blinded participants)	Prime (controls only)	Boost (controls only)
Timeline** (days)	Following approval of protocol V19.0	Any point following unblinding	4 to 12 weeks post prime
Informed Consent		X	X
Review contraindications to ChAdOx1 nCoV-19 against the current UK Greenbook COVID-19 vaccination guidance		X	X
ChAdOx1 nCoV-19 Vaccination		X	X
Ascertainment of adverse events (SAE or AESIs only)		X	X
Participant Unblinding	X		
Exploratory immunology (mL)		up to 50 (depending on site capacity)	
Blood volume per visit	-	(50)	-

All participants (that remain blinded) will be unblinded following approval of protocol V19.0. Control participants aged 30 and above (only) will be invited to receive the MHRA approved 4-to-12 week ChAdOx1 nCoV-19 prime-boost schedule as part of provision of treatment to controls efforts. The visits in this table will happen ***concurrently*** with the remaining follow up visits and study procedures that are listed for each group (as per tables 4 to 13 above) which should remain unchanged following unblinding and provision of treatment. Contraindications to vaccination will be reviewed against the current UK Greenbook guidance on COVID-19 vaccination https://www.gov.uk/government/collections/immunisation-against-infectious-disease-the-green-book. Pregnancy testing will not be required prior to vaccination as this is not advised by the UK Greenbook.

7.3.4 Symptomatic volunteers

Participants who become symptomatic during follow-up will be instructed to call the study team who will then advise on how to proceed with clinical testing for COVID-19 if necessary, as per the trial working instructions. Participants will get weekly reminders (email or text messages) to get in touch with the study team if they present with a fever or cough or shortness of breath or anosmia/ageusia, experience any new event requiring medical attendance, or if they are admitted to hospital for any reason.

7.3.4.1 Diagnostic SARS-CoV-2 NAAT testing outside of the trial

In a (primary endpoint symptom) symptomatic illness episode, if a participant has already received a negative SARS-CoV-2 test result via a validated NAAT assay that was performed after symptom onset and where this test was carried out outside of the study, they will now (as of SA20) be considered to be a negative case without requiring further NAAT testing by the study team. In such instances, documentation relating to the result should be acquired by investigators and filed as part of the individual participant record. Additionally, if investigators believe a symptomatic participant can obtain their initial diagnostic SARS-CoV-2 NAAT test more rapidly outside of the study (for logistical or other reasons) they may advise participants be tested via that route e.g. by being advised to arrange a test through a local NHS testing clinic or similar service. Where symptomatic participants report a SARS-CoV-2 test outside of the trial but are unable to provide details of the assay and/or documentation of the test result, they will be asked to attend the trial site for a COVID-19 testing visit.

7.3.4.2 COVID-19 testing visit

Participants will be invited for a COVID-19 testing visit in two circumstances:

- They report primary endpoint symptoms AND have not already had a documented negative SARS-CoV-2 NAAT test result (including trial weekly swabs and tests performed outside of the trial) since their symptom onset
- They report primary endpoint symptoms and have had a positive SARS-CoV-2 NAAT test from their trial weekly swab test or from outside of the trial since symptom onset

At the COVID-19 testing visit, a nose/throat and immunology bloods (paxgenes, cytokine profile, PBMCs, serum and others, according to local site capacity) will be acquired. Clinical assessments may be performed at COVID-19 testing visits at the discretion of investigators. Symptomatic volunteers may be regularly reviewed over the phone or via video call using a smartphone or computer app if clinically appropriate. Participants who have a positive NAAT will be reviewed by phone for symptom severity data at approximately 7 days post positive swab. Closer follow-up and safety monitoring may be carried out by local trial teams if felt this is clinically indicated. Symptomatic participants with a positive NAAT test outside of the trial or as part of their trial weekly swab tests will be asked to attend for a COVID-19 testing visit in order to take a further swab to facilitate SARS-CoV-2 sequencing and additionally to acquire immunology bloods.

7.3.4.3 *COVID-19 Testing plus 7 day phone call*

For symptomatic participants with a positive SARS-CoV-2 NAAT test, a remote follow up (via phone or other appropriate means) will take place at approximately 7 days (see schedule of procedure tables for window, section **Error! Reference source not found.**) from the date of their first positive NAAT test in that illness episode. The purpose of this remote follow up is to capture further symptom severity data.

7.3.4.4 *Positive SARS-CoV-2 NAAT test and vaccination*

Participants who develop COVID-19 symptoms and have a positive NAAT test after the first vaccination can only receive a booster dose after a minimum 4 weeks interval from their first NAAT positive test, provided their symptoms have significantly improved. The decision to proceed with booster vaccinations in those cases will be at clinical discretion of the investigators. For participants who are asymptomatic and have a positive NAAT test, a minimum of 2 weeks from first NAAT positivity will be required before boosting.

7.3.5 Weekly PCR samples

Participants may be asked provide a saliva and/or a nose/throat self-swab sample every week from the date of enrolment, which will be posted and processed in the Department of Health and Social Care’s community testing programme. This process will be detailed in trial specific instructions. Weekly PCR samples will be collected and processed depending on test availability, laboratory

capacity, and other local screening programmes, which will determine the number of participants asked to provide weekly samples.

Participants with a positive test result from home testing (self-swabbing) will be notified of their test results by the Department of Health and Social Care community testing programme and advised to self-isolate as per current government guidance. No additional trial follow up of these participants will occur at this time unless they become symptomatic. Symptomatic volunteers will then be reviewed follow the procedures outlined in section 7.3.5 above.

7.3.6 Stool samples

Those participants who have a SARS-CoV-2 positive NAAT test result, may be asked to provide a stool sample at approximately 7 days after symptom onset or positive NAAT result if asymptomatic and 14 days after the first sample if necessary, as per trial specific instructions. Samples will be processed to look at differences in viral shedding between the investigational vaccine and control arms, and to measure calprotectin levels as a marker of gastrointestinal inflammation. These samples will be collected and processed depending on test availability, laboratory capacity, and will not be compulsory to the volunteers. Further exploratory immunology and microbiology tests may be conducted at the investigators' discretion.

7.4 Household Weekly Questionnaire (optional)

Participants will be asked to record information on a weekly basis about illnesses amongst household contacts and friends, their contact with the general public, and infection control procedures. This will be optional.

Volunteers will be asked to enter data in a diary from baseline to the end of the follow-up period. This will be recorded via a web-based electronic diary to which participants will be provided access at baseline. Participants working in clinical areas will be exempt from this questionnaire to reduce the study load on these participants whose exposure to COVID-19 is likely to be in the workplace rather than the home or community.

7.5 Medical notes review

With the participant's consent, the study team will request access to medical notes or submit a data collection form for completion by attending clinical staff on any medically attended COVID-19

episodes. Any data which are relevant to ascertainment of efficacy endpoints and disease enhancement (AESI) will be collected. These are likely to include, but not limited to, information on ICU admissions, clinical parameters such as oxygen saturation, respiratory rates and vital signs, need for oxygen therapy, need for ventilatory support, imaging and blood tests results, amongst others.

7.6 Randomisation, blinding and code-breaking

Participants will be randomised to investigational vaccine or MenACWY in a 3:1:3:1 (Groups 1 and 7), 5:1:5:1 (Groups 2 and 8) and 1:1 (groups 3, 4, 5a, 5b, 5c, 6, 9 and 10) and 5:1 (group 5d) allocation, using block randomisation. Groups 5e, 5f, 11 and 12 will be open-label and randomisation to investigational vaccine or comparator will not apply.

Participants will be blinded to the arm they have been allocated to, whether investigational vaccine or MenACWY. The trial staff administering the vaccine will not be blinded. Vaccines will be prepared out of sight of the participant and syringes will be covered with an opaque object/material until ready for administration to ensure blinding.

If the clinical condition of a participant necessitates breaking the code, this will be undertaken according to a trial specific working instruction and group allocation sent to the attending physician, if unblinding is thought to be relevant and likely to change clinical management.

Additional steps may be taken to keep clinical investigators assessing primary endpoints blinded to group allocation, where this is possible and practical to do so. A designated member of the clinical team may be unblinded for the purposes of safety reporting procedures. All data from participants with NAAT-positive swabs will be assessed for inclusion in the primary efficacy analysis by two blinded assessors who will independently review each case according to pre-specified criteria as detailed in the statistical analysis plan, to classify each for inclusion in the primary and secondary outcomes. Protocol V19.0: Exploratory COVID-19 disease endpoints will be assessed by two trial team following pre-specified criteria, rather than independent blinded assessors.

In the event of other SARS-CoV-2 vaccines being approved for use by the MHRA, participants will be supported to access SARS-CoV-2 vaccines as early as possible, particularly the control group. To allow for a baseline consideration prior to receipt of a different vaccine, all participants who are unblinded and wish to receive an approved or authorised vaccine in line with government prioritisation will be asked to attend an extra visit visit before receiving the deployed vaccine where practical to do so.

This is to ensure that they have a complete status before they become unevaluable for the trial arm they were in.

Those who are eligible (as per government prioritisation strategy) and have been invited to receive an approved or licensed SARS-CoV-2 vaccine can request to be unblinded, all participants will be kept in the study regardless of the combination of COVID-19 vaccines received.

Participants will be asked about the vaccine they received and date of administration which will be recorded along with other vaccines received in a specific externally administered vaccination case report form. Follow up visits will continue as per the participants previous schedule and their data will contribute to ongoing safety monitoring according to the vaccine combinations they have received. Participants will be censored in the analysis of efficacy endpoints at the time of their unblinding and vaccination, but will contribute to exploratory immunological analyses which are descriptive or observational according to the vaccines they have received. When participants have been unblinded they and the GP will receive a letter detailing the vaccines received in the study.

7.6.1 Unblinding of all trial participants (protocol V19.0)

All trial participants will be unblinded following the approval of protocol V19.0. This follows the earlier completion of the primary efficacy analysis, the significant level of unblinding that has occurred due to the national vaccine rollout already within the trial and the fall in transmission of SARS-CoV-2 within the UK. Following unblinding and provision of COVID-19 vaccination (in or outside the study), participants will continue to follow all other study procedures and should continue to complete the visit schedule for their group. This will enable further analysis of immunogenicity, safety and immunological correlate of protection analysis.

Provision of treatment for controls is described in section 8.11

7.6.2 Unblinded participants who are eligible to receive an approved or licensed SARS-CoV-2 vaccine

For those who choose to receive a deployed vaccine:

An extra visit A may be arranged (where possible and practical to do so) for those who are offered and are willing to accept a SARS-CoV-2 approved or licensed vaccine where a blood sample for immunogenicity assessment (up to 50mL) may be drawn, regardless of which arm of the study they

have been allocated to (ChAdOx1 nCoV-19 or MenACWY) or their COVID-19 status. SAEs and AESIs will be collected at this point. Participants will then follow their normal schedule of attendances according to the group they were previously randomised or allocated to.

For those who have received a single dose of the ChAdOx1 nCov-19 vaccine:

Those who are eligible to receive an approved or licensed SARS-CoV-2 vaccine, and who after unblinding are found to have received only a single dose of ChAdOx1 nCoV-19 will be given an option to receive a booster standard dose (ChAdOx1 nCoV-19 3.5-6.5x10^{10} vp) at an extra visit B, instead of a deployed vaccine, as per national policy. The safety and benefit of mixed vaccine schedules is unknown. The extra visit B will take place ≥4 weeks post prime and should be conducted at the earliest possibility. The participant will be counselled on the timings of the planned vaccination and will be able to decide which vaccine to choose. SAEs and AESIs will be collected at this point. A blood sample for immunogenicity assessment (up to 50mL) may be drawn and participants will then follow their normal schedule of attendances according to the group they were previously randomised or allocated to. When participants have been unblinded they and the GP will receive a letter detailing the vaccines received in the study.

8 INVESTIGATIONAL PRODUCT

8.1 Description of ChAdOx1 nCoV-19

ChAdOx1 nCoV-19 vaccine consists of the replication-deficient simian adenovirus vector ChAdOx1, containing the structural surface glycoprotein (Spike protein) antigens of SARS-CoV-2.

8.2 Supply

ChAdOx1 nCoV-19 has been formulated and vialed at Advent S.r.I. (Pomezia, Italy). Labelling has been done at the Clinical BioManufacturing Facility (University of Oxford) or Advent S.r.I. (Pomezia, Italy). It will be certified by a Qualified Person (QP) at the Clinical BioManufacturing Facility (University of Oxford) before release and transfer to the clinical site.

ChAdOx1 nCoV-19 (AZD1222) has been formulated at Cobra Biologics Ltd, vialed at Symbiosis Pharmaceutical Services, and labelled and packaged at Thermo Fisher Scientific (Hertfordshire, United Kingdom). It will be certified by a Qualified Person (QP) at the MedImmune Pharma, BV

(Nijmegen, The Netherlands) or MedImmune Ltd (Cambridge, United Kingdom) before release and transfer to the clinical site.

ChAdOx1 nCoV-19 (Covishield) has been formulated and vialed at Serum Institute of India Pvt. Ltd. Labelling and QP certification was done at Bilthoven Biologicals (Biltoven, Netherlands) before release and transfer to the clinical site.

8.3 Storage

The vaccine manufactured by Advent is stored at nominal -80°C (+/- 20 ^{o}C) in a secure freezer, at the clinical site. The vaccines manufactured by Cobra Biologics Ltd and Serum Institute of India are stored at 2-8°C in a secure fridge, at the clinical site. All movements of the study vaccines will be documented in accordance with existing standard operating procedure (SOP). Vaccine accountability, storage, shipment and handling will be in accordance with relevant SOPs and forms. To allow for large number of participants to receive the vaccine in a short time period, additional clinic locations may be used. In this instance vaccines will be transported in accordance with local SOP's and approvals as required

8.4 Administration

On vaccination day, ChAdOx1 nCoV-19 will be allowed to thaw to room temperature and will be administered in accordance with trial specific instructions or stored at 2-8 for a maximum of up to 48 hours, where multiple doses are required from a single vial The vaccine manufactured by Cobra Biologics is a multi-dose vial which is stored at 2-8 degrees and does not require thawing. If the vaccine is stored outside of 2-8 it must be used within 6 hours. If stored at 2-8°C after the first vial puncture, it can be used within 48 hours. The Covishield vaccine is stored at 2-8°C at all times unless it is being used for the dose preparation process. Total time from needle puncture of the vaccine vial to the start of administration must not exceed 6 hours at 2-8°C. If preparation time exceeds the time limit, a new dose must be prepared. The vaccine will be administered intramuscularly into the deltoid of the non-dominant arm (preferably).). Prior to protocol V19.0: All volunteers will be observed in the unit for a minimum of 15 minutes (+15 minutes) after vaccination. Following protocol V19.0 approval: In line with UK Greenbook recommendations, participants do not need to be routinely observed following either prime (1st) or boost (2nd) dose of ChAdOx1 nCoV-19. During administration of the investigational products, Advanced Life Support drugs and resuscitation equipment will be

immediately available for the management of anaphylaxis. Vaccination will be performed and the IMPs handled according to the relevant SOPs.

8.5 Rationale for selected dose

The dose to be administered in this trial have been selected on the basis of clinical experience with the ChAdOx1 adenovirus vector expressing different inserts and other similar adenovirus vectored vaccines (eg. ChAd63).

A first-in-man dose escalation study using the ChAdOx1 vector encoding an influenza antigen (FLU004), safely administered ChAdOx1 NP+M1 at doses ranging from 5 x 10^8 to 5 x 10^{10} vp. Subsequent review of the data identified an optimal dose of 2.5 x 10^{10} vp balancing immunogenicity and reactogenicity. This dose has subsequently been given to over hundreds of volunteers in numerous larger phase 1 studies at the Jenner Institute. ChAdOx1 vectored vaccines have thus far demonstrated to be very well tolerated. The vast majority of AEs have been mild-moderate and there have been no SARs until this date.

Another simian adenovirus vector (ChAd63) has been safely administered at doses up to 2 x 10^{11} vp with an optimal dose of 5 x 10^{10} vp, balancing immunogenicity and reactogenicity.

MERS001 was the first clinical trial of a ChAdOx1 vectored expressing the full-length Spike protein from a separate, but related betacoronavirus. ChAdOx1 MERS has been given to 31 participants to date at doses ranging from 5x10^9 vp to 5x10^{10} vp. Despite higher reactogeniticy observed at the 5x10^{10} vp, this dose was safe, with self-limiting AEs and no SARs recorded. The 5x10^{10} vp was the most immunogenic, in terms of inducing neutralising antibodies against MERS-CoV using a live virus assay [16]. Given the immunology findings and safety profile observed with a ChAdOx1 vectored vaccine against MERS-CoV, the 5x10^{10} vp dose was chosen for ChAdOx1 nCoV-19.

The Clinical BioManufacturing Facility (CBF), who manufactured and tested batches 02P20-01 and 02P20-02 for the COV001 trial, determined vp/mL for Advent manufactured batch(es). This was done using a spectrophotometry-based methodology documented in their internal SOP (SOP A104). The doses to be administered in COV002, on Advent manufactured batches, will be determined by both methods (Abs260 and qPCR). Overseas studies conducted on Advent batch(es) will be dosed based on Advent's qPCR method. The University of Oxford is also performing a number of for information only tests for ChAdOx1 nCoV-19 batch(es) manufactured by Advent to demonstrate comparability

between the different manufacturing processes and suitability of a reconstitution process involving dilution of the product with 0.9 % (w/v) saline that will be managed using local clinical trial SOPs available at each site/location.

For Advent Lot Number K.0007 the concentration is $1.7x10^{11}$ vp/mL (qPCR) which has been assessed by the CBF as equivalent to $3.89x10^{11}$ vp (Abs 260).

An analytical comparability assessment of ChAdOx1 nCoV-19 (AZD1222) manufactured by CBF, Advent and Cobra Biologics was conducted using a comprehensive set of physiochemical and biological release and characterization tests. In order to support the analytical comparability assessment, A260 testing of Advent's process (K.0007, K.0008, and K.0009 lots) was performed, where corrections to the absorbance due to excess polysorbate 80 were made to compensate for polysorbate 80 concentrations above the formulation target of 0.1% (w/v).

Differences in strength related attributes (ie, virus particle concentration, virus genome concentration, and infectious virus concentration) are noted. These differences in strength is further examined for potential impact on clinical dosing. The target clinical dosage of CBF's product is 5×10^{10} viral particles per dose based on vp/mL concentration determined by UV spectroscopy (A260), whereas that of Advent's product is 5×10^{10} viral genome copies per dose based on vg/mL concentration determined by qPCR. The target clinical dosage of Symbiosis' product is $3.5 – 6.5 \times 10^{10}$ viral particles per dose based on the vp/mL concentration determined by A260, with a 0.5 mL dosing volume. This dosing range is based on a target 5×10^{10} viral particles per dose and a ± 30% range to take into account process and method variabilities. The planned clinical dosage of Symbiosis' product is compared to that of CBF and Advent products, the resulting Symbiosis' product dosage at 0.5 mL for lot 20481A is somewhat lower in total viral particle per dose (20% from the lower range limit), slightly higher in total viral genome copies per dose (12% from the higher range limit), and slightly lower in total infectious particle per dose (8% from the lower range limit). These differences are considered to be comparable to or within the variabilities from the analytical methods used in concentration determination (A260, qPCR, and infectivity) and the dosing volumes during clinical administration. In summary, with a 0.5 mL dosing volume for Symbiosis' product, strength difference from CBF and Advent products is not expected to have significant clinical impact in terms of reactogenicity and immunogenicity/efficacy.

Manufacturing of ChAdOx1 nCoV-19 is also taking place at Serum Institute of India (SII). The vaccine is manufactured under the named ChAdOx1 nCoV-19 (Covishield). The target clinical dose for ChAdOx1 nCoV-19 (Covishield) is nominal 5×10^{10} viral particles per dose based on vp/mL concentration determined by UV spectroscopy. An analytical comparability assessment of ChAdOx1 nCoV-19 manufactured by Advent, Cobra Biologics and SII was conducted using a comprehensive set of physiochemical and biological release and characterization tests. The details are available in the IMPD. The planned clinical dosage of SII's product is compared to that of Cobra and Advent products, with a dosing volume of 0.5 mL.

Table 14 Clinical Strengths of ChAdOx1 nCoV-19 (AZD1222) Drug Product

Strength Attribute	CBF		Advent			Cobra
	Lot 02P20-01	Lot 02P20-02	Lot K.0007	Lot K.0008	Lot K.0009	Lot 20481A
**Concentration**						
Virus particle concentration (A_{260}) (vp/mL)	1.49×10^{11}	1.22×10^{11}	3.12×10^{11}	3.16×10^{11}	2.45×10^{11}	0.8×10^{11}
Virus genome concentration (qPCR) (vg/mL)	1.7×10^{11}	Not tested	1.7×10^{11}	2.1×10^{11}	1.4×10^{11}	1.3×10^{11}
Infectious particle concentration (ifu/mL) [a]	2.6×10^{9}	Not tested	2.9×10^{9}	3.0×10^{9}	2.4×10^{9}	1.3×10^{9}
**Target Clinical Dosage**						
Equivalent DP volume per dose (mL)	0.34	0.41	0.294	0.235	0.356	0.50
Dosing of virus particle (vp/dose)	5.1×10^{10}	5.0×10^{10}	9.2×10^{10}	7.4×10^{10}	8.7×10^{10}	4.0×10^{10}
Dosing of viral genome (vg/dose)	5.8×10^{10}	NA	5.0×10^{10}	4.9×10^{10}	5.0×10^{10}	6.5×10^{10}
Dosing of infectious particle (ifu/dose)	8.8×10^{8}	NA	8.5×10^{8}	7.1×10^{8}	8.5×10^{8}	6.5×10^{8}

[a] ifu = infectious units; NA = not applicable; vp = virus particle; vg = virus genome

[a] **Testing performed using the Advent infectivity assay.**

8.6 Minimising environmental contamination with genetically modified organisms (GMO)

The study will be performed in accordance with the current version of the UK Genetically Modified Organisms (Contained Use) Regulations. Approved SOPs will be followed to minimise dissemination of the recombinant vectored vaccine virus into the environment. GMO waste will be inactivated according to approved SOPs.

8.7 Control vaccine

Participants who are allocated to the control groups will receive one or two injections of MenACWY vaccine instead of ChAdOx1 nCoV-19. Either of the two licensed quadrivalent protein -polysaccharide conjugate vaccine MenACWY vaccines will be used, i.e.:

- Nimenrix (Pfizer). The licensed posology of this vaccine for those over 6 months of age is a single (0.5ml) intramuscular dose, containing 5mcg each of *Neisseria meningitidis* group A, C, W and Y polysaccharide, each conjugated to 44 mcg tetanus toxoid carrier protein.
- Menveo (Glaxosmithkline). The licensed posology of this vaccine for those 2 years of age and over is a single (0.5ml) intramuscular dose, containing
    - 10 mcg meningococcal group A polysaccharide, conjugated to 16.7 to 33.3 mcg *Corynebacterium diphtheriae* CRM_{197} protein
    - 5mcg meningococcal group C polysaccharide, conjugated to 7.1 to 12.5 mcg *C. diphtheriae* CRM_{197} protein
    - 5mcg meningococcal group W polysaccharide, conjugated to 3.3 to 8.3 mcg *C. diphtheriae* CRM_{197} protein
    - 5mcg meningococcal group Y polysaccharide, conjugated to 5.6 to 10.0 mcg *C. diphtheriae* CRM_{197} protein

The summary of product characteristics for both vaccines allows for administration of a booster dose if indicated by ongoing risk, therefore allows for the two doses administered to a subset of participants in this study. Similarly, previous receipt of either vaccine (or a plain polysaccharide quadrivalent meningococccal A, C, W and Y vaccine) will not be a contraindication to receiving a further vaccine in this study.

Participants will be blinded as to which injection they are receiving. A vaccine accountability log of MenACWY will be maintained at each trial site. There will be no additional labelling of these vaccines beyond their licensed packaging.

MenACWY will be stored in a locked (or access controlled) refrigerator (2°C – 8°C) at the sites, as per SmPC.

8.8 Compliance with Trial Treatment

All vaccinations will be administered by the research team and recorded in the CRF. The study medication will be at no time in the possession of the participant and compliance will not, therefore, be an issue.

8.9 Accountability of the Trial Treatment

Accountability of the IMP and MenACWY will be conducted in accordance with the relevant SOPs.

8.10 Concomitant Medication

As set out by the exclusion criteria, volunteers may not enter the study if they have received: any vaccine in the 30 days prior to enrolment, any investigational product within 30 days prior to enrolment or if receipt is planned during the study period, or if there is any chronic use (>14 days) of any immunosuppressant medication within 6 months prior to enrolment or if receipt is planned at any time during the study period (topical steroids are permitted).

Participants on continuous use of oral anticoagulants, such as coumarins and related anticoagulants (i.e. warfarin) or novel oral anticoagulants (i.e. apixaban, rivaroxaban, dabigatran and edoxaban) will be excluded from this trial, as per the exclusion criteria.

Participants in groups 4 and 6 will be advised to take Paracetamol after vaccination at 1g every 4-6 hours for the first 24 hours (maximum dose 4g within 24 hours). This will not be a requirement for study participation, and participants will have the option to not follow the advice.

Participants who become eligible for a SARS-CoV-2 vaccine according to government guidance will be able to receive the vaccine as recommended. We will advise participants to allow a minimum period of 2 weeks between vaccination with MenACWY and the SARS-CoV-2 vaccine for those in the control

group and 3 weeks between vaccination with ChAdOx1 nCoV-19 and the deployed SARS-CoV-2 vaccine. Participants will be asked to record the date and type of vaccine received.

8.11 Provision of Treatment for Controls

If this vaccine is proven to be efficacious following analysis of the primary endpoint and if the DSMB agrees, participants allocated to MenACWY group may be offered the IMP, should extra doses become available.

(Protocol V19.0) Following unblinding, control participants aged 30 and above will be offered ChAdOx1 nCoV-19 in the approved MHRA 4-to-12 week, 2-dose schedule (table 14). Participants will remain enrolled in the study and continue the follow up visit schedule listed for their sub-group *concurrently* with the provision of treatment prime and boost visits.

Due to the updated guidance relating to the emerging association of thrombosis with thrombocytopenia and ChAdOx1 nCoV-19, trial participants aged 29 and under will not be offered (prime) vaccinations with ChAdOx1 nCoV-19. These individuals will instead be advised to await vaccination under the national rollout program.

9 ASSESSMENT OF SAFETY

Safety will be assessed by the frequency, incidence and nature of AEs and SAEs arising during the study.

9.1 Definitions

9.1.1 Adverse Event (AE)

An AE is any untoward medical occurrence in a volunteer, which may occur during or after administration of an IMP and does not necessarily have a causal relationship with the intervention. An AE can therefore be any unfavourable and unintended sign (including any clinically significant abnormal laboratory finding or change from baseline), symptom or disease temporally associated with the study intervention, whether or not considered related to the study intervention.

9.1.2 Adverse Reaction (AR)

An AR is any untoward or unintended response to an IMP. This means that a causal relationship between the IMP and an AE is at least a reasonable possibility, i.e., the relationship cannot be ruled out. All cases judged by the reporting medical Investigator as having a reasonable suspected causal relationship to an IMP (i.e. possibly, probably or definitely related to an IMP) will qualify as AR.

Adverse events that may be related to the IMP are listed in the Investigator's Brochure for each product.

9.1.3 Serious Adverse Event (SAE)

An SAE is an AE that results in any of the following outcomes, whether or not considered related to the study intervention.

- Death
- Life-threatening event (i.e., the volunteer was, in the view of the Investigator, at immediate risk of death from the event that occurred). This does not include an AE that, if it occurred in a more severe form, might have caused death.
- Persistent or significant disability or incapacity (i.e., substantial disruption of one's ability to carry out normal life functions).
- Hospitalisation or prolongation of existing hospitalisation, regardless of length of stay, even if it is a precautionary measure for continued observation. Hospitalisation (including inpatient or outpatient hospitalisation for an elective procedure) for a pre-existing condition that has not worsened unexpectedly does not constitute a serious AE.
- An important medical event (that may not cause death, be life threatening, or require hospitalisation) that may, based upon appropriate medical judgment, jeopardise the volunteer and/or require medical or surgical intervention to prevent one of the outcomes listed above. Examples of such medical events include allergic reaction requiring intensive treatment in an emergency room or clinic, blood dyscrasias, or convulsions that do not result in inpatient hospitalisation.
- Congenital anomaly or birth defect.

9.1.4 Serious Adverse Reaction (SAR)

An AE that is both serious and, in the opinion of the reporting Investigator or Sponsors, believed to be possibly, probably or definitely due to an IMP or any other study treatments, based on the information provided.

Reporting of SARs will also apply to concomitant medications, including any other approved or licensed SARS-CoV-2 vaccine, where there is the possibility of an interaction between study interventions and those concomitant medications.

9.1.5 Suspected Unexpected Serious Adverse Reaction (SUSAR)

A SAR, the nature and severity of which is not consistent with the information about the medicinal product in question set out in the IB.

9.2 Expectedness

No IMP related SAEs are expected in this study. All SARs will therefore be reported as SUSARs.

9.3 Foreseeable Adverse Reactions:

The foreseeable ARs following vaccination with ChAdOx1 nCoV-19 include injection site pain, tenderness, erythema, warmth, swelling, induration, pruritus, myalgia, arthralgia, headache, fatigue, fever, feverishness, chills, malaise, nausea and vomiting.

9.4 Adverse Events of Special Interest

- Disease enhancement following vaccination with ChAdOx1 nCoV-19 will be monitored. Severe COVID-19 disease will be defined using clinical criteria. Detailed clinical parameters will be collected from medical records and aligned with agreed definitions as they emerge. These are likely to include, but are not limited to, oxygen saturation, need for oxygen therapy, respiratory rate, need for ventilatory support, imaging and blood test results, amongst other clinically relevant parameters.
- Acute respiratory distress, pneumonitis, acute cardiac injury, arrhythmia, septic-shock like syndrome and acute kidney injury related with COVID-19 disease will be monitored from medical records review of hospitalised participants.
- Neurological events of >Grade 2 severity

- Eosinophilia as a marker skewed Th2 responses will be routinely monitored in participants attending their COVID-19 testing and follow-up visits. Marked eosinophilia of ≥ 1.5 x109/L will be reported as an SAE.
- AESI relevant to vaccination in general will also be monitored such as: generalised convulsion, Guillain-Barre Syndrome (GBS), Acute Disseminated Encephalomyelitis (ADEM), Thrombocytopenia, Anaphylaxis, Vasculitides in addition to serious solicited AEs will be monitored.
- (Protocol V19.0) The following conditions will be recorded as AESIs:
  - Cerebral Venous Sinus Thrombosis
  - Heparin-Induced Thrombocytopenia
  - Major thrombosis with concurrent thrombocytopenia

9.5 Causality

For every AE, an assessment of the relationship of the event to the administration of the vaccine will be undertaken by the CI-delegated clinician. An interpretation of the causal relationship of the intervention to the AE in question will be made, based on the type of event; the relationship of the event to the time of vaccine administration; and the known biology of the vaccine therapy (Table 13). Alternative causes of the AE, such as the natural history of pre-existing medical conditions, concomitant therapy, other risk factors and the temporal relationship of the event to vaccination will be considered and investigated. Causality assessment will take place during planned safety reviews, interim analyses (e.g. if a holding or stopping rule is activated) and at the final safety analysis, except for SAEs, which should be assigned by the reporting investigator, immediately, as described in SOP OVC005 Safety Reporting for CTIMPs.

0	**No Relationship**	No temporal relationship to study product ***and***<br>Alternate aetiology (clinical state, environmental or other interventions); ***and***<br>Does not follow known pattern of response to study product

1	**Unlikely**	Unlikely temporal relationship to study product ***and***<br>Alternate aetiology likely (clinical state, environmental or other interventions) ***and***<br>Does not follow known typical or plausible pattern of response to study product.
2	**Possible**	Reasonable temporal relationship to study product; ***or***<br>Event not readily produced by clinical state, environmental or other interventions; ***or***<br>Similar pattern of response to that seen with other vaccines
3	**Probable**	Reasonable temporal relationship to study product; ***and***<br>Event not readily produced by clinical state, environment, or other interventions ***or***<br>Known pattern of response seen with other vaccines
4	**Definite**	Reasonable temporal relationship to study product; ***and***<br>Event not readily produced by clinical state, environment, or other interventions; ***and***<br>Known pattern of response seen with other vaccines

Table 15. Guidelines for assessing the relationship of vaccine administration to an AE.

9.6 Reporting Procedures for All Adverse Events

All local and systemic AEs occurring in the 28 days following each study vaccination observed by the Investigator or reported by the volunteer, whether or not attributed to study medication, will be recorded in electronic diaries or study database. AEs occurring following vaccination at Extra Visit B will not be recorded, unless meeting criteria for AESI or SAE. Only SAEs and AESI will be recorded for participants who are unblinded and receive an approved/licensed COVID-19 vaccine either as part of the national roll-out strategy OR vaccines administered as part of provision of treatment to controls (introduced in protocol V19.0). Participants in a subset of groups 4, 6, 9 and 10 will be asked to record local and systemic AE's for 7 days following vaccination in the electronic diary. Any unsolicited non serious AEs reported by participants in any group at subsequent routine visits will be documented on the eCRF until at least 6 months of safety data has accrued for ChAdOx1 nCOV19 (1st November 2020). All AEs that result in a volunteer's withdrawal from the study will be followed up until a satisfactory

resolution occurs (if the volunteer consents to this), or until a non-study related causality is assigned. SAEs and Adverse Events of Special Interest will be collected throughout the entire trial period.

9.7 Assessment of severity

The severity of clinical and laboratory adverse events will be assessed according to scales based on FDA toxicity grading scales for healthy and adolescent volunteers enrolled in preventive vaccine clinical trials, listed in the study specific working instructions and tables 14-16 below.

Adverse Event	Grade	Intensity
Pain at injection site	1	Pain that is easily tolerated
	2	Pain that interferes with daily activity
	3	Pain that prevents daily activity
	4	A&E visit or hospitalization
Tenderness	1	Mild discomfort to touch
	2	Discomfort with movement
	3	Significant discomfort at rest
	4	A&E visit or hospitalization
Erythema at injection site*	1	2.5 - 5 cm
	2	5.1 - 10 cm
	3	>10 cm
	4	Necrosis or exfoliative dermatitis
Induration/Swelling at injection site	1	2.5 – 5 cm and does not interfere with activity
	2	5.1 - 10 cm or interferes with activity
	3	>10 cm or prevents daily activity
	4	Necrosis

Table 16**. Severity grading criteria for local adverse events** *erythema ≤2.5cm is an expected consequence of skin puncture and will therefore not be considered an adverse event

Vital Signs	Grade 1 (mild)	Grade 2 (moderate)	Grade 3 (severe)	Grade 4 Potentially Life threatening
Fever (oral)	38.0°C - 38.4°C	38.5°C – 38.9°C	39.0°C - 40°C	> 40°C
Tachycardia (bpm)*	101 - 115	116 – 130	>130	A&E visit or hospitalisation for arrhythmia
Bradycardia (bpm)**	50 – 54	45 – 49	<45	A&E visit or hospitalisation for arrhythmia
Systolic hypertension (mmHg)	141 - 150	151 – 155	≥155	A&E visit or hospitalization for malignant hypertension
Diastolic hypertension (mmHg)	91 - 95	96 – 100	>100	A&E visit or hospitalization for malignant hypertension
Systolic hypotension (mmHg)***	85 - 89	80 – 84	<80	A&E visit or hospitalization for hypotensive shock
Respiratory Rate –breaths per minute	17 - 20	21-25	>25	Intubation

Table 17***. Severity grading criteria for physical observations (applies to adults only).*** **Taken after ≥10 minutes at rest **When resting heart rate is between 60 – 100 beats per minute. Use clinical judgement when characterising bradycardia among some healthy subject populations, for example, conditioned athletes. ***Only if symptomatic (e.g. dizzy/ light-headed)*

**GRADE 0**	None
**GRADE 1**	Mild: Transient or mild discomfort (< 48 hours); No interference with activity; No medical intervention/therapy required
**GRADE 2**	Moderate: Mild to moderate limitation in activity – some assistance may be needed; no or minimal medical intervention/therapy required
**GRADE 3**	Severe: Marked limitation in activity, some assistance usually required; medical intervention/therapy required.
**GRADE 4**	Potentially Life-threatening: requires assessment in A&E or hospitalisation

Table 18. *Severity grading criteria for local and systemic AEs.* NB: A&E assessment in itself does not constitute a SAE. Refer to 9.1.3 for SAE definition

9.8 Reporting Procedures for Serious AEs

In order to comply with current regulations on SAE reporting to regulatory authorities, the event will be documented accurately and notification deadlines respected. SAEs will be reported on the SAE forms to members of the study team immediately the Investigators become aware of their occurrence, as described in SOP OVC005 Safety Reporting for CTIMPs. Copies of all reports will be forwarded for review to the Chief Investigator (as the Sponsor's representative) within 24 hours of the Investigator being aware of the suspected SAE. The DSMB will be notified of SAEs that are deemed possibly, probably or definitely related to study interventions; the chair of DSMB will be notified immediately (within 24 hours) of the sponsor being aware of their occurrence. SAEs will not normally be reported immediately to the ethical committee(s) unless there is a clinically important increase in occurrence rate, an unexpected outcome, or a new event that is likely to affect safety of trial volunteers, at the discretion of the Chief Investigator and/or DSMB. In addition to the expedited reporting above, the Investigator shall include all SAEs in the annual Development Safety Update Report (DSUR) report.

Grade 4 laboratory AEs should be reported as SAEs and under the category of outcome of an important medical event. A&E attendances should not routinely be reported as SAEs unless they meet the SAE definition described above.

Cases falling under the Hy's Law should be reported as SAEs. A Hy's Law Case is defined by FDA Guidance for Industry "Drug-Induced Liver Injury: Premarketing Clinical Evaluation" (2009). Any study participant with an increase in Aspartate Aminotransferase (AST) or **Alanine Aminotransferase (ALT) ≥ 3x Upper Limit of Normal (ULN) together with Total Bilirubin ≥2xULN**, **where no other reason can be found to explain the combination of these abnormal results**, e.g., elevated serum alkaline phosphatase (ALP) indicating cholestasis, viral hepatitis A, B or C, another drug capable of causing the observed injury, amongst others.

9.9 Reporting Procedures for SUSARS

All SUSARs will be reported by the sponsor delegate to the relevant Competent Authority and to the REC and other parties as applicable. For fatal and life-threatening SUSARS, this will be done no later than 7 calendar days after the Sponsor or delegate is first aware of the reaction. Any additional relevant information will be reported within 8 calendar days of the initial report. All other SUSARs will be reported within 15 calendar days.

Principal Investigators will be informed of all SUSARs for the relevant IMP for all studies with the same Sponsor, whether or not the event occurred in the current trial.

9.10 Development Safety Update Report

A Development Safety Update Report (DSUR) will be prepared annually, within 60 days of the anniversary of the first approval date from the regulatory authority for each IMP. The DSUR will be submitted by the CI to the Competent Authority, Ethics Committee, HRA (where required), Host NHS Trust and Sponsor.

9.11 Procedures to be followed in the event of abnormal findings

Eligibility for enrolment in the trial in terms of laboratory findings will be assessed by clinically qualified staff. Abnormal clinical findings from medical history, examination or blood tests will be assessed as to their clinical significance throughout the trial. Laboratory AEs will be assessed using specific toxicity grading scales adapted from the FDA Toxicity Grading Scale for Healthy Adult and Adolescent Volunteers Enrolled in Preventive Vaccine Clinical Trials. Paediatric laboratory AEs will be assessed using site specific paediatric laboratory reference ranges, values outside of these age

specific ranges will be reviewed by a study clinician to determine clinical significance. If a test is deemed clinically significant, it may be repeated, to ensure it is not a single occurrence. If a test remains clinically significant, the volunteer will be informed and appropriate medical care arranged as appropriate and with the permission of the volunteer. Decisions to exclude the volunteer from enrolling in the trial or to withdraw a volunteer from the trial will be at the discretion of the Investigator

9.12 Interim Safety Reviews

The safety profile will be assessed on an on-going basis by the Investigators. The CI and relevant Investigators (as per the trial delegation log) will also review safety issues and SAEs as they arise.

Immunopathology data from pre-clinical and phase 1 studies will be assessed by the CI, relevant investigators and the DSMB as soon as they are available and before any volunteers receive a dose of the IMP.

The DSMB will evaluate frequency of events, safety and efficacy data every 4-8 weeks and/or as required. The DSMB will make recommendations concerning the conduct, continuation or modification of the study.

In particular, the DSMB will review the data at the following key timepoints:

- Before vaccination of the first participant (all accumulated data available will be reviewed from COV001, with a minimum 4 weeks safety and immunogenicity data from the first 54 participants receiving the IMP and all accumulated data from the animal studies)
- Prior to expansion of the recruitment of groups 4 and 6 in those aged older than 55 years (data will be reviewed from groups 1 and 2)

9.13 Data Safety Monitoring Board

The Data Safety Monitoring Board that is in place for COV001 will also oversee COV002 and review data from both studies combined

The chair of the DSMB may be contacted for advice and independent review by the Investigator or trial Sponsor in the following situations:

• Following any SAE deemed to be possibly, probably or definitively related to a study intervention.

• Any other situation where the Investigator or trial Sponsor feels independent advice or review is important.

The DSMB will review SAEs deemed possibly, probably or definitively related to study interventions. The DSMB will be notified within 24 hours of the Investigators' being aware of their occurrence. The DSMB can recommend placing the study on hold if deemed necessary following a study intervention-related SAE.

The DSMB will only be able to judge the short-term safety of the ChAdOx1 nCoV-19 vaccine. Given the complexity of the underlying immunology and the minimal immunological data that will be available for review early in the study, the DSMB will not be in a position to comment on the effects of a later wave of SARS-CoV-2 as vaccine-induced immunity wanes and the theoretical risk of immune enhancement increases.

9.14 Safety Group Holding Rules

These safety holding rules apply to ChAdOx1 nCoV-19 vaccine only. Solicited, unsolicited and laboratories adverse events will be systematically collected in groups 1, 2, 5, 7 and 8. A sub-set of up to 1000 participants in each of Groups 4, 6, 9 and 10 will be asked to record solicited and unsolicited AEs for 7 days only.

- **Solicited local adverse events:**
  - If more than 25% of doses of the vaccine at a given time point (e.g. Day 0, Day 28) in a study group are followed by the same Grade 3 solicited local adverse event beginning within 2 days after vaccination (day of vaccination and one subsequent day) and persisting at Grade 3 for >72 hrs
- **Solicited systemic adverse events:**
  - If more than 25% of doses of the vaccine at a given time point (e.g. Day 0, Day 28) in a study group are followed by the same Grade 3 solicited systemic adverse event beginning within 2 days after vaccination (day of vaccination and one subsequent day) and persisting at Grade 3 for >72 hrs

- **Unsolicited adverse events:**
    - If more than 25% of doses of the vaccine at a given time point (e.g. Day 0, Day 28) in a study group are followed by the same Grade 3 unsolicited adverse event beginning within 2 days after vaccination (day of vaccination and one subsequent day) and persisting at Grade 3 for >72 hrs
- **Laboratory adverse event:**
    - If more than 25% of doses of the vaccine at a given time point (e.g. Day 0, Day 28) in a study group are followed by the same Grade 3 laboratory adverse event beginning within 3 days after vaccination and persisting at Grade 3 for >72 hrs
- **A serious adverse event considered possibly, probably or definitely related to vaccination occurs**
    - If an SAE occurs in any one individual, which is possibly, probably or definitely related to vaccination this would trigger a holding rule. There are two exemptions from this rule, which would not activate a holding rule. These include:
        - COVID-19 related hospital admissions considered to be at least possibly related to ChAdOx1 nCoV-19 (e.g. if considered to be a clinical presentation of a disease enhancement episode). COVID-19 related SAEs will be regularly reviewed by the DSMB, and a single event will not trigger a holding rule.
        - SAEs reported under the Hy's Law requirement will not necessarily trigger a holding rule. These cases will also be reviewed by the DSMB

If any of the above holding rules are activated, then further vaccinations in any of the groups will not occur until a safety review by the DSMB, study sponsor and the chief investigator has been conducted and it is deemed appropriate to restart dosing. The Regulatory Authority will be informed and a request to restart dosing with pertinent data will be submitted as a substantial amendment. The safety review will consider:

- The relationship of the AE or SAE to the vaccine.
- The relationship of the AE or SAE to the vaccine dose, or other possible causes of the event.

- If appropriate, additional screening or laboratory testing for other volunteers to identify those who may develop similar symptoms and alterations to the current Participant Information Sheet (PIS) are discussed.
- New, relevant safety information from ongoing research programs on the various components of the vaccine.

The local ethics committee and vaccine manufacturers will also be notified if a holding rule is activated or released.

All vaccinated volunteers will be followed for safety until resolution or stabilisation (if determined to be chronic sequelae) of their AEs.

Since the approval of ChAdOx1 nCoV-19 for emergency use by the MHRA and its subsequent national roll-out, group holding rules will no longer apply. Any SUSARs occurring in the trial from the time of approval onwards will be notified to and discussed with the DSMB and the MHRA, but will no longer automatically trigger a holding rule.

9.14.1 Individual stopping rules (will apply to prime-boost groups only)

In addition to the above stated group holding rules, stopping rules for individual volunteers will apply (i.e., indications to withdraw individuals from further vaccinations). Study participants who present with at least one of the following stopping rules will be withdrawn from further vaccination in the study:

- **Local reactions:** Injection site ulceration, abscess or necrosis

- **Laboratory AEs:**
  the volunteer develops a Grade 3 laboratory AE considered possibly, probably or definitely related within 7 days after vaccination and persisting continuously at Grade 3 for > 72hrs.

- **Systemic solicited adverse events:**
  - the volunteer develops a Grade 3 systemic solicited AE considered possibly, probably or definitely related within 2 days after vaccination (day of vaccination and one subsequent day) and persisting continuously at Grade 3 for > 72hrs.

- **Unsolicited adverse events:**

- the volunteer has a Grade 3 adverse event, considered possibly, probably or definitely related to vaccination, persisting continuously at Grade 3 for >72hrs.
- the volunteer has a SAE considered possibly, probably or definitely related to vaccination.
- the volunteer has an acute allergic reaction or anaphylactic shock following the administration of vaccine investigational product.

If a volunteer has an acute respiratory illness (moderate or severe illness with or without fever) or a fever (oral temperature greater than 37.8°C) at the scheduled time of administration of investigational product/control, the volunteer will not be enrolled and will be withdrawn from the study.

All vaccinated volunteers will be followed for safety until the end of their planned participation in the study or until resolution or stabilisation (if determined to be chronic sequelae) of their AEs, providing they consent to this.

Participants who met individual holding rules or were advised not to receive a booster dose as a result of an AE and have been offered a COVID-19 approved/licensed vaccine will be given an opportunity to discuss potential safety implications if they were to accept the offered vaccine with clinically qualified investigators.

In addition to these pre-defined criteria, the study can be put on hold upon advice of the DSMB, Chief Investigator, Study Sponsor, regulatory authority, Ethical Committee(s), for any single event or combination of multiple events which, in their professional opinion, jeopardise the safety of the volunteers or the reliability of the data.

10 STATISTICS

9.1 Description of Statistical Methods

Both a fully detailed study level statistical analysis plan (SAP) as well as a separate Statistical Analysis Plan for the Marketing Authorisation Application (MAA SAP) will be written and signed off before any interim data analyses are conducted.

The data from this study will be included in prospective pooled analyses of studies for efficacy and safety of ChAdOx1 nCoV-19 to provide greater precision of both efficacy and safety outcomes.

10.1.1 Efficacy Outcomes

The primary efficacy endpoint is PCR* positive symptomatic COVID-19.

This is defined as a participant with a PCR+* swab and at least one of the following symptoms: cough, fever ≥ 37.8, shortness of breath, anosmia, or ageusia.

Where possible, sensitivity analyses will be conducted using common alternative definitions of virologically-confirmed COVID-19 disease, including those in use in other phase 3 protocols (including but not limited to: USA AstraZeneca phase 3 trial, South Africa COV005 trial, WHO solidarity trial, CEPI definition). This will aid in comparisons between various studies and meta-analyses. These alternative definitions will be detailed in the statistical analysis plan as exploratory analyses.

*or other nucleic acid amplification test

9.2 Primary efficacy

The primary and secondary analyses will be conducted on participants who are seronegative at baseline. A sensitivity analysis will be conducted including all participants regardless of baseline serostatus.

Analysis of the primary endpoint will be computed as follows:

1. **Efficacy of two doses of vaccine** where the booster vaccine was a high-dose ChAdOx1 nCoV-19. Only participants who received two doses will be included (LD/SD or SD/SD) and only cases occurring more than 14 days after the second vaccine will be included.

Secondary analysis

2. **Efficacy of at least one standard-dose** of any ChAdOx1 nCoV-19. Cases occurring more than 21 days after the first vaccination will be included if the first vaccine was a high-dose vaccine. For participants who received a low-dose as their first vaccine, only cases occurring more than 14 days after a standard-dose booster will be included. Participants receiving only low-dose vaccines will be excluded.

3. **Efficacy of two standard-doses of vaccine**. Only participants who received two standard-dose vaccines will be included and only cases occurring more than 14 days after the second vaccine will be included.

Proportions will be compared between ChAdOx1 nCoV-19 and MenACWY groups using a Poisson regression model with robust variance [21]. The model will contain terms including treatment group, and age group at randomization if there is a sufficient sample size within each age category. The logarithm of the period at risk for primary endpoint will be used as an offset variable in the model to adjust for volunteers having different follow up times during which the events occur. Vaccine efficacy (VE) will be calculated as (1 – RR) x 100%, where RR is the relative risk of symptomatic infection (ChADOx1 nCoV-19: Control) and 95% confidence intervals will be presented.

If the Poisson regression model with robust variance fails to converge, the exact conditional method for stratified poisson regression will be used.

Cumulative incidence of symptomatic infections will be presented using the Kaplan-Meier method.

Secondary efficacy endpoints will be analysed in the same way as the primary efficacy endpoint.

Analyses will conducted for all adults combined as well as conducting analyses stratified by age cohorts.

All data from participants with PCR-positive swabs will be assessed for inclusion in the efficacy analyses by two blinded assessors who will independently review each case according to pre-specified criteria as detailed in the statistical analysis plan, to classify each for inclusion in the primary and secondary outcomes. A separate CRF will be designed for this purpose.

All PCR-positive results will be assessed for the primary outcome, including those with symptoms swabbed by trial staff, those with positive throat swabs from weekly home-testing, and other potential sources of information such as health-care workers who are tested at their workplace as either a routine test procedure or due to developing symptoms.

PCR+ swabs from outside the trial (for example, a workplace routine swab result in a healthcare worker) will be reviewed by blinded staff and only included as a potential endpoint if the test was conducted in 1) a medical laboratory with ISO 15189 accreditation (provided by UKAS in UK) AND 2) an assay that is either CE marked or that has a derogation authorisation from the MHRA.

*or other nucleic acid amplification test

9.3 Safety & Reactogenicity

Counts and percentages of each local and systemic solicited adverse reaction from diary cards, and all unsolicited AEs and SAEs will be presented for each group.

9.4 Immunogenicity

Highly skewed antibody data will be log-transformed prior to analysis. The geometric mean concentration and associated 95% confidence interval will be summarised for each group at each timepoint, by computing the anti-log of the mean difference of the log-transformed data.

The geometric mean concentration at day 28 and the proportion of participants seroconverting to the S-spike protein from day 0 to day 28 will be computed. Comparisons between ChAdOx1 nCoV-19 vaccine and MenACWY groups will be made using a Mann Whitney U test due to the low titres expected in the control group which will cause a non-normal distribution.

In addition, those aged 56 years or older receiving either a single-dose or two-doses of ChAdOx1 nCoV-19 vaccine will be compared with those in phase 1 (COV001) aged 18-55 years who received single-dose ChAdOx1 nCoV-19.

Spike-specific T cell responses (ELISpot) will be presented as means and confidence intervals, or medians and interquartile ranges if non-normally distributed at all post vaccination time points. Comparisons between ChAdOx1 nCoV-19 vaccine and MenACWY groups will be made using a Mann Whitney U test due to the low responses expected in the control group which will cause a non-normal distribution. Comparison between two different dose levels of ChAdOx1 nCoV-19 will be made using t-tests. In addition, those aged 56 years or older receiving either a single-dose or two-doses of ChAdOx1 nCoV-19 vaccine will be compared with those in phase 1 (COV001) aged 18-55 years who received single-dose ChAdOx1 nCoV-19.

9.5 Subgroup analyses

Subgroup comparisons of efficacy, and safety will be conducted by incorporating vaccine-group by subgroup interaction terms into appropriate regression models. Subgroup comparisons will only be conducted if there are at least 5 cases in all subgroups.

Comparisons will include:

1. Males vs females

2. Age (18 to 55 years vs 56-<70 years vs 70+ years)

3. Seropositive to S-spike or non-spike proteins at baseline vs not seropositive

4. Health care workers and highly-exposed participants versus others

5. Standard dose versus low dose

9.6 Interim and primary analyses of the primary outcome

It is planned that the primary evidence of efficacy and safety for the ChADOx1 nCoV-19 vaccine will be based on global analyses utilizing studies COV001 (the UK P1/2 study), COV002 (the UK P2/3 study), COV003 (the Brazil P3 study) and COV005 (the South Africa P1/2 study) including a pooled analysis across the studies. As such the interim and primary analyses for the primary outcome will be based on cases accumulated across multiple studies, details of which will be specified within the MAA SAP rather than for each individual study. Interim and primary data cuts from this study will therefore be carried out to support the pooled analysis.

The global MAA SAP allows for interim and primary analyses to be conducted once sufficient eligible cases have accumulated, where the overall type 1 error is controlled at the 5% level using a flexible alpha–spending approach that accounts for the incorporation of data from this study into pooled interim analyses under the global MAA SAP.

Evidence of efficacy will be determined if the lower bound of the multiplicity adjusted confidence interval is greater than a 20% threshold. The primary analysis will have approximately 90% power assuming a vaccine efficacy of 60%. A flexible alpha spending approach will be implemented to allow an earlier primary analysis in the situation where accumulation of eligible cases were lower than expected.

Evidence of efficacy at an interim or primary analysis of pooled data will not be considered a reason to stop the trial, but instead will be interpreted as early evidence of efficacy. However if an interim analysis

demonstrates evidence of efficacy then a study level analysis according to the study SAP may be used to support study level evidence of efficacy.

Participants who received other vaccines during the trial will have their vaccination data recorded. If a participant receives an approved or licensed COVID-19 vaccine outside of the trial procedures (such as through their workplace) this information will be recorded. Participants will be censored in the analysis at the time of their vaccination with the alternative COVID-19 vaccine.
If a large proportion of trial volunteers are vaccinated in this way then an exploratory, non-randomised analysis of efficacy according to vaccine(s) received will be undertaken if data are sufficient.

9.7 Final Analysis

A final analysis will be conducted at the end of the study. The final study-specific analysis will incorporate all data from the study, including data that has previously contributed to global efficacy estimates under the pooled analysis strategy. The final analysis will be considered a supportive analysis to the global efficacy analysis. Alpha at the final study-specific analysis will be adjusted to incorporate the number of previous global analyses to which the study contributed data in order to control the overall study level type 1 error at 5%. Details will be specified in the study level SAP.

9.8 Procedure for Accounting for Missing, Unused, and Spurious Data.

All available data will be included in the analysis

9.9 Inclusion in Analysis

All vaccinated participants will be included in the analysis unless otherwise specified in the SAP.

9.10 Interim analysis for the combined DSMB

The independent DSMB will meet regularly to review safety data from all available studies of ChAdOx1 nCoV-19 and will assess whether the assumptions underlying the sample size calculation

are in line with the observed cases. Additionally the independent DSMB will make recommendations based on the interim analyses to assess evidence of efficacy.

11 DATA MANAGEMENT

11.1 Data Handling

The Chief Investigator will be responsible for all data that accrues from the study.

All study data including participant diary will be recorded directly into an Electronic Data Capture (EDC) system (e.g. OpenClinica, REDCap, or similar) or onto a paper source document for later entry into EDC if direct entry is not available. This includes safety data, laboratory data and outcome data. Any additional information that needs recording but is not relevant for the CRF (such as signed consent forms etc.) will be recorded on a separate paper source document. All documents will be stored safely and securely in confidential conditions.

All adverse event data (both solicited and unsolicited) reported by the volunteer will be entered onto a volunteer's electronic diary card (eDiary) for a maximum of 28 days following administration of the IMP. The eDiary provides a full audit trial of edits and will be reviewed at time-points as indicated in the schedule of events. Any adverse event continuing beyond the period of the diary will be copied into the eCRF and followed to resolution, if there is a causal relationship to the IMP, or to the end of the study if there is no causal relationship.

The participants will be identified by a unique trial specific number and code in any database. The name and any other identifying detail will NOT be included in any trial data electronic file, with the exception of the electronic diaries and household questionnaire, for which consent will be obtained to store the participant email address for quality control purposes. Only site research staff and sponsor data managers have access to view the email address.

The EDC system (CRF data) uses a relational database (MySQL/ PostgreSQL) via a secure web interface with data checks applied during data entry to ensure data quality. The database includes a complete suite of features which are compliant with GCP, EU and UK regulations and Sponsor security policies, including a full audit trail, user-based privileges, and integration with the institutional LDAP server. The MySQL and PostgreSQL database and the webserver will both be housed on secure servers maintained by the University of Oxford IT personnel. The servers are in a physically secure location in Europe. Backups will be stored in accordance with the IT department schedule of daily, weekly, and monthly retained for one month, three months, and six months, respectively. The IT servers provide a stable, secure, well-maintained, and high capacity data storage environment. REDCap and

OpenClinica are widely-used, powerful, reliable, well-supported systems. Access to the study's database will be restricted to the members of the study team by username and password.

If participants consent to provide stool samples; the stool sample (in an anonymised form) will be collected from them by a courier and processed in a laboratory by International Health Management Associates (IHMA), an accredited central laboratory. The sample will then be shipped for analysis by Astra Zeneca in a laboratory in the US. The participant would need to provide their name and address to the courier company.

11.2 Record Keeping

The Investigators will maintain appropriate medical and research records for this trial, in compliance with GCP and regulatory and institutional requirements for the protection of confidentiality of volunteers. The Chief Investigator, co-Investigators and clinical research nurses will have access to records. The Investigators will permit authorised representatives of the Sponsor(s), as well as ethical and regulatory agencies to examine (and when required by applicable law, to copy) clinical records for the purposes of quality assurance reviews, audits and evaluation of the study safety and progress.

Identifiable information such as contact details will be stored for a minimum of 5 years and until the youngest participant turns 21 years. De-identified research data maybe be stored indefinitely. If volunteers consent to be contacted for future research, information about their consent form will be recorded, retained and stored securely and separately from the research data. If volunteers consent to have their samples stored and used in future research, information about their consent form will be recorded, retained and stored securely as per Biobanking procedures and SOP.

11.3 Source Data and Case Report Forms (CRFs)

All protocol-required information will be collected in CRFs designed by the Investigator. All source documents will be filed in the CRF. Source documents are original documents, data, and records from which the volunteer's CRF data are obtained. For this study, these will include, but are not limited to, volunteer consent form, blood results, GP response letters, laboratory records, diaries, medical records and correspondence. In the majority of cases, CRF entries will be considered source data as the CRF is the site of the original recording (i.e. there is no other written or electronic record of data).

In this study this will include, but is not limited to medical history, medication records, vital signs, physical examination records, urine assessments, blood results, adverse event data and details of vaccinations. All source data and volunteer CRFs will be stored securely.

To prevent withdrawal of a participant due to relocation, if there is a nearby participating site and with the consent of the participant, copies of relevant participant research records (such as ICF, paper source documents) will be transferred to the local site using secure email addresses such as nhs.net or by password protected sheets. The electronic research data stored on REDCap will also be transferred to the new site. The original records will be retained by the recruiting site.

11.4 Data Protection

The study protocol, documentation, data and all other information generated will be held in strict confidence. No information concerning the study or the data will be released to any unauthorised third party, without prior written approval of the sponsor.

11.5 Data Quality

Data collection tools will undergo appropriate validation to ensure that data are collected accurately and completely. Datasets provided for analysis will be subject to quality control processes to ensure analysed data is a true reflection of the source data.

Trial data will be managed in compliance with local data management SOPs. If additional, study specific processes are required, an approved Data Management Plan will be implemented

11.6 Archiving

Study data may be stored electronically on a secure server, and paper notes will be kept in a key-locked filing cabinet at the site. All essential documents will be retained for a minimum of 5 years after the study has finished. The need to store study data for longer in relation to licensing of the vaccine will be subject to ongoing review. For effective vaccines that may be licensed, we may store research data securely at the site at least 15 years after the end of the study, subject to adjustments

in clinical trials regulations. Where relevant participants' bank details will be stored for 7 years in line with the site financial policy. De-identified research data maybe be stored indefinitely

General archiving procedures will be conducted in compliance to SOP OVC020 Archiving.

Individual level COV002 data may be shared with COV009, a follow on long-term safety and immunogenicity study, providing participants have consented to this within the COV009 study consent.

12 QUALITY CONTROL AND QUALITY ASSURANCE PROCEDURES

12.1 Investigator procedures

Approved site-specific standard operating procedures (SOPs) will be used at all clinical and laboratory sites.

12.2 Trial Steering Committee

A Trial Steering Committee will be appointed and will consist of an independent Chairman, not less than two other independent members and the Chief Investigator. All significant operational matters relating to the research will be decided upon by the trial steering committee that would have as main objectives:

- provide advice, through its chair, to the investigators, the trial sponsor, the collaborators on all appropriate aspects of the trial
- concentrate on progress of the trial, adherence to the protocol, patient safety and the consideration of new information of relevance to the research question
- agree proposals for substantial protocol amendments and provide advice to the sponsor and funder regarding approvals of such amendments

The trial steering committee will meet regularly and as required.

12.3 Monitoring

Regular monitoring will be performed according to GCP by the monitor. Following written SOPs, the monitor will verify that the clinical trial is conducted and data are generated, documented and

reported in compliance with the protocol, GCP and the applicable regulatory requirements. The site will provide direct access to all trial related source data/documents and reports for the purpose of monitoring and auditing by the Sponsor and inspection by local and regulatory authorities.

12.4 Protocol deviation

Any deviations from the protocol will be documented in a protocol deviation form and filed in the trial master file. Each deviation will be assessed as to its impact on volunteer safety and study conduct. Significant protocol deviations will be listed in the end of study report.

12.5 Audit & inspection

The QA manager conducts systems based internal audits to check that trials are being conducted according to local procedures and in compliance with study protocols, departmental SOPs, GCP and applicable regulations.

The Sponsor, trial sites, and ethical committee(s) may carry out audit to ensure compliance with the protocol, GCP and appropriate regulations.

GCP inspections may also be undertaken by the MHRA to ensure compliance with protocol and the Medicines for Human Use (Clinical Trials) Regulations 2004, as amended. The Sponsor will assist in any inspections and will support the response to the MHRA as part of the inspection procedure.

13 SERIOUS BREACHES

The Medicines for Human Use (Clinical Trials) Regulations contain a requirement for the notification of "serious breaches" to the MHRA within 7 days of the Sponsor becoming aware of the breach.

A serious breach is defined as “A breach of GCP or the trial protocol which is likely to effect to a significant degree

(a) the safety or physical or mental integrity of the subjects of the trial; or

(b) the scientific value of the trial”.

In the event that a potential serious breach is suspected the Sponsor will be informed as soon as possible, to allow preliminary assessment of the breach and reporting to the MHRA within the required timelines.

14 ETHICS AND REGULATORY CONSIDERATIONS

14.1 Declaration of Helsinki

The Investigators will ensure that this study is conducted according to the principles of the current revision of the Declaration of Helsinki.

14.2 Guidelines for Good Clinical Practice

The Investigator will ensure that this trial is conducted in accordance with relevant regulations and with Good Clinical Practice.

14.3 Ethical and Regulatory Approvals

Following Sponsor approval the protocol, informed consent form, participant information sheet and any proposed advertising material will be submitted to an appropriate Research Ethics Committee (REC), HRA (where required), regulatory authorities (MHRA in the UK), and host institution(s) for written approval. No amendments to this protocol will be made without consultation with, and agreement of, the Sponsor.

The Investigator is responsible for ensuring that changes to an approved trial, during the period for which regulatory and ethical committee(s) approval has already been given, are not initiated without regulatory and ethical committee(s)' review and approval except to eliminate apparent immediate hazards to the subject (i.e. as an Urgent Safety Measure).

14.4 Volunteer Confidentiality

The study will comply with the General Data Protection Regulation (GDPR) and Data Protection Act 2018, which require data to be de-identified as soon as it is practical to do so. The processing of personal data of participants will be minimised by making use of a unique participant study number only on all study documents and any electronic database(s), with the exception of informed consent forms, participant ID logs, electronic diaries and the Household Questionnaire. All documents will be stored securely and only accessible by study staff and authorised personnel. The study staff will safeguard the privacy of participants' personal data. A separate confidential file containing identifiable information will be stored in a secured location in accordance with the current data

protection legislation. Photographs taken of vaccination sites (if required, with the volunteer's written, informed consent) will not include the volunteer's face and will be identified by the date, trial code and subject's unique identifier. Once developed, photographs will be stored as confidential records, as above. This material may be shown to other professional staff, used for educational purposes, or included in a scientific publication.

If participants have a positive swab result for COVID-19 during the course of the study then the Public Health Authority will be notified as COVID-19 is a “notifiable disease” and this is a legal requirement in the UK. This may mean participants personal information from their health records will be shared with Public Health either by the processing lab or the study site. Participants may also be contacted by the NHS Test and Trace service. Samples collected using home swab kits may be processed at laboratories within and outside the UK, as determined by the community testing programme. These laboratories provide a test result for the barcode to NPEx (National Pathology Exchange) and this result is then recombined with participant identifiable information by NHS Digital. NHS Digital provide lab results to the Sponsor (University of Oxford) who will match this with personal data including identifying contact information sent to them by the site in order to centralise the processing of weekly surveillance results. Participants will be required to separately consent to the terms and conditions of the national community swabbing programme, each time they perform a self-swab. This is available at: https://www.gov.uk/government/publications/coronavirus-covid-19-testing-privacy-information/testing-for-coronavirus-privacy-information

15 FINANCING AND INSURANCE

15.1 Financing

The study is funded by the UK Government through the National Institute for Health Research (NIHR). AstraZeneca have provided funding for some exploratory objectives.

15.2 Insurance

The University has a specialist insurance policy in place which would operate in the event of any participant suffering harm as a result of their involvement in the research (Newline Underwriting Management Ltd, at Lloyd's of London). NHS indemnity operates in respect of the clinical treatment which is provided.

15.3 Compensation

Volunteers in groups 1, 2, 5, 7, 8 and 12 will be compensated for their time, the inconvenience of having blood tests and procedures, and their travel expenses. The total amount compensated will be approximately £390-555 depending on the exact number of visits, and whether any repeat or additional visits are necessary (including symptomatic pathway visits). Participants screening for groups 1, 2, 5, 7, 8 and 12 who do not fully enrol will be compensated £25 for attending the screening visit. For all other trial visits as outlined in Tables 5-14, compensation will be calculated according to the following:

- Travel expenses: £15 per visit
- Inconvenience of blood tests: £10 per blood donation
- Time required for visit: £20 per hour

To clarify, this would equate to £75 for a vaccination visit and £45 for a follow up visit.

Should a volunteer from any group decide to withdraw from the trial before it is completed, payment will be pro rata.

Participants in Groups 4, 6, 9, 10 and 11 will not be compensated.

15.4 Publication Policy

The Investigators will be involved in reviewing drafts of the manuscripts, abstracts, press releases and any other publications arising from the study. Data from the study may also be used as part of a thesis for a PhD or MD.

16 DEVELOPMENT OF A NEW PRODUCT/ PROCESS OR THE GENERATION OF INTELLECTUAL PROPERTY

Ownership of IP generated by employees of the University rests in the University. The protection and exploitation of any new IP is managed by the University's technology transfer office, Oxford University Innovations. Investigators in this study may benefit from the royalty sharing policy of the University if new intellectual property is generated from the trial. Several investigators are applicants

or co-inventors on previous patent filings or patents related to ChAdOx1 vaccines. The University of Oxford, which is partnered with the Oxford University Hospitals NHS Foundation Trust in the NIHR Oxford Biomedical Research Centre, is committed to the translational progress and commercial development of healthcare products potentially meeting medical and global health needs, and does and will work with commercial partners towards these goals.

References

1 Zhu, N. *et al.* A Novel Coronavirus from Patients with Pneumonia in China, 2019. *New England Journal of Medicine* **382**, 727-733, doi:10.1056/NEJMoa2001017 (2020).

2 (!!! INVALID CITATION !!! 2).

3 Li, F. Structure, Function, and Evolution of Coronavirus Spike Proteins. *Annual review of virology* **3**, 237-261, doi:10.1146/annurev-virology-110615-042301 (2016).

4 Zhou, P. *et al.* A pneumonia outbreak associated with a new coronavirus of probable bat origin. *Nature*, doi:10.1038/s41586-020-2012-7 (2020).

5 Alharbi, N. K. *et al.* ChAdOx1 and MVA based vaccine candidates against MERS-CoV elicit neutralising antibodies and cellular immune responses in mice. *Vaccine* **35**, 3780-3788, doi:10.1016/j.vaccine.2017.05.032 (2017).

6 Tseng, C. T. *et al.* Immunization with SARS coronavirus vaccines leads to pulmonary immunopathology on challenge with the SARS virus. *PLoS One* **7**, e35421, doi:10.1371/journal.pone.0035421 (2012).

7 Weingartl, H. *et al.* Immunization with modified vaccinia virus Ankara-based recombinant vaccine against severe acute respiratory syndrome is associated with enhanced hepatitis in ferrets. *J Virol* **78**, 12672-12676, doi:10.1128/JVI.78.22.12672-12676.2004 (2004).

8 Liu, L. *et al.* Anti-spike IgG causes severe acute lung injury by skewing macrophage responses during acute SARS-CoV infection. *JCI insight* **4**, e123158, doi:10.1172/jci.insight.123158 (2019).

9 Agrawal, A. S. *et al.* Immunization with inactivated Middle East Respiratory Syndrome coronavirus vaccine leads to lung immunopathology on challenge with live virus. *Hum Vaccin Immunother* **12**, 2351-2356, doi:10.1080/21645515.2016.1177688 (2016).

10 Munster, V. J. *et al.* Protective efficacy of a novel simian adenovirus vaccine against lethal MERS-CoV challenge in a transgenic human DPP4 mouse model. *NPJ vaccines* **2**, 28, doi:10.1038/s41541-017-0029-1 (2017).

11 Alharbi, N. K. *et al.* Humoral Immunogenicity and Efficacy of a Single Dose of ChAdOx1 MERS Vaccine Candidate in Dromedary Camels. *Scientific reports* **9**, 16292, doi:10.1038/s41598-019-52730-4 (2019).

12 Gao, Q. *et al.* Development of an inactivated vaccine candidate for SARS-CoV-2. *Science*, eabc1932, doi:10.1126/science.abc1932 (2020).

13 Antrobus, R. D. *et al.* Clinical assessment of a novel recombinant simian adenovirus ChAdOx1 as a vectored vaccine expressing conserved Influenza A antigens. *Molecular therapy : the journal of the American Society of Gene Therapy* **22**, 668-674, doi:10.1038/mt.2013.284 (2014).

14 Coughlan, L. *et al.* Heterologous Two-Dose Vaccination with Simian Adenovirus and Poxvirus Vectors Elicits Long-Lasting Cellular Immunity to Influenza Virus A in Healthy Adults. *EBioMedicine*, doi:https://doi.org/10.1016/j.ebiom.2018.02.011 (2018).

15 Wilkie, M. *et al.* A phase I trial evaluating the safety and immunogenicity of a candidate tuberculosis vaccination regimen, ChAdOx1 85A prime - MVA85A boost in healthy UK adults. *Vaccine* **38**, 779-789, doi:10.1016/j.vaccine.2019.10.102 (2020).

16 Folegatti, P. M. *et al.* Safety and immunogenicity of a candidate Middle East respiratory syndrome coronavirus viral-vectored vaccine: a dose-escalation, open-label, non-randomised, uncontrolled, phase 1 trial. *The Lancet Infectious Diseases*, doi:https://doi.org/10.1016/S1473-3099(20)30160-2 (2020).

17 Modjarrad, K. MERS-CoV vaccine candidates in development: The current landscape. *Vaccine* **34**, 2982-2987, doi:10.1016/j.vaccine.2016.03.104 (2016).

18 Parmigiani, A. *et al.* Impaired antibody response to influenza vaccine in HIV-infected and uninfected aging women is associated with immune activation and inflammation. *PLoS One* **8**, e79816, doi:10.1371/journal.pone.0079816 (2013).

19 Shive, C. L. *et al.* Pre-vaccine plasma levels of soluble inflammatory indices negatively predict responses to HAV, HBV, and tetanus vaccines in HCV and HIV infection. *Vaccine* **36**, 453-460, doi:10.1016/j.vaccine.2017.12.018 (2018).

20 JCVI. Priority groups for coronavirus (COVID-19) vaccination: advice from the JCVI (30 December 2020).

21 Zou, G. A modified poisson regression approach to prospective studies with binary data. *Am J Epidemiol* **159**, 702-706, doi:10.1093/aje/kwh090 (2004).

APPENDIX A: AMENDMENT HISTORY

Amendment No.	Protocol Version No.	Date issued	Author(s) of changes	Details of Changes made
N/A	1.0	03 APR 2020	Pedro Folegatti, Andrew Pollard, Merryn Voysey, Sarah Gilbert	N/A
1	2.0	14 APR 2020	Emma Plested	Addition of North Bristol NHS Trust as a site.
N/A	3.0	30 APR 2020	Pedro Folegatti	Added rationale for recruiting paediatric groups; Added stopping/holding rules to groups 1, 2 and 3; Specified minimum safety and immunogenicity data required from COV001 prior to start of COV002; Specified minimum safety data required prior to enrolment into older participants in group 4 and enrolment of children into group 3; Added staggered enrolment with interim reviews for groups 1, 2 and 3.
2	4.0	14 May 2020	Pedro Folegatti	Added Dr Angela Minassian as an Investigator; 1 year follow-up as standard trial procedures; added group 5 for batch safety and immunogenicity comparison with COV001; increased sample size to up to 10,260 and adjusted statistical analysis section to reflect this; changes and clarifications to exclusion criteria; added priority groups for recruitment; added anosmia/ageusia as part of the trigger for swabbing criteria; added efficacy against infection as tertiary/exploratory

Amendment No.	Protocol Version No.	Date issued	Author(s) of changes	Details of Changes made
				endpoint; weekly PCR samples subject to test availability and site capacity; HCW exemption from filling-out weekly COVID-19 exposure diaries; adjusted blood volumes; added a section on changes to group numbers in the event of further different batches being required in order to complete dosing; clarifications to storage conditions of the IMP; harmonised AESI section with COV001 and as per Brighton Collaboration suggestions; introduced prophylactic paracetamol in group 4; Changes to funding arrangements; added multiple sites.
3	5.0	26 May 2020	Merryn Voysey, Pedro Folegatti, Maheshi Ramasamy	Addition of age stratification in randomisation to group 4 (<55years and ≥56years); Corrections to site addresses; updated information on pre-clinical data and disease enhancement/immunopathology; expanded on rationale for recruiting children; addition of exploratory endpoint for batch comparison between COV001 and COV002; Groups 1 and 2 to be recruited sequentially instead of in parallel following request from the DSMB with a minimum of 7 days interval; updated section on potential risks to volunteers following preliminary pre-clinical and clinical data on ChAdOx1 nCoV-19; removed potential benefit from taking part in the study as participants in

Amendment No.	Protocol Version No.	Date issued	Author(s) of changes	Details of Changes made
				group 4 won't necessarily undergo physical examination; clarification to recruitment strategy on priority groups; clarification to additional exclusion criteria in group 4 where caution to be taken when advising Paracetamol to participants on chronic use; Added PAXgene sample to last follow-up visit in participants who had a positive COVID-19 PCR sample at diagnosis visit; removed baseline PCR swab; additional text to encourage participants to contact study team for any medical attended event; clarification to weekly swab procedures; Swab testing to be undertaken by DHSC and data from this and any COVID-19 testing will be shared with lead site for central analysis; Nasopharyngeal swab to be conducted at 7 days post COVID-19 diagnosis visit only if considered necessary or if first sample is negative; added Kawasaki-like disease and other hyperinflammatory syndromes as AESI in the paediatric group; clarification to holding rule procedures, so it applies to all groups if a holding rule is met; clarifications to statistical analysis section; added information on volunteer confidentiality regarding weekly swabs data processing; clarification that participants in Group 4 will not be compensated; correction of formatting and typographical errors throughout the document; added information blinding

Amendment No.	Protocol Version No.	Date issued	Author(s) of changes	Details of Changes made
				for efficacy endpoints; clarification of the groups required to report AEs; Option for participants to transfer to existing sites if relocating
4	6.0	05-JUN-2020	Pedro Folegatti	Reduced number of participants recruited into group 4; Added group 6 for comparison between dosing on Abs260 and qPCR methods.
5	7.0	18 Jun 2020	Pedro Folegatti, Merryn Voysey, Hannah Robinson	Addition of groups 5 A, B/C, 7 A and B & 8 A and B (reactogeniticy and immunogenicity comparison between different doses given with different methods for measuring doseS); Increase in sample size to up to 10,560;Group 4b has been added to provide immunogenicity data on homologous prime-boost at 5x10^{10}vp (Abs260) prime and 2.2x1010vp (qPCR) boost, where up to 100 volunteers aged 18-55 initially recruited into group 4a will receive a booster dose of the vaccine 4-6 weeks apart<br><br>Addition of process should a participant who wishes to continue in the trial, relocate and to an area with a study site; Inclusion of a mucosal immunity swabs in a subset of participants<br><br>Addition of optional stool samples; Clarification to AE grading table where not all A&E assessments should be

Amendment No.	Protocol Version No.	Date issued	Author(s) of changes	Details of Changes made
				recorded as SAEs; clarification on which PCR positive tests conducted outside the study procedures would be acceptable and included in primary endpoint analysis.<br>Change of PI at Cambridge site.
6	8.0	22 Jun 2020	Pedro Folegatti	Added day 42 visit in group 4b and increased the volume of serum taken. Clarification that the mucosal immunity assessments is to be done in a subset of group 6 individuals only.
9	9.0	20 Jul 2020	Pedro Folegatti, Merryn Voysey	Increased overall sample size; Added groups 5d (batch comparison group on Cobra material) and 9 and 10 (efficacy groups in participants aged 56 and above); Addition of diary completion for 7 days for groups 9 and 10 and 28 days for group 11; Groups 9 and 10 will be recommended to take paracetamol post vaccination; Removed participants aged 56 and above from groups 4 and 6; Added booster doses to groups 4 and 6; updated study endpoints to reflect comparisons of 1 vs 2 doses (groups 1, 2, 7 and 8); added information on Cobra material and product comparability and administration; updated primary efficacy analysis to reflect changes above; expanded the number of volunteers filling out diaries.; clarifications to holding/stopping rules; added Hy's law

Amendment No.	Protocol Version No.	Date issued	Author(s) of changes	Details of Changes made
				cases to be reported as SAEs; removed the requirement for SARS-CoV-2 serology prior to enrolment in groups 5d, 9 , 10 and 11.; added group 11 to recruit participants who previously received a ChAdOx1 vectored vaccine,; Paola Cicconi added as Investigator
Minor Amendment	9.1	31 Jul 2020	Pedro Folegatti,	Correction of errors with blood volumes for participants in groups 9, 10 and 11; Clarification of dose batches; clarification that the physical examination for group 5d is only if required.
10	10.0	06 Aug 2020	Pedro Folegatti, Merryn Voysey, Emma Plested, Hannah Robinson, Maheshi Ramasamy	Inclusion of D14 visit for group 11, changed swabbing pathway (S7 to be conducted only on positive cases, added S3-5 visit for second swab or home testing); an update to the 'Planned receipt of any vaccine other than the study intervention within 30 days before and after each study vaccination' exclusion criteria to allow an exception for the seasonal flu vaccine; clarifications made to visit time points; Re-consent may be collected with electronic signatures if required for infection control purposes; Confirmation that 15 minutes is a minimum time for post vaccine observations; clarification on the collection of AEs. ; addition of word 'boost' in group description; Clarification of the criteria for exclusion or delay of booster vaccination; Correction of length

Amendment No.	Protocol Version No.	Date issued	Author(s) of changes	Details of Changes made
				of study missed in previous amendment; Clarification of the process for information sharing following diagnosis with COVID-19; additional exclusion criteria for boosting doses to include AEs post prime that may affect the safety of the participant or the interpretation of the study results and SARS-CoV-2 PCR positivity within 4 weeks if symptomatic, or 2 weeks if asymptomatic; clarification to length of follow-up (i.e. 12 months from last vaccination); clarification to the process for sharing of information with public health authorities on COVID-19 positive cases; changes to the analysis procedures on the primary endpoint to reflect the new 2 dose schedule proposed
11	11.0	15 SEP 2020	Pedro Folegatti, Merryn Voysey, Andrew Pollard, Julie Fox	Clarifications to which groups are entitled to receive financial compensation; Clarification to efficacy objectives to include efficacy against severe disease; Clarification to exclusion criteria where only licensed seasonal influenza vaccines will be allowed within 30 days of vaccine administration, inclusion of HIV volunteers into sub-study, clarification to inclusion of participants with previous laboratory confirmed SARS-CoV-2 infection ; Clarifications to the statistical analysis section on primary, secondary and exploratory analysis; Clarifications to symptomatic pathway; Addition of boosting doses to groups 1a, 2a and 5a;

Amendment No.	Protocol Version No.	Date issued	Author(s) of changes	Details of Changes made
				Addition of HIV cohort sub-study; Correction of formatting and typographical errors; Changes to re-consent process to allow re-consent over the phone and electronic signatures when is not possible to have a physical visit;<br><br>.Updated exploratory immunology assays to reflect inclusion of HIV group (Group 12); Increase in time to vaccine administration from 4 to 6 hours; clarification to window for booster vaccinations.
12	12.0	20 – OCT -2020	K Emary	Clarification that home swabs may be processed outside UK; Clarification of the flow of information from home swabbing results to sponsor Clarification that stool may be collected at approximately 7 days post PCR positive result in those who are asymptomatic; Inclusion of CD4 count for screening of group 12; HIV serology is at investigators discretion for group 12; AstraZeneca have provided funding for some exploratory objectives; Clarification to exclusion criteria where licensed pneumococcal vaccines will be allowed within 7 days of study vaccine administration; increase in G12 sample volume to allow sufficient volume for exploratory objectives.

Amendment No.	Protocol Version No.	Date issued	Author(s) of changes	Details of Changes made
13 (SA16)	V13.0	03 NOV 2020	Merryn Voysey<br>Pedro Folegatti	Updated statistical analysis section; statistician signature space;<br>Number of participants recruited in groups 9 and 10 will be 1000 +/- 10% each, to account for the multiple site recruitment activity and recognizing the potential for over recruitment. The overall sample size is unchanged.
14 (SA17)	V14.0	13 NOV 2020	Maheshi Ramasamy, Emma Plested	Updated diagnostic PCR to NAAT (nucleic acid amplification assay) for purposes of endpoint definition; The number of diaries for Group 9 and 10 have been listed as approximately 500 in each group given the concurrent recruitment across multiple sites.
15 (SA18)	V15.0	03 DEC 2020	Pedro Folegatti<br>Maheshi Ramasamy<br>Hannah Robinson<br>Merryn Voysey	Removal of Group 3 - separate study protocol to assess safety and immunogenicity of ChAdOx1 nCoV-19 in paediatric age groups.<br>Addition of groups 5e and 5f– batch comparison with material manufactured by Serum Institute of India at different dose regimens.<br>Addition of >Grade 2 neurological events as AESI.

Amendment No.	Protocol Version No.	Date issued	Author(s) of changes	Details of Changes made
				Amended bleeding volumes to all groups<br>Clarification to compensation section<br>Volunteers previously having bloods taken for screening for COV002 will not have bloods repeated at screening if within 6 months unless indicated by medical history, safety bloods will be repeated pre-vaccination<br>Collection of safety information by phone if a participant is unable to attend a scheduled visit, allowing AE data to be collected but safety bloods cannot be collected or assessed. Safety oversight continues
SA20	V16.0	08 – DEC - 2020	Hannah Robinson, Emma Plested, Pedro Folegatti	Addition of plan and management for unblinding of participants to allow administration of a SARS-CoV-2 vaccine according to government guidance.<br>Those who received an approved or licensed COVID-19 will not be withdrawn from the study and will be followed-up as per their schedule of attendances. Exploratory, non-randomised analysis of efficacy according to vaccine(s) received will be undertaken if data are sufficient on other COVID-19 vaccines.<br>Extra visits A & B included for administration of additional study vaccine or collection of samples following early unblinding.

Amendment No.	Protocol Version No.	Date issued	Author(s) of changes	Details of Changes made
				Added the 'extra visit A' and extra visit B in the schedule of events<br>Safety section updated to consider assessment of causality of deployed vaccines.
SA22	V17.0	20-JAN-2021	Hannah Robinson, Pedro Folegatti	Only AESI and SAEs will be collected following Extra Visit B and vaccination as part of the NHS roll out<br>Clarification that visits can be conducted over the phone for pregnant participants and those unable to attend in person<br>Outline discontinuation processes that prevent withdrawal<br>Change in IMP storage instructions to use within 48 hours of vial piercing<br>Holding rules will no longer apply following approval for emergency use by the UK regulator and subsequent national roll-out.<br>Minor clarification to groups where diaries are collected; groups 6, 9 and 10
SA23	V18.0	04-MAR-2021	Daniel Jenkin	Changes to symptomatic participant follow up (to reduce study procedure burden on participants and simplify safety data collection):<br>If negative NAAT test outside of the trial during a symptomatic episode then no need to bring participant in for repeat swab

Amendment No.	Protocol Version No.	Date issued	Author(s) of changes	Details of Changes made
				If positive NAAT test outside + symptoms then still bring in for repeat swab and immunology bloods (no safety bloods, observations or examination needed)<br><br>No repeat home swab (A single negative NAAT test in or out of the trial is now sufficient to declare it a negative case)<br><br>Replace S7 physical visit with phone call<br><br>Removal of symptomatic ediary<br><br>Removal of S3-5 repeat swab/visit<br><br>Miscellaneous<br><br>Blood donation now permitted 3 months following receipt of final planned study vaccine<br><br>Update to extra visit B vaccination for pregnancy: Vaccination now permissible at extra visit B if participant has discussed with her usual clinician (e.g. GP) and chosen to receive a covid-19 vaccination<br><br>Clarification that a letter will be sent to the participant and their GP detailing the vaccinations received in the study, once participant has been unblinded.
SA24	V19.0	12-Apr-2021	Daniel Jenkin	Unblinding and provision of ChAdOx1 nCoV-19 to participants aged 30 years and above<br><br>Removal of 1 hour post prime and 15 minute post boost observation period

Amendment No.	Protocol Version No.	Date issued	Author(s) of changes	Details of Changes made
				Contraception advice following vaccination now harmonised with UK Greenbook guidance<br>Immunological correlate of protection endpoint added<br>Unblinded exploratory COVID-19 disease endpoints may be determined by trained trial team members (rather than blinded independent endpoint assessors)<br>The final trial visit paxgene bloods will now be taken on participant that attended for any CT visit(s) during the trial (regardless of whether the COVID-19 swab at the visit was positive or not)<br>Update to risks and AESIs sections regarding the MHRA & JCVI recommendations regarding reports of thrombosis and thrombocytopenia.
SA25	V20.0		Daniel Jenkin	Added details on recruitment of COV002 participants to booster studies<br>Added details on COV009 follow on safety and immunogenicity study

List details of all protocol amendments here whenever a new version of the protocol is produced.

17 Appendix

Investigator Agreement and Notification of Conflict of Interest

I approve this protocol for use in the above named clinical trial and agree to abide by all provisions set forth therein.

According to the Declaration of Helsinki, 2008, I have read this protocol, and declare no/~~the following (delete as appropriate)~~ conflict of interest

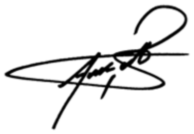

Prof Andrew Pollard		07/06/2021
Chief Investigator	Signature	Date

Site: **Centre for Clinical Vaccinology and Tropical Medicine, University of Oxford**

I have read this protocol and agree to abide by all provisions set forth therein.

According to the Declaration of Helsinki, 2008, I have read this protocol, and declare the following conflict of interest. AH is a cofounder of and minor shareholder in an Oxford University spin-off company, Vaccitech Ltd, that has some non-exclusive rights to the vector, ChAdOx1, used in the vaccine to be tested, that may be of commercial value"

Principal Investigator<br>Prof Adrian Hill	Signature	Date: 07/06/2021

Site: **NIHR WTCRF**

I have read this protocol and agree to abide by all provisions set forth therein.

According to the Declaration of Helsinki, 2008, I have read this protocol, and declare no/~~the following (delete as appropriate)~~ conflict of interest

Principal Investigator<br>Prof Saul Faust	Signature	Date: 08/06/2021

Site: **NIHR Imperial CRF**

I have read this protocol and agree to abide by all provisions set forth therein.

According to the Declaration of Helsinki, 2008, I have read this protocol, and declare no/~~the following (delete as appropriate)~~ conflict of interest

Principal Investigator<br><br>Dr Katrina M. Pollock	Signature	Date: 08/06/2021

Site: **Oxford University Hospital Foundation Trust**

I have read this protocol and agree to abide by all provisions set forth therein.

According to the Declaration of Helsinki, 2008, I have read this protocol, and declare no/~~the following (delete as appropriate)~~ conflict of interest

Principal Investigator<br><br>Dr Maheshi Ramasamy	Signature	Date: 07/06/2021

Site: **St Georges University Hospital NHS Foundation Trust**

I have read this protocol and agree to abide by all provisions set forth therein.

According to the Declaration of Helsinki, 2008, I have read this protocol, and declare no/~~the following (delete as appropriate)~~ conflict of interest

Principal Investigator<br><br>Prof Paul Heath	Signature	Date: 07/06/2021

Site: **University Hospitals Bristol and Weston NHS Foundation Trust**

I have read this protocol and agree to abide by all provisions set forth therein.

According to the Declaration of Helsinki, 2008, I have read this protocol, and declare no/~~the following (delete as appropriate)~~ conflict of interest

Principal Investigator<br><br>Prof Adam Finn	Signature	Date: 08/06/2021

Site: **North Bristol NHS Trust**

I have read this protocol and agree to abide by all provisions set forth therein.

According to the Declaration of Helsinki, 2008, I have read this protocol, and declare no/~~the following (delete as appropriate)~~ conflict of interest

Principal Investigator	Signature	Date:
Dr Rajeka Lazarus		07/06/2021

Site: **University of Nottingham Health Service and Nottingham University Hospitals NHS Trust**

I have read this protocol and agree to abide by all provisions set forth therein.

According to the Declaration of Helsinki, 2008, I have read this protocol, and declare no/~~the following (delete as appropriate)~~ conflict of interest

Principal Investigator	Signature	Date: 07/06/2021
Dr David Turner		

Site: **Sheffield Teaching Hospitals**

I have read this protocol and agree to abide by all provisions set forth therein.

According to the Declaration of Helsinki, 2008, I have read this protocol, and declare no/~~the following (delete as appropriate)~~ conflict of interest

Principal Investigator	Signature	Date: 15/06/2021
Dr. Thomas Darton		

Site: **University Hospitals Birmingham NHS Foundation Trust (UHB)**

I have read this protocol and agree to abide by all provisions set forth therein.

According to the Declaration of Helsinki, 2008, I have read this protocol, and declare no/~~the following (delete as appropriate)~~ conflict of interest

Principal Investigator	Signature	Date: 07/06/2021
Dr Christopher Green		

Site: **Wales (Public Health Wales)**

I have read this protocol and agree to abide by all provisions set forth therein.

According to the Declaration of Helsinki, 2008, I have read this protocol, and declare no/~~the following (delete as appropriate)~~ conflict of interest

Principal Investigator<br>Dr Chris J Williams	Signature	Date: 09/06/2021

Site: **Castle Hill Hospital**

I have read this protocol and agree to abide by all provisions set forth therein.

According to the Declaration of Helsinki, 2008, I have read this protocol, and declare no/~~the following (delete as appropriate)~~ conflict of interest

Principal Investigator<br>Dr Patrick Lillie	Signature	Date: 07/06/2021

Site: **NHS Greater Glasgow & Clyde Hospitals**

I have read this protocol and agree to abide by all provisions set forth therein.

According to the Declaration of Helsinki, 2008, I have read this protocol, and declare no/~~the following (delete as appropriate)~~ conflict of interest

Principal Investigator<br>Professor Emma Thomson	Signature	Date: 07/06/2021

Site: **Guy's and St Thomas' NHS Foundation Trust**

I have read this protocol and agree to abide by all provisions set forth therein.

According to the Declaration of Helsinki, 2008, I have read this protocol, and declare no/~~the following (delete as appropriate)~~ conflict of interest

Principal Investigator<br>Dr Anna Goodman	Signature	Date: 07/06/2021

Site: **Liverpool School of Tropical Medicine (LSTM)**

I have read this protocol and agree to abide by all provisions set forth therein.

According to the Declaration of Helsinki, 2008, I have read this protocol, and declare no/~~the following (delete as appropriate)~~ conflict of interest

Principal Investigator<br>Dr Andrea Collins	Signature	Date: 09/06/2021

Site: **The Newcastle upon Tyne Hospitals NHS Foundation Trust**

I have read this protocol and agree to abide by all provisions set forth therein.

According to the Declaration of Helsinki, 2008, I have read this protocol, and declare no/~~the following (delete as appropriate)~~ conflict of interest

Principal Investigator<br>Dr Christopher Duncan	Signature	Date: 08/06/2021

Site: **UCLH**

I have read this protocol and agree to abide by all provisions set forth therein.

According to the Declaration of Helsinki, 2008, I have read this protocol, and declare no/~~the following (delete as appropriate)~~ conflict of interest

Principal Investigator<br><br>Prof Vincenzo Libri	Signature	Date: 07/06/2021

Site: **NHS Lothian**

I have read this protocol and agree to abide by all provisions set forth therein.

According to the Declaration of Helsinki, 2008, I have read this protocol, and declare no/~~the following (delete as appropriate)~~ conflict of interest

Principal Investigator<br><br>Dr Rebecca Sutherland	Signature	Date: 08/06/2021

Site: **Cambridge**

I have read this protocol and agree to abide by all provisions set forth therein.

According to the Declaration of Helsinki, 2008, I have read this protocol, and declare no/~~the following (delete as appropriate)~~ conflict of interest

Principal Investigator<br><br>Dr Mark Toshner	Signature	Date: 11/06/2021

Site: **Northwick Park**

I have read this protocol and agree to abide by all provisions set forth therein.

According to the Declaration of Helsinki, 2008, I have read this protocol, and declare no/~~the following (delete as appropriate)~~ conflict of interest

Principal Investigator<br><br>Dr Alastair McGregor	Signature	Date: 09/06/2021
