# Supplementary material for: Reactogenicity and immunogenicity after a late second dose or a third dose of ChAdOx1 nCoV-19 in the UK: a substudy of two randomised controlled trials (COV001 and COV002)
Source: Lancet. 2021 Sep 11;398(10304):981–90. doi: 10.1016/S0140-6736(21)01699-8 (PMC8409975; doi:10.1016/S0140-6736(21)01699-8)
Supplement: Supplementary appendix 3 [file mmc3.pdf]

THE LANCET

Supplementary appendix

This appendix formed part of the original submission and has been peer reviewed. We post it as supplied by the authors.

Supplement to: Flaxman A, Marchevsky NG, Jenkin D, et al. Reactogenicity and immunogenicity after a late second dose or a third dose of ChAdOx1 nCoV-19 in the UK: a substudy of two randomised controlled trials (COV001 and COV002). *Lancet* 2021; published online Sept 1. http://dx.doi.org/10.1016/S0140-6736(21)01699-8.

Supplementary Methods

MSD multi-plex immunoassay

V-PLEX SARS-CoV-2 Panel 6 (IgG) kits were used following manufacturer's instructions (Meso Scale Discovery, K15433U) Briefly, plates pre-coated with a panel of 7 SARS-CoV-2 WT and variant antigens were blocked for 30 minutes at room temperature with shaking (600rpm) and washed 3x with PBS-T. Reference calibrator and three internal controls provided in the kit were diluted as instructed and test sera were diluted 1:2000 and plated in duplicate. Plates were incubated as above for 2 hours, and then washed 3x with PBS-T. Wells were then incubated in the dark as above with SULFO-TAG Anti-human IgG for 1 hour. Plates were washed 3x with PBS-T followed by a final wash with PBS and read within 5 minutes of the addition of read buffer on a MESO QuickPlex SQ 120MM plate reader with Methodical Mind ™ 2.0.15 software. Data was exported and analysed using MSD Discovery Workbench v4.0 ensuring the following QC criteria were met: sample CVs < 20%; the IC calculated concentration was within 70-130% of expected concentration; and the reference standard percentage recovery was within 80-120%. Samples with signals above the detection limit of the assay were repeated at a higher dilution to ensure that the signal fell within the detection range of the assay.

Delta Variant ELISA

To measure antibody responses to Delta variant (B.1.617.2) the ELISA assay as previously described[1] was adapted to coat with 2.0ug/ml of SARS-CoV-2 spike protein from Delta variant, provided by AstraZeneca.

Supplementary Figures

Supplementary Figure 1 – CONSORT flow diagram for single dose persistence cohorts

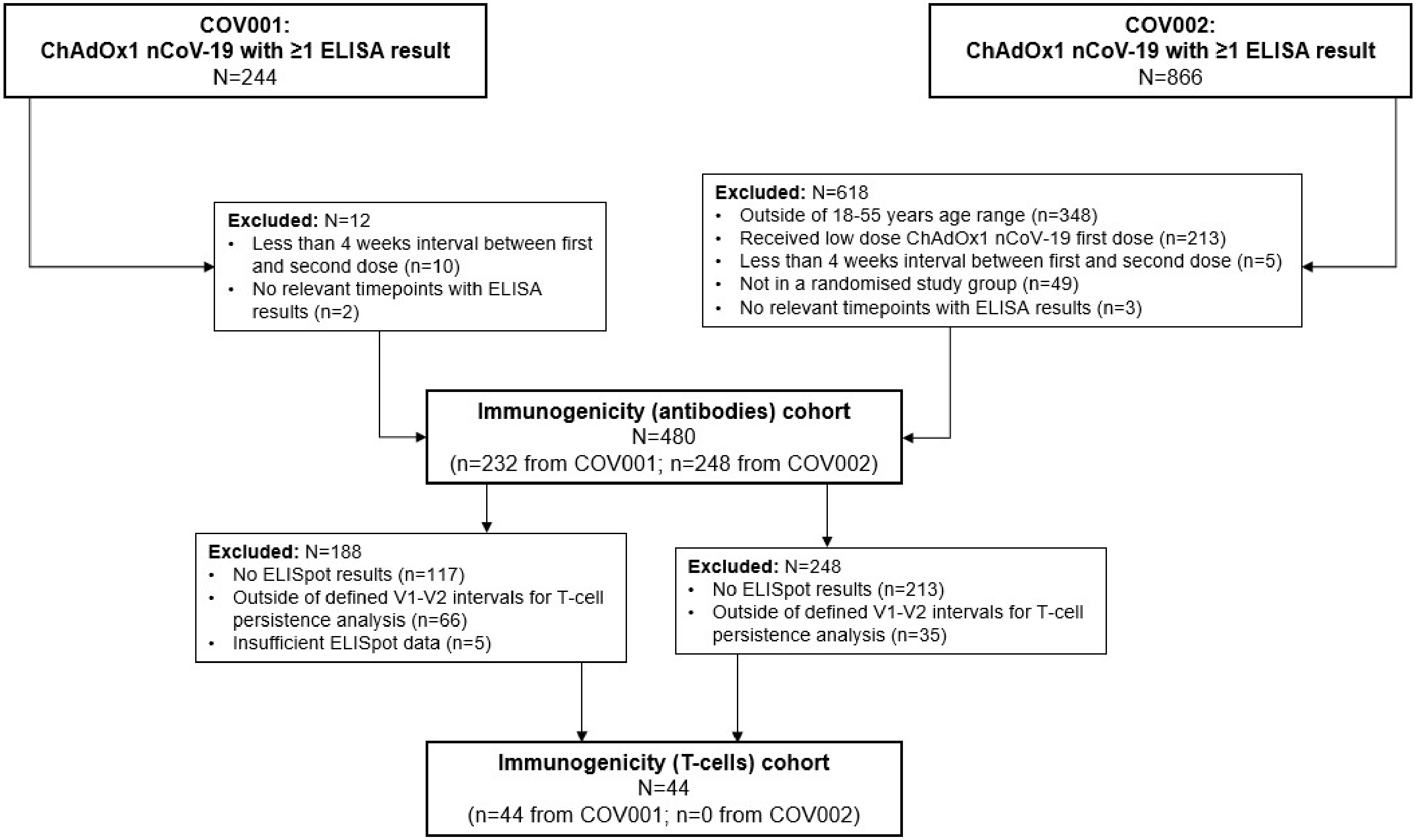

Supplementary Figure 2 – CONSORT flow diagram for two dose cohorts. SD = standard dose.

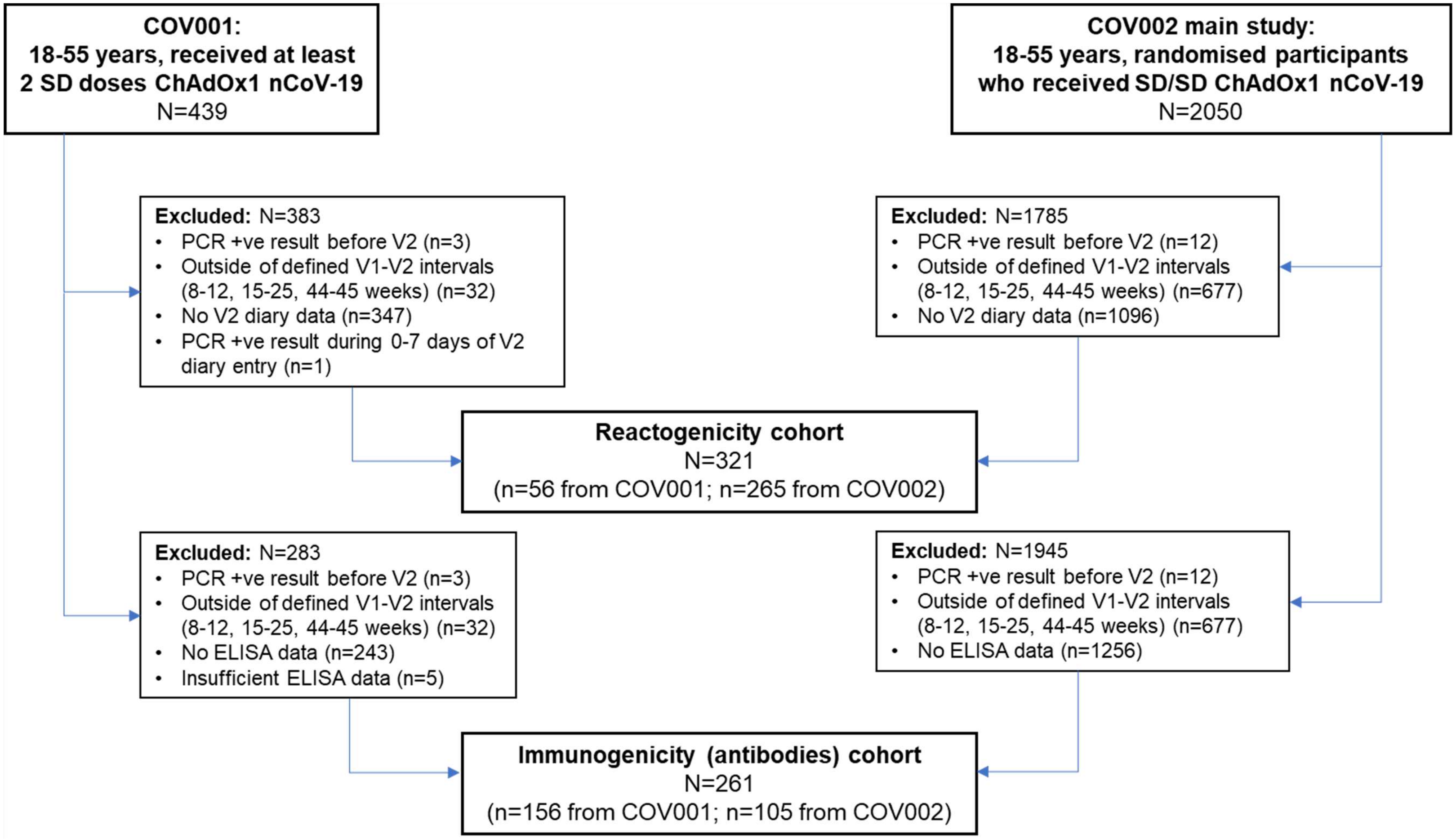

Supplementary Tables

Supplementary Table 1- Baseline characteristics of the single dose persistence cohort

	**Immunogenicity: antibodies**	**Immunogenicity: T-cells**
Number of participants	480	44
Sex (female)	237 (49.4%)	19 (43.2%)
Age (median [IQR])	37.2 [29.0, 47.0]	31.0 [24.1, 40.4]
18-29 years	133 (27.7%)	18 (40.9%)
30-39 years	136 (28.3%)	14 (31.8%)
40-55 years	211 (44.0%)	12 (27.3%)
BMI (median [IQR])	24.9 [22.4, 27.8]	23.7 [21.8, 25.8]
Ethnicity:		
White	435 (90.6%)	40 (90.9%)
Black	5 (1.0%)	0 (0.0%)
Asian	23 (4.8%)	1 (2.3%)
Other	17 (3.5%)	3 (6.8%)
Missing	0 (0.0%)	0 (0.0%)

Supplementary Table 1 - Baseline characteristics of the two dose cohort

	Reactogencity			Immunogenicity: antibodies		
	**All**	**COV001**	**COV002**	**All**	**COV001**	**COV002**
Number of participants	321	56	265	261	156	105
Sex (female)	204 (63.6%)	31 (55.4%)	173 (65.3%)	124 (47.5%)	67 (42.9%)	57 (54.3%)
Age (median [IQR])	41.0 [30.2, 49.0]	33.5 [26.4, 44.1]	42.0 [32.0, 49.3]	37.2 [29.4, 46.0]	35.0 [29.4, 43.7]	39.0 [30.0, 50.0]
18-29 years	74 (23.1%)	17 (30.4%)	57 (21.5%)	68 (26.1%)	42 (26.9%)	26 (24.8%)
30-39 years	74 (23.1%)	18 (32.1%)	56 (21.1%)	82 (31.4%)	55 (35.3%)	27 (25.7%)
40-55 years	173 (53.9%)	21 (37.5%)	152 (57.4%)	111 (42.5%)	59 (37.8%)	52 (49.5%)
BMI (median [IQR])	24.8 [22.4, 28.2]	23.8 [21.3, 26.3]	25.1 [22.8, 28.4]	24.5 [22.2, 27.3]	24.0 [22.2, 26.8]	24.9 [22.1, 27.7]
Ethnicity:						
White	299 (93.1%)	51 (91.1%)	248 (93.6%)	238 (91.2%)	141 (90.4%)	97 (92.4%)
Black	1 (0.3%)	1 (1.8%)	0 (0.0%)	3 (1.1%)	3 (1.9%)	0 (0.0%)
Asian	14 (4.4%)	0 (0.0%)	14 (5.3%)	10 (3.8%)	4 (2.6%)	6 (5.7%)
Other	7 (2.2%)	4 (7.1%)	3 (1.1%)	10 (3.8%)	8 (5.1%)	2 (1.9%)
Missing	0 (0.0%)	0 (0.0%)	0 (0.0%)	0 (0.0%)	0 (0.0%)	0 (0.0%)
V1-V2 interval:						
<8 weeks	0 (0.0%)	0 (0.0%)	0 (0.0%)	0 (0.0%)	0 (0.0%)	0 (0.0%)
8-12 weeks	267 (83.2%)	20 (35.7%)	247 (93.2%)	115 (44.1%)	20 (12.8%)	95 (90.5%)
15-25 weeks	24 (7.5%)	6 (10.7%)	18 (6.8%)	116 (44.4%)	106 (67.9%)	10 (9.5%)
44-45 weeks	30 (9.3%)	30 (53.6%)	0 (0.0%)	30 (11.5%)	30 (19.2%)	0 (0.0%)

Supplementary Table 2 - Baseline characteristics of the third dose cohort

	**Reactogencity**	**Immunogenicity: antibodies**	**Immunogenicity: T-cells**
Number of participants	80	75	15
Sex (female)	32 (40.0%)	29 (38.7%)	10 (66.7%)
Age (median [IQR])	37.0 [30.6, 41.9]	37.2 [30.8, 42.2]	39.5 [32.1, 44.2]
18-29 years	16 (20.0%)	14 (18.7%)	3 (20.0%)
30-39 years	36 (45.0%)	33 (44.0%)	5 (33.3%)
40-55 years	28 (35.0%)	28 (37.3%)	7 (46.7%)
BMI (median [IQR])	24.0 [22.5, 26.7]	24.0 [22.6, 26.8]	23.9 [22.0, 29.2]
Ethnicity:			
White	71 (88.8%)	67 (89.3%)	14 (93.3%)
Black	1 (1.2%)	1 (1.3%)	1 (6.7%)
Asian	3 (3.8%)	2 (2.7%)	0 (0.0%)
Other	5 (6.2%)	5 (6.7%)	0 (0.0%)
Missing	0 (0.0%)	0 (0.0%)	0 (0.0%)
V1-V2 interval:			
<8 weeks	0 (0.0%)	0 (0.0%)	0 (0.0%)
8-12 weeks	15 (18.8%)	15 (20.0%)	15 (100.0%)
15-25 weeks	65 (81.2%)	60 (80.0%)	0 (0.0%)
44-45 weeks	0 (0.0%)	0 (0.0%)	0 (0.0%)

Supplementary Table 4 - Local and solicited adverse reactions in the first 7 days after the second dose of vaccine for the two dose cohort

Symptom	V1-V2 Interval	None	Mild	Moderate	Severe	Potentially life threatening/ hospitalisation	Any
Pain	8-12 weeks	170/267 (64%, 58%-69%)	96/267 (36%, 30%-42%)	1/267 (0%, 0%-2%)	0/267 (0%, 0%-1%)	0/267 (0%, 0%-1%)	97/267 (36%, 31%-42%)
	15-25 weeks	13/24 (54%, 33%-74%)	9/24 (38%, 19%-59%)	2/24 (8%, 1%-27%)	0/24 (0%, 0%-14%)	0/24 (0%, 0%-14%)	11/24 (46%, 26%-67%)
	44-45 weeks	18/30 (60%, 41%-77%)	9/30 (30%, 15%-49%)	3/30 (10%, 2%-27%)	0/30 (0%, 0%-12%)	0/30 (0%, 0%-12%)	12/30 (40%, 23%-59%)
Redness	8-12 weeks	266/267 (100%, 98%-100%)	1/267 (0%, 0%-2%)	0/267 (0%, 0%-1%)	0/267 (0%, 0%-1%)	0/267 (0%, 0%-1%)	1/267 (0%, 0%-2%)
	15-25 weeks	24/24 (100%, 86%-100%)	0/24 (0%, 0%-14%)	0/24 (0%, 0%-14%)	0/24 (0%, 0%-14%)	0/24 (0%, 0%-14%)	0/24 (0%, 0%-14%)
	44-45 weeks	30/30 (100%, 88%-100%)	0/30 (0%, 0%-12%)	0/30 (0%, 0%-12%)	0/30 (0%, 0%-12%)	0/30 (0%, 0%-12%)	0/30 (0%, 0%-12%)
Warmth	8-12 weeks	234/267 (88%, 83%-91%)	32/267 (12%, 8%-16%)	1/267 (0%, 0%-2%)	0/267 (0%, 0%-1%)	0/267 (0%, 0%-1%)	33/267 (12%, 9%-17%)
	15-25 weeks	21/24 (88%, 68%-97%)	3/24 (12%, 3%-32%)	0/24 (0%, 0%-14%)	0/24 (0%, 0%-14%)	0/24 (0%, 0%-14%)	3/24 (12%, 3%-32%)
	44-45 weeks	25/30 (83%, 65%-94%)	5/30 (17%, 6%-35%)	0/30 (0%, 0%-12%)	0/30 (0%, 0%-12%)	0/30 (0%, 0%-12%)	5/30 (17%, 6%-35%)
Itch	8-12 weeks	255/267 (96%, 92%-98%)	12/267 (4%, 2%-8%)	0/267 (0%, 0%-1%)	0/267 (0%, 0%-1%)	0/267 (0%, 0%-1%)	12/267 (4%, 2%-8%)
	15-25 weeks	23/24 (96%, 79%-100%)	1/24 (4%, 0%-21%)	0/24 (0%, 0%-14%)	0/24 (0%, 0%-14%)	0/24 (0%, 0%-14%)	1/24 (4%, 0%-21%)
	44-45 weeks	29/30 (97%, 83%-100%)	1/30 (3%, 0%-17%)	0/30 (0%, 0%-12%)	0/30 (0%, 0%-12%)	0/30 (0%, 0%-12%)	1/30 (3%, 0%-17%)
Swelling	8-12 weeks	267/267 (100%, 99%-100%)	0/267 (0%, 0%-1%)	0/267 (0%, 0%-1%)	0/267 (0%, 0%-1%)	0/267 (0%, 0%-1%)	0/267 (0%, 0%-1%)
	15-25 weeks	23/24 (96%, 79%-100%)	1/24 (4%, 0%-21%)	0/24 (0%, 0%-14%)	0/24 (0%, 0%-14%)	0/24 (0%, 0%-14%)	1/24 (4%, 0%-21%)
	44-45 weeks	30/30 (100%, 88%-100%)	0/30 (0%, 0%-12%)	0/30 (0%, 0%-12%)	0/30 (0%, 0%-12%)	0/30 (0%, 0%-12%)	0/30 (0%, 0%-12%)
Induration	8-12 weeks	266/267 (100%, 98%-100%)	1/267 (0%, 0%-2%)	0/267 (0%, 0%-1%)	0/267 (0%, 0%-1%)	0/267 (0%, 0%-1%)	1/267 (0%, 0%-2%)
	15-25 weeks	24/24 (100%, 86%-100%)	0/24 (0%, 0%-14%)	0/24 (0%, 0%-14%)	0/24 (0%, 0%-14%)	0/24 (0%, 0%-14%)	0/24 (0%, 0%-14%)
	44-45 weeks	30/30 (100%, 88%-100%)	0/30 (0%, 0%-12%)	0/30 (0%, 0%-12%)	0/30 (0%, 0%-12%)	0/30 (0%, 0%-12%)	0/30 (0%, 0%-12%)
Tenderness	8-12 weeks	81/267 (30%, 25%-36%)	181/267 (68%, 62%-73%)	5/267 (2%, 1%-4%)	0/267 (0%, 0%-1%)	0/267 (0%, 0%-1%)	186/267 (70%, 64%-75%)
	15-25 weeks	10/24 (42%, 22%-63%)	12/24 (50%, 29%-71%)	2/24 (8%, 1%-27%)	0/24 (0%, 0%-14%)	0/24 (0%, 0%-14%)	14/24 (58%, 37%-78%)

Symptom	V1-V2 Interval	None	Mild	Moderate	Severe	Potentially life threatening/ hospitalisation	Any
	44-45 weeks	9/30 (30%, 15%-49%)	17/30 (57%, 37%-75%)	4/30 (13%, 4%-31%)	0/30 (0%, 0%-12%)	0/30 (0%, 0%-12%)	21/30 (70%, 51%-85%)
Feverish	8-12 weeks	232/267 (87%, 82%-91%)	25/267 (9%, 6%-14%)	8/267 (3%, 1%-6%)	2/267 (1%, 0%-3%)	0/267 (0%, 0%-1%)	35/267 (13%, 9%-18%)
	15-25 weeks	21/24 (88%, 68%-97%)	1/24 (4%, 0%-21%)	2/24 (8%, 1%-27%)	0/24 (0%, 0%-14%)	0/24 (0%, 0%-14%)	3/24 (12%, 3%-32%)
	44-45 weeks	20/30 (67%, 47%-83%)	5/30 (17%, 6%-35%)	5/30 (17%, 6%-35%)	0/30 (0%, 0%-12%)	0/30 (0%, 0%-12%)	10/30 (33%, 17%-53%)
Fever ≥38°C	8-12 weeks	261/263 (99%, 97%-100%)	1/263 (0%, 0%-2%)	1/263 (0%, 0%-2%)	0/263 (0%, 0%-1%)	0/263 (0%, 0%-1%)	2/263 (1%, 0%-3%)
	15-25 weeks	22/23 (96%, 78%-100%)	0/23 (0%, 0%-15%)	0/23 (0%, 0%-15%)	1/23 (4%, 0%-22%)	0/23 (0%, 0%-15%)	1/23 (4%, 0%-22%)
	44-45 weeks	27/28 (96%, 82%-100%)	1/28 (4%, 0%-18%)	0/28 (0%, 0%-12%)	0/28 (0%, 0%-12%)	0/28 (0%, 0%-12%)	1/28 (4%, 0%-18%)
Chills	8-12 weeks	252/267 (94%, 91%-97%)	12/267 (4%, 2%-8%)	3/267 (1%, 0%-3%)	0/267 (0%, 0%-1%)	0/267 (0%, 0%-1%)	15/267 (6%, 3%-9%)
	15-25 weeks	19/24 (79%, 58%-93%)	3/24 (12%, 3%-32%)	2/24 (8%, 1%-27%)	0/24 (0%, 0%-14%)	0/24 (0%, 0%-14%)	5/24 (21%, 7%-42%)
	44-45 weeks	18/30 (60%, 41%-77%)	11/30 (37%, 20%-56%)	1/30 (3%, 0%-17%)	0/30 (0%, 0%-12%)	0/30 (0%, 0%-12%)	12/30 (40%, 23%-59%)
Joint pain	8-12 weeks	230/267 (86%, 81%-90%)	30/267 (11%, 8%-16%)	7/267 (3%, 1%-5%)	0/267 (0%, 0%-1%)	0/267 (0%, 0%-1%)	37/267 (14%, 10%-19%)
	15-25 weeks	21/24 (88%, 68%-97%)	3/24 (12%, 3%-32%)	0/24 (0%, 0%-14%)	0/24 (0%, 0%-14%)	0/24 (0%, 0%-14%)	3/24 (12%, 3%-32%)
	44-45 weeks	22/30 (73%, 54%-88%)	6/30 (20%, 8%-39%)	2/30 (7%, 1%-22%)	0/30 (0%, 0%-12%)	0/30 (0%, 0%-12%)	8/30 (27%, 12%-46%)
Muscle ache	8-12 weeks	193/267 (72%, 67%-78%)	64/267 (24%, 19%-30%)	10/267 (4%, 2%-7%)	0/267 (0%, 0%-1%)	0/267 (0%, 0%-1%)	74/267 (28%, 22%-33%)
	15-25 weeks	14/24 (58%, 37%-78%)	9/24 (38%, 19%-59%)	1/24 (4%, 0%-21%)	0/24 (0%, 0%-14%)	0/24 (0%, 0%-14%)	10/24 (42%, 22%-63%)
	44-45 weeks	14/30 (47%, 28%-66%)	11/30 (37%, 20%-56%)	5/30 (17%, 6%-35%)	0/30 (0%, 0%-12%)	0/30 (0%, 0%-12%)	16/30 (53%, 34%-72%)
Fatigue	8-12 weeks	141/267 (53%, 47%-59%)	86/267 (32%, 27%-38%)	38/267 (14%, 10%-19%)	2/267 (1%, 0%-3%)	0/267 (0%, 0%-1%)	126/267 (47%, 41%-53%)
	15-25 weeks	12/24 (50%, 29%-71%)	9/24 (38%, 19%-59%)	3/24 (12%, 3%-32%)	0/24 (0%, 0%-14%)	0/24 (0%, 0%-14%)	12/24 (50%, 29%-71%)
	44-45 weeks	9/30 (30%, 15%-49%)	15/30 (50%, 31%-69%)	6/30 (20%, 8%-39%)	0/30 (0%, 0%-12%)	0/30 (0%, 0%-12%)	21/30 (70%, 51%-85%)
Headache	8-12 weeks	142/267 (53%, 47%-59%)	103/267 (39%, 33%-45%)	22/267 (8%, 5%-12%)	0/267 (0%, 0%-1%)	0/267 (0%, 0%-1%)	125/267 (47%, 41%-53%)
	15-25 weeks	16/24 (67%, 45%-84%)	7/24 (29%, 13%-51%)	1/24 (4%, 0%-21%)	0/24 (0%, 0%-14%)	0/24 (0%, 0%-14%)	8/24 (33%, 16%-55%)

Symptom	V1-V2 Interval	None	Mild	Moderate	Severe	Potentially life threatening/ hospitalisation	Any
	44-45 weeks	9/30 (30%, 15%-49%)	17/30 (57%, 37%-75%)	3/30 (10%, 2%-27%)	1/30 (3%, 0%-17%)	0/30 (0%, 0%-12%)	21/30 (70%, 51%-85%)
Malaise	8-12 weeks	200/267 (75%, 69%-80%)	50/267 (19%, 14%-24%)	15/267 (6%, 3%-9%)	2/267 (1%, 0%-3%)	0/267 (0%, 0%-1%)	67/267 (25%, 20%-31%)
	15-25 weeks	18/24 (75%, 53%-90%)	4/24 (17%, 5%-37%)	2/24 (8%, 1%-27%)	0/24 (0%, 0%-14%)	0/24 (0%, 0%-14%)	6/24 (25%, 10%-47%)
	44-45 weeks	17/30 (57%, 37%-75%)	8/30 (27%, 12%-46%)	5/30 (17%, 6%-35%)	0/30 (0%, 0%-12%)	0/30 (0%, 0%-12%)	13/30 (43%, 25%-63%)
Nausea	8-12 weeks	234/267 (88%, 83%-91%)	27/267 (10%, 7%-14%)	4/267 (1%, 0%-4%)	2/267 (1%, 0%-3%)	0/267 (0%, 0%-1%)	33/267 (12%, 9%-17%)
	15-25 weeks	23/24 (96%, 79%-100%)	1/24 (4%, 0%-21%)	0/24 (0%, 0%-14%)	0/24 (0%, 0%-14%)	0/24 (0%, 0%-14%)	1/24 (4%, 0%-21%)
	44-45 weeks	25/30 (83%, 65%-94%)	4/30 (13%, 4%-31%)	1/30 (3%, 0%-17%)	0/30 (0%, 0%-12%)	0/30 (0%, 0%-12%)	5/30 (17%, 6%-35%)

Supplementary Table 5 - Overall summary of local and solicited adverse reactions in the first 7 days after each dose of vaccine for the two dose cohort

Interval group	Symptom	Dose	None	Any	Mild	Moderate	Severe	Moderate or severe	>2 moderate/severe symptoms
8-12 weeks	Any	1	4/267 (1%, 0%-4%)	263/267 (99%, 96%-100%)	149/267 (56%, 50%-62%)	95/267 (36%, 30%-42%)	19/267 (7%, 4%-11%)	114/267 (43%, 37%-49%)	63/267 (24%, 19%-29%)
		2	27/267 (10%, 7%-14%)	240/267 (90%, 86%-93%)	183/267 (69%, 63%-74%)	52/267 (19%, 15%-25%)	5/267 (2%, 1%-4%)	57/267 (21%, 17%-27%)	13/267 (5%, 3%-8%)
	Local	1	28/267 (10%, 7%-15%)	239/267 (90%, 85%-93%)	203/267 (76%, 70%-81%)	35/267 (13%, 9%-18%)	1/267 (0%, 0%-2%)	36/267 (13%, 10%-18%)	1/267 (0%, 0%-2%)
		2	66/267 (25%, 20%-30%)	201/267 (75%, 70%-80%)	195/267 (73%, 67%-78%)	6/267 (2%, 1%-5%)	0/267 (0%, 0%-1%)	6/267 (2%, 1%-5%)	0/267 (0%, 0%-1%)
	Systemic	1	33/267 (12%, 9%-17%)	234/267 (88%, 83%-91%)	127/267 (48%, 41%-54%)	88/267 (33%, 27%-39%)	19/267 (7%, 4%-11%)	107/267 (40%, 34%-46%)	57/267 (21%, 17%-27%)
		2	77/267 (29%, 23%-35%)	190/267 (71%, 65%-77%)	135/267 (51%, 44%-57%)	50/267 (19%, 14%-24%)	5/267 (2%, 1%-4%)	55/267 (21%, 16%-26%)	13/267 (5%, 3%-8%)
15-25 weeks	Any	1	2/24 (8%, 1%-27%)	22/24 (92%, 73%-99%)	11/24 (46%, 26%-67%)	8/24 (33%, 16%-55%)	3/24 (12%, 3%-32%)	11/24 (46%, 26%-67%)	5/24 (21%, 7%-42%)
		2	5/24 (21%, 7%-42%)	19/24 (79%, 58%-93%)	14/24 (58%, 37%-78%)	4/24 (17%, 5%-37%)	1/24 (4%, 0%-21%)	5/24 (21%, 7%-42%)	2/24 (8%, 1%-27%)
	Local	1	4/24 (17%, 5%-37%)	20/24 (83%, 63%-95%)	17/24 (71%, 49%-87%)	2/24 (8%, 1%-27%)	1/24 (4%, 0%-21%)	3/24 (12%, 3%-32%)	0/24 (0%, 0%-14%)
		2	9/24 (38%, 19%-59%)	15/24 (62%, 41%-81%)	12/24 (50%, 29%-71%)	3/24 (12%, 3%-32%)	0/24 (0%, 0%-14%)	3/24 (12%, 3%-32%)	0/24 (0%, 0%-14%)
	Systemic	1	3/24 (12%, 3%-32%)	21/24 (88%, 68%-97%)	10/24 (42%, 22%-63%)	9/24 (38%, 19%-59%)	2/24 (8%, 1%-27%)	11/24 (46%, 26%-67%)	3/24 (12%, 3%-32%)
		2	6/24 (25%, 10%-47%)	18/24 (75%, 53%-90%)	14/24 (58%, 37%-78%)	3/24 (12%, 3%-32%)	1/24 (4%, 0%-21%)	4/24 (17%, 5%-37%)	2/24 (8%, 1%-27%)
44-45 weeks	Any	1	0/30 (0%, 0%-12%)	30/30 (100%, 88%-100%)	6/30 (20%, 8%-39%)	17/30 (57%, 37%-75%)	7/30 (23%, 10%-42%)	24/30 (80%, 61%-92%)	13/30 (43%, 25%-63%)
		2	2/30 (7%, 1%-22%)	28/30 (93%, 78%-99%)	16/30 (53%, 34%-72%)	11/30 (37%, 20%-56%)	1/30 (3%, 0%-17%)	12/30 (40%, 23%-59%)	7/30 (23%, 10%-42%)
	Local	1	5/30 (17%, 6%-35%)	25/30 (83%, 65%-94%)	20/30 (67%, 47%-83%)	5/30 (17%, 6%-35%)	0/30 (0%, 0%-12%)	5/30 (17%, 6%-35%)	0/30 (0%, 0%-12%)
		2	7/30 (23%, 10%-42%)	23/30 (77%, 58%-90%)	19/30 (63%, 44%-80%)	4/30 (13%, 4%-31%)	0/30 (0%, 0%-12%)	4/30 (13%, 4%-31%)	0/30 (0%, 0%-12%)
	Systemic	1	1/30 (3%, 0%-17%)	29/30 (97%, 83%-100%)	6/30 (20%, 8%-39%)	16/30 (53%, 34%-72%)	7/30 (23%, 10%-42%)	23/30 (77%, 58%-90%)	12/30 (40%, 23%-59%)
		2	4/30 (13%, 4%-31%)	26/30 (87%, 69%-96%)	16/30 (53%, 34%-72%)	9/30 (30%, 15%-49%)	1/30 (3%, 0%-17%)	10/30 (33%, 17%-53%)	6/30 (20%, 8%-39%)

Supplementary Table 6 - Local and solicited adverse reactions in the first 7 days after vaccine for the third dose cohort

Symptom	Dose	None	Mild	Moderate	Severe	Potentially life threatening/ hospitalisation	Any
**Pain**	1	32/80 (40%, 29%-52%)	40/80 (50%, 39%-61%)	7/80 (9%, 4%-17%)	1/80 (1%, 0%-7%)	0/80 (0%, 0%-5%)	48/80 (60%, 48%-71%)
	2	10/15 (67%, 38%-88%)	5/15 (33%, 12%-62%)	0/15 (0%, 0%-22%)	0/15 (0%, 0%-22%)	0/15 (0%, 0%-22%)	5/15 (33%, 12%-62%)
	3	40/80 (50%, 39%-61%)	32/80 (40%, 29%-52%)	8/80 (10%, 4%-19%)	0/80 (0%, 0%-5%)	0/80 (0%, 0%-5%)	40/80 (50%, 39%-61%)
**Redness**	1	77/80 (96%, 89%-99%)	2/80 (2%, 0%-9%)	1/80 (1%, 0%-7%)	0/80 (0%, 0%-5%)	0/80 (0%, 0%-5%)	3/80 (4%, 1%-11%)
	2	15/15 (100%, 78%-100%)	0/15 (0%, 0%-22%)	0/15 (0%, 0%-22%)	0/15 (0%, 0%-22%)	0/15 (0%, 0%-22%)	0/15 (0%, 0%-22%)
	3	79/80 (99%, 93%-100%)	1/80 (1%, 0%-7%)	0/80 (0%, 0%-5%)	0/80 (0%, 0%-5%)	0/80 (0%, 0%-5%)	1/80 (1%, 0%-7%)
**Warmth**	1	69/80 (86%, 77%-93%)	10/80 (12%, 6%-22%)	1/80 (1%, 0%-7%)	0/80 (0%, 0%-5%)	0/80 (0%, 0%-5%)	11/80 (14%, 7%-23%)
	2	14/15 (93%, 68%-100%)	1/15 (7%, 0%-32%)	0/15 (0%, 0%-22%)	0/15 (0%, 0%-22%)	0/15 (0%, 0%-22%)	1/15 (7%, 0%-32%)
	3	67/80 (84%, 74%-91%)	13/80 (16%, 9%-26%)	0/80 (0%, 0%-5%)	0/80 (0%, 0%-5%)	0/80 (0%, 0%-5%)	13/80 (16%, 9%-26%)
**Itch**	1	73/80 (91%, 83%-96%)	7/80 (9%, 4%-17%)	0/80 (0%, 0%-5%)	0/80 (0%, 0%-5%)	0/80 (0%, 0%-5%)	7/80 (9%, 4%-17%)
	2	14/15 (93%, 68%-100%)	1/15 (7%, 0%-32%)	0/15 (0%, 0%-22%)	0/15 (0%, 0%-22%)	0/15 (0%, 0%-22%)	1/15 (7%, 0%-32%)
	3	73/80 (91%, 83%-96%)	7/80 (9%, 4%-17%)	0/80 (0%, 0%-5%)	0/80 (0%, 0%-5%)	0/80 (0%, 0%-5%)	7/80 (9%, 4%-17%)
**Swelling**	1	77/80 (96%, 89%-99%)	3/80 (4%, 1%-11%)	0/80 (0%, 0%-5%)	0/80 (0%, 0%-5%)	0/80 (0%, 0%-5%)	3/80 (4%, 1%-11%)

Symptom	Dose	None	Mild	Moderate	Severe	Potentially life threatening/ hospitalisation	Any
	2	15/15 (100%, 78%-100%)	0/15 (0%, 0%-22%)	0/15 (0%, 0%-22%)	0/15 (0%, 0%-22%)	0/15 (0%, 0%-22%)	0/15 (0%, 0%-22%)
	3	79/80 (99%, 93%-100%)	1/80 (1%, 0%-7%)	0/80 (0%, 0%-5%)	0/80 (0%, 0%-5%)	0/80 (0%, 0%-5%)	1/80 (1%, 0%-7%)
**Induration**	1	78/80 (98%, 91%-100%)	1/80 (1%, 0%-7%)	1/80 (1%, 0%-7%)	0/80 (0%, 0%-5%)	0/80 (0%, 0%-5%)	2/80 (2%, 0%-9%)
	2	15/15 (100%, 78%-100%)	0/15 (0%, 0%-22%)	0/15 (0%, 0%-22%)	0/15 (0%, 0%-22%)	0/15 (0%, 0%-22%)	0/15 (0%, 0%-22%)
	3	79/80 (99%, 93%-100%)	1/80 (1%, 0%-7%)	0/80 (0%, 0%-5%)	0/80 (0%, 0%-5%)	0/80 (0%, 0%-5%)	1/80 (1%, 0%-7%)
**Tenderness**	1	14/80 (18%, 10%-28%)	57/80 (71%, 60%-81%)	9/80 (11%, 5%-20%)	0/80 (0%, 0%-5%)	0/80 (0%, 0%-5%)	66/80 (82%, 72%-90%)
	2	6/15 (40%, 16%-68%)	9/15 (60%, 32%-84%)	0/15 (0%, 0%-22%)	0/15 (0%, 0%-22%)	0/15 (0%, 0%-22%)	9/15 (60%, 32%-84%)
	3	20/80 (25%, 16%-36%)	54/80 (68%, 56%-78%)	6/80 (8%, 3%-16%)	0/80 (0%, 0%-5%)	0/80 (0%, 0%-5%)	60/80 (75%, 64%-84%)
**Feverish**	1	38/80 (48%, 36%-59%)	19/80 (24%, 15%-35%)	19/80 (24%, 15%-35%)	4/80 (5%, 1%-12%)	0/80 (0%, 0%-5%)	42/80 (52%, 41%-64%)
	2	13/15 (87%, 60%-98%)	1/15 (7%, 0%-32%)	0/15 (0%, 0%-22%)	1/15 (7%, 0%-32%)	0/15 (0%, 0%-22%)	2/15 (13%, 2%-40%)
	3	68/80 (85%, 75%-92%)	8/80 (10%, 4%-19%)	3/80 (4%, 1%-11%)	1/80 (1%, 0%-7%)	0/80 (0%, 0%-5%)	12/80 (15%, 8%-25%)
**Fever ≥38°C**	1	65/80 (81%, 71%-89%)	6/80 (8%, 3%-16%)	8/80 (10%, 4%-19%)	1/80 (1%, 0%-7%)	0/80 (0%, 0%-5%)	15/80 (19%, 11%-29%)
	2	15/15 (100%, 78%-100%)	0/15 (0%, 0%-22%)	0/15 (0%, 0%-22%)	0/15 (0%, 0%-22%)	0/15 (0%, 0%-22%)	0/15 (0%, 0%-22%)
	3	76/77 (99%, 93%-100%)	1/77 (1%, 0%-7%)	0/77 (0%, 0%-5%)	0/77 (0%, 0%-5%)	0/77 (0%, 0%-5%)	1/77 (1%, 0%-7%)

Symptom	Dose	None	Mild	Moderate	Severe	Potentially life threatening/ hospitalisation	Any
**Chills**	1	38/80 (48%, 36%-59%)	21/80 (26%, 17%-37%)	18/80 (22%, 14%-33%)	3/80 (4%, 1%-11%)	0/80 (0%, 0%-5%)	42/80 (52%, 41%-64%)
	2	15/15 (100%, 78%-100%)	0/15 (0%, 0%-22%)	0/15 (0%, 0%-22%)	0/15 (0%, 0%-22%)	0/15 (0%, 0%-22%)	0/15 (0%, 0%-22%)
	3	67/80 (84%, 74%-91%)	9/80 (11%, 5%-20%)	4/80 (5%, 1%-12%)	0/80 (0%, 0%-5%)	0/80 (0%, 0%-5%)	13/80 (16%, 9%-26%)
**Joint pain**	1	53/80 (66%, 55%-76%)	18/80 (22%, 14%-33%)	8/80 (10%, 4%-19%)	1/80 (1%, 0%-7%)	0/80 (0%, 0%-5%)	27/80 (34%, 24%-45%)
	2	15/15 (100%, 78%-100%)	0/15 (0%, 0%-22%)	0/15 (0%, 0%-22%)	0/15 (0%, 0%-22%)	0/15 (0%, 0%-22%)	0/15 (0%, 0%-22%)
	3	71/80 (89%, 80%-95%)	8/80 (10%, 4%-19%)	1/80 (1%, 0%-7%)	0/80 (0%, 0%-5%)	0/80 (0%, 0%-5%)	9/80 (11%, 5%-20%)
**Muscle ache**	1	29/80 (36%, 26%-48%)	36/80 (45%, 34%-57%)	15/80 (19%, 11%-29%)	0/80 (0%, 0%-5%)	0/80 (0%, 0%-5%)	51/80 (64%, 52%-74%)
	2	13/15 (87%, 60%-98%)	2/15 (13%, 2%-40%)	0/15 (0%, 0%-22%)	0/15 (0%, 0%-22%)	0/15 (0%, 0%-22%)	2/15 (13%, 2%-40%)
	3	53/80 (66%, 55%-76%)	24/80 (30%, 20%-41%)	3/80 (4%, 1%-11%)	0/80 (0%, 0%-5%)	0/80 (0%, 0%-5%)	27/80 (34%, 24%-45%)
**Fatigue**	1	16/80 (20%, 12%-30%)	36/80 (45%, 34%-57%)	26/80 (32%, 22%-44%)	2/80 (2%, 0%-9%)	0/80 (0%, 0%-5%)	64/80 (80%, 70%-88%)
	2	7/15 (47%, 21%-73%)	5/15 (33%, 12%-62%)	2/15 (13%, 2%-40%)	1/15 (7%, 0%-32%)	0/15 (0%, 0%-22%)	8/15 (53%, 27%-79%)
	3	37/80 (46%, 35%-58%)	31/80 (39%, 28%-50%)	11/80 (14%, 7%-23%)	1/80 (1%, 0%-7%)	0/80 (0%, 0%-5%)	43/80 (54%, 42%-65%)
**Headache**	1	19/80 (24%, 15%-35%)	32/80 (40%, 29%-52%)	23/80 (29%, 19%-40%)	6/80 (8%, 3%-16%)	0/80 (0%, 0%-5%)	61/80 (76%, 65%-85%)
	2	7/15 (47%, 21%-73%)	5/15 (33%, 12%-62%)	3/15 (20%, 4%-48%)	0/15 (0%, 0%-22%)	0/15 (0%, 0%-22%)	8/15 (53%, 27%-79%)

Symptom	Dose	None	Mild	Moderate	Severe	Potentially life threatening/ hospitalisation	Any
	3	43/80<br>(54%, 42%-65%)	29/80<br>(36%, 26%-48%)	8/80<br>(10%, 4%-19%)	0/80<br>(0%, 0%-5%)	0/80<br>(0%, 0%-5%)	37/80<br>(46%, 35%-58%)
**Malaise**	1	27/80<br>(34%, 24%-45%)	27/80<br>(34%, 24%-45%)	22/80<br>(28%, 18%-39%)	4/80<br>(5%, 1%-12%)	0/80<br>(0%, 0%-5%)	53/80<br>(66%, 55%-76%)
	2	12/15<br>(80%, 52%-96%)	1/15<br>(7%, 0%-32%)	1/15<br>(7%, 0%-32%)	1/15<br>(7%, 0%-32%)	0/15<br>(0%, 0%-22%)	3/15<br>(20%, 4%-48%)
	3	54/80<br>(68%, 56%-78%)	22/80<br>(28%, 18%-39%)	3/80<br>(4%, 1%-11%)	1/80<br>(1%, 0%-7%)	0/80<br>(0%, 0%-5%)	26/80<br>(32%, 22%-44%)
**Nausea**	1	59/80<br>(74%, 63%-83%)	14/80<br>(18%, 10%-28%)	7/80<br>(9%, 4%-17%)	0/80<br>(0%, 0%-5%)	0/80<br>(0%, 0%-5%)	21/80<br>(26%, 17%-37%)
	2	12/15<br>(80%, 52%-96%)	1/15<br>(7%, 0%-32%)	1/15<br>(7%, 0%-32%)	1/15<br>(7%, 0%-32%)	0/15<br>(0%, 0%-22%)	3/15<br>(20%, 4%-48%)
	3	72/80<br>(90%, 81%-96%)	7/80<br>(9%, 4%-17%)	1/80<br>(1%, 0%-7%)	0/80<br>(0%, 0%-5%)	0/80<br>(0%, 0%-5%)	8/80<br>(10%, 4%-19%)

Supplementary Table 7 - Overall summary of local and solicited adverse reactions in the first 7 days after vaccine for the third dose cohort

Symptom	Dose	None	Any	Mild	Moderate	Severe	Moderate or severe	>2 moderate/severe symptoms
**Any**	1	2/80 (2%, 0%-9%)	78/80 (98%, 91%-100%)	29/80 (36%, 26%-48%)	37/80 (46%, 35%-58%)	12/80 (15%, 8%-25%)	49/80 (61%, 50%-72%)	28/80 (35%, 25%-46%)
	2	3/15 (20%, 4%-48%)	12/15 (80%, 52%-96%)	8/15 (53%, 27%-79%)	3/15 (20%, 4%-48%)	1/15 (7%, 0%-32%)	4/15 (27%, 8%-55%)	2/15 (13%, 2%-40%)
	3	7/80 (9%, 4%-17%)	73/80 (91%, 83%-96%)	52/80 (65%, 54%-75%)	20/80 (25%, 16%-36%)	1/80 (1%, 0%-7%)	21/80 (26%, 17%-37%)	7/80 (9%, 4%-17%)
**Local**	1	11/80 (14%, 7%-23%)	69/80 (86%, 77%-93%)	57/80 (71%, 60%-81%)	11/80 (14%, 7%-23%)	1/80 (1%, 0%-7%)	12/80 (15%, 8%-25%)	0/80 (0%, 0%-5%)
	2	5/15 (33%, 12%-62%)	10/15 (67%, 38%-88%)	10/15 (67%, 38%-88%)	0/15 (0%, 0%-22%)	0/15 (0%, 0%-22%)	0/15 (0%, 0%-22%)	0/15 (0%, 0%-22%)
	3	15/80 (19%, 11%-29%)	65/80 (81%, 71%-89%)	56/80 (70%, 59%-80%)	9/80 (11%, 5%-20%)	0/80 (0%, 0%-5%)	9/80 (11%, 5%-20%)	0/80 (0%, 0%-5%)
**Systemic**	1	7/80 (9%, 4%-17%)	73/80 (91%, 83%-96%)	25/80 (31%, 21%-43%)	37/80 (46%, 35%-58%)	11/80 (14%, 7%-23%)	48/80 (60%, 48%-71%)	27/80 (34%, 24%-45%)
	2	5/15 (33%, 12%-62%)	10/15 (67%, 38%-88%)	6/15 (40%, 16%-68%)	3/15 (20%, 4%-48%)	1/15 (7%, 0%-32%)	4/15 (27%, 8%-55%)	2/15 (13%, 2%-40%)
	3	18/80 (22%, 14%-33%)	62/80 (78%, 67%-86%)	45/80 (56%, 45%-67%)	16/80 (20%, 12%-30%)	1/80 (1%, 0%-7%)	17/80 (21%, 13%-32%)	4/80 (5%, 1%-12%)

Supplementary Table 8 – Model estimated geometric mean antibody responses assessed by tIgG ELISA after a single dose, from regression analysis shown in Figure 2A

Days from V1	Geometric mean (95% CI)	Geometric mean ratio compared with day 28 (95% CI)	Adjusted* geometric mean ratio compared with day 28 (95% CI)
28	198.5 (194.3, 202.9)	ref	ref
90	150.6 (140.5, 161.5)	0.76 (0.72, 0.80)	0.76 (0.72, 0.79)
180	100.9 (87.82, 116.0)	0.51 (0.45, 0.57)	0.50 (0.45, 0.57)
320	54.13 (42.27, 69.32)	0.27 (0.22, 0.34)	0.27 (0.21, 0.34)

*adjusted for age at enrolment

Supplementary Table 9 – Model estimated geometric mean T-cell responses assessed by IFNγ ELISpot after a single dose, from regression analysis shown in Figure 2B

Days from V1	Geometric mean (95% CI)	Geometric mean ratio compared with day 28 (95% CI)	Adjusted* geometric mean ratio compared with day 28 (95% CI)
28	490.4 (473.8, 507.4)	ref	ref
90	369.0 (330.5, 411.9)	0.75 (0.70, 0.81)	0.75 (0.70, 0.81)
180	244.2 (195.9, 304.3)	0.50 (0.41, 0.60)	0.50 (0.41, 0.60)

*adjusted for age at enrollment (age variable statistically insignificant, p=0.8990)

Supplementary Table 10 – Antibody responses assessed by tIgG ELISA after 2 doses.

***P* values shown for Kruskal-Wallis test when comparing three groups and Wilcoxon rank sum test with continuity correction when comparing two groups.**

*adjusted for age at enrolment

Time-point	V1-V2 interval	n	Median [IQR]	Range	GMT (95% CI)	GMR (95% CI)	*P* value	Adjusted* GMT (95% CI)	Adjusted* GMR (95% CI)	*P* value*
V1	8-12 weeks	112	1 [1, 2]	1 - 290	2 (1, 2)					
	15-25 weeks	116	1 [1, 4]	1 - 916	2 (2, 3)					
	44-45 weeks	30	1 [1, 1]	1 - 267	2 (1, 3)					
V1+28	8-12 weeks	113	215 [124, 505]	1 - 5471	236 (185, 300)					
	15-25 weeks	115	184 [122, 393]	13 - 7092	245 (195, 309)					
	44-45 weeks	30	183 [138, 369]	35 - 3289	217 (150, 312)					
V1+56	44-45 weeks	29	119 [75, 216]	1 - 504	94 (54, 163)					
V1+182	44-45 weeks	30	112 [67, 147]	16 - 439	100 (74, 135)					
V2	8-12 weeks	115	161 [97, 376]	1 - 2524	173 (142, 212)					
	15-25 weeks	114	123 [74, 255]	4 - 1865	138 (112, 171)					
	44-45 weeks	30	76 [41, 109]	6 - 291	66 (48, 91)					
V2+28	8-12 weeks	113	923 [525, 1764]	1 - 4699	919 (756, 1117)	ref	<0.0001	931 (756, 1147)	ref	<0.0001
	15-25 weeks	108	1860 [917, 4992]	109 - 38065	1986 (1578, 2499)	2.16 (1.60, 2.92)		1969 (1592, 2434)	2.11 (1.57, 2.85)	
	44-45 weeks	30	3738 [1824, 6625]	348 - 46683	3982 (2703, 5864)	4.33 (2.82, 6.66)		3909 (2613, 5849)	4.20 (2.66, 6.62)	
V2+90	8-12 weeks	54	493 [213, 995]	76 - 2726	469 (360, 611)					
	15-25 weeks	79	1784 [649, 3811]	53 - 18067	1534 (1157, 2034)					
V2+182	8-12 weeks	62	278 [166, 499]	38 - 1715	307 (248, 380)	ref	<0.0001	306.3 (237, 395)	ref	<0.0001
	15-25 weeks	64	1280 [458, 2009]	45 - 15558	1034 (779, 1372)	3.37 (2.37, 4.79)		1036 (805, 1331)	3.38 (2.36, 4.85)	

Supplementary Table 11 - Antibody responses to SARS-CoV-2 Spike (Victoria strain) and Spike of other variants assessed by multiplex MSD assay in participants receiving 2 doses 44-46 weeks apart.

Antigen	Time Point	n	Median [IQR]	Range	GMT (95% CI)	n paired*	*P* value*
SARS-CoV-2 Spike (Victoria)	V1+28	29	7093 [4297, 17236]	1579 -86914	8752 (5827, 13144)		
	V2	29	2167 [1211, 3522]	473 - 19566	2078 (1532, 2820)		
	V2+28	29	116758 [70316, 160414]	12111 - 420027	106929 (78743, 145206)	29	< 0.0001
D614G Spike	V1+28	29	7106 [4672, 14668]	1355 - 99692	9012 (5976, 13591)		
	V2	29	2697 [1812, 3647]	448 - 24893	2516 (1861, 3402)		
	V2+28	29	111967 [67682, 149049]	11497 - 417632	101452 (74723, 137743)	29	< 0.0001
B.1.1.7 Spike (alpha)	V1+28	29	5651 [3283, 12191]	765 - 62051	6034 (4044, 9004)		
	V2	29	1539 [1134, 2245]	161 - 5418	1478 (1102, 1982)		
	V2+28	29	81600 [49757, 119309]	6902 - 327702	75020 (54795, 102710)	29	< 0.0001
P.1 Spike (gamma)	V1+28	29	4062 [2512, 8059]	562- 56681	4484 (2936, 6848)		
	V2	29	1298 [574, 2098]	130 - 5917	1131 (803, 1594)		
	V2+28	29	71093 [44260, 160209]	7234 - 260694	63376 (46638, 86122)	29	< 0.0001
B.1.351 Spike (beta)	V1+28	29	3073 [2190, 6373]	690 - 45433	3868 (2580, 5798)		
	V2	29	1111 [713, 1848]	103 - 3196	1067 (781, 1456)		
	V2+28	29	50315 [29210, 76079]	3831 - 182596	45478 (33061, 62558)	29	< 0.0001

*Statistics from Wilcoxon signed rank tests using V2 as the reference time-point

Supplementary Table 12 - Antibody responses assessed by tIgG ELISA in participants receiving 3 doses.

Time-point	V1-V2 interval	n	Median [IQR]	Range	GMT (95% CI)	n paired†	*P* value†
V1	8-16 weeks	75	2 [1, 5]	1 - 52	2 (2, 3)		
V1+28	8-16 weeks	74	192 [126, 504]	8 - 7092	254 (191, 337)		
V2	8-16 weeks	73	180 [84, 454]	4 - 1865	187 (141, 247)		
V2+28	8-16 weeks	73	1792 [899, 4634]	128 - 38065	1926 (1465, 2534)	73	ref
V3	8-16 weeks	75	555 [243, 1172]	36 - 6736	543 (419, 704)		
V3+14	8-16 weeks	74	2225 [1237, 4292]	47 - 7952	2007 (1615, 2494)		
V3+28	8-16 weeks	73	3746 [2047, 6420]	256 - 15865	3495 (2833, 4312)	73	0.0043

† Statistics from Wilcoxon signed rank tests using V2+28 as the reference time-point

Supplementary Table 13 - Antibody response to Beta variant (B.1.351) as assessed by tIgG ELISA in participants receiving 2 doses 44-46 weeks apart or 3 doses

Regimen	Time Point	n	Median [IQR]	Range	GMT (95% CI)	n paired*	*P* value*	n paired†	*P* value†
2 doses 44-46 weeks apart	V1+28	29	201 [129 – 448]	41 – 1747	233 (160, 339)			29	< 0.0001
	V2	30	105 [63 – 163]	1 – 322	79 (51, 122)				
	V2+28	30	3364 [2330 -5802]	152 - 21798	3429 (2316, 5078)				ref
3 doses	V2	45	122 [68 – 235]	13 - 962	118 (89, 156)				
	V2+28	45	1427 [680 – 2673]	114 – 18418	1407 (983, 2013)				ref
	V3	45	233 [129 – 611]	26 – 1983	268 (195, 368)		ref		
	V3+28	45	2016 [1009 – 3319]	55 - 15433	1794 (1341, 2401)	45	< 0.0001	45	0.2669

*Statistics from Wilcoxon signed rank tests using V3 as the reference time-point

† Statistics from Wilcoxon signed rank tests using V2+28 as the reference time-point

Supplementary Table 14 – Neutralising antibody responses to three VoCs in participants receiving 3 doses.

P values shown for pairwise comparisons using Wilcoxon sign rank test using V2+28 as the reference time-point

Time-point	Variant	n	Median [IQR]	Range	GMT (95% CI)	n paired	*P* value
V2+28	B.1.1.7 / Alpha	45	319 [176, 591]	20 - 3503	279 (200, 389)		
V3+28	B.1.1.7 / Alpha	42	612 [351, 920]	77 - 2606	545 (426, 698)	42	0.0023
V2+28	B.1.351 / Beta	45	54 [10, 113]	10 - 601	43 (30, 61)		
V3+28	B.1.351 / Beta	41	184 [66, 312]	10 - 1189	118 (78, 179)	41	< 0.0001
V2+28	B.1.617.2 / Delta	45	97 [38, 135]	10 - 1130	78 (55, 110)		
V3+28	B.1.617.2 / Delta	41	221 [110 – 471]	10 - 1496	206 (149, 284)	41	< 0.0001

Supplementary Table 15 - IFN-γ ELISpot response to peptides spanning the SARS-CoV-2 spike vaccine insert in participants receiving three doses of ChAdOx1 nCov-19.

Time-point	n	Median [IQR]	Range	GMT (95% CI)	n paired	GMR (95% CI)	*P* value *	n paired	GMR (95% CI)	*P* value†
V2+14	14	347 [200, 894]	2948 - 14	393 (221, 697)						
V2+28	15	475 [307, 1087]	1343 - 15	452 (272, 749)				-	ref	-
V3	15	200 [127, 389]	993 - 15	235 (149, 369)	-	ref	-			
V3+14	15	264 [131, 452]	1060 - 15	255 (155, 420)	15	1.09 (0.78, 1.51)	0.5701			
V3+28	12	399 [314, 662]	1826 - 12	442 (296, 659)	12	1.73 (1.23, 2.43)	0.0121	12	0.79 (0.63, 1.00)	0.0597

*Statistics from Wilcoxon signed rank tests using V3 as the reference time-point

† Statistics from Wilcoxon signed rank tests using V2+28 as the reference time-point

Supplementary References

1. Ramasamy MN, Minassian AM, Ewer KJ, et al. Safety and immunogenicity of ChAdOx1 nCoV-19 vaccine administered in a prime-boost regimen in young and old adults (COV002): a single-blind, randomised, controlled, phase 2/3 trial. *Lancet* 2021; **396**(10267): 1979-93.

Group Authorship – Oxford COVID Vaccine Trial Group

Syed Adlou	Oxford Vaccine Group, Department of Paediatrics, University of Oxford, UK
Robert Aley	Oxford Vaccine Group, Department of Paediatrics, University of Oxford, UK
Aabidah Ali	NDM Experimental medicine, NDM Experimental Medicine, University of Oxford, Oxford
Rachel Anslow	Oxford Vaccine Group, Department of Paediatrics, University of Oxford, UK
Megan Baker	Jenner Institute, Nuffield Department of Medicine, University of Oxford, UK
Phillip Baker	Oxford Vaccine Group, Department of Paediatrics, University of Oxford, UK
Jordan R. Barrett	Jenner Institute, Nuffield Department of Medicine, University of Oxford, UK
Louise Bates	Oxford Vaccine Group, Department of Paediatrics, University of Oxford, UK
Kirsten Beadon	Oxford Vaccine Group, Department of Paediatrics, University of Oxford, UK
Rebecca Beckley	Oxford Vaccine Group, Department of Paediatrics, University of Oxford, UK
Jonathan Bell	Oxford Vaccine Group, Department of Paediatrics, University of Oxford, UK
Duncan Bellamy	Jenner Institute, Nuffield Department of Medicine, University of Oxford, UK
Amy Beveridge	Oxford Vaccine Group, Department of Paediatrics, University of Oxford, UK
Cameron Bissett	Jenner Institute, Nuffield Department of Medicine, University of Oxford, UK
Luke Blackwell	Oxford Vaccine Group, Department of Paediatrics, University of Oxford, UK
Heather Bletchly	Oxford Vaccine Group, Department of Paediatrics, University of Oxford, UK
Amy Boyd	Jenner Institute, Nuffield Department of Medicine, University of Oxford, UK
Alice Bridges-Webb	Oxford Vaccine Group, Department of Paediatrics, University of Oxford, UK
Charlie Brown	NDM Experimental medicine, NDM Experimental Medicine, University of Oxford, Oxford
Nicholas Byard	Jenner Institute, Nuffield Department of Medicine, University of Oxford, UK
Susana Camara	Oxford Vaccine Group, Department of Paediatrics, University of Oxford, UK
Liliana Cifuentes Gutierrez	NDORMS, Botnar Research Centre, Old Rd, Headington, University of Oxford, UK
Andrea M. Collins	Department of Clinical Sciences, Liverpool School of Tropical Medicine and Liverpool University Hospitals NHS Foundation Trust, Liverpool, UK
Rachel Cooper	Oxford Vaccine Group, Department of Paediatrics, University of Oxford, UK
Wendy E.M. Crocker	Jenner Institute, Nuffield Department of Medicine, University of Oxford, UK
Thomas C. Darton	Department of Infection, Immunity and Cardiovascular Disease, University of Sheffield and Department of Infection and Tropical Medicine, Sheffield Teaching Hospitals NHS Foundation Trust, UK
Hannah Davies	Jenner Institute, Nuffield Department of Medicine, University of Oxford, UK
Judith Davies	Oxford Vaccine Group, Department of Paediatrics, University of Oxford, UK
Tesfaye Demissie	Oxford Vaccine Group, Department of Paediatrics, University of Oxford, UK
Claudio Di Maso	Oxford Vaccine Group, Department of Paediatrics, University of Oxford, UK
Tanya Dinesh	Oxford Vaccine Group, Department of Paediatrics, University of Oxford, UK
Francesca R. Donnellan	Jenner Institute, Nuffield Department of Medicine, University of Oxford, UK
Alexander D. Douglas	Jenner Institute, Nuffield Department of Medicine, University of Oxford, UK
Rachael Drake-Brockman	Oxford Vaccine Group, Department of Paediatrics, University of Oxford, UK
Christopher J. A. J. ADuncan	Department of Infection and Tropical Medicine, Newcastle upon Tyne Hospitals NHS Foundation Trust; 2. Translational and Clinical Research Institute, Immunity and Inflammation Theme, Newcastle University
Sean C. Elias	Jenner Institute, Nuffield Department of Medicine, University of Oxford, UK
Katherine R. W. Emary	Oxford Vaccine Group, Department of Paediatrics, University of Oxford, UK
Mutjaba Ghulam Farooq	Oxford Vaccine Group, Department of Paediatrics, University of Oxford, UK

Saul N. Faust	NIHR Southampton Clinical Research Facility and Biomedical Research Centre, University Hospital Southampton NHS Foundation Trust, and Faculty of Medicine and Institute for Life Sciences, University of Southampton, Southampton, UK
Sally Felle	Oxford Vaccine Group, Department of Paediatrics, University of Oxford, UK
Daniela Ferreira	Department of Clinical Sciences, Liverpool School of Tropical Medicine, UK
Carla Ferreira Da Silva	Oxford Vaccine Group, Department of Paediatrics, University of Oxford, UK
Adam Finn	School of Population Health Sciences, University of Bristol and University Hospitals Bristol and Weston NHS Foundation Trust, UK
Karen J. Ford	Oxford Vaccine Group, Department of Paediatrics, University of Oxford, UK
Emma Francis	Oxford Vaccine Group, Department of Paediatrics, University of Oxford, UK
Julie Furze	Jenner Institute, Nuffield Department of Medicine, University of Oxford, UK
Michelle Fuskova	Jenner Institute, Nuffield Department of Medicine, University of Oxford, UK
Eva Galiza	St George's Vaccine Institute, St George's, University of London, UK
Ana Gibertoni Cruz	Nuffield Department of Population Health
Leila Godfrey	Jenner Institute, Nuffield Department of Medicine, University of Oxford, UK
Anna L. Goodman	Department of Infectious Diseases, Guy's and St Thomas' NHS Foundation Trust, St Thomas' Hospital, London, UK and MRC Clinical Trials Unit at University College London, UK
Catherine Green	Jenner Institute, Nuffield Department of Medicine, University of Oxford, UK
Christopher A. Green	NIHR/Wellcome Trust Clinical Research Facility, University Hospitals Birmingham NHS Foundation Trust and Institute of Microbiology & Infection, University of Birmingham, UK
Nicola Greenwood	Jenner Institute, Nuffield Department of Medicine, University of Oxford, UK
Daisy Harrison	Oxford Vaccine Group, Department of Paediatrics, University of Oxford, UK
Thomas C. Hart	Oxford Vaccine Group, Department of Paediatrics, University of Oxford, UK
Sophia Hawkins	Oxford Vaccine Group, Department of Paediatrics, University of Oxford, UK
Paul T. Heath	St George's Vaccine Institute, St George's, University of London, UK
Helen Hill	Department of Clinical Sciences, Liverpool School of Tropical Medicine, UK
Kushalinii Hillson	Oxford Vaccine Group, Department of Paediatrics, University of Oxford, UK
Bryn Horsington	Oxford Vaccine Group, Department of Paediatrics, University of Oxford, UK
Mimi M. Hou	Jenner Institute, Nuffield Department of Medicine, University of Oxford, UK
Elizabeth Howe	Oxford Vaccine Group, Department of Paediatrics, University of Oxford, UK
Nicola Howell	Oxford Vaccine Group, Department of Paediatrics, University of Oxford, UK
Carina Joe	Jenner Institute, Nuffield Department of Medicine, University of Oxford, UK
Elizabeth Jones	Oxford Vaccine Group, Department of Paediatrics, University of Oxford, UK
Mwila Kasanyinga	Oxford Vaccine Group, Department of Paediatrics, University of Oxford, UK
Jade Keen	Oxford Vaccine Group, Department of Paediatrics, University of Oxford, UK
Sarah Kelly	Oxford Vaccine Group, Department of Paediatrics, University of Oxford, UK
David Kerr	Oxford Vaccine Group, Department of Paediatrics, University of Oxford, UK
Liaquat Khan	Oxford Vaccine Group, Department of Paediatrics, University of Oxford, UK
Baktash Khozoee	Jenner Institute, Nuffield Department of Medicine, University of Oxford, UK
Jasmin Kinch	Oxford Vaccine Group, Department of Paediatrics, University of Oxford, UK
Patrick Kinch	Oxford Vaccine Group, Department of Paediatrics, University of Oxford, UK
Stanislava Koleva	Oxford Vaccine Group, Department of Paediatrics, University of Oxford, UK
Jonathan Kwok	NDM Experimental medicine
Colin W. Larkworthy	Jenner Institute, Nuffield Department of Medicine, University of Oxford, UK
Alison M. Lawrie	Jenner Institute, Nuffield Department of Medicine, University of Oxford, UK

Rajeka Lazarus	Severn Pathology, North Bristol NHS Trust
Emily A. Lees	Oxford Vaccine Group, Department of Paediatrics, University of Oxford, UK
Grace Li	Oxford Vaccine Group, Department of Paediatrics, University of Oxford, UK
Vincenzo Libri	NIHR UCLH Clinical Research Facility and NIHR UCLH Biomedical Research Centre, London, UK
Patrick J. Lillie	Hull University Teaching Hospitals NHS Trust and Hull York Medical School, UK
Aline Linder	Oxford Vaccine Group, Department of Paediatrics, University of Oxford, UK
Fei Long	Oxford Vaccine Group, Department of Paediatrics, University of Oxford, UK
Raquel Lopez Ramon	Jenner Institute, Nuffield Department of Medicine, University of Oxford, UK
Reece Mabbett	Jenner Institute, Nuffield Department of Medicine, University of Oxford, UK
Rebecca Makinson	Jenner Institute, Nuffield Department of Medicine, University of Oxford, UK
Spyridoula Marinou	Oxford Vaccine Group, Department of Paediatrics, University of Oxford, UK
Emma Marlow	Jenner Institute, Nuffield Department of Medicine, University of Oxford, UK
Julia L. Marshall	Jenner Institute, Nuffield Department of Medicine, University of Oxford, UK
Olga Mazur	Oxford Vaccine Group, Department of Paediatrics, University of Oxford, UK
Joanne McEwan	Oxford Vaccine Group, Department of Paediatrics, University of Oxford, UK
Alastair C. McGregor	London North West University Healthcare NHS Trust and the Department of Medicine, Imperial College London, UK
Jolynne Mokaya	Jenner Institute, Nuffield Department of Medicine, University of Oxford, UK
Ella Morey	Oxford Vaccine Group, Department of Paediatrics, University of Oxford, UK
Gertraud Morshead	Oxford Vaccine Group, Department of Paediatrics, University of Oxford, UK
Richard Morter	Jenner Institute, Nuffield Department of Medicine, University of Oxford, UK
Jilly Muller	Oxford Vaccine Group, Department of Paediatrics, University of Oxford, UK
Philomena Mweu	Oxford Vaccine Group, Department of Paediatrics, University of Oxford, UK
Rabiullah Noristani	Jenner Institute, Nuffield Department of Medicine, University of Oxford, UK
Nelly Owino	Oxford Vaccine Group, Department of Paediatrics, University of Oxford, UK
Marco Polo Peralta Alvarez	Jenner Institute, Nuffield Department of Medicine, University of Oxford, UK
Abigail Platt	Jenner Institute, Nuffield Department of Medicine, University of Oxford, UK
Katrina M. Pollock	NIHR Imperial Clinical Research Facility and NIHR Imperial Biomedical Research Centre, London, UK
Ian Poulton	Jenner Institute, Nuffield Department of Medicine, University of Oxford, UK
Samuel Provstgaard-Morys	Oxford Vaccine Group, Department of Paediatrics, University of Oxford, UK
David Pulido-Gomez	Jenner Institute, Nuffield Department of Medicine, University of Oxford, UK
Matthew Rajan	Oxford Vaccine Group, Department of Paediatrics, University of Oxford, UK
Fernando Ramos Lopez	Jenner Institute, Nuffield Department of Medicine, University of Oxford, UK
Adam Ritchie	Jenner Institute, Nuffield Department of Medicine, University of Oxford, UK
Hannah Roberts	Oxford Vaccine Group, Department of Paediatrics, University of Oxford, UK
Christine Rollier	Oxford Vaccine Group, Department of Paediatrics, University of Oxford, UK
Indra Rudiansyah	Jenner Institute, Nuffield Department of Medicine, University of Oxford, UK
Katherine Sanders	Oxford Vaccine Group, Department of Paediatrics, University of Oxford, UK
Jack E. Saunders	Jenner Institute, Nuffield Department of Medicine, University of Oxford, UK
Samiullah Seddiqi	Oxford Vaccine Group, Department of Paediatrics, University of Oxford, UK
Hannah R. Sharpe	Jenner Institute, Nuffield Department of Medicine, University of Oxford, UK
Robert Shaw	Oxford Vaccine Group, Department of Paediatrics, University of Oxford, UK
Laura Silva-Reyes	Oxford Vaccine Group, Department of Paediatrics, University of Oxford, UK
Nisha Singh	Oxford Vaccine Group, Department of Paediatrics, University of Oxford, UK

David J. Smith	Oxford Vaccine Group, Department of Paediatrics, University of Oxford, UK
Catherine C. Smith	Oxford Vaccine Group, Department of Paediatrics, University of Oxford, UK
Andrew Smith	College of Medical, Veterinary & Life Sciences, Glasgow Dental Hospital & School, University of Glasgow, UK
Alexandra J. Spencer	Jenner Institute, Nuffield Department of Medicine, University of Oxford, UK
Arabella S. V. Stuart	Oxford Vaccine Group, Department of Paediatrics, University of Oxford, UK
Rebecca Sutherland	Clinical Infection Research Group, Regional Infectious Diseases Unit, NHS Lothian, Edinburgh, UK
Anna Szigeti	Oxford Vaccine Group, Department of Paediatrics, University of Oxford, UK
Karly Tang	Oxford Vaccine Group, Department of Paediatrics, University of Oxford, UK
Merin Thomas	Jenner Institute, Nuffield Department of Medicine, University of Oxford, UK
Tonia M. Thomas	Oxford Vaccine Group, Department of Paediatrics, University of Oxford, UK
Amber Thompson	Oxford Vaccine Group, Department of Paediatrics, University of Oxford, UK
Emma C. Thomson	MRC - University of Glasgow Centre for Virus Research & Department of Infectious Diseases, Queen Elizabeth University Hospital, Glasgow, UK
Estée M. Török	Cambridge University Hospitals NHS Foundation Trust
Mark Toshner	Heart Lung Research Institute, Dept of Medicine, University of Cambridge<br>NIHR Cambridge Clinical Research Facility, Cambridge University Hospital and Royal Papworth NHS Trusts
Nguyen Tran	Jenner Institute, Nuffield Department of Medicine, University of Oxford, UK
Rose Trivett	Oxford Vaccine Group, Department of Paediatrics, University of Oxford, UK
Iain Turnbull	Clinical Trial Service Unit, , NDPH
Cheryl Turner	Jenner Institute, Nuffield Department of Medicine, University of Oxford, UK
David P. J. Turner	University of Nottingham and Nottingham University Hospitals NHS Trust, UK
Marta Ulaszewska	Jenner Institute, Nuffield Department of Medicine, University of Oxford, UK
Iason Vichos	Oxford Vaccine Group, Department of Paediatrics, University of Oxford, UK
Laura Walker	Oxford Vaccine Group, Department of Paediatrics, University of Oxford, UK
Marion E. Watson	Jenner Institute, Nuffield Department of Medicine, University of Oxford, UK
Conor Whelan	Oxford Vaccine Group, Department of Paediatrics, University of Oxford, UK
Rachel White	Oxford Vaccine Group, Department of Paediatrics, University of Oxford, UK
Sarah J. Williams	Oxford Vaccine Group, Department of Paediatrics, University of Oxford, UK
Christopher J. Williams	Aneurin Bevan University Health Board, Newport, Wales
Daniel Wright	Jenner Institute, Nuffield Department of Medicine, University of Oxford, UK
Andy Yao	Oxford Vaccine Group, Department of Paediatrics, University of Oxford, UK

Supplementary Acknowledgements

**Clinical Trials Research Governance Office, University of Oxford**
Elaine Chick
Claire Riddle
**Department of Paediatrics, University of Oxford**
Georg A. Holländer
**Jenner Institute, University of Oxford**
Iona Tarbet
**Nuffield Department of Medicine, University of Oxford**
Richard Cornall
Denis Murphy
Philip Taylor
Oto Velicka
**Oxford Research Services (Contracts)**
Sally Pelling-Deeves
Gary Priest
**Oxford University Hospitals Trust**
Bruno Holthof
**Public Affairs Directorate and Divisional Communication Team**
Alison Brindle
Alexander Buxton
James Colman
Steve Pritchard
